# Panaroma of microglia in traumatic brain injury: a bibliometric analysis and visualization study during 2000–2023

**DOI:** 10.3389/fncel.2024.1495542

**Published:** 2024-11-07

**Authors:** Yuhang Zhang, Tingzhen Deng, Xiao Ding, Xingyuan Ma, Yatao Wang, Haijun Yang, Ruiwen Ding, Dawen Wang, Haotian Li, Maohua Zheng

**Affiliations:** ^1^The First School of Clinical Medicine, Lanzhou University, Lanzhou, China; ^2^Department of Neurosurgery, The First Hospital of Lanzhou University, Lanzhou, China; ^3^Department of Neurosurgery, Armed Police Hospital of Chongqing, Chongqing, China

**Keywords:** traumatic brain injury, TBI, microglia, bibliometric analysis, CiteSpace, VOSviewer

## Abstract

**Background:**

Traumatic brain injury (TBI) is a critical global health concern characterized by elevated rates of both morbidity and mortality. The pathological and physiological changes after TBI are closely related to microglia. Microglia, the primary immune cells in the brain, are closely linked to the mechanisms and treatment of TBI. With increasing research in this area, this study employs bibliometric analysis to identify current research hotspots and predict future trends.

**Objective:**

We decided to perform a bibliometric analysis to provide a comprehensive overview of the advancements in microglia research related to traumatic brain injury. We aim to offer researchers insights into current trends and future research directions.

**Method:**

We collected all articles and reviews related to microglia and traumatic brain injury published between 2000 and 2023 from the Web of Science Core Collection. These records were analyzed using VOSviewer, CiteSpace, and the R package “bibliometrix”.

**Results:**

We retrieved 665 publications from 25 countries, with the majority contributed by the United States and China. The number of publications on traumatic brain injury and microglia has been steadily increasing each year. Our analysis highlighted the *Journal of Neurotrauma* and the *Journal of Neuroinflammation* as the most influential journals in this field. Alan I. Faden and David J. Loane are recognized as leading contributors. Keyword analysis indicates that neuroinflammation, microglial polarization, and neurodegenerative diseases are pivotal areas for future research.

**Conclusion:**

In recent years, research on TBI-related microglia has proliferated, with current studies primarily focusing on microglial involvement in neuroinflammation, neurodegenerative changes, and microglial polarization following TBI. Since neuroinflammation and neurodegeneration are two hallmark features of TBI, targeting microglia in TBI treatment may become a central focus for future research.

## 1 Introduction

Traumatic brain injury (TBI) is a leading neurological disorder and a significant public health issue. By 2030, it is anticipated to be among the top three causes of injury-related death and disability. TBI affects 50 to 60 million people annually, resulting in approximately $400 billion in economic losses (Maas et al., 2022). TBI inflicts immediate primary damage and exacerbates secondary damage through complications such as hypotension, hypoxia, hemorrhage, and neurotoxicity (Morganti-Kossmann et al., 2019). About half of TBI patients are unable to return to their previous employment within a year, and roughly 28% never return to work (James et al., 2019; Gage and Temple, 2013; Ma et al., 2009). Evidence suggests that TBI is not only an acute event but also a chronic condition with enduring long-term consequences, including post-traumatic stress disorder, memory impairments, chronic traumatic encephalopathy, persistent neuroinflammation, and delayed neurodegeneration (Maas et al., 2022; Dams-O'Connor et al., 2023; Simon et al., 2017).

Microglia are resident cells in the adult mammalian brain, originating from the embryonic yolk sac, and makeup 10–15% of all glial cells (Ginhoux et al., 2010; Li et al., 2022). These highly active cells maintain brain homeostasis by clearing metabolic waste and tissue debris through phagocytosis (Davalos et al., 2005). They are crucial for brain development, activity-dependent synaptic plasticity, and central nervous system (CNS) learning by regulating synaptic pruning, cell death and neurogenesis (Salter and Beggs, 2014; Katsumoto et al., 2014; Tremblay et al., 2011). After TBI, microglia can adopt different states and perform various functions. Recent research has extensively investigated these roles, and this review summarizes the current understanding of microglial mechanisms, functions, and potential therapeutic targets in TBI, aiming to guide future research and highlight emerging trends.

Bibliometric analysis effectively examines publications in a specific field, focusing on elements like countries, institutions, authors, and keywords to reveal critical characteristics and trends (Zhu et al., 2023). The process involves three main steps: retrieving and cleaning relevant literature, analyzing the data from various angles using specialized software, and writing the manuscript to conclude. To date, there has been no bibliometric analysis explicitly focused on microglia in the context of traumatic brain injury. Therefore, we aim to fill this gap and provide insights into the field's development and future research directions.

## 2 Methods

### 2.1 Data collection

**Figure 1:**
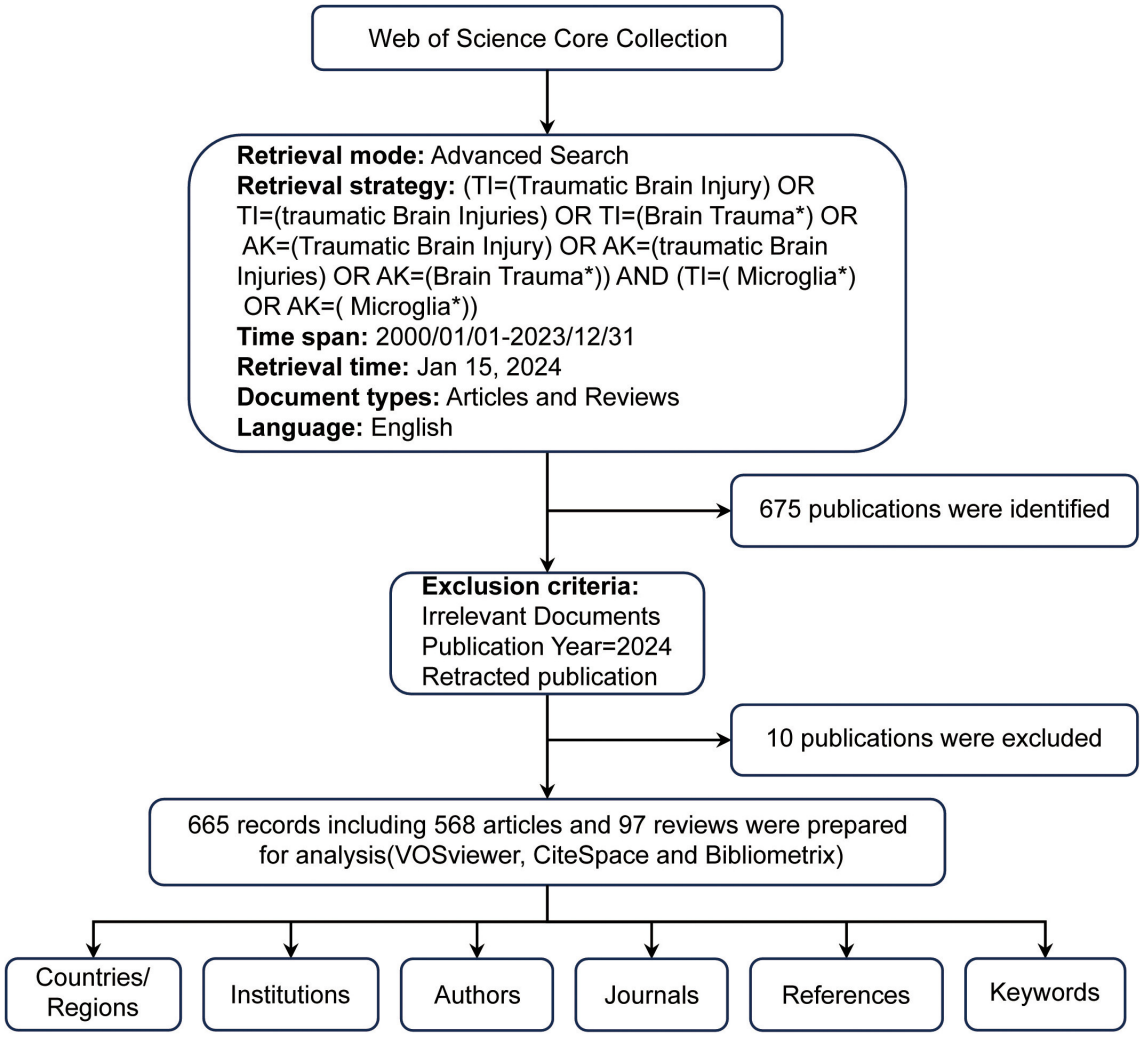
Flowchart of Publication Search and Selection Process.

The Web of Science Core Collection (WoSCC) was the primary database for retrieving data. As one of the largest and most comprehensive online databases, WoSCC provides a vast array of authoritative scientific research and analysis (Zhang et al., 2024). It provides vital information, including title, country/region, institution, author, keywords, and references (Deng et al., 2024). For our study, the search formula was: [TI= (Traumatic Brain Injury) OR TI= (Traumatic Brain Injuries) OR TI= (Brain Trauma^*^) OR AK= (Traumatic Brain Injury) OR AK= (Traumatic Brain Injuries) OR AK= (Brain Trauma^*^)] AND [TI= (Microglia^*^) OR AK= (Microglia^*^)]. We considered only English-language publications categorized as “articles” or “reviews” from January 1, 2000, to December 31, 2023. This search retrieved 675 documents, consisting of 578 articles and 97 reviews. To avoid bias due to database updates, all searches and downloads were finalized on May 21, 2024. Two independent researchers then manually reviewed the titles, abstracts, and full texts to ensure relevance, excluding irrelevant studies based on disease type, research focus, animal models, interventions, cell types, and outcome measures. We excluded ten records, including meeting abstracts, irrelevant literature and retracted publications, resulting in 665 valid documents. These were exported in TXT format, labeled as “full record and cited references” for further examination (Figure 1).

### 2.2 Data analysis

We employed three software tools for data analysis: R software (version 4.4.1), VOSviewer (version 1.6.19), and CiteSpace (version 6.3.1). Using the R package “bibliometrix” (version 3.2.1), we performed a detailed analysis of the thematic evolution and global distribution network of the literature (Aria and Cuccurullo, 2017). The application of R software is straightforward. After entering the relevant code, users can access a working environment. Once the raw data is imported, analyses can be performed using the options in the left-hand directory. VOSviewer, a complimentary software created by Van Eck and Waltman, was used to analyze countries, authors, journals, institutions, and keywords found in the collected literature (van Eck and Waltman, 2010; Pan et al., 2018). We import the cleaned data into the software, choose the analysis type, and specify the research subjects. Then, we apply filters to refine the node density, ultimately generating a clear visual representation of the results. In VOSviewer, node sizes reflect the number of citations, publications, or occurrences, while links between nodes denote relationships such as co-citation, co-authorship, or co-occurrence. Node and link colors indicate different clusters or average appearance years (AAY), and line thickness represents the level of collaboration or co-citation (Deng et al., 2024). Additionally, CiteSpace, a widely recognized bibliometric tool, provided insights into research hotspots and trends, enabling us to predict future development directions (Synnestvedt et al., 2005). We used CiteSpace to analyze keyword and citation bursts in the literature.

## 3 Results

### 3.1 Publication volume and trends

**Figure 2:**
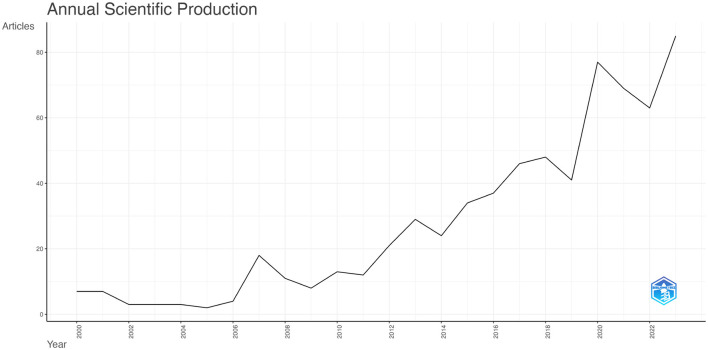
Annual Publication Trends in Traumatic Brain Injury and Microglia. The curve illustrates the annual trend in the number of publications.

The volume of published documents can indicate the level of research activity and progress within a field (Zhang et al., 2024). Figure 2 illustrates the number of publications from 2000 to 2023, highlighting 665 articles related to TBI and microglia, including 568 research articles and 97 reviews. From 2000 to 2011, the annual publication volume increased gradually but consistently, though it stayed within 20 publications per year. This indicates that the field received relatively little attention during this period. In contrast, from 2012 to 2023, there was a noticeable increase in annual publications, highlighting the growing prominence of this research area as a hotspot from 2012 onwards.

### 3.2 Most productive and influential countries

**Figure 3:**
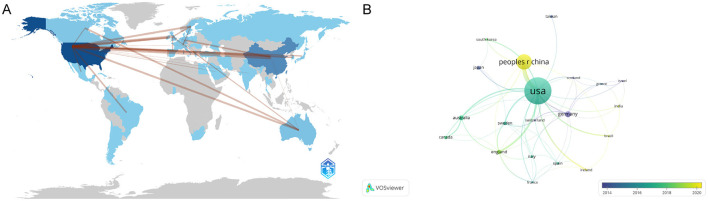
**(A)** Geographic Distribution Map of Global Publications. The varying shades of blue reflect the volume of publications from each country, while gray signifies countries with no publications. The lines in the diagram illustrate the connections between countries/regions. **(B)** Geographical Distribution and Collaboration Network of Countries/Regions. Each node represents a country or region, with connecting lines illustrating their collaborative relationships. The thickness of these lines denotes the strength of the collaborations. Additionally, the color of each node reflects its position on the timeline, indicating the periods during which it participated in collaborative activities with other nodes.

Figure 3A illustrates the distribution of publications related to traumatic brain injury and microglia by country and region. Darker colors indicate a higher volume of publications, while the lines connecting different regions reflect their collaborative relationships. We observe that the majority of research is concentrated in the Northern Hemisphere, where countries engage in close cooperation. In contrast, Australia is the only active participant in this field in the Southern Hemisphere, maintaining some exchanges with other nations. Table 1A presents the top 10 countries or regions ranked by publication volume. The United States tops the list with 299 papers (45%), while China follows with 158 papers (24%). These two countries collectively contribute to nearly half of the publications, underscoring their prominent role. Germany (33 papers, 5%), Australia (25 papers, 3.8%), and Japan (18 papers, 2.7%) also play prominent roles. Regarding overall connection strength, the top five are the United States (1,641), the United Kingdom (865), Germany (828), China (506), and Spain (415). To explore collaboration among countries, we visualized a co-authorship network using VOSviewer (Figure 3B). This analysis highlights strong collaboration among numerous countries, particularly close ties between China and the United States. The nodes representing each country are color-coded according to the average year of appearance (AAY). Germany and Japan, shown in bluish colors, represent early contributors. At the same time, China and several other countries in yellow are newer participants in microglia research in traumatic brain injury.

**Table 1:** The top 10 countries and institutions with the most publications in the field on TBI and microglia.

**Rank**	**Part A**			**Part B**	
	**Country/region**	**Count**	**Percent**	**Institutions**	**Count**
1	United States	299	45%	University of Maryland Baltimore	76
2	China	158	24%	University Of Pittsburgh	73
3	Germany	33	5%	University of California System	69
4	Australia	25	3.8%	Pennsylvania Commonwealth System Of Higher Education (PCSHE)	64
5	Japan	18	2.7%	University of California San Francisco	55
6	United Kingdom	18	2.7%	Ohio State University	51
7	Canada	14	2.1%	University System Of Maryland	46
8	Sweden	13	2%	University System of Ohio	43
9	Korea	10	1.5%	Veterans Health Administration (VHA)	43
10	Italy	9	1.4%	US Department Of Veterans Affairs	32

### 3.3 Analysis of affiliations

Eight hundred and thirty four institutions have participated in the 665 studies on microglia in traumatic brain injury. Table 1B lists the top 10 institutions by publication volume, all of which are based in the United States, highlighting the significant role that American institutions play in this field. Of these, six institutions have each published over 50 articles. Specifically, University of Maryland, Baltimore emerged as the most prolific institution (76, 9.1%), followed by the University of Pittsburgh (73, 8.7%), University of California (69, 8.2%), and Pennsylvania State System of Higher Education (64, 7.6%).

**Figure 4:**
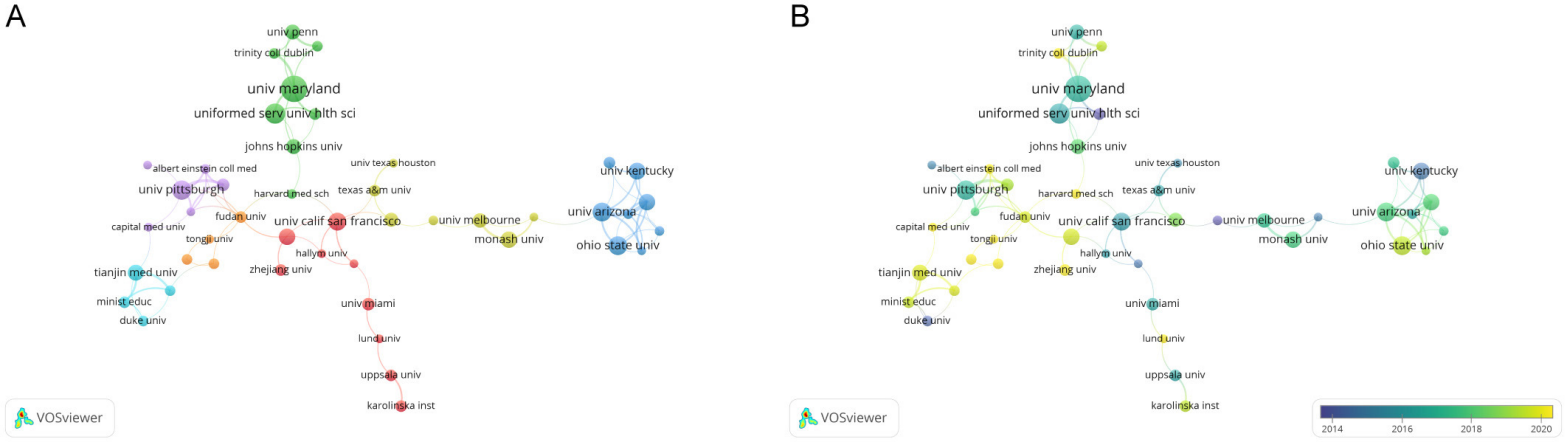
The network map of institutions. **(A)** The network visualization map of Institution co-authorship analysis conducted by VOSviewer. **(B)** The overlay visualization map of Institution co-authorship analysis conducted by VOSviewer.

Figure 4A presents a clustering analysis of institutions, visually examining the collaboration network using VOSviewer. Based on the colors of the nodes, institutions are categorized into seven clusters, primarily centered around well-known institutions such as the University of Maryland, Baltimore, the University of Pittsburgh, and the University of California, San Francisco. Articles within the same cluster often exhibit similar research directions or underlying logic. However, connections among institutions tend to be stronger within the same cluster, while inter-cluster links are relatively sparse, relying on only one or two institutions for connections. In Figure 4B, American institutions such as the University of Maryland, Baltimore, the University of Pittsburgh, and the University of California, San Francisco, are predominantly blue, signifying their early involvement in this research area. In contrast, Chinese institutions like Tianjin University, Fudan University, and Capital Medical University are highlighted in yellow, indicating a recent surge in engagement or a notable increase in publication output in recent years.

### 3.4 Analysis of core journals

**Figure 5:**
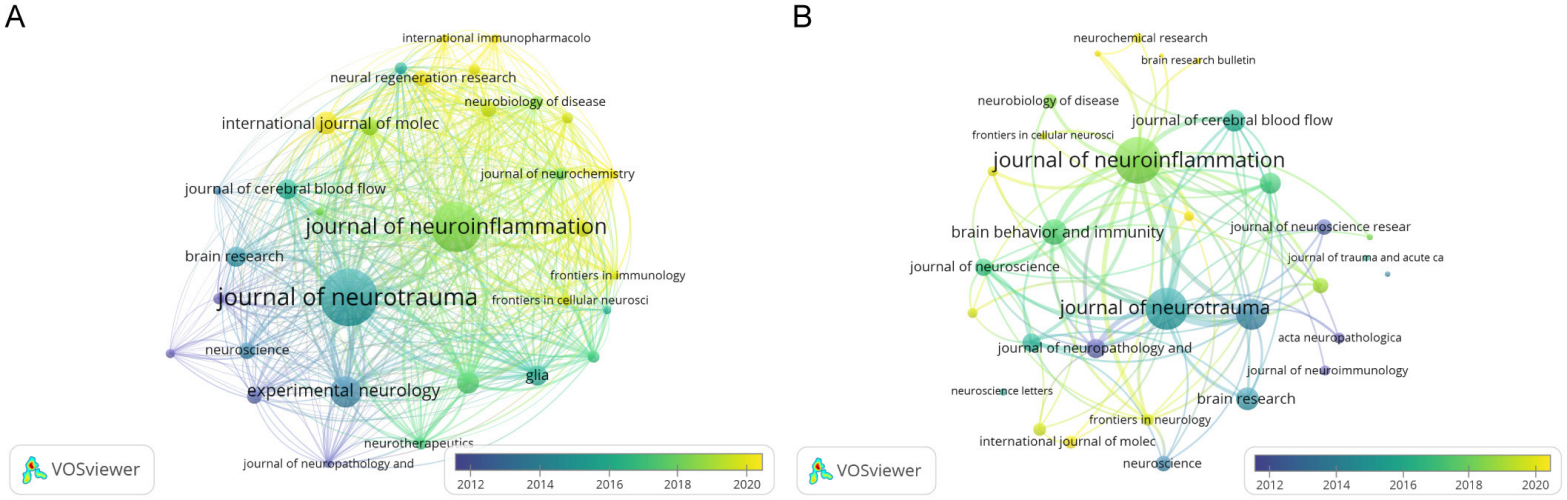
The network map of journal. **(A)** The overlay visualization map of journal coupling analysis conducted by VOSviewer. **(B)** The overlay visualization map of journal citation analysis conducted by VOSviewer.

We identified 665 articles on microglia in traumatic brain injury published in 215 journals. Table 2 details this field's top 10 most productive and most cited journals. The *Journal of Neurotrauma* leads with the highest number of publications (59, 8.87%), followed by the *Journal of Neuroinflammation* (49, 7.37%) and *Experimental Neurology* (26, 3.91%). Over half of the top 10 journals by publication volume are classified in Q1. The journal with the highest Impact Factor (IF) is the *Journal of Neuroinflammation*, with an IF of 9.3. We also analyzed 32 journals with more than five publications and created a journal coupling map using VOSviewer. The *Journal of Neurotrauma* exhibits strong coupling relationships with the *Journal of Neuroinflammation, Experimental Neurology*, and others (Figure 5A). The map also shows each journal's appearance year (AAY), indicated by different colors. Among the top 10 cited journals, two-thirds are classified in Q1. Five journals have been cited more than 1,000 times, with the *Journal of Neuroinflammation* being the most cited (3,095 times), highlighting its significant impact on research in microglia and traumatic brain injury. It is followed by the *Journal of Neurotrauma* (2,601 citations) and *Experimental Neurology* (1,678 citations). Figure 5B illustrates that the *Journal of Neuroinflammation* has active citation relationships with the *Journal of Neurotrauma, Brain, Behavior, and Immunity* and the *Journal of Cerebral Blood Flow & Metabolism*.

**Table 2:** The top 10 productive journals and top 10 most cited journals.

**Rank**	**Most productive journals**	**Articles**	**JCR**	**Most cited journals**	**Citations**	**JCR**
1	Journal of Neurotrauma	59	Q1	Journal of Neuroinflammation	3085	Q1
2	Journal of Neuroinflammation	49	Q1	Journal of Neurotrauma	2601	Q1
3	Experimental Neurology	26	Q1	Experimental Neurology	1678	Q1
4	International Journal of Molecular Sciences	18	Q1	Brain, Behavior, and Immunity	1220	Q1
5	Brain, Behavior, and Immunity	17	Q1	Brain Research	1038	Q1
6	Brain Research	15	Q3	Journal of Cerebral Blood Flow & Metabolism	958	Q3
7	Glia	15	Q1	neurotherapeutics	906	Q1
8	Journal of Cerebral Blood Flow & Metabolism	15	Q1	Journal of Neuropathology & Experimental Neurology	802	Q2
9	Molecular Neurobiology	13	Q1	Glia	763	Q1
10	Frontiers in Neurology	12	Q2	Journal of Neuroscience	684	Q1

### 3.5 Analysis of the influential authors

**Figure 6:**
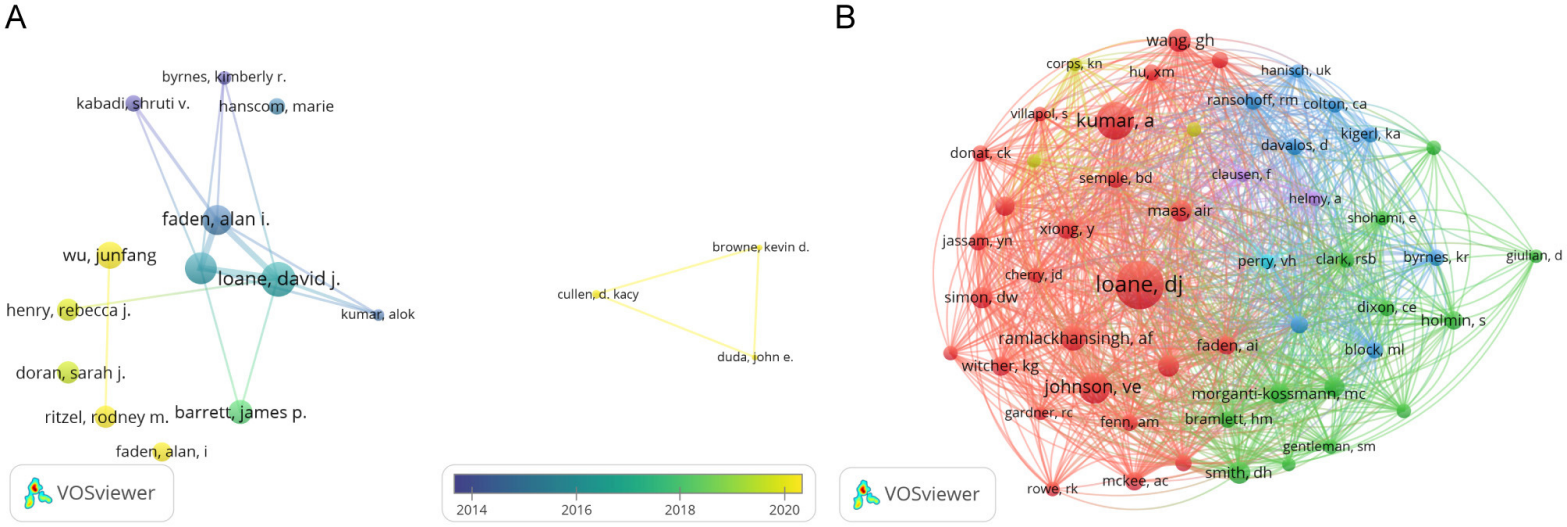
The network map of authors. **(A)** The overlay visualization map of author co-authorship analysis conducted by VOSviewer. **(B)** The network visualization map of co-cited authors analysis conducted by VOSviewer.

The volume of scientific publications an individual authored indicates their research activity and scholarly contributions to the field (Wu et al., 2021). We identified over 3,588 authors involved in research on microglia in traumatic brain injury. Table 3 presents the top 10 authors ranked by publication volume, citation count, and co-citation frequency. Alan I. Faden from the University of Maryland School of Medicine and David J. Loane from the University of Pittsburgh School of Medicine are the most prolific authors, with 27 publications. They are followed by Bogdan A. Stoica (20 publications) and Charles S. Cox Jr. (19 publications). Figure 6A, created using VOSviewer, displays a co-authorship map with three distinct clusters of researchers, each represented by different colors corresponding to different periods. The connections between these clusters are not very dense. Co-citation relationships, which occur when two authors or works are cited together in the reference lists of other publications, are commonly analyzed to identify prominent authors within a particular field. Table 3 shows that eight authors have been cited more than 100 times each. Figure 6B depicts the collaborative network among co-cited authors, highlighting that highly co-cited authors occupy central positions, linking multiple nodes within the network.

**Table 3:** The top 10 authors and co-cited authors involved in research on TBI and microglia.

**Rank**	**Highly Published Authors**	**Count**	**Highly Cited Authors**	**Citations**	**Co-Cited Authors**	**Citations**
1	Faden AI	27	Loane DJ	701	Loane DJ	360
2	Loane DJ	27	Kumar A	470	Kumar A	240
3	Stoica BA	20	Faden AI	458	Johnson VE	177
4	Cox CS	19	Stoica BA	393	Ramlackhansingh AF	130
5	Kumar A	16	Lifshitz J	185	Wang GH	111
6	Lifshitz J	15	Byrnes KR	168	Smith DH	105
7	Schluesener HJ	15	Sharp DJ	147	Ziebell JM	101
8	Byrnes KR	12	Brooks DJ	139	Simon DW	101
9	Godbout JP	12	Ramlackhansingh AF	139	Mass AIR	98
10	Bedi SS	11	Bose SK	130	Morganti-Kossmann MC	95

### 3.6 Publications and co-cited reference analysis

**Figure 7:**
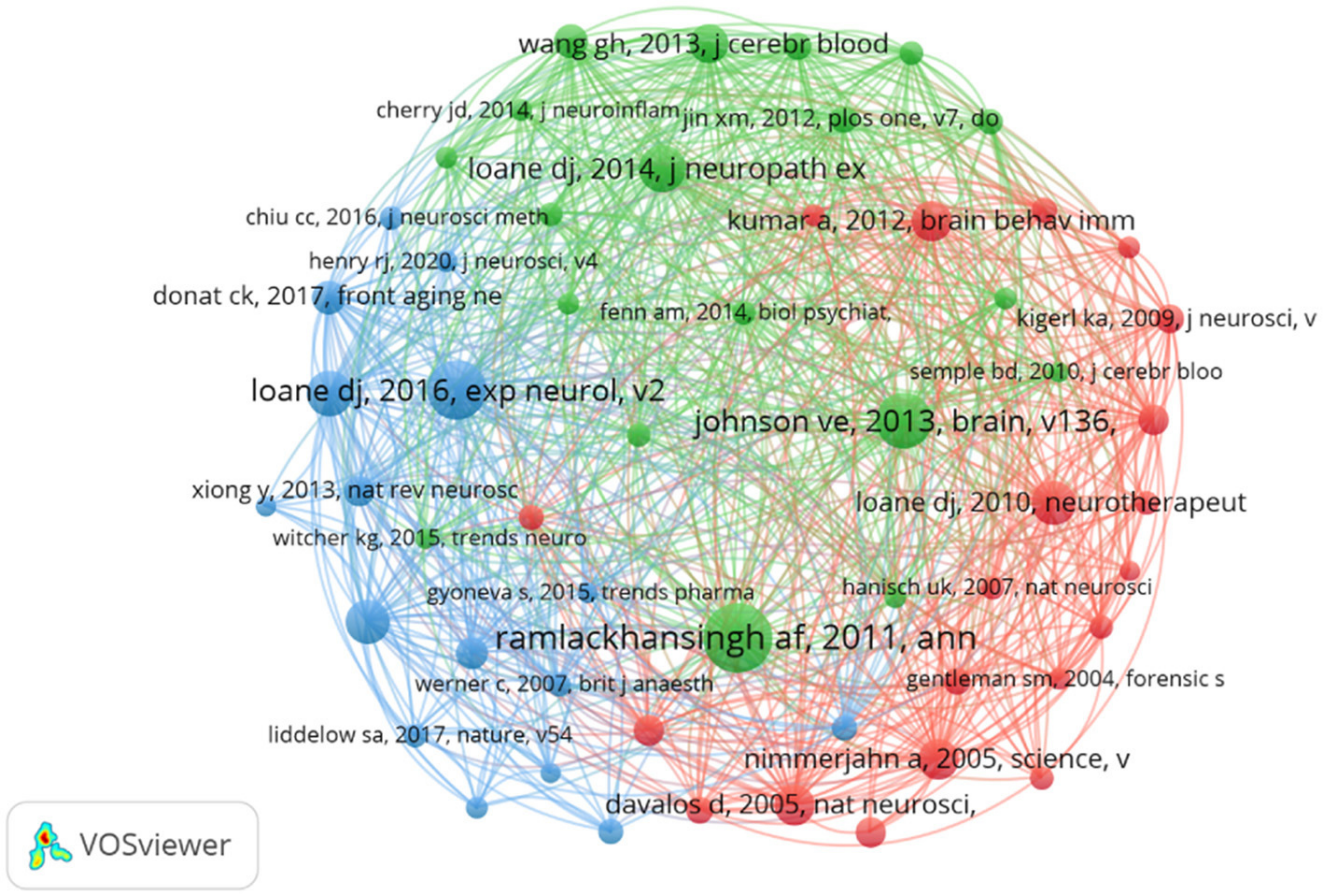
Network map of co-cited references.

Despite ongoing debates about the significance of citation rates (Fuster, 2017; Nieminen et al., 2006), the number of citations remains a fundamental gauge of academic influence (Chatterjee and Werner, 2021). Highly cited papers often reflect substantial academic interest and pinpoint emerging research hotspots within a field (Deng et al., 2024). Our statistical analysis of the 665 selected papers identified 77 that have been cited over 100 times. Table 4 summarizes the 10 papers with the highest citation counts. Most of these papers were published after 2010, including six review articles and four research papers. The most highly cited article, with 691 citations, is a 2013 study by Ballabh (Johnson et al., 2013), which explores the relationship between neuroinflammation and white matter degeneration following traumatic brain injury. The papers ranked second and third in citation count were authored by Ramlackhansingh and Gensel, respectively, and both address microglial activation following TBI (Gensel and Zhang, 2015; Ramlackhansingh et al., 2011). In summary, these 10 papers cover topics such as microglial polarization (Gensel and Zhang, 2015; Ramlackhansingh et al., 2011; Wang et al., 2013; Loane et al., 2014), neuroinflammation (Kumar and Loane, 2012; Lenzlinger et al., 2001) and neurodegenerative changes occurring after traumatic brain injury (Johnson et al., 2013; Wang et al., 2013). Furthermore, analyzing co-citations of references is a practical method to assess research trends and monitor progress in the field. Figure 7 displayed the network of co-cited references, revealing that the document authored by Ramlackhansingh in *Annals of Neurology* had the largest co-citation node size. The top-cited papers are intricately connected.

**Table 4:** Top 10 cited references of publications in TBI and stem cells.

**Rank**	**Title**	**Journal**	**First author**	**Year**	**Citations**	**Type**
1	Inflammation and white matter degeneration persist for years after a single traumatic brain injury	Brain	Johnson VE	2013	691	Article
2	Inflammation after trauma: microglial activation and traumatic brain injury	Ann Neurol	Ramlackhansingh AF	2011	681	Article
3	Macrophage activation and its role in repair and pathology after spinal cord injury	Brain Res	Gensel JC	2015	490	Review
4	Role of microglia in neurotrauma	Neurotherapeutics	Loane DJ	2010	474	Review
5	Neuroinflammation after traumatic brain injury: opportunities for therapeutic intervention	Brain Behav Immun	Kumar A	2012	472	Review
6	Microglia in the TBI brain: The good, the bad, and the dysregulated	Exp Neurol	Loane DJ	2016	444	Review
7	Progressive neurodegeneration after experimental brain trauma: association with chronic microglial activation	J Neuropath Exp Neur	Loane DJ	2014	340	Article
8	The duality of the inflammatory response to traumatic brain injury	Mol Neurobiol	Lenzlinger PM	2001	337	Review
9	Microglia/macrophage polarization dynamics in white matter after traumatic brain injury	J Cerebr Blood F Met	Wang GH	2013	334	Article
10	The contribution of astrocytes and microglia to traumatic brain injury	Brit J Pharmacol	Karve IP	2016	301	Review

### 3.7 Analysis of keywords

**Figure 8:**
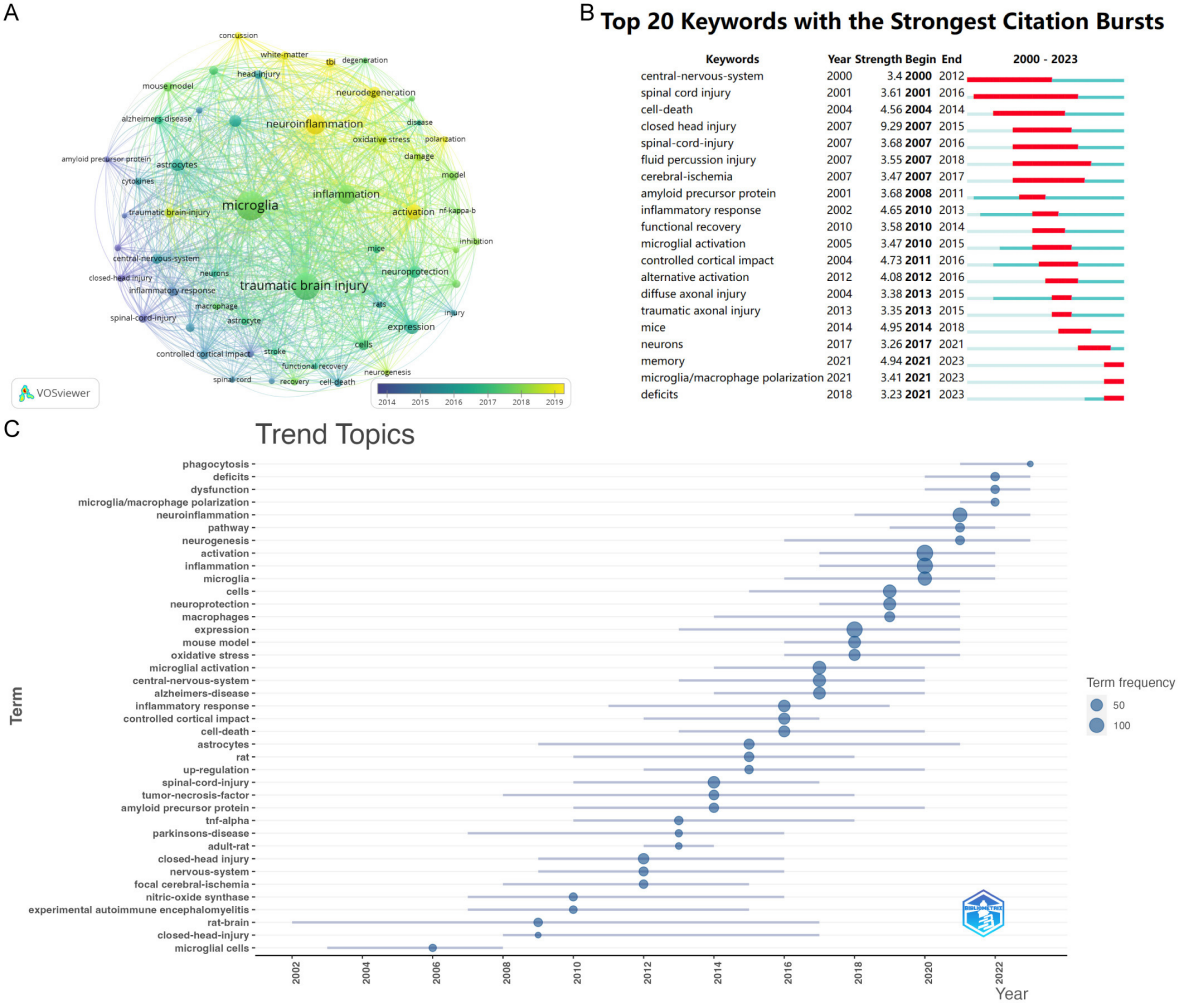
**(A)** The overlay visualization map of keywords analysis conducted by VOSviewer. **(B)** Top 15 keywords with the strongest citation bursts. The light green line segment represents the period before the keyword appears, the dark green line segment denotes the period after the keyword appears, and the red line segment indicates the duration of the citation burst for the keyword. **(C)** Top trend topics over the time for Microglia research in TBI.

Keywords are crucial as they effectively represent the main topics and broader scope of articles and their themes (Agnusdei and Coluccia, 2022). We utilized VOSviewer to analyze keywords that occurred more than twenty times throughout the literature (Figure 8A). In total, 55 were identified. The top five are microglia (497 occurrences), traumatic brain injury (421 occurrences), neuroinflammation (236 occurrences), inflammation (207 occurrences), and activation (137 occurrences). The nodes in the figure, distinguished by color, represent research hotspots from different periods. Blue nodes indicate early research themes, such as closed traumatic brain injury and spinal cord injury, which primarily focus on the injuries themselves. In contrast, the yellow nodes, which include neuroinflammation, activation, neurodegenerative diseases, and polarization, highlight long-term complications and mechanisms following traumatic brain injury. These topics represent recent hotspots and are likely to remain at the forefront of future research.

Figure 8B, analyzed using Citespace, presents the top 20 keywords exhibiting the most significant citation bursts. The earliest highly cited keywords over the past 24 years were “central nervous system” (strength: 3.4) and “spinal cord injury” (strength: 3.61). The citation burst for “spinal cord injury” lasted from 2001 to 2016. The most prominent keyword was “closed head injury” (strength: 9.29), followed by “memory” (strength: 4.94). After 2021, “memory” and “microglial polarization” emerged as two of the most influential keywords in terms of citations.

By analyzing the trends topics (Figure 8C), we observed that popular themes have shifted from previous focuses on closed-head-injury and rat-brain to more recent topics such as microglial polarization, neuroinflammation, and dysfunction. This transition indicates that researchers' understanding of microglia related to traumatic brain injury has evolved from superficial insights to more nuanced perspectives.

## 4 Discussion

This study evaluated the current status and research hotspots of microglia in TBI. We retrieved articles related to TBI and microglia from the Web of Science database covering 2000 to 2023. A total of 665 English-language papers from 834 institutions across 25 countries/regions were included in the study. Subsequently, statistical analysis and data processing were performed using VOSviewer, the R package “bibliometrix”, Excel, and Citespace software.

### 4.1 The general characteristics of the papers

The annual volume and trend of literature can offer insights into the evolution and advancement of the research field (Ma et al., 2021). Figure 2 shows that the number of publications related to microglia in TBI increased only modestly from 2000 to 2010, indicating that research in this area was still in its early stages. However, there was a rapid surge in publications after 2010, particularly in 2019, with the number of articles peaking at 85 in 2023. This suggests that the role of microglia in TBI has garnered increasing scholarly attention in recent years.

In terms of distribution by country/region, the United States leads in both the number of published articles and citation counts. The analysis of publishing institutions reveals that most of the top 10 institutions are located in the U.S. These organizations not only entered the field early but also fostered strong collaborative relationships, solidifying the U.S.'s significant and influential position in this area. Notably, China's publication volume has surged since 2010, closely following the U.S. In recent years, numerous Chinese institutions have emerged in this research domain, introducing fresh perspectives and revitalizing the field. This growth can be attributed to the research policies and funding initiatives implemented by the Chinese government over the past decade (Dong et al., 2019).

According to Table 3, the *Journal of Neurotrauma* and the *Journal of Neuroinflammation* occupy the top two positions in terms of publication volume and citation count. Additionally, *Experimental Neurology* ranks third in both metrics, highlighting the significance of these three journals in this field. Figure 5A reveals that among the three journals, the Journal of Neurotrauma was one of the earliest to address the role of microglia in TBI. At the same time, the *Journal of Neuroinflammation* has increasingly focused on this topic in recent years. Furthermore, this area has a close connection between the three journals.

As demonstrated in Table 4, David J Loane from the University of Pittsburgh School of Medicine is both the most prolific author and the most co-cited author, underscoring his pivotal role in this area of research. Dr. Loane is a distinguished scientist with substantial contributions to neuroscience. His work has extensively examined the impact of neuroinflammation on the progression of neurodegenerative diseases and has provided valuable insights for developing related therapeutic strategies. As shown in Figure 5, three of the top 10 most cited papers are authored by David J. Loane. In 2010, David J Loane et al. published a review titled “Role of Microglia in Neurotrauma” in *Neurotherapeutics*. This comprehensive review delineates the functional roles of microglia in traumatic brain injury (Loane and Byrnes, 2010). In 2014, Loane DJ et al. published an article titled “Progressive Neurodegeneration After Experimental Brain Trauma: Association with Chronic Microglial Activation” in the *Journal of Neuropathology & Experimental Neurology* (Loane et al., 2014). This study identified a potential link between neurodegenerative changes following traumatic brain injury and chronic microglial activation. In 2016, Loane DJ and colleagues published a review titled “Microglia in the TBI Brain: The Good, the Bad, and the Dysregulated” in *Experimental Neurology*. This review focuses on microglia's various phenotypes and functions following traumatic brain injury (Loane and Kumar, 2016).

### 4.2 Research hotspots and keywords

We analyzed literature keywords using R software, VOSviewer, and CiteSpace to identify research hotspots related to TBI and microglia. Our analysis indicates a shift in research focus toward microglial polarization, neurodegenerative diseases, and neuroinflammation. The shift results from an evolving understanding of TBI. Previously viewed as an acute condition, research now shows that TBI can lead to ongoing issues even after the initial recovery phase, characterizing it as a chronic health condition (Wilson et al., 2017). Shi et al. (2019) demonstrated that brain inflammation post-TBI extends beyond the injury site, potentially influencing distant regions and contributing to progressive neurodegeneration (Washington et al., 2016). Numerous studies have linked TBI with loss of consciousness to Alzheimer's disease, while Crane highlighted its association with Parkinson's disease progression (Washington et al., 2016; Crane et al., 2016). Learning and memory deficits remain among the most frequently reported long-term symptoms following TBI (Eyolfson et al., 2024). On the other hand, as the primary immune cells in the brain, researchers have found that microglia remain activated for decades following TBI (Ramlackhansingh et al., 2011). This prolonged activation has made the relationship between TBI and microglial response a focal point of research in recent years.

#### 4.2.1 Changes in microglial states and functions following traumatic brain injury

Microglia constitute approximately 10–15% of all glial cells and are often described as the CNS resident macrophages (Nayak et al., 2014). Unlike meningeal, choroid plexus, and perivascular macrophages, originating from bone marrow, microglia are derived from the yolk sac (Bolte and Lukens, 2021). Their highly dynamic nature, widespread distribution throughout the brain, and phagocytic capabilities render them critical first responders to brain injury (Roth et al., 2014). Upon detecting specific signals resulting from parenchymal injury, degeneration, or infection, microglia undergo morphological transformations and promptly activate genetic programs to address and repair CNS damage (Nayak et al., 2014). Following TBI, microglia transition to another state and address neurotoxic substances by phagocytosing and processing cellular debris and stimuli within the central nervous system (Damisah et al., 2020; Sierra et al., 2010; Márquez-Ropero et al., 2020). On the other hand, one of the most significant changes after TBI is the leakage of substances into the brain parenchyma due to the disruption of cellular barriers. Microglia can transform into jellyfish-like phagocytic cells, replacing deceased astrocytes by integrating into the damaged neuroglial limitans to maintain the integrity of the neuroglial barrier (Corps et al., 2015). This response depends on connexin hemichannels and purinergic receptor signaling. Local inhibition of these microglial responses by blocking purinergic receptor signaling or connexin hemichannels leads to more pronounced pathological mechanisms following brain injury (Corps et al., 2015).

Activated microglia can polarize into various phenotypes in response to different environmental signals, similar to the activation of peripheral macrophages, with transitions to M1 and M2 states. However, microglia exhibit greater heterogeneity, and their activation states are influenced by the interplay between external stimuli and the intracellular environment (Aria and Cuccurullo, 2017). As a result, many studies describe microglial polarization as favoring “M1-like” or “M2-like” states. Research shows that the changes in microglial states following traumatic brain injury (TBI) are time-dependent (Wang et al., 2013). Additionally, changes in microglial states may be reversible, adapting to different phenotypes in response to fluctuating environmental stimuli (Karve et al., 2016). Microglia in one state are primarily associated with inflammatory responses, neural injury, and infection, playing a crucial role in defending against pathogens and responding to neural damage. However, excessive or uncontrolled activation of this state can be detrimental to the nervous system. In contrast, microglia in another state are essential for regeneration and repair in the central nervous system. On one hand, they can enhance neurogenesis in co-culture systems by increasing insulin-like growth factor-1 (IGF-1) (Butovsky et al., 2006). On the other hand, research indicates their critical role in oligodendrocyte differentiation and myelin regeneration following central nervous system injury (Loane and Kumar, 2016). However, these findings have yet to be validated in TBI models, as they have primarily been demonstrated in *in vitro* studies involving multiple sclerosis and hypoxia-ischemia. This gap presents a promising avenue for future research.

#### 4.2.2 Microglia and neuroinflammation following TBI

Neuroinflammation following TBI is classified into acute and chronic phases (Simon et al., 2017). Acute neuroinflammation typically begins within minutes after the injury, as damaged neurons release danger signals, such as damage-associated molecular patterns (DAMPs), triggering a cascade of immune responses. This process recruits peripheral immune cells, including neutrophils, monocytes, and lymphocytes, to the injury site. These cells secrete various inflammatory mediators, promoting complex interactions that drive post-TBI inflammation (Shi et al., 2019). Due to their abundant DAMP sensors, microglia are typically among the first responders to brain injury. Following TBI, microglia, similar to peripheral macrophages, react to changes in the brain's microenvironment by transitioning through multiple states (Colton, 2009). One state is characterized by the production of pro-inflammatory cytokines, chemokines, and reactive oxygen species (ROS). While this state is generally viewed as harmful, moderate microglial stimulation in this state may offer neuroprotection after TBI. In contrast, excessive stimulation can lead to secondary brain injury and the spread of neuroinflammation (Loane and Byrnes, 2010; Wang et al., 2013).

After the acute inflammatory response following TBI, it is generally expected that this response will gradually subside. However, in some patients, chronic neuroinflammation may develop and persist for years after the injury (Simon et al., 2017; Johnson et al., 2013; Morganti-Kossmann et al., 2019). Ramlackhansingh found that changes in microglial states can persist for up to 17 years following TBI, indicating that TBI triggers chronic inflammatory responses, particularly in subcortical regions (Ramlackhansingh et al., 2011). It remains unclear whether persistent inflammation induces characteristic neuropathological changes, underscoring the need for further research to elucidate the chronic pathological mechanisms following TBI and to provide new avenues for patient treatment.

#### 4.2.3 Microglia and neurodegenerative diseases associated with TBI

Growing evidence highlights a strong link between TBI and the development of neurodegenerative diseases, including Alzheimer's disease, Parkinson's disease and dementia (Wilson et al., 2017; Crane et al., 2016; Stocchetti and Zanier, 2016). Research indicates that a long history of TBI can accelerate the onset of cognitive impairments associated with Alzheimer's disease and increase the presence of pathology-related biomarkers linked to the condition (Vaibhav et al., 2024). Collectively, these neurodegenerative conditions are referred to as chronic traumatic encephalopathy, characterized by frontal and temporal lobe atrophy, neuronal and axonal loss, and abnormal protein deposition (Johnson et al., 2013; Ghosh et al., 2013; Maphis et al., 2015). The activation of microglia and the persistence of neuroinflammation following TBI play a crucial role in these pathological manifestations. Consequently, investigating the role of microglia in the aftermath of TBI is essential for advancing therapeutic strategies aimed at mitigating neurodegenerative changes. Current research has reported a significant increase in mitochondrial fission in mice following TBI, which triggers chronic neurodegeneration persisting for 17 months. Notably, blocking excessive mitochondrial fission within 2 weeks post-TBI can prevent chronic neurodegeneration. This finding offers insights for potential treatments of neurodegenerative conditions in humans following TBI.

### 4.3 Limitations

This bibliometric study provides a relatively straightforward analysis of articles related to microglia in the context of TBI. The results are objective and accurate, offering valuable insights for scholars in this field. However, there are some limitations to this study. Firstly, our research only includes publications from the WoSCC, excluding other databases and articles in different languages. As a result, some relevant articles may have been overlooked. Nevertheless, WoSCC is widely acknowledged as one of the most comprehensive sources of scientific literature, and the data retrieved are sufficient to represent the current research landscape. Additionally, earlier publications often receive higher citation rates, which may hinder newer high-quality studies from ranking among the most cited. However, we have conducted a recent hotspot analysis on related topics. Finally, this analysis only includes publications up to 2023; readers interested in the latest developments will need to conduct further searches for up-to-date literature.

## 5 Conclusions

This study offers the first thorough bibliometric analysis of literature on microglia related to TBI from 2000 to 2023. The increasing number of publications and citations each year underscores the growing research interest in this field. The United States, China, Germany, and Australia are leading, with China showing particularly notable recent engagement. The Journal of Neurotrauma and the Journal of Neuroinflammation are identified as the most influential journals, while Alan I. Faden from the University of Maryland School of Medicine and David J. Loane from the University of Pittsburgh School of Medicine are recognized as key contributors. Keyword analysis indicates that neuroinflammation, microglial polarization, and neurodegenerative diseases are current research hotspots, suggesting a need for further investigation into microglial roles in post-TBI neuroinflammation and neurodegeneration, which could improve TBI prognosis.

## Data Availability

The original contributions presented in the study are included in the article/Supplementary material, further inquiries can be directed to the corresponding author.
