# Supplementary material for: Panaroma of microglia in traumatic brain injury: a bibliometric analysis and visualization study during 2000–2023
Source: Front Cell Neurosci. 2024 Nov 7;18:1495542. doi: 10.3389/fncel.2024.1495542 (PMC11578739; doi:10.3389/fncel.2024.1495542)
Supplement: Supplementary file 1 [file Data_Sheet_1.DOCX]

FN Clarivate Analytics Web of Science

VR 1.0

PT J

AU Rao, VLR

Dogan, A

Bowen, KK

Dempsey, RJ

AF Rao, VLR

Dogan, A

Bowen, KK

Dempsey, RJ

TI Traumatic brain injury leads to increased expression of peripheral-type

benzodiazepine receptors, neuronal death, and activation of astrocytes

and microglia in rat thalamus

SO EXPERIMENTAL NEUROLOGY

LA English

DT Article

DE astrocytes; microglia; mRNA; peripheral-type benzodiazepine receptor;

PK11195; quantitative autoradiography; traumatic brain injury

ID TUMOR-NECROSIS-FACTOR; GLOBAL FOREBRAIN ISCHEMIA; CORTICAL IMPACT

INJURY; PROTEIN MESSENGER-RNA; LIGAND H-3 PK11195; BINDING-SITES;

PORTACAVAL ANASTOMOSIS; QUANTITATIVE AUTORADIOGRAPHY; CULTURED

ASTROCYTES; ENHANCED EXPRESSION

AB In mammalian CNS, the peripheral-type benzodiazepine receptor (PTBR) is localized on the outer mitochondrial membrane within the astrocytes and microglia. PTBR transports cholesterol to the site of neurosteroid biosynthesis. Several neurodegenerative disorders were reported to be associated with increased densities of PTBR. In the present study, we evaluated the changes in the PTBR density and gene expression in the brains of rats as a function of time (6 h to 14 days) after traumatic brain injury (TBI). Sham-operated rats served as control. Between 3 and 14 days after TBI, there was a significant increased in the binding of PTBR antagonist [H-3]PK11195 (by 106 to 185%, P < 0.01, as assessed by quantitative autoradiography and in vitro filtration binding) and PTBR mRNA expression (by 2- to 3.4-fold, P < 0.01, as assessed by RT-PCR) in the ipsilateral thalamus. At 14 days after the injury, the neuronal number decreased significantly (by 85 to 90%, P < 0.01) in the ipsilateral thalamus. At the same time point, the ipsilateral thalamus also showed increased numbers of the glial fibrillary acidic protein positive cells (astrocytes, by similar to 3.5-fold) and the ED-1 positive cells (microglia/macrophages, by similar to 36-fold), the two cell types known to be associated with PTBR, Increased PTBR expression following TBI seems to be associated with microglia/macrophages than astrocytes as PTBR density at different periods after TBI correlated better with the number of ED-1 positive cells (r(2) = 0.95) than the GFAP positive cells (r(2) = 0.56). TBI-induced increased PTBR expression is possibly an adaptive response to cellular injury and may play a role in the pathophysiology of TBI. (C) 2000 Academic Press.

C1 Univ Wisconsin, Dept Neurol Surg, Madison, WI 53706 USA.

William S Middleton Mem Vet Adm Med Ctr, Madison, WI USA.

C3 University of Wisconsin System; University of Wisconsin Madison; US

Department of Veterans Affairs; Veterans Health Administration (VHA);

William S Middleton Memorial Veterans Hospital

RP Rao, VLR (corresponding author), Univ Wisconsin, Dept Neurol Surg, Madison, WI 53706 USA.

RI Dogan, Aclan/AAF-8305-2019

CR AIHARA N, 1995, J NEUROTRAUM, V12, P53, DOI 10.1089/neu.1995.12.53

Baskaya MK, 1997, NEUROSCI LETT, V226, P33

BLACK KL, 1990, CANCER, V65, P93, DOI 10.1002/1097-0142(19900101)65:1<93::AID-CNCR2820650120>3.0.CO;2-1

BOURDIOL F, 1991, BRAIN RES, V543, P194, DOI 10.1016/0006-8993(91)90028-T

Clark RSB, 1997, J NEUROSCI, V17, P9172

Colicos MA, 1996, BRAIN RES, V739, P111, DOI 10.1016/S0006-8993(96)00819-0

Conway EL, 1998, NEUROSCIENCE, V82, P805

CORNU P, 1992, ACTA NEUROCHIR, V119, P146, DOI 10.1007/BF01541799

Désarnaud F, 1998, J NEUROCHEM, V71, P1765

Desjardins P, 1997, BRAIN RES, V758, P255, DOI 10.1016/S0006-8993(97)00339-9

DESJARDINS P, 1999, IN PRESS NEUROCHEM I

Dixon CE, 1998, J NEUROTRAUM, V15, P95, DOI 10.1089/neu.1998.15.95

DUCIS I, 1990, BRAIN RES, V531, P318, DOI 10.1016/0006-8993(90)90793-B

DunnMeynell AA, 1997, BRAIN RES, V761, P25, DOI 10.1016/S0006-8993(97)00210-2

Frye CA, 1998, PSYCHONEUROENDOCRINO, V23, P385, DOI 10.1016/S0306-4530(98)00009-2

GarciaSegura LM, 1996, CELL MOL NEUROBIOL, V16, P225, DOI 10.1007/BF02088178

Gavish M, 1997, CLIN NEUROPHARMACOL, V20, P473, DOI 10.1097/00002826-199712000-00001

Gehlert DR, 1997, NEUROCHEM INT, V31, P705, DOI 10.1016/S0197-0186(97)00007-7

Gourin CG, 1997, J TRAUMA, V42, P1101, DOI 10.1097/00005373-199706000-00020

GROOM GN, 1995, J NUCL MED, V36, P2207

GUILARTE TR, 1995, NEUROTOXICOLOGY, V16, P441

GUTH L, 1994, P NATL ACAD SCI USA, V91, P12308, DOI 10.1073/pnas.91.25.12308

Hirsch T, 1998, EXP CELL RES, V241, P426, DOI 10.1006/excr.1998.4084

HOLMIN S, 1995, ACTA NEUROCHIR, V132, P110, DOI 10.1007/BF01404857

ITZHAK Y, 1993, GLIA, V9, P211, DOI 10.1002/glia.440090306

Jiang N, 1996, BRAIN RES, V735, P101, DOI 10.1016/0006-8993(96)00605-1

JosephLiauzun E, 1997, J BIOL CHEM, V272, P28102, DOI 10.1074/jbc.272.44.28102

Kaya SS, 1999, BRAIN RES, V818, P23, DOI 10.1016/S0006-8993(98)01204-9

Krueger KE, 1995, BBA-REV BIOMEMBRANES, V1241, P453, DOI 10.1016/0304-4157(95)00016-X

Lacor P, 1996, NEUROSCI LETT, V220, P61, DOI 10.1016/S0304-3940(96)13187-6

LAVOIE J, 1990, HEPATOLOGY, V11, P874, DOI 10.1002/hep.1840110524

LEONG DK, 1994, J CEREBR BLOOD F MET, V14, P100, DOI 10.1038/jcbfm.1994.14

Leong DK, 1996, ALCOHOL CLIN EXP RES, V20, P601, DOI 10.1111/j.1530-0277.1996.tb01101.x

LOWRY OH, 1951, J BIOL CHEM, V193, P265

Matthews MA, 1998, BRAIN RES, V794, P1, DOI 10.1016/S0006-8993(98)00107-3

McGeer E G, 1988, Alzheimer Dis Assoc Disord, V2, P331, DOI 10.1097/00002093-198802040-00001

MCPHERSON GA, 1994, RADLIG MANUAL VERSIO

Meziane H, 1996, PSYCHOPHARMACOLOGY, V126, P323, DOI 10.1007/BF02247383

MOHLER H, 1977, SCIENCE, V198, P849, DOI 10.1126/science.918669

Panizzon KL, 1998, NEUROREPORT, V9, P4131, DOI 10.1097/00001756-199812210-00024

Papadopoulos V, 1998, P SOC EXP BIOL MED, V217, P130

Paxinos G, 1998, RAT BRAIN IN STEREOTAXIC COORDINATES, FOURTH ED., pix

Pierce JES, 1998, NEUROSCIENCE, V87, P359, DOI 10.1016/S0306-4522(98)00142-0

RAMSAY SC, 1992, LANCET, V339, P1054, DOI 10.1016/0140-6736(92)90576-O

Rao V. L. Raghavendra, 1993, Pediatric Research, V34, P777

RAO VLR, 1994, DIGEST DIS SCI, V39, P1055

Rao VLR, 1997, BRAIN RES, V765, P169, DOI 10.1016/S0006-8993(97)00652-5

Rao VLR, 1997, EUR J PHARMACOL, V340, P89, DOI 10.1016/S0014-2999(97)01395-2

Rao VLR, 1998, J NEUROCHEM, V70, P2020

Schumacher M, 1996, DEV NEUROSCI-BASEL, V18, P6, DOI 10.1159/000111391

SempleRowland SL, 1995, J NEUROTRAUM, V12, P1003, DOI 10.1089/neu.1995.12.1003

SPRENGEL R, 1989, J BIOL CHEM, V264, P20415

STEPHENSON DT, 1995, J NEUROSCI, V15, P5263

Todd KG, 1999, GLIA, V25, P190, DOI 10.1002/(SICI)1098-1136(19990115)25:2<190::AID-GLIA9>3.0.CO;2-B

Urani A, 1998, BRAIN RES, V799, P64, DOI 10.1016/S0006-8993(98)00469-7

Verma A, 1998, MOL MED, V4, P40, DOI 10.1007/BF03401728

Vowinckel E, 1997, J NEUROSCI RES, V50, P345, DOI 10.1002/(SICI)1097-4547(19971015)50:2<345::AID-JNR22>3.0.CO;2-5

Waters SL, 1997, AM J PHYSIOL-RENAL, V273, pF869, DOI 10.1152/ajprenal.1997.273.6.F869

Whalen MJ, 1997, J NEUROTRAUM, V14, P561, DOI 10.1089/neu.1997.14.561

ZAVALA F, 1990, J PHARMACOL EXP THER, V255, P442

Zhu WJ, 1997, J NEUROSCI, V17, P4022

Zisterer DM, 1997, GEN PHARMACOL-VASC S, V29, P305, DOI 10.1016/S0306-3623(96)00473-9

NR 62

TC 127

Z9 143

U1 0

U2 10

PU ACADEMIC PRESS INC ELSEVIER SCIENCE

PI SAN DIEGO

PA 525 B ST, STE 1900, SAN DIEGO, CA 92101-4495 USA

SN 0014-4886

EI 1090-2430

J9 EXP NEUROL

JI Exp. Neurol.

PD JAN

PY 2000

VL 161

IS 1

BP 102

EP 114

DI 10.1006/exnr.1999.7269

PG 13

WC Neurosciences

WE Science Citation Index Expanded (SCI-EXPANDED)

SC Neurosciences & Neurology

GA 290FU

UT WOS:000085666300009

PM 10683277

DA 2024-03-03

ER

PT J

AU Dietrich, A

Fülöp, ZL

Chambers, MD

Darrell, RS

Stein, DG

AF Dietrich, A

Fülöp, ZL

Chambers, MD

Darrell, RS

Stein, DG

TI The effect of Ginkgo biloba extract (EGb 761) on gliotic reactions in

the hippocampal formation after unilateral entorhinal cortex lesions

SO RESTORATIVE NEUROLOGY AND NEUROSCIENCE

LA English

DT Article

DE Ginkgo biloba; microglia; astrocyte; neuronal death; entorhinal cortex;

traumatic brain injury; stereology

ID CENTRAL-NERVOUS-SYSTEM; MESSENGER-RNA; CULTURED ASTROCYTES; GERBIL

HIPPOCAMPUS; AMEBOID MICROGLIA; COUNTING METHODS; RAT HIPPOCAMPUS;

NEURONS; RECOVERY; CELLS

AB Purpose: Ginkgo biloba extract (EGb 761) has been shown to facilitate behavioral and neuro-morphological recovery from brain injury, but less is known about its effects on glia. Since gliosis may be an important component of the recovery process, we tested the hypothesis that EGb 761 alters the time course and development of microglial activation and astrocytosis after brain injury.

Methods. Rats were treated with either saline or EGb 761 and killed at 2 hrs, 1, 3, 7, and 14 days following unilateral entorhinal cortex (EC) lesions. Microglia and their precursors were visualized with a silver impregnation method. and astrocytes with GFAP.

Results: Blood-borne monocytes/macrophages were seen as early as 2 hrs after injury in all animals. The side contralateral to the injury showed minimal microglial activation and there were no significant effects of drug treatment. On the side ipsilateral to the lesion EGb 761 enhanced microglial activation at 3, 7, and 14 days in the molecular layer and the hilus of the dentate gyrus; the areas of most profound deafferentation after EC injury. Regions of the corpus callosum also showed enhanced microglial activation over the same time course. Reactive astrocytes were stained with GFAP and were found to be more numerous than activated microglia, particularly in the ipsilateral corpus callosum. EGb 761 treatment enhanced astrocytosis at 3 days in the molecular layer, the hilus, and the corpus callosum on the ipsilateral side.

Conclusions. Taken together our results show that EGb 761 enhances, accelerates and prolongs the activation of microglia and astrocytosis at the site of injury.

C1 Emory Univ, Grad Sch, Sch Med, Dept Neurol,Brain Res Lab, Atlanta, GA 30322 USA.

C3 Emory University

RP Stein, DG (corresponding author), Emory Univ, Grad Sch, Sch Med, Dept Neurol,Brain Res Lab, 202 Adm Bldg,575 Rollins Way, Atlanta, GA 30322 USA.

EM dgstein@grad.gsas.emery.edu

RI Stein, Donald/AAJ-5139-2020

CR ARAKI T, 1994, METAB BRAIN DIS, V9, P369, DOI 10.1007/BF02098883

Beltramino C A, 1993, NIDA Res Monogr, V136, P101

BORZEIX MG, 1985, EFFECTS GINKGO BILOB

Brailowsky S, 1997, NEUROBIOL AGING, V18, P219, DOI 10.1016/S0197-4580(97)00007-9

CADMAN ED, 1994, J NEUROCHEM, V63, P980

COGGESHALL RE, 1992, TRENDS NEUROSCI, V15, P9, DOI 10.1016/0166-2236(92)90339-A

DeFeudis F.V., 1991, GINKGO BILOBA EXTRAC

Deller T, 1997, PROG NEUROBIOL, V53, P687, DOI 10.1016/S0301-0082(97)00044-0

Foster JA, 1997, MOL BRAIN RES, V45, P207, DOI 10.1016/S0169-328X(96)00138-6

Fulop ZL, 1997, INT J NEUROSCI, V90, P203, DOI 10.3109/00207459709000639

GAGE FH, 1988, EXP NEUROL, V102, P2, DOI 10.1016/0014-4886(88)90073-8

GALLYAS F, 1993, J NEUROSCI METH, V50, P159, DOI 10.1016/0165-0270(93)90004-B

Gallyas Ferenc, 1994, Neurobiology (Budapest), V2, P245

GIULIAN D, 1990, EOS-RIV IMMUNOL, V10, P15

GIULIAN D, 1985, J CELL BIOL, V101, P2411, DOI 10.1083/jcb.101.6.2411

GIULIAN D, 1987, J NEUROSCI RES, V18, P155, DOI 10.1002/jnr.490180123

GUNDERSEN HJG, 1986, J MICROSC-OXFORD, V143, P3, DOI 10.1111/j.1365-2818.1986.tb02764.x

Hoffman SW, 1997, RESTOR NEUROL NEUROS, V11, P1, DOI 10.3233/RNN-1997-111201

Hu JG, 1998, BRAIN RES, V785, P195, DOI 10.1016/S0006-8993(97)01318-8

HUGUET F, 1992, J PHARM PHARMACOL, V44, P24, DOI 10.1111/j.2042-7158.1992.tb14357.x

INAGAKI N, 1991, NEUROSCI LETT, V128, P257, DOI 10.1016/0304-3940(91)90274-W

Jones LL, 1997, J NEUROCYTOL, V26, P755, DOI 10.1023/A:1018514415073

JUCKER M, 1995, MOL BRAIN RES, V28, P149, DOI 10.1016/0169-328X(94)00206-T

KETTENMANN H, 1984, NEUROSCI LETT, V52, P25, DOI 10.1016/0304-3940(84)90345-8

Klein MA, 1997, GLIA, V19, P227, DOI 10.1002/(SICI)1098-1136(199703)19:3<227::AID-GLIA5>3.0.CO;2-W

KREUTZBERG GW, 1995, ARZNEIMITTEL-FORSCH, V45-1, P357

Lamproglou I, 1997, ADV GINKGO, V6, P73

LOESCHE J, 1977, BRAIN RES BULL, V2, P31, DOI 10.1016/0361-9230(77)90022-3

MANTHORPE M, 1986, ASTROCYTES, P315

MESHUL CK, 1987, BRAIN RES, V402, P139, DOI 10.1016/0006-8993(87)91056-0

MICHEL RP, 1988, J MICROSC-OXFORD, V150, P117, DOI 10.1111/j.1365-2818.1988.tb04603.x

OTANI M, 1986, ACTA NEUROPATHOL, V69, P54, DOI 10.1007/BF00687039

PATEL AJ, 1990, NEUROL NEUR, V55, P123

Paxinos G., 1986, RAT BRAIN STEREOTAXI, Vsecond

Payne AN, 1996, INFLAMM RES, V45, P575, DOI 10.1007/BF02312037

RAMASSAMY C, 1993, ADV GINKGO, V2, P39

Ramirez JJ, 1996, P NATL ACAD SCI USA, V93, P15512, DOI 10.1073/pnas.93.26.15512

REEVES TM, 1987, BEHAV NEUROSCI, V101, P179

Saper CB, 1996, J COMP NEUROL, V364, P5

Shifman MI, 1996, ADV GINKGO, V5, P61

SIEVERS J, 1994, ANN ANAT, V176, P45, DOI 10.1016/S0940-9602(11)80414-0

SONNENFELD MJ, 1995, J COMP NEUROL, V359, P644, DOI 10.1002/cne.903590410

STERIO DC, 1984, J MICROSC-OXFORD, V134, P127, DOI 10.1111/j.1365-2818.1984.tb02501.x

STEWARD O, 1992, HIPPOCAMPUS, V2, P247, DOI 10.1002/hipo.450020305

SVENSSON M, 1993, J NEUROSCI RES, V35, P373, DOI 10.1002/jnr.490350404

TROOST D, 1993, NEUROPATH APPL NEURO, V19, P390, DOI 10.1111/j.1365-2990.1993.tb00459.x

VANDENPOL AN, 1990, J COMP NEUROL, V296, P654, DOI 10.1002/cne.902960410

WEST MJ, 1991, ANAT RECORD, V231, P482, DOI 10.1002/ar.1092310411

Zalewska T, 1996, ACTA NEUROBIOL EXP, V56, P41, DOI 10.55782/ane-1996-1102

NR 49

TC 1

Z9 1

U1 0

U2 0

PU IOS PRESS

PI AMSTERDAM

PA NIEUWE HEMWEG 6B, 1013 BG AMSTERDAM, NETHERLANDS

SN 0922-6028

J9 RESTOR NEUROL NEUROS

JI Restor. Neurol. Neurosci.

PY 2000

VL 16

IS 2

BP 87

EP 96

PG 10

WC Neurosciences

WE Science Citation Index Expanded (SCI-EXPANDED)

SC Neurosciences & Neurology

GA 299CB

UT WOS:000086177300002

PM 12671211

DA 2024-03-03

ER

PT J

AU Koshinaga, M

Katayama, Y

Fukushima, M

Oshima, H

Suma, T

Takahata, T

AF Koshinaga, M

Katayama, Y

Fukushima, M

Oshima, H

Suma, T

Takahata, T

TI Rapid and widespread microglial activation induced by traumatic brain

injury in rat brain slices

SO JOURNAL OF NEUROTRAUMA

LA English

DT Article

DE brain slice; C3b complement receptor (CR3); controlled impact injury;

microglial activation; OX42

ID COLONY-STIMULATING FACTORS; NITRIC-OXIDE; EXTRACELLULAR POTASSIUM;

MONONUCLEAR PHAGOCYTES; SPREADING DEPRESSION; MACROPHAGES; INVITRO;

ADULT; CELLS; EXPRESSION

AB In order to assess the role of circulating blood in early microglial activation after traumatic brain injury (TBI), controlled cortical impact injury was applied to adult rat brain slices (400 mu m in thickness) and the microglial response was examined. The complement receptor (CR3) expression and morphological transformation of the microglia were evaluated by OX42 immunohistochemistry. At 5 min following injury, activated microglia with intense CR3 expression appeared throughout the hemisphere on the injured side. In contrast, the morphology and CR3 expression of the microglia on the contralateral side were indistinguishable from those of the resident ramified microglia seen in normal brains, At 30 min following injury, microglial activation was more pronounced on the injured side, while the microglia on the contralateral side still retained a ramified morphology, These results are consistent with our previous observations made in in vivo experiments, which indicate that, as the brain slice paradigm excludes variables arising from the circulating blood, the rapid and widespread microglial activation observed following TBI can not be attributed exclusively to the infiltration of blood-borne macrophages or molecules. Rather this activation is most likely caused by intrinsic mechanisms within the brain tissue, such as traumatic depolarization.

C1 Nihon Univ, Sch Med, Dept Neurol Surg, Itabashi Ku, Tokyo 1738610, Japan.

C3 Nihon University

RP Koshinaga, M (corresponding author), Nihon Univ, Sch Med, Dept Neurol Surg, Itabashi Ku, 30-1 Oyaguchi Kamimachi, Tokyo 1738610, Japan.

CR AKIYAMA H, 1988, J NEUROSCI RES, V20, P147, DOI 10.1002/jnr.490200202

BANATI RB, 1994, J CEREBR BLOOD F MET, V14, P145, DOI 10.1038/jcbfm.1994.19

BANATI RB, 1993, GLIA, V7, P111, DOI 10.1002/glia.440070117

BOJE KM, 1992, BRAIN RES, V587, P250, DOI 10.1016/0006-8993(92)91004-X

Caggiano AO, 1996, J COMP NEUROL, V369, P93, DOI 10.1002/(SICI)1096-9861(19960520)369:1<93::AID-CNE7>3.0.CO;2-F

CHAO CC, 1992, J IMMUNOL, V149, P2736

COLTON CA, 1993, J NEUROSCI RES, V35, P297, DOI 10.1002/jnr.490350309

Del Rio-Hortega R., 1965, CYTOLOGY CELLULAR PA, P483

DIXON CE, 1991, J NEUROSCI METH, V39, P253

Elkabes S, 1996, J NEUROSCI, V16, P2508

FINSEN BR, 1993, J CHEM NEUROANAT, V6, P267, DOI 10.1016/0891-0618(93)90048-9

GEHRMANN J, 1993, BRAIN PATHOL, V3, P11, DOI 10.1111/j.1750-3639.1993.tb00720.x

GIULIAN D, 1989, J NEUROSCI, V9, P4416

GIULIAN D, 1993, J NEUROSCI RES, V36, P681, DOI 10.1002/jnr.490360609

GIULIAN D, 1990, ANN NEUROL, V27, P33, DOI 10.1002/ana.410270107

GIULIAN D, 1988, J NEUROSCI, V8, P4707

GRAEBER MB, 1988, J NEUROSCI RES, V21, P18, DOI 10.1002/jnr.490210104

HADDAD GG, 1989, J NEUROPHYSIOL, V62, P1213, DOI 10.1152/jn.1989.62.6.1213

Jensen MB, 1997, EXP NEUROL, V143, P103, DOI 10.1006/exnr.1996.6337

KATAYAMA Y, 1995, BRAIN PATHOL, V5, P427, DOI 10.1111/j.1750-3639.1995.tb00621.x

KATAYAMA Y, 1990, J NEUROSURG, V73, P889, DOI 10.3171/jns.1990.73.6.0889

KETTENMANN H, 1990, J NEUROSCI RES, V26, P278, DOI 10.1002/jnr.490260303

KOSHINAGA M, 1995, J NEUROTRAUM, V12, P209, DOI 10.1089/neu.1995.12.209

KREUTZBERG GW, 1989, METAB BRAIN DIS, V4, P81, DOI 10.1007/BF00999498

Kreutzberg GW, 1996, TRENDS NEUROSCI, V19, P312, DOI 10.1016/0166-2236(96)10049-7

LIGHTHALL JW, J NEUROTRAUMA, V5, P1

MORIOKA T, 1991, J CEREBR BLOOD F MET, V11, P966, DOI 10.1038/jcbfm.1991.162

NILSSON P, 1993, J CEREBR BLOOD F MET, V13, P183, DOI 10.1038/jcbfm.1993.22

PIANI D, 1991, NEUROSCI LETT, V133, P159, DOI 10.1016/0304-3940(91)90559-C

SHIMOHAMA S, 1989, MOL BRAIN RES, V5, P271, DOI 10.1016/0169-328X(89)90061-2

SHIPLEY MT, 1994, SOC NEUR ABSTR, V365, P4

STREIT WJ, 1988, GLIA, V1, P301, DOI 10.1002/glia.440010502

SUZUMURA A, 1990, J NEUROIMMUNOL, V30, P111, DOI 10.1016/0165-5728(90)90094-4

SUZUMURA A, 1994, NEUROIMMUNOLOGY TOKY, V1, P122

TAKAHASHI H, 1981, J NEUROSURG, V55, P708, DOI 10.3171/jns.1981.55.5.0708

NR 35

TC 69

Z9 74

U1 0

U2 8

PU MARY ANN LIEBERT INC PUBL

PI LARCHMONT

PA 2 MADISON AVENUE, LARCHMONT, NY 10538 USA

SN 0897-7151

J9 J NEUROTRAUM

JI J. Neurotrauma

PD MAR

PY 2000

VL 17

IS 3

BP 185

EP 192

DI 10.1089/neu.2000.17.185

PG 8

WC Critical Care Medicine; Clinical Neurology; Neurosciences

WE Science Citation Index Expanded (SCI-EXPANDED)

SC General & Internal Medicine; Neurosciences & Neurology

GA 297QY

UT WOS:000086095200002

PM 10757324

DA 2024-03-03

ER

PT J

AU Csuka, E

Hans, VHJ

Ammann, E

Trentz, O

Kossmann, T

Morganti-Kossmann, MC

AF Csuka, E

Hans, VHJ

Ammann, E

Trentz, O

Kossmann, T

Morganti-Kossmann, MC

TI Cell activation and inflammatory response following traumatic axonal

injury in the rat

SO NEUROREPORT

LA English

DT Article

DE astrocytes; experimental; immunohistochemistry; impact-acceleration;

inflammation; MHC class II; microglia; rat; traumatic brain injury

ID DIFFUSE BRAIN INJURY; CEREBROSPINAL-FLUID; MESSENGER-RNA; HEAD-INJURY;

ASTROCYTES; MODEL; EXPRESSION

AB In a rat model of traumatic brain injury cell activation was characterized immunohistochemically from 2 h up to 2 weeks. Reactive astrocytosis became apparent perivascularly and in the grey matter within 4 h after trauma. Increased OX42 immunoreactivity indicated microglial activation in cortex and hippocampus as early as 4 h, whereas up-regulation of MHC class II (OX6) was evident in white matter tracts at 24 h. Although macrophage (EDI) numbers increased in the meninges and perivascularly, brain infiltration appeared marginal. Accumulation of lymphocytes and granulocytes was not observed. Our results show that traumatic axonal injury induces a rapid and sustained glial activation in the absence of leukocyte infiltration. Thus, cell activation following diffuse trauma strongly differs from that found after focal brain damage, awaiting further functional characterization. NeuroReport 11:2587-2590 (C) 2000 Lippincott Williams & Wilkins.

C1 Univ Spital Zurich, Dept Chirurg, Forsch Abt, CH-8091 Zurich, Switzerland.

Univ Spital Zurich, Unfallchirurg Klin, CH-8091 Zurich, Switzerland.

Univ Kliniken Bonn, Inst Neuropathol, D-53105 Bonn, Germany.

C3 University of Zurich; University Zurich Hospital; University of Zurich;

University Zurich Hospital; University of Bonn

RP Morganti-Kossmann, MC (corresponding author), Univ Spital Zurich, Dept Chirurg, Forsch Abt, Ramistr 100, CH-8091 Zurich, Switzerland.

OI Morganti-Kossmann, Cristina/0000-0002-0807-2063

CR ADAMS JH, 1989, HISTOPATHOLOGY, V15, P49, DOI 10.1111/j.1365-2559.1989.tb03040.x

[Anonymous], RAT BRAIN STEREOTAXI

CLARK RSB, 1994, J NEUROTRAUM, V11, P499, DOI 10.1089/neu.1994.11.499

EDDLESTON M, 1993, NEUROSCIENCE, V54, P15, DOI 10.1016/0306-4522(93)90380-X

Engel S, 1996, Acta Neurochir Suppl, V66, P89

Engelborghs K, 1998, J NEUROSURG, V89, P796, DOI 10.3171/jns.1998.89.5.0796

FODA MAA, 1994, J NEUROSURG, V80, P301, DOI 10.3171/jns.1994.80.2.0301

GRAEBER MB, 1988, J NEUROSCI RES, V21, P18, DOI 10.1002/jnr.490210104

Hans VHJ, 1999, J CEREBR BLOOD F MET, V19, P184, DOI 10.1097/00004647-199902000-00010

Hausmann R, 1999, INT J LEGAL MED, V112, P227, DOI 10.1007/s004140050241

Hill SJ, 1996, J NEUROPATH EXP NEUR, V55, P1221, DOI 10.1097/00005072-199612000-00005

Holmin S, 1999, NEUROREPORT, V10, P1889, DOI 10.1097/00001756-199906230-00017

Kossmann T, 1996, BRAIN RES, V713, P143, DOI 10.1016/0006-8993(95)01501-9

MARMAROU A, 1994, J NEUROSURG, V80, P291, DOI 10.3171/jns.1994.80.2.0291

MorgantiKossman MC, 1997, MOL PSYCHIATR, V2, P133, DOI 10.1038/sj.mp.4000227

MORGANTIKOSSMAN.MC, 1995, IMMUNE RESPONSES NER, P159

Neumann H, 1996, EUR J NEUROSCI, V8, P2582, DOI 10.1111/j.1460-9568.1996.tb01552.x

SEKIYA S, 1989, J LEUKOCYTE BIOL, V46, P96, DOI 10.1002/jlb.46.2.96

Soares HD, 1995, J NEUROSCI, V15, P8223

Stahel PF, 1997, MOL BRAIN RES, V50, P205, DOI 10.1016/S0169-328X(97)00189-7

NR 20

TC 86

Z9 97

U1 0

U2 2

PU LIPPINCOTT WILLIAMS & WILKINS

PI PHILADELPHIA

PA 530 WALNUT ST, PHILADELPHIA, PA 19106-3621 USA

SN 0959-4965

J9 NEUROREPORT

JI Neuroreport

PD AUG 3

PY 2000

VL 11

IS 11

BP 2587

EP 2590

DI 10.1097/00001756-200008030-00047

PG 4

WC Neurosciences

WE Science Citation Index Expanded (SCI-EXPANDED)

SC Neurosciences & Neurology

GA 340ZY

UT WOS:000088567600049

PM 10943727

DA 2024-03-03

ER

PT J

AU Engel, S

Schluesener, H

Mittelbronn, M

Seid, K

Adjodah, D

Wehner, HD

Meyermann, R

AF Engel, S

Schluesener, H

Mittelbronn, M

Seid, K

Adjodah, D

Wehner, HD

Meyermann, R

TI Dynamics of microglial activation after human traumatic brain injury are

revealed by delayed expression of macrophage-related proteins MRP8 and

MRP14

SO ACTA NEUROPATHOLOGICA

LA English

DT Article

DE intracellular signalling; Ki-67; microglia activation; normal brain

ischemia

ID CALCIUM-BINDING PROTEINS; LYMPHOCYTE CHEMOATTRACTANT FACTOR;

CENTRAL-NERVOUS-SYSTEM; MULTIPLE-SCLEROSIS LESIONS; CLASS-II ANTIGENS;

CD8(+) T-CELLS; HEAD-INJURY; RAT-BRAIN; CNS; ASTROCYTES

AB Human traumatic brain injury (TBI) is ideally suited for investigation of the kinetics of human microglial cell activation as the onset of lesion formation is precisely defined. The present study provides evidence of a distinct delay in macrophage/microglia response following TBI. Eighteen brains of patients who had survived TBI for 1 h to 6 months were analysed by immunohistology. Samples of contusional and non-contusional areas were studied using antibodies directed against antigens of microglia/macrophages [major histocompatibility complex class II, CD4, interleukin (IL)-16, macrophage-related protein (MRP) 8 and MRP14]. IL-16, a natural ligand to CD4, was expressed constitutively by numerous microglial cells in all cases throughout the brain. CD4 could be detected regularly on perivascular cells. MRP8 and MRP14, which are only expressed on activated macrophages and microglial cells, could be detected only within brains with a survival time of more than 72 h post TBI. In addition, proliferation of microglia detected by MIB-1 was not present until 72 h. This delayed expression of the activation markers MRP8 and MRP14 and the proliferation marker MIB-1 is comparable to experimental closed head injuries but strictly different from acute activation found in ischemic brains.

C1 Univ Tubingen, Inst Brain Res, D-72076 Tubingen, Germany.

Univ Tubingen, Inst Legal Med, D-72076 Tubingen, Germany.

C3 Eberhard Karls University of Tubingen; Eberhard Karls University of

Tubingen

RP Engel, S (corresponding author), Univ Tubingen, Inst Brain Res, Calwer Str 3, D-72076 Tubingen, Germany.

OI Mittelbronn, Michel/0000-0002-2998-052X

CR AIHARA N, 1995, J NEUROTRAUM, V12, P53, DOI 10.1089/neu.1995.12.53

AKIYAMA H, 1994, J NEUROIMMUNOL, V50, P195, DOI 10.1016/0165-5728(94)90046-9

BANATI RB, 1994, DEV NEUROSCI-BASEL, V16, P114, DOI 10.1159/000112098

Bernaudin M, 1998, EXP NEUROL, V150, P30, DOI 10.1006/exnr.1997.6728

BO L, 1994, J NEUROIMMUNOL, V51, P135, DOI 10.1016/0165-5728(94)90075-2

Bobryshev YV, 1996, PATHOL RES PRACT, V192, P260, DOI 10.1016/S0344-0338(96)80229-9

BRUCK W, 1995, ANN NEUROL, V38, P788, DOI 10.1002/ana.410380514

CENTER DM, 1995, J LAB CLIN MED, V125, P167

CENTER DM, 1982, J IMMUNOL, V128, P2562

Cruikshank W W, 1998, Int Rev Immunol, V16, P523, DOI 10.3109/08830189809043007

De la Riva I, 1990, REV ESP HERPETOL, V4, P81

Deininger MH, 1998, HISTOCHEM CELL BIOL, V110, P425, DOI 10.1007/s004180050303

Engel S, 1996, ACT NEUR S, V66, P87

FABRY Z, 1994, IMMUNOL TODAY, V15, P218, DOI 10.1016/0167-5699(94)90247-X

FINSEN BR, 1993, GLIA, V7, P41, DOI 10.1002/glia.440070109

FLARIS NA, 1993, GLIA, V7, P34, DOI 10.1002/glia.440070108

GIULIAN D, 1994, NEUROCHEM INT, V25, P227, DOI 10.1016/0197-0186(94)90066-3

GOEBELER M, 1995, BIOCHEM J, V309, P419, DOI 10.1042/bj3090419

GRAEBER MB, 1988, J NEUROSCI RES, V21, P18, DOI 10.1002/jnr.490210104

Graham DI, 1997, GREENFIELDS NEUROPAT, P197

Heizmann CW, 1996, MOL CELLS, V6, P629

HICKEY WF, 1992, J NEUROPATH EXP NEUR, V51, P246, DOI 10.1097/00005072-199205000-00002

HILT DC, 1988, TRENDS BIOCHEM SCI, V13, P437

Holmin S, 1998, NEUROSURGERY, V42, P291, DOI 10.1097/00006123-199802000-00047

Holmin S, 1997, J NEUROSURG, V86, P493, DOI 10.3171/jns.1997.86.3.0493

Ikura M, 1996, TRENDS BIOCHEM SCI, V21, P14, DOI 10.1016/0968-0004(96)80879-6

KETTENMANN H, 1990, J NEUROSCI RES, V26, P278, DOI 10.1002/jnr.490260303

KLIGMAN D, 1988, TRENDS BIOCHEM SCI, V13, P437, DOI 10.1016/0968-0004(88)90218-6

Krautwald S, 1998, J IMMUNOL, V160, P5874

Kreutzberg GW, 1996, TRENDS NEUROSCI, V19, P312, DOI 10.1016/0166-2236(96)10049-7

Kuruto-Niwa R, 1998, CELL STRUCT FUNCT, V23, P109, DOI 10.1247/csf.23.109

Laberge S, 1996, J IMMUNOL, V156, P310

LABERGE S, 1995, J IMMUNOL, V155, P2902

Lassmann H., 1997, Journal of Neural Transmission Supplement, V50, P183

MATIAZEK JW, 1997, J IMMUNOL, V158, P5

McKeever PE, 1998, J HISTOCHEM CYTOCHEM, V46, P585, DOI 10.1177/002215549804600504

Moller T, 1997, J NEUROSCI, V17, P615

PERRY VH, 1987, J EXP MED, V166, P1138, DOI 10.1084/jem.166.4.1138

Postler E, 1997, GLIA, V19, P27, DOI 10.1002/(SICI)1098-1136(199701)19:1<27::AID-GLIA3>3.0.CO;2-7

POVLISHOCK JT, 1995, J NEUROTRAUM, V12, P555, DOI 10.1089/neu.1995.12.555

QI JF, 1995, J IMMUNOL, V155, P867

RAINE CS, 1994, J NEUROPATH EXP NEUR, V53, P328, DOI 10.1097/00005072-199407000-00002

RENAUD W, 1994, BIOCHEM BIOPH RES CO, V201, P1518, DOI 10.1006/bbrc.1994.1876

Rostworowski M, 1997, J NEUROSCI, V17, P3664

ROTH J, 1994, BIOCHEM J, V301, P655, DOI 10.1042/bj3010655

SASAKI A, 1992, NEUROPATH APPL NEURO, V18, P13, DOI 10.1111/j.1365-2990.1992.tb00761.x

Schafer BW, 1996, TRENDS BIOCHEM SCI, V21, P134, DOI 10.1016/S0968-0004(96)80167-8

Schluesener HJ, 1996, J NEUROSCI RES, V44, P606

SCHLUESENER HJ, 1993, ACTA NEUROPATHOL, V86, P393, DOI 10.1007/BF00369453

SCHMID KW, 1995, HUM PATHOL, V26, P334, DOI 10.1016/0046-8177(95)90067-5

SIEVERS J, 1994, GLIA, V12, P245, DOI 10.1002/glia.440120402

Sorg C, 1992, Behring Inst Mitt, P126

STEVENS A, 1993, J NEUROL SCI, V118, P117, DOI 10.1016/0022-510X(93)90100-D

STREIT WJ, 1989, J NEUROIMMUNOL, V21, P117, DOI 10.1016/0165-5728(89)90167-7

Tanaka S, 1998, J NEUROSCI, V18, P6358

TATOR CH, 1995, BRAIN PATHOL, V5, P407, DOI 10.1111/j.1750-3639.1995.tb00619.x

UNTERHARNSCHEID.F, 1993, PATHOLOGIE NERVENSYS, P396

Verkhratsky A, 1996, TRENDS NEUROSCI, V19, P346, DOI 10.1016/0166-2236(96)10048-5

NR 58

TC 97

Z9 108

U1 0

U2 2

PU SPRINGER VERLAG

PI NEW YORK

PA 175 FIFTH AVE, NEW YORK, NY 10010 USA

SN 0001-6322

J9 ACTA NEUROPATHOL

JI Acta Neuropathol.

PD SEP

PY 2000

VL 100

IS 3

BP 313

EP 322

DI 10.1007/s004019900172

PG 10

WC Clinical Neurology; Neurosciences; Pathology

WE Science Citation Index Expanded (SCI-EXPANDED)

SC Neurosciences & Neurology; Pathology

GA 336MW

UT WOS:000088307700011

PM 10965802

DA 2024-03-03

ER

PT J

AU Beschorner, R

Engel, S

Mittelbronn, M

Adjodah, D

Dietz, K

Schluesener, HJ

Meyermann, R

AF Beschorner, R

Engel, S

Mittelbronn, M

Adjodah, D

Dietz, K

Schluesener, HJ

Meyermann, R

TI Differential regulation of the monocytic calcium-binding peptides

macrophage-inhibiting factor related protein-8 (MRP8/S100A8) and

allograft inflammatory factor-1 (AIF-1) following human traumatic brain

injury

SO ACTA NEUROPATHOLOGICA

LA English

DT Article

DE allograft-inflammatory factor-1; microglia response factor-1;

macrophage-inhibiting factor related-protein-8/S 100A8; traumatic brain

injury; human

ID FACTOR-I; ACTIVATED MACROPHAGES; MICROGLIAL CELLS; EXPRESSION; LESIONS;

MRP14; ENCEPHALOMYELITIS; POLYPEPTIDE; CONTUSION; NEURITIS

AB Intracellular calcium (Ca2+) has been shown to function as second messenger and to be associated with activation of different cell types including microglia. Previously, in human focal cerebral infarctions an early expression of macrophage-related protein-8 (MRP8/ S100A8), a member of the Ca2+-binding S100-protein family, in microglia has been reported. On the other hand, a delayed activation of microglia was observed following traumatic brain injury (TBI). We therefore examined immunohistochemically microglial expression of MRP8 and allograft inflammatory factor-1 (AIF-1), identical to microglial response factor-1 (mrf-1) and ionized calcium binding adaptor molecule-1 (iba1) in human brains after TBI and in control brains. Both, MRP8 and AIF-1 are Ca2+-binding peptides which have been associated with microglial activation in experimental models and in human cerebral infarctions. Detection of AIF-1 in controls confirmed constitutive expression of this peptide in a subset of microglial cells. After TBI, the density of AIF-1(+) microglia did not increase significantly. Lesional expression of AIF-1 did not significantly differ from other brain regions. Furthermore, following TBI, we found no significant differences in the density of AIF-1+ microglia as compared to controls. Microglial MRP8 expression was not detectable in controls and within the first 3 days post TBI, but increased rapidly after 3 days post TBI, suggesting a subpopulation of microglial cells to be AIF-1(-)/MRP8(+). We conclude that the delayed expression of MRP8 and the lack of AIF-1 up-regulation in microglia after TBI is in contrast to ischemic brain lesions and might reflect different activation cascades of microglia.

C1 Univ Tubingen, Inst Brain Res, D-72076 Tubingen, Germany.

Univ Tubingen, Dept Med Biometry, D-72076 Tubingen, Germany.

C3 Eberhard Karls University of Tubingen; Eberhard Karls University of

Tubingen; Eberhard Karls University Hospital

RP Beschorner, R (corresponding author), Univ Tubingen, Inst Brain Res, Calwerstr 3, D-72076 Tubingen, Germany.

RI Beschorner, Rudi/M-6397-2014; Dietz, Klaus/R-9268-2016

OI Dietz, Klaus/0000-0001-8503-9737; Mittelbronn,

Michel/0000-0002-2998-052X; Beschorner, Rudi/0000-0003-1109-915X

CR Abe M, 1999, J NEUROL, V246, P358, DOI 10.1007/s004150050363

Autieri MV, 1996, BIOCHEM BIOPH RES CO, V228, P29, DOI 10.1006/bbrc.1996.1612

Chen ZW, 1997, P NATL ACAD SCI USA, V94, P13879, DOI 10.1073/pnas.94.25.13879

ENGEL S, 1996, NACTA NEUROCHIR WI S, V66, P89

ENGEL S, 2000, IN PRESS ACTA NEUROP

GILCHRIST JSC, 1994, MOL CELL BIOCHEM, V135, P79, DOI 10.1007/BF00925963

Graeber MB, 1998, BRAIN RES, V813, P241, DOI 10.1016/S0006-8993(98)00859-2

Graham DI, 1997, GREENFIELDS NEUROPAT, P197

Holmin S, 1998, NEUROSURGERY, V42, P291, DOI 10.1097/00006123-199802000-00047

Holmin S, 1997, J NEUROSURG, V86, P493, DOI 10.3171/jns.1997.86.3.0493

Imai Y, 1996, BIOCHEM BIOPH RES CO, V224, P855, DOI 10.1006/bbrc.1996.1112

Ito D, 1998, MOL BRAIN RES, V57, P1, DOI 10.1016/S0169-328X(98)00040-0

LAGASSE E, 1992, BLOOD, V79, P1907

LAN HY, 1995, J HISTOCHEM CYTOCHEM, V43, P97, DOI 10.1177/43.1.7822770

Lugering N, 1998, Z GASTROENTEROL, V36, P173

MAHNKE K, 1995, J LEUKOCYTE BIOL, V57, P63, DOI 10.1002/jlb.57.1.63

MATTIACE LA, 1990, AM J PATHOL, V136, P1101

McLarnon JG, 1997, NEUROSCIENCE, V78, P1217, DOI 10.1016/S0306-4522(96)00680-X

Moller T, 1997, J NEUROSCI, V17, P615

Postler E, 1997, GLIA, V19, P27, DOI 10.1002/(SICI)1098-1136(199701)19:1<27::AID-GLIA3>3.0.CO;2-7

Postler E, 2000, J NEUROIMMUNOL, V104, P85, DOI 10.1016/S0165-5728(99)00222-2

ROCHE E, 1994, CELL CALCIUM, V16, P331, DOI 10.1016/0143-4160(94)90097-3

ROTH J, 1994, BIOCHEM J, V301, P655, DOI 10.1042/bj3010655

Schafer BW, 1996, TRENDS BIOCHEM SCI, V21, P134, DOI 10.1016/S0968-0004(96)80167-8

Schluesener HJ, 1999, ACTA NEUROPATHOL, V97, P119, DOI 10.1007/s004010050964

Schluesener HJ, 1998, GLIA, V24, P244, DOI 10.1002/(SICI)1098-1136(199810)24:2<244::AID-GLIA9>3.0.CO;2-3

Streit Wolfgang J., 1995, P85

Tanaka S, 1998, J NEUROSCI, V18, P6358

UTANS U, 1995, J CLIN INVEST, V95, P2954, DOI 10.1172/JCI118003

Utans U, 1996, TRANSPLANTATION, V61, P1387, DOI 10.1097/00007890-199605150-00018

Wege H, 1998, ADV EXP MED BIOL, V440, P437

NR 31

TC 27

Z9 28

U1 0

U2 6

PU SPRINGER-VERLAG

PI NEW YORK

PA 175 FIFTH AVE, NEW YORK, NY 10010 USA

SN 0001-6322

J9 ACTA NEUROPATHOL

JI Acta Neuropathol.

PD DEC

PY 2000

VL 100

IS 6

BP 627

EP 634

DI 10.1007/s004010000232

PG 8

WC Clinical Neurology; Neurosciences; Pathology

WE Science Citation Index Expanded (SCI-EXPANDED)

SC Neurosciences & Neurology; Pathology

GA 365ZY

UT WOS:000089981400006

PM 11078214

OA Green Published, Bronze

DA 2024-03-03

ER

PT J

AU Beschorner, R

Schluesener, HJ

Nguyen, TD

Magdolen, V

Luther, T

Pedal, I

Mattern, R

Meyermann, R

Schwab, JM

AF Beschorner, R

Schluesener, HJ

Nguyen, TD

Magdolen, V

Luther, T

Pedal, I

Mattern, R

Meyermann, R

Schwab, JM

TI Lesion-associated accumulation of uPAR/CD87-expressing infiltrating

granulocytes, activated microglial cells/macrophages and upregulation by

endothelial cells following TBI and FCI in humans

SO NEUROPATHOLOGY AND APPLIED NEUROBIOLOGY

LA English

DT Article

DE fibrinolysis; human; ischaemia; tissue remodelling; traumatic brain

injury; urokinase-type plasminogen activator receptor

ID PLASMINOGEN-ACTIVATOR; UROKINASE RECEPTOR; MESSENGER-RNA; MATRIX

METALLOPROTEINASES; CLINICAL-TRIALS; BRAIN EDEMA; U937 CELLS;

EXPRESSION; MODULATION; SYSTEM

AB Urokinase-type plasminogen activator receptor (uPAR/CD87) together with its ligand, urokinase-type plasminogen activator (uPA), constitutes a proteolytic system associated with tissue remodelling and leucocyte infiltration. uPAR is a member of the glycosyl phosphatidyl inositol (GPI) anchored protein family. The functional role of uPAR comprises fibrinolysis by conversion of plasminogen to plasmin. In addition, uPAR promotes cell adhesion, migration, proliferation, re-organization of the actin cytoskeleton, and angiogenesis. Furthermore, uPAR is involved in prevention of scar formation and is chemoattractant to macrophages and leucocytes. In order to investigate the pathophysiological role of uPAR following human CNS injury we examined necrotic brain lesions resulting from traumatic brain injury (TBI; n = 28) and focal cerebral infarctions (FCI; n = 17) by immunohistochemistry. Numbers of uPAR(+) cells and uPAR(+) blood vessels were counted. Following brain damage, uPAR(+) cells increased significantly within 12 h, reached a maximum after 3-4 days and remained elevated until later stages. uPAR was expressed by infiltrating granulocytes, activated microglia/macrophages and endothelial cells. Numbers of uPAR(+) vessels increased in parallel subsiding earlier following FCI than post TBI. The restricted, lesion-associated accumulation of uPAR(+) cells in the brain parenchyma and upregulated expression by endothelial cells suggests a crucial role for the influx of inflammatory cells and blood-brain barrier (BBB) disturbance. Through a failure in BBB function, uPAR participates in formation of brain oedema and thus contributes to secondary brain damage. In conclusion, the study defines the localization, kinetic course and cellular source of uPAR as a potential pharmacological target following human TBI and FCI.

C1 Univ Tubingen, Inst Brain Res, D-72076 Tubingen, Germany.

Tech Univ Munich, Klinikum Rechts Isar, Dept Gynecol, D-8000 Munich, Germany.

Tech Univ Dresden, Inst Pathol, D-8027 Dresden, Germany.

C3 Eberhard Karls University of Tubingen; Technical University of Munich;

Technische Universitat Dresden

RP Beschorner, R (corresponding author), Univ Tubingen, Inst Brain Res, Calwer Str 3, D-72076 Tubingen, Germany.

EM rudi.beschorner@med.uni-tuebingen.de

RI Beschorner, Rudi/M-6397-2014

OI Beschorner, Rudi/0000-0003-1109-915X; Schwab, Jan/0000-0001-6784-4919

CR Abbott NJ, 2000, CELL MOL NEUROBIOL, V20, P131, DOI 10.1023/A:1007074420772

Beschorner R, 2000, ACTA NEUROPATHOL, V100, P377, DOI 10.1007/s004010000202

Chen ZL, 1997, CELL, V91, P917, DOI 10.1016/S0092-8674(00)80483-3

Cross AK, 1999, GLIA, V28, P183, DOI 10.1002/(SICI)1098-1136(199912)28:3<183::AID-GLIA2>3.0.CO;2-3

De Keyser J, 1999, TRENDS NEUROSCI, V22, P535, DOI 10.1016/S0166-2236(99)01463-0

Dietzmann K, 2000, PATHOL RES PRACT, V196, P15, DOI 10.1016/S0344-0338(00)80017-5

Fauser S, 2000, J NEUROIMMUNOL, V111, P234, DOI 10.1016/S0165-5728(00)00368-4

Fujimoto J, 1996, J STEROID BIOCHEM, V59, P1, DOI 10.1016/S0960-0760(96)00084-2

Heymans S, 1999, NAT MED, V5, P1135, DOI 10.1038/13459

Higazi AAR, 1996, BIOCHEMISTRY-US, V35, P6884, DOI 10.1021/bi9514774

HOOGERWERF WA, 1994, GENE CHROMOSOME CANC, V9, P88, DOI 10.1002/gcc.2870090203

KALIMO H, 1997, GREENFIELDS NEUROPAT, P315

LAN HY, 1995, J HISTOCHEM CYTOCHEM, V43, P97, DOI 10.1177/43.1.7822770

LAURSEN H, 1993, ACTA NEUROPATHOL, V86, P378, DOI 10.1007/BF00369451

Lee JM, 1999, NATURE, V399, pA7, DOI 10.1038/399a007

LUND LR, 1991, J BIOL CHEM, V266, P5177

Luther T, 1997, AM J PATHOL, V150, P1231

MAGDOLEN V, 1995, ELECTROPHORESIS, V16, P813, DOI 10.1002/elps.11501601134

MENZIES SA, 1993, J NEUROSURG, V78, P257, DOI 10.3171/jns.1993.78.2.0257

Morganti-Kossmann MC, 1999, J NEUROTRAUM, V16, P617, DOI 10.1089/neu.1999.16.617

Moser KL, 1998, P NATL ACAD SCI USA, V95, P14869, DOI 10.1073/pnas.95.25.14869

Nonaka T, 2000, J RHEUMATOL, V27, P997

Ober C, 1998, HUM MOL GENET, V7, P1393, DOI 10.1093/hmg/7.9.1393

Peiretti F, 1996, BLOOD, V87, P162

Piguet PF, 1999, CIRCULATION, V99, P3315, DOI 10.1161/01.CIR.99.25.3315

PLOUG M, 1992, BLOOD, V79, P1447

Reid E, 2000, AM J HUM GENET, V66, P728, DOI 10.1086/302783

Reinert MM, 1999, NEUROL RES, V21, P330, DOI 10.1080/01616412.1999.11740940

Reuning U, 1998, INT J ONCOL, V13, P893

Shetty S, 1998, ARCH BIOCHEM BIOPHYS, V356, P265, DOI 10.1006/abbi.1998.0789

Shimazaki C, 1999, BRIT J HAEMATOL, V104, P672, DOI 10.1111/j.1365-2141.1999.01239.x

Speth C, 1998, IMMUNOBIOLOGY, V199, P152, DOI 10.1016/S0171-2985(98)80071-5

Stichel CC, 1999, NEUROSCIENCE, V93, P321, DOI 10.1016/S0306-4522(99)00112-8

Stratakis CA, 1998, J CLIN ENDOCR METAB, V83, P2972, DOI 10.1210/jc.83.8.2972

TranThang C, 1996, BRIT J CANCER, V74, P846, DOI 10.1038/bjc.1996.447

TSIRKA SE, 1995, NATURE, V377, P340, DOI 10.1038/377340a0

Washington RA, 1996, J NEUROSCI RES, V45, P392

YAMAMOTO M, 1994, CANCER RES, V54, P5016

Yong VW, 1998, TRENDS NEUROSCI, V21, P75

Yoshida E, 1996, INFLAMMATION, V20, P319, DOI 10.1007/BF01488206

NR 40

TC 35

Z9 41

U1 0

U2 0

PU WILEY

PI HOBOKEN

PA 111 RIVER ST, HOBOKEN 07030-5774, NJ USA

SN 0305-1846

EI 1365-2990

J9 NEUROPATH APPL NEURO

JI Neuropathol. Appl. Neurobiol.

PD DEC

PY 2000

VL 26

IS 6

BP 522

EP 527

DI 10.1046/j.0305-1846.2000.287.x

PG 6

WC Clinical Neurology; Neurosciences; Pathology

WE Science Citation Index Expanded (SCI-EXPANDED)

SC Neurosciences & Neurology; Pathology

GA 384XL

UT WOS:000165972500005

PM 11123718

DA 2024-03-03

ER

PT J

AU Lenzlinger, PM

Hans, VHJ

Joller-Jemelka, HI

Trentz, O

Morganti-Kossmann, MC

Kossmann, T

AF Lenzlinger, PM

Hans, VHJ

Joller-Jemelka, HI

Trentz, O

Morganti-Kossmann, MC

Kossmann, T

TI Markers for cell-mediated immune response are elevated in cerebrospinal

fluid and serum after severe traumatic brain injury in humans

SO JOURNAL OF NEUROTRAUMA

LA English

DT Article

DE blood-brain barrier; cellular immunity; cerebrospinal fluid; human brain

injuries; immune markers; microglia activation; T-cell activation

ID SEVERE HEAD-INJURY; TUMOR-NECROSIS-FACTOR; ACUTE-PHASE RESPONSE;

SPINAL-CORD INJURY; RAT-BRAIN; BETA-2-MICROGLOBULIN LEVELS;

INTERLEUKIN-2 RECEPTOR; INFLAMMATORY RESPONSE; MESSENGER-RNA;

INTERFERON-GAMMA

AB The brain is believed to be an immunologically privileged organ, sheltered from the systemic immunological defense by the blood-brain barrier (BBB). However, there is increasing evidence for a marked inflammatory response in the brain after traumatic brain injury (TBI), Markers for cellular immune activation, neopterin, beta2-microglobulin (beta 2M), and soluble interleukin-2 receptor (sIL-2R), were measured for up to 3 weeks in cerebrospinal fluid (CSF) and serum of 41 patients with severe TBI in order to elucidate the time course and the origin of the cellular immune response following TBI. Neopterin gradually increased during the first posttraumatic week in both CSF and serum. Concentrations in CSF were generally higher than in serum, suggesting intrathecal release of this marker. beta 2M showed similar kinetics but with higher serum than CSF concentrations. Nonetheless, intrathecal release as assessed by the beta 2M index could be postulated for most of the patients. The mean levels of sIL-2R in both CSF and serum were elevated during the whole study period, serum concentrations being up to 2 x 10(4) times higher than in CSF. No significant intrathecal production of sIL-2R could be detected. The present data shows that severe TBI leads to a marked cell-mediated immune response within the brain and in the systemic circulation. In the intrathecal compartment the activated cells appear to be predominantly of the macrophage/microglia lineage, while the immune activation in the systemic circulation seems to involve mainly T-lymphocytes.

C1 Univ Zurich Hosp, Div Trauma Surg, CH-8091 Zurich, Switzerland.

Univ Zurich Hosp, Dept Surg, Div Res, CH-8091 Zurich, Switzerland.

Univ Zurich Hosp, Dept Internal Med, Div Clin Immunol, Zurich, Switzerland.

Univ Hosp, Inst Neuropathol, Bonn, Germany.

C3 University of Zurich; University Zurich Hospital; University of Zurich;

University Zurich Hospital; University of Zurich; University Zurich

Hospital; University of Bonn

RP Lenzlinger, PM (corresponding author), Univ Penn, Dept Neurosurg, 105 Hayden Hall,3320 Smith Walk, Philadelphia, PA 19104 USA.

RI Lenzlinger, Philipp M/A-3822-2008

OI Morganti-Kossmann, Cristina/0000-0002-0807-2063

CR ADACHI N, 1991, EUR NEUROL, V31, P181, DOI 10.1159/000116674

AIHARA N, 1995, J NEUROTRAUM, V12, P53, DOI 10.1089/neu.1995.12.53

BALOGH D, 1992, BURNS, V18, P185, DOI 10.1016/0305-4179(92)90068-6

BANATI RB, 1993, GLIA, V7, P11

Barzo P, 1996, J NEUROSURG, V85, P1113, DOI 10.3171/jns.1996.85.6.1113

Baskaya MK, 1997, NEUROSCI LETT, V226, P33

CHIOLERO R, 1989, JPEN-PARENTER ENTER, V13, P5, DOI 10.1177/014860718901300105

CHUGANI DC, 1991, J NEUROSCI, V11, P256

COHEN J, 1991, HUM REPROD, V6, P118, DOI 10.1093/oxfordjournals.humrep.a137244

Csuka E, 2000, NEUROREPORT, V11, P2587, DOI 10.1097/00001756-200008030-00047

Csuka E, 1999, J NEUROIMMUNOL, V101, P211, DOI 10.1016/S0165-5728(99)00148-4

Fan L, 1996, MOL BRAIN RES, V36, P287, DOI 10.1016/0169-328X(95)00274-V

Fan Lei, 1995, Molecular Brain Research, V30, P125, DOI 10.1016/0169-328X(94)00287-O

FUKUDA K, 1995, J NEUROTRAUM, V12, P315, DOI 10.1089/neu.1995.12.315

GIULIAN D, 1989, J NEUROSCI, V9, P4416

GOODMAN JC, 1990, J NEUROIMMUNOL, V30, P213

Hagberg L, 2000, J NEUROIMMUNOL, V102, P51, DOI 10.1016/S0165-5728(99)00150-2

Hamerlinck FFV, 1999, EXP DERMATOL, V8, P167, DOI 10.1111/j.1600-0625.1999.tb00367.x

Hans VHJ, 1999, J CEREBR BLOOD F MET, V19, P184, DOI 10.1097/00004647-199902000-00010

Hausmann R, 1999, INT J LEGAL MED, V112, P227, DOI 10.1007/s004140050241

Hicks RR, 1997, MOL CHEM NEUROPATHOL, V32, P1, DOI 10.1007/BF02815164

Holmin S, 1998, NEUROSURGERY, V42, P291, DOI 10.1097/00006123-199802000-00047

Holmin S, 1997, J NEUROSURG, V86, P493, DOI 10.3171/jns.1997.86.3.0493

HOLMIN S, 1995, ACTA NEUROCHIR, V132, P110, DOI 10.1007/BF01404857

HOYT DB, 1990, J TRAUMA, V30, P759, DOI 10.1097/00005373-199007000-00001

HUBER C, 1984, J EXP MED, V160, P310, DOI 10.1084/jem.160.1.310

JENNETT B, 1975, LANCET, V1, P480

KITTUR SD, 1990, ANN NEUROL, V28, P168, DOI 10.1002/ana.410280209

Knoblach SM, 1999, J NEUROIMMUNOL, V95, P115, DOI 10.1016/S0165-5728(98)00273-2

KOREMATSU K, 1994, J CEREBR BLOOD F MET, V14, P825, DOI 10.1038/jcbfm.1994.103

KOSSMANN T, 1995, SHOCK, V4, P311, DOI 10.1097/00024382-199511000-00001

Kossmann T, 1997, J CEREBR BLOOD F MET, V17, P280, DOI 10.1097/00004647-199703000-00005

Kossmann T, 1996, BRAIN RES, V713, P143, DOI 10.1016/0006-8993(95)01501-9

KREUTZBERG GW, 1995, ARZNEIMITTEL-FORSCH, V45-1, P357

Kreutzberg GW, 1996, TRENDS NEUROSCI, V19, P312, DOI 10.1016/0166-2236(96)10049-7

LAMELIN JP, 1982, CLIN IMMUNOL IMMUNOP, V24, P55, DOI 10.1016/0090-1229(82)90088-5

LINK H, 1977, SCAND J CLIN LAB INV, V37, P397, DOI 10.3109/00365517709091498

MARSHALL LF, 1992, J NEUROTRAUM, V9, pS287

MARTINEZ M, 1993, J NEUROIMMUNOL, V48, P235, DOI 10.1016/0165-5728(93)90197-7

MATHIESEN T, 1990, J NEUROSURG, V73, P69, DOI 10.3171/jns.1990.73.1.0069

MCCLAIN CJ, 1987, J LAB CLIN MED, V110, P48

McIntosh T K, 1987, Cent Nerv Syst Trauma, V4, P119

Mereiter K., 1981, FORTSCHR MINERAL, V59, P125

MINAMI Y, 1993, ANNU REV IMMUNOL, V11, P245, DOI 10.1146/annurev.iy.11.040193.001333

Morganti-Kossmann MC, 1999, J NEUROTRAUM, V16, P617, DOI 10.1089/neu.1999.16.617

MorgantiKossman MC, 1997, MOL PSYCHIATR, V2, P133, DOI 10.1038/sj.mp.4000227

MORGANTIKOSSMANN MC, 1992, TRENDS PHARMACOL SCI, V13, P286, DOI 10.1016/0165-6147(92)90087-M

NILSSON K, 1974, TRANSPLANT REV, V21, P53

OTT L, 1994, J NEUROTRAUM, V11, P447, DOI 10.1089/neu.1994.11.447

OTT M, 1993, J NEUROL, V241, P108, DOI 10.1007/BF00869773

PIANI D, 1991, NEUROSCI LETT, V133, P159, DOI 10.1016/0304-3940(91)90559-C

PIEK J, 1992, J NEUROSURG, V77, P901, DOI 10.3171/jns.1992.77.6.0901

Pleines UE, 1998, J NEUROTRAUM, V15, P399, DOI 10.1089/neu.1998.15.399

Popovich PG, 1997, J COMP NEUROL, V377, P443, DOI 10.1002/(SICI)1096-9861(19970120)377:3<443::AID-CNE10>3.0.CO;2-S

QUATTROCCHI K B, 1990, Journal of Neurotrauma, V7, P77, DOI 10.1089/neu.1990.7.77

QUATTROCCHI KB, 1992, J NEUROTRAUM, V9, P1, DOI 10.1089/neu.1992.9.1

QUATTROCCHI KB, 1992, J NEUROSURG, V77, P694, DOI 10.3171/jns.1992.77.5.0694

REIBER H, 1980, J NEUROL, V224, P89, DOI 10.1007/BF00313347

REIBER H, 1987, CLIN CHIM ACTA, V163, P319, DOI 10.1016/0009-8981(87)90250-6

ROSS SA, 1994, BRIT J NEUROSURG, V8, P419, DOI 10.3109/02688699408995109

Rostworowski M, 1997, J NEUROSCI, V17, P3664

RUBIN LA, 1990, ANN INTERN MED, V113, P619, DOI 10.7326/0003-4819-113-8-619

Shohami E, 1999, J NEUROTRAUM, V16, P365, DOI 10.1089/neu.1999.16.365

SHOHAMI E, 1994, J CEREBR BLOOD F MET, V14, P615, DOI 10.1038/jcbfm.1994.76

Soares HD, 1995, J NEUROSCI, V15, P8223

SONNERBORG AB, 1989, AIDS, V3, P277, DOI 10.1097/00002030-198905000-00005

Spranger M, 1996, NEUROSCIENTIST, V2, P293, DOI 10.1177/107385849600200515

STREIT WJ, 1988, GLIA, V1, P301, DOI 10.1002/glia.440010502

TAUPIN V, 1993, J NEUROIMMUNOL, V42, P177, DOI 10.1016/0165-5728(93)90008-M

TEASDALE G, 1974, LANCET, V2, P81

Teasdale G, 1979, Acta Neurochir Suppl (Wien), V28, P13

TENHUNEN R, 1978, ACTA NEUROL SCAND, V58, P366

Teodorczyk-Injeyan J A, 1989, J Burn Care Rehabil, V10, P112, DOI 10.1097/00004630-198903000-00003

TEODORCZYKINJEYAN JA, 1989, CLIN IMMUNOL IMMUNOP, V51, P205, DOI 10.1016/0090-1229(89)90020-2

TEODORCZYKINJEYAN JA, 1991, CLIN EXP IMMUNOL, V85, P515

THOMAS WE, 1992, BRAIN RES REV, V17, P61

WALDMANN TA, 1989, ANNU REV BIOCHEM, V58, P875, DOI 10.1146/annurev.bi.58.070189.004303

Whalen MJ, 2000, CRIT CARE MED, V28, P929, DOI 10.1097/00003246-200004000-00003

WOODROOFE MN, 1991, J NEUROIMMUNOL, V33, P227, DOI 10.1016/0165-5728(91)90110-S

YAMASHITA K, 1994, J NEUROCHEM, V63, P1042

YOUNG AB, 1988, J NEUROSURG, V69, P375, DOI 10.3171/jns.1988.69.3.0375

Zhang ZY, 1996, J COMP NEUROL, V371, P485

ZHUANG J, 1993, J TRAUMA, V35, P415, DOI 10.1097/00005373-199309000-00014

NR 83

TC 57

Z9 61

U1 2

U2 9

PU MARY ANN LIEBERT INC PUBL

PI LARCHMONT

PA 2 MADISON AVENUE, LARCHMONT, NY 10538 USA

SN 0897-7151

J9 J NEUROTRAUM

JI J. Neurotrauma

PD MAY

PY 2001

VL 18

IS 5

BP 479

EP 489

DI 10.1089/089771501300227288

PG 11

WC Critical Care Medicine; Clinical Neurology; Neurosciences

WE Science Citation Index Expanded (SCI-EXPANDED)

SC General & Internal Medicine; Neurosciences & Neurology

GA 435CN

UT WOS:000168860000001

PM 11393251

DA 2024-03-03

ER

PT J

AU Lenzlinger, PM

Morganti-Kossmann, MC

Laurer, HL

McIntosh, TK

AF Lenzlinger, PM

Morganti-Kossmann, MC

Laurer, HL

McIntosh, TK

TI The duality of the inflammatory response to traumatic brain injury

SO MOLECULAR NEUROBIOLOGY

LA English

DT Review

DE traumatic brain injury; inflammation; neurons; astrocytes; microglia;

blood brain barrier; cytokines; interleukin-6; transforming growth

factor-beta; tumor necrosis factor-alpha

ID TUMOR-NECROSIS-FACTOR; SEVERE HEAD-INJURY; AMYLOID PRECURSOR PROTEIN;

CENTRAL-NERVOUS-SYSTEM; GROWTH-FACTOR-BETA; INTERCELLULAR-ADHESION

MOLECULE-1; TRANSIENT FOREBRAIN ISCHEMIA; CORTICAL IMPACT INJURY;

ACUTE-PHASE RESPONSE; ADULT-RAT BRAIN

AB One and a half to two million people sustain a traumatic brain injury (TBI) in the US each year, of which approx 70,000-90,000 will suffer from long-term disability with dramatic impacts on their own and their families' lives and enormous socio-economic costs. Brain damage following traumatic injury is a result of direct (immediate mechanical disruption of brain tissue, or primary injury) and indirect (secondary or delayed) mechanisms. These secondary mechanisms involve the initiation of an acute inflammatory response, including breakdown of the blood-brain barrier (BBB), edema formation and swelling, infiltration of peripheral blood cells and activation of resident immunocompetent cells, as well as the intrathecal release of numerous immune mediators such as interleukins and chemotactic factors. An overview over the inflammatory response to trauma as observed in clinical and in experimental TBI is presented in this review. The possibly harmful /beneficial sequelae of post-traumatic inflammation in the central nervous system (CNS) are discussed using three model mediators of inflammation in the brain, tumor necrosis factor-alpha (TNF-alpha), interleukin-6 (IL-6), and transforming growth factor-beta (TGF-beta). While the former two may act as important mediators for the initiation and the support of post-traumatic inflammation, thus causing additional cell death and neurologic dysfunction, they may also pave the way for reparative processes. TGF-beta, on the other hand, is a potent anti-inflammatory agent, which may also have some deleterious long-term effects in the injured brain. The implications of this duality of the post-traumatic inflammatory response for the treatment of brain-injured patients using anti-inflammatory strategies are discussed.

C1 Univ Penn, Dept Neurosurg, Philadelphia, PA 19104 USA.

Vet Adm Med Ctr, Philadelphia, PA 19104 USA.

Univ Zurich Hosp, Div Trauma Surg, CH-8091 Zurich, Switzerland.

C3 University of Pennsylvania; US Department of Veterans Affairs; Veterans

Health Administration (VHA); University of Zurich; University Zurich

Hospital

RP McIntosh, TK (corresponding author), Univ Penn, Dept Neurosurg, 105 Hayden Hall,3320 Smith Walk, Philadelphia, PA 19104 USA.

RI Lenzlinger, Philipp M/A-3822-2008

OI Morganti-Kossmann, Cristina/0000-0002-0807-2063

FU NIGMS NIH HHS [R01-GM34690] Funding Source: Medline; NINDS NIH HHS

[R01-NS40978, P50-NS08803, T32 NS07413-04] Funding Source: Medline

CR AIHARA N, 1995, J NEUROTRAUM, V12, P53, DOI 10.1089/neu.1995.12.53

[Anonymous], 2000, Eur J Trauma Emerg Surg, DOI [10.1007/s000680050007, DOI 10.1007/S000680050007]

Barzo P, 1996, J NEUROSURG, V85, P1113, DOI 10.3171/jns.1996.85.6.1113

Baskaya MK, 1997, NEUROSCI LETT, V226, P33

Bell MJ, 1997, J NEUROTRAUM, V14, P451, DOI 10.1089/neu.1997.14.451

Benveniste EN, 1998, CYTOKINE GROWTH F R, V9, P259, DOI 10.1016/S1359-6101(98)00015-X

BENVENISTE EN, 1995, INT J DEV NEUROSCI, V13, P341, DOI 10.1016/0736-5748(94)00061-7

CAMPBELL IL, 1993, P NATL ACAD SCI USA, V90, P10061, DOI 10.1073/pnas.90.21.10061

Carlos TM, 1997, J LEUKOCYTE BIOL, V61, P279, DOI 10.1002/jlb.61.3.279

CHAO CC, 1995, CLIN IMMUNOL IMMUNOP, V77, P358, DOI 10.1006/clin.1995.1163

COHEN J, 1991, HUM REPROD, V6, P118, DOI 10.1093/oxfordjournals.humrep.a137244

CORTEZ SC, 1989, BRAIN RES, V482, P271, DOI 10.1016/0006-8993(89)91190-6

Csuka E, 1999, J NEUROIMMUNOL, V101, P211, DOI 10.1016/S0165-5728(99)00148-4

DACUNHA A, 1992, J NEUROIMMUNOL, V36, P157

Emmerling MR, 2000, ANN NY ACAD SCI, V903, P118, DOI 10.1111/j.1749-6632.2000.tb06357.x

Fan L, 1996, MOL BRAIN RES, V36, P287, DOI 10.1016/0169-328X(95)00274-V

Fan Lei, 1995, Molecular Brain Research, V30, P125, DOI 10.1016/0169-328X(94)00287-O

Fattori E, 1995, EUR J NEUROSCI, V7, P2441, DOI 10.1111/j.1460-9568.1995.tb01042.x

FINCH CE, 1993, J CELL BIOCHEM, V53, P314, DOI 10.1002/jcb.240530408

Frautschy SA, 1996, NEUROBIOL AGING, V17, P311, DOI 10.1016/0197-4580(95)02073-X

FREI K, 1989, EUR J IMMUNOL, V19, P689, DOI 10.1002/eji.1830190418

FREI K, 1993, INT REV EXP PATHOL, V34, P183

FUKUDA K, 1995, J NEUROTRAUM, V12, P315, DOI 10.1089/neu.1995.12.315

GADIENT RA, 1990, NEUROSCI LETT, V117, P335, DOI 10.1016/0304-3940(90)90687-5

GOODMAN JC, 1990, J NEUROIMMUNOL, V30, P213

Graham DI, 1996, ACT NEUR S, V66, P96

GRAY CW, 1993, MOL BRAIN RES, V19, P251, DOI 10.1016/0169-328X(93)90037-P

GRIFFIN WST, 1994, NEUROSCI LETT, V176, P133, DOI 10.1016/0304-3940(94)90066-3

Gruol DL, 1997, MOL NEUROBIOL, V15, P307, DOI 10.1007/BF02740665

Hans VHJ, 1999, J CEREBR BLOOD F MET, V19, P184, DOI 10.1097/00004647-199902000-00010

Hans VHJ, 1999, NEUROREPORT, V10, P409

HENRICHNOACK P, 1994, J NEURAL TRANSM-SUPP, P33

Hicks RR, 1997, MOL CHEM NEUROPATHOL, V32, P1, DOI 10.1007/BF02815164

Holmin S, 1998, NEUROSURGERY, V42, P291, DOI 10.1097/00006123-199802000-00047

HOLMIN S, 1995, ACTA NEUROCHIR, V132, P110, DOI 10.1007/BF01404857

HOYT DB, 1990, J TRAUMA, V30, P759, DOI 10.1097/00005373-199007000-00001

Isaksson J, 1997, ACTA NEUROPATHOL, V94, P16, DOI 10.1007/s004010050666

Knoblach SM, 1999, J NEUROIMMUNOL, V95, P115, DOI 10.1016/S0165-5728(98)00273-2

Knoblach SM, 1998, EXP NEUROL, V153, P143, DOI 10.1006/exnr.1998.6877

KOLESNICK R, 1994, CELL, V77, P325, DOI 10.1016/0092-8674(94)90147-3

KOREMATSU K, 1994, J CEREBR BLOOD F MET, V14, P825, DOI 10.1038/jcbfm.1994.103

KOSSMANN T, 1995, SHOCK, V4, P311, DOI 10.1097/00024382-199511000-00001

Kossmann T, 1997, J CEREBR BLOOD F MET, V17, P280, DOI 10.1097/00004647-199703000-00005

Kossmann T, 1996, BRAIN RES, V713, P143, DOI 10.1016/0006-8993(95)01501-9

Kreutzberg GW, 1996, TRENDS NEUROSCI, V19, P312, DOI 10.1016/0166-2236(96)10049-7

KUSHIMA Y, 1992, NEUROSCI RES, V13, P267, DOI 10.1016/0168-0102(92)90039-F

Lehrmann E, 1998, GLIA, V24, P437, DOI 10.1002/(SICI)1098-1136(199812)24:4<437::AID-GLIA9>3.0.CO;2-X

Lenzlinger PM, 2001, J NEUROTRAUM, V18, P479, DOI 10.1089/089771501300227288

LEWEN A, 1995, NEUROREPORT, V6, P357, DOI 10.1097/00001756-199501000-00032

LOGAN A, 1994, EUR J NEUROSCI, V6, P355, DOI 10.1111/j.1460-9568.1994.tb00278.x

LOGAN A, 1993, TRENDS PHARMACOL SCI, V14, P337, DOI 10.1016/0165-6147(93)90007-7

MARMAROU A, 1991, J NEUROSURG S, V75, P59

März P, 1999, GLIA, V26, P191, DOI 10.1002/(SICI)1098-1136(199905)26:3<191::AID-GLIA1>3.0.CO;2-#

Mattson MP, 2001, J CLIN INVEST, V107, P247, DOI 10.1172/JCI11916

MAYEUX R, 1993, ANN NEUROL, V33, P494, DOI 10.1002/ana.410330513

MCCLAIN CJ, 1987, J LAB CLIN MED, V110, P48

Morganti-Kossmann MC, 1999, J NEUROTRAUM, V16, P617, DOI 10.1089/neu.1999.16.617

MorgantiKossman MC, 1997, MOL PSYCHIATR, V2, P133, DOI 10.1038/sj.mp.4000227

MORTIMER JA, 1991, INT J EPIDEMIOL, V20, P28

Nonaka M, 1999, J NEUROTRAUM, V16, P1023, DOI 10.1089/neu.1999.16.1023

OTT L, 1994, J NEUROTRAUM, V11, P447, DOI 10.1089/neu.1994.11.447

Penkowa M, 1999, GLIA, V25, P343

Perry VH, 1997, MOL MED TODAY, V3, P335, DOI 10.1016/S1357-4310(97)01077-0

PIEK J, 1992, J NEUROSURG, V77, P901, DOI 10.3171/jns.1992.77.6.0901

Pierce JES, 1996, J NEUROSCI, V16, P1083

Pleines UE, 1998, J NEUROTRAUM, V15, P399, DOI 10.1089/neu.1998.15.399

Popovich PG, 1997, J COMP NEUROL, V377, P443, DOI 10.1002/(SICI)1096-9861(19970120)377:3<443::AID-CNE10>3.0.CO;2-S

Qiu ZH, 1998, J NEUROSCI, V18, P10445

QUATTROCCHI K B, 1990, Journal of Neurotrauma, V7, P77, DOI 10.1089/neu.1990.7.77

QUATTROCCHI KB, 1992, J NEUROTRAUM, V9, P1, DOI 10.1089/neu.1992.9.1

QUATTROCCHI KB, 1992, J NEUROSURG, V77, P694, DOI 10.3171/jns.1992.77.5.0694

Raby CA, 1998, J NEUROCHEM, V71, P2505

Rancan M, 2001, J NEUROSCI RES, V63, P438, DOI 10.1002/1097-4547(20010301)63:5<438::AID-JNR1039>3.3.CO;2-G

REID TR, 1989, J BIOL CHEM, V264, P4583

Rimaniol AC, 1995, NEUROREPORT, V7, P133, DOI 10.1097/00001756-199512000-00032

ROBERTS GW, 1994, J NEUROL NEUROSUR PS, V57, P419, DOI 10.1136/jnnp.57.4.419

ROSS SA, 1994, BRIT J NEUROSURG, V8, P419, DOI 10.3109/02688699408995109

Rostworowski M, 1997, J NEUROSCI, V17, P3664

ROTHWELL NJ, 1995, TRENDS NEUROSCI, V18, P130, DOI 10.1016/0166-2236(95)93890-A

Ruocco A, 1999, J CEREBR BLOOD F MET, V19, P1345, DOI 10.1097/00004647-199912000-00008

Scherbel U, 1999, P NATL ACAD SCI USA, V96, P8721, DOI 10.1073/pnas.96.15.8721

Shibayama M, 1996, J NEUROTRAUM, V13, P801, DOI 10.1089/neu.1996.13.801

Shohami E, 1999, CYTOKINE GROWTH F R, V10, P119, DOI 10.1016/S1359-6101(99)00008-8

SHOHAMI E, 1994, J CEREBR BLOOD F MET, V14, P615, DOI 10.1038/jcbfm.1994.76

Shohami E, 1996, J CEREBR BLOOD F MET, V16, P378, DOI 10.1097/00004647-199605000-00004

Smith DH, 1998, AM J PATHOL, V153, P1005, DOI 10.1016/S0002-9440(10)65643-X

SOARES HD, 1992, J NEUROCHEM, V58, P1845, DOI 10.1111/j.1471-4159.1992.tb10061.x

Soares HD, 1995, J NEUROSCI, V15, P8223

Spranger M, 1996, NEUROSCIENTIST, V2, P293, DOI 10.1177/107385849600200515

Stahel PF, 2000, J CEREBR BLOOD F MET, V20, P369, DOI 10.1097/00004647-200002000-00019

Stahel PF, 2000, J NEUROIMMUNOL, V109, P164, DOI 10.1016/S0165-5728(00)00304-0

Stover JF, 2000, NEUROSCI LETT, V288, P25, DOI 10.1016/S0304-3940(00)01187-3

Sullivan PG, 1999, J NEUROSCI, V19, P6248

TAUPIN V, 1993, J NEUROIMMUNOL, V42, P177, DOI 10.1016/0165-5728(93)90008-M

THOMAS WE, 1992, BRAIN RES REV, V17, P61

TOULMOND S, 1992, NEUROSCI LETT, V144, P49, DOI 10.1016/0304-3940(92)90713-H

Unterberg AW, 1997, ACT NEUR S, V70, P106

Van den Heuvel C, 1999, EXP NEUROL, V159, P441, DOI 10.1006/exnr.1999.7150

Vanden Berghe W, 2000, BIOCHEM PHARMACOL, V60, P1185, DOI 10.1016/S0006-2952(00)00412-3

WAHL SM, 1994, J EXP MED, V180, P1587, DOI 10.1084/jem.180.5.1587

Whalen MJ, 2000, CRIT CARE MED, V28, P929, DOI 10.1097/00003246-200004000-00003

WOODROOFE MN, 1991, J NEUROIMMUNOL, V33, P227, DOI 10.1016/0165-5728(91)90110-S

Wu JP, 2000, NEUROSCI LETT, V292, P203, DOI 10.1016/S0304-3940(00)01472-5

YAMASHITA K, 1994, J NEUROCHEM, V63, P1042

YOUNG AB, 1988, J NEUROSURG, V69, P375, DOI 10.3171/jns.1988.69.3.0375

ZHOU DH, 1991, FASEB J, V5, P2582, DOI 10.1096/fasebj.5.11.1868982

Zhu Y, 2000, BRAIN RES, V866, P286, DOI 10.1016/S0006-8993(00)02240-X

ZHUANG J, 1993, J TRAUMA, V35, P415, DOI 10.1097/00005373-199309000-00014

NR 108

TC 337

Z9 382

U1 3

U2 57

PU HUMANA PRESS INC

PI TOTOWA

PA 999 RIVERVIEW DRIVE SUITE 208, TOTOWA, NJ 07512 USA

SN 0893-7648

J9 MOL NEUROBIOL

JI Mol. Neurobiol.

PD AUG-DEC

PY 2001

VL 24

IS 1-3

BP 169

EP 181

PG 13

WC Neurosciences

WE Science Citation Index Expanded (SCI-EXPANDED)

SC Neurosciences & Neurology

GA 510YB

UT WOS:000173234200010

PM 11831551

DA 2024-03-03

ER

PT J

AU Van Landeghem, FKH

Stover, JF

Bechmann, I

Brück, W

Unterberg, A

Bührer, C

Von Deimling, A

AF Van Landeghem, FKH

Stover, JF

Bechmann, I

Brück, W

Unterberg, A

Bührer, C

Von Deimling, A

TI Early expression of glutamate transporter proteins in ramified microglia

after controlled cortical impact injury in the rat

SO GLIA

LA English

DT Article

DE astrocytes; controlled cortical impact injury; glutamate; glutamate

transporter; glutamate uptake; microglia; traumatic brain injury

ID TRAUMATIC BRAIN-INJURY; AMINO-ACID TRANSPORTER; RELEASE; LOCALIZATION;

GLT-1; EXCITOTOXICITY; INHIBITION; INCREASES; NEURONS; MODEL

AB Traumatic brain injury is followed by increased extracellular glutamate concentration. Uptake of glutamate is mainly mediated by the glial glutamate transporters GLAST and GLT-1. Extent and distribution of GLAST and GLT-1 were studied in a rat model of controlled cortical impact injury (CCH). Western Blot analysis revealed lowest levels of GLAST and GLT-1 with a decrease by 40%-54% and 42%-49% between 24 and 72 h posttrauma. By 8 h after CCll, CSF glutamate levels were increased (10.5 muM vs. 2.56 VM in controls; P < 0.001), reaching maximum values by 48 h. A significant increase in de novo GLAST and GLT-1 expressing ramified microglia was observed within 4 h, reached a stable level by 48 li, and remained high up to 72 h after CCH. Furthermore, ramified microglia de novo expressed the neuronal glutamate transporter EAAC1 after CCII Following CCII, GLAST/GLT-1 and GFAP coexpressing astrocytes were immediately reduced, reaching minimum levels within 8 h. This reduction of expression could be either due to protein downregulation or loss of astrocytes. At 72 h, a marked population of GLAST- and GLT-1-positive reactive astrocytes appeared. These results support the hypothesis that reduced astrocytic GLAST and GLT-1 protein levels following MI contribute to evolving secondary injury. Microglia are capable of de novo expressing glutamate transporter proteins, indicating that the expression of glial and neuronal glutamate transporters is not restricted to a specific glial or neuronal lineage. Ramified microglia may play an important compensatory role in the early regulation of extracellular glutamate after CCII. (C) 2001 Wiley-Liss, Inc.

C1 Humboldt Univ, Inst Neuropathol, Charite, D-13353 Berlin, Germany.

Humboldt Univ, Dept Neurosurg, Charite, D-13353 Berlin, Germany.

Humboldt Univ, Inst Anat, Dept Cell & Neurobiol, Charite, D-13353 Berlin, Germany.

Humboldt Univ, Dept Neonatol, Charite, D-13353 Berlin, Germany.

C3 Free University of Berlin; Humboldt University of Berlin; Charite

Universitatsmedizin Berlin; Free University of Berlin; Humboldt

University of Berlin; Charite Universitatsmedizin Berlin; Free

University of Berlin; Humboldt University of Berlin; Charite

Universitatsmedizin Berlin; Free University of Berlin; Humboldt

University of Berlin; Charite Universitatsmedizin Berlin

RP Van Landeghem, FKH (corresponding author), Humboldt Univ, Inst Neuropathol, Charite, Charite Campus Virchow Klinikum,Augustenburger Pl, D-13353 Berlin, Germany.

EM frank.van_landeghem@charite.de

RI von Deimling, Andreas/F-7774-2013

OI von Deimling, Andreas/0000-0002-5863-540X; van Landeghem,

Frank/0000-0002-9404-7031

CR Arriza JL, 1997, P NATL ACAD SCI USA, V94, P4155, DOI 10.1073/pnas.94.8.4155

BALEAR VJ, 1972, J NEUROCHEM, V19, P2657

BLINZING.K, 1968, Z ZELLFORSCH MIK ANA, V85, P145, DOI 10.1007/BF00325030

Carbonnel WS, 1999, ACTA NEUROPATHOL, V98, P396, DOI 10.1007/s004010051100

CHAUDHRY FA, 1995, NEURON, V15, P711, DOI 10.1016/0896-6273(95)90158-2

CHOI DW, 1992, J NEUROBIOL, V23, P1261, DOI 10.1002/neu.480230915

DANBOLT NC, 1992, NEUROSCIENCE, V51, P295, DOI 10.1016/0306-4522(92)90316-T

DIXON CE, 1991, J NEUROSCI METH, V39, P253

FADEN AI, 1989, SCIENCE, V244, P798, DOI 10.1126/science.2567056

FAIRMAN WA, 1995, NATURE, V375, P599, DOI 10.1038/375599a0

Haugeto O, 1996, J BIOL CHEM, V271, P27715, DOI 10.1074/jbc.271.44.27715

JOHNSTON GAR, 1981, GLUTAMATE TRANSMITTE, P77

KANAI Y, 1992, NATURE, V360, P467, DOI 10.1038/360467a0

KANNER BI, 1987, CRIT REV BIOCHEM MOL, V22, P1, DOI 10.3109/10409238709082546

KATAYAMA Y, 1990, J NEUROSURG, V73, P889, DOI 10.3171/jns.1990.73.6.0889

KIMELBERG HK, 1995, J CEREBR BLOOD F MET, V15, P409, DOI 10.1038/jcbfm.1995.51

Klose J, 1999, METH MOL B, V112, P67

KONDO K, 1995, NEUROSCI LETT, V188, P140, DOI 10.1016/0304-3940(95)11408-O

LEHRE KP, 1995, J NEUROSCI, V15, P1835

LEVY LM, 1993, FEBS LETT, V317, P79, DOI 10.1016/0014-5793(93)81495-L

LEVY LM, 1995, EUR J NEUROSCI, V7, P2036, DOI 10.1111/j.1460-9568.1995.tb00626.x

LI S, 1999, J NEUROSCI, V19, P1

LOGAN WJ, 1972, BRAIN RES, V42, P413, DOI 10.1016/0006-8993(72)90540-9

Lopez-Redondo F, 2000, MOL BRAIN RES, V76, P429, DOI 10.1016/S0169-328X(00)00022-X

Martin LJ, 1997, ANN NEUROL, V42, P335, DOI 10.1002/ana.410420310

NICHOLLS D, 1990, TRENDS PHARMACOL SCI, V11, P462, DOI 10.1016/0165-6147(90)90129-V

NILSSON P, 1990, J CEREBR BLOOD F MET, V10, P631, DOI 10.1038/jcbfm.1990.115

Noda M, 1999, NEUROSCIENCE, V92, P1465, DOI 10.1016/S0306-4522(99)00036-6

PALMER AM, 1993, J NEUROCHEM, V61, P2015, DOI 10.1111/j.1471-4159.1993.tb07437.x

PERRY VH, 1985, NEUROSCIENCE, V15, P313, DOI 10.1016/0306-4522(85)90215-5

PINES G, 1992, NATURE, V360, P464, DOI 10.1038/360464a0

Rao VLR, 1998, J NEUROCHEM, V70, P2020

Rao VLR, 2001, J NEUROSCI, V21, P1876, DOI 10.1523/JNEUROSCI.21-06-01876.2001

ROTHSTEIN JD, 1994, NEURON, V13, P713, DOI 10.1016/0896-6273(94)90038-8

Rothstein JD, 1996, NEURON, V16, P675, DOI 10.1016/S0896-6273(00)80086-0

ROTHSTEIN JD, 1993, P NATL ACAD SCI USA, V90, P6591, DOI 10.1073/pnas.90.14.6591

Rutledge EM, 1996, J NEUROSCI, V16, P7803

Rutledge EM, 1998, AM J PHYSIOL-CELL PH, V274, pC1511, DOI 10.1152/ajpcell.1998.274.6.C1511

SCHNEIDER GH, 1992, CAN J PHYSIOL PHARM, V70, pS334, DOI 10.1139/y92-280

STORCK T, 1992, P NATL ACAD SCI USA, V89, P10955, DOI 10.1073/pnas.89.22.10955

Stover JF, 2000, BRAIN RES, V875, P51, DOI 10.1016/S0006-8993(00)02597-X

STREIT WJ, 1985, J HISTOCHEM CYTOCHEM, V33, P1042, DOI 10.1177/33.10.4045182

STREIT WJ, 1987, J NEUROCYTOL, V16, P249, DOI 10.1007/BF01795308

STREIT WJ, 1988, J COMP NEUROL, V268, P248, DOI 10.1002/cne.902680209

Swanson RA, 1997, J NEUROSCI, V17, P932

TANAKA H, 1994, ACTA NEUROCHIR, P524

Tanaka K, 1997, SCIENCE, V276, P1699, DOI 10.1126/science.276.5319.1699

Watase K, 1998, EUR J NEUROSCI, V10, P976, DOI 10.1046/j.1460-9568.1998.00108.x

NR 48

TC 106

Z9 128

U1 0

U2 5

PU WILEY

PI HOBOKEN

PA 111 RIVER ST, HOBOKEN 07030-5774, NJ USA

SN 0894-1491

EI 1098-1136

J9 GLIA

JI Glia

PD SEP

PY 2001

VL 35

IS 3

BP 167

EP 179

DI 10.1002/glia.1082

PG 13

WC Neurosciences

WE Science Citation Index Expanded (SCI-EXPANDED)

SC Neurosciences & Neurology

GA 470TB

UT WOS:000170886300001

PM 11494408

DA 2024-03-03

ER

PT J

AU Schwab, JM

Seid, K

Schluesener, HJ

AF Schwab, JM

Seid, K

Schluesener, HJ

TI Traumatic brain injury induces prolonged accumulation of

cyclooxygenase-1 expressing microglia/brain macrophages in rats

SO JOURNAL OF NEUROTRAUMA

LA English

DT Article

DE bystander damage; inflammation; prostaglandin; tissue remodeling

ID SPINAL-CORD INJURY; CEREBRAL-ISCHEMIA; GENE DISRUPTION; EFFECTOR-CELLS;

IN-VIVO; INFLAMMATION; COX-1; DIFFERENTIATION; DISEASE; GLIOMA

AB Inflammatory cellular responses to brain injury are promoted by proinflammatory messengers. Cyclooxygenases (prostaglandin endoperoxide H synthases [PGH]) are key enzymes in the conversion of arachidonic acid into prostanoids, which mediate immunomodulation, mitogenesis, apoptosis, blood flow, secondary injury (lipid peroxygenation), and inflammation. Here, we report COX-1 expression following brain injury. In control brains, COX-1 expression was localized rarely to brain microglia/macrophages. One to 5 days after injury, we observed a highly significant (P < 0.0001) increase in COX-1(+) microglia/macrophages at perilesional areas and in the developing core with a delayed culmination of cell accumulation at day 7, correlating with phagocytic activity. There, cell numbers remained persistently elevated up to 21 days following injury. Further, COX-1(+) cells were located in perivascular Virchow-Robin spaces also reaching maximal numbers at day 7. Lesion-confined COX-1(+) vessels increased in numbers from day 1, reaching the maximum at days 5-7. Double-labeling experiments confirmed coexpression of COX-1 by ED-1(+) and OX-42(+) microglia/ macrophages. Transiently after injury, most COX-1(+) microglia/macrophages coexpress the activation antigen OX-6 (MHC class II). However, the prolonged accumulation of COX-1(+), ED-1(+) microglia/macrophages in lesional areas enduring the acute postinjury inflammatory response points to a role of COX-1 in the pathophysiology of secondary injury. We have identified localized, accumulated COX-1 expression as a potential pharmacological target in the treatment of brain injury. Our results suggest that therapeutic approaches based on long-term blocking including COX-1, might be superior to selective COX-2 blocking to suppress the local synthesis of prostanoids.

C1 Univ Tubingen, Sch Med, Inst Brain Res, D-72076 Tubingen, Germany.

C3 Eberhard Karls University of Tubingen

RP Schwab, JM (corresponding author), Univ Tubingen, Sch Med, Inst Brain Res, Calwer Str 3, D-72076 Tubingen, Germany.

OI Schwab, Jan/0000-0001-6784-4919

CR [Anonymous], NEUROTRAUMA

Becher B, 1996, GLIA, V18, P1

Crofford LJ, 1997, J RHEUMATOL, V24, P15

Dash PK, 2000, J NEUROTRAUM, V17, P69, DOI 10.1089/neu.2000.17.69

Deininger MH, 1999, J NEUROIMMUNOL, V95, P202, DOI 10.1016/S0165-5728(98)00257-4

Deininger MH, 1999, ACTA NEUROPATHOL, V98, P240, DOI 10.1007/s004010051075

Dirnagl U, 1999, TRENDS NEUROSCI, V22, P391, DOI 10.1016/S0166-2236(99)01401-0

Dubois RN, 1998, FASEB J, V12, P1063, DOI 10.1096/fasebj.12.12.1063

DUSART I, 1994, EUR J NEUROSCI, V6, P712, DOI 10.1111/j.1460-9568.1994.tb00983.x

Fujita T, 1998, ACTA NEUROCHIR, V140, P275, DOI 10.1007/s007010050095

Gilroy DW, 1999, NAT MED, V5, P698, DOI 10.1038/9550

GUILIAN D, 1993, GLIA, V7, P102

HOFF T, 1993, FEBS LETT, V320, P38, DOI 10.1016/0014-5793(93)81653-H

Iadecola C, 1999, ACTA NEUROPATHOL, V98, P9, DOI 10.1007/s004010051045

Kaplan MD, 1997, J BIOL CHEM, V272, P18534, DOI 10.1074/jbc.272.30.18534

Kreutzberg GW, 1996, TRENDS NEUROSCI, V19, P312, DOI 10.1016/0166-2236(96)10049-7

LANGENBACH R, 1995, CELL, V83, P483, DOI 10.1016/0092-8674(95)90126-4

Lee JM, 1999, NATURE, V399, pA7, DOI 10.1038/399a007

LEIBOVICH SJ, 1987, NATURE, V329, P630, DOI 10.1038/329630a0

MALLAT M, 1994, J LEUKOCYTE BIOL, V56, P416, DOI 10.1002/jlb.56.3.416

MATSUO M, 1995, BRAIN RES, V685, P201, DOI 10.1016/0006-8993(95)00490-H

Mattson MP, 1998, TRENDS NEUROSCI, V21, P53, DOI 10.1016/S0166-2236(97)01188-0

MAXWELL WL, 1990, PHILOS T R SOC B, V328, P479, DOI 10.1098/rstb.1990.0121

Minghetti L, 1998, PROG NEUROBIOL, V54, P99, DOI 10.1016/S0301-0082(97)00052-X

MORHAM SG, 1995, CELL, V83, P473, DOI 10.1016/0092-8674(95)90125-6

Nogawa S, 1997, J NEUROSCI, V17, P2746

Perry V H, 1992, Curr Opin Neurobiol, V2, P679, DOI 10.1016/0959-4388(92)90038-M

Popovich PG, 1997, J COMP NEUROL, V377, P443, DOI 10.1002/(SICI)1096-9861(19970120)377:3<443::AID-CNE10>3.0.CO;2-S

Portanova JP, 1996, J EXP MED, V184, P883, DOI 10.1084/jem.184.3.883

Rapalino O, 1998, NAT MED, V4, P814, DOI 10.1038/nm0798-814

Resnick DK, 1998, J NEUROTRAUM, V15, P1005, DOI 10.1089/neu.1998.15.1005

Roller A, 1999, BIOCHEM BIOPH RES CO, V259, P600, DOI 10.1006/bbrc.1999.0825

Sairanen T, 1998, ANN NEUROL, V43, P738, DOI 10.1002/ana.410430608

SANDERS KM, 1978, AM J PHYSIOL, V234, pE209, DOI 10.1152/ajpendo.1978.234.2.E209

Schwab JM, 2000, ACTA NEUROPATHOL, V99, P609, DOI 10.1007/s004010051170

SEIBERT K, 1994, P NATL ACAD SCI USA, V91, P12013, DOI 10.1073/pnas.91.25.12013

Shillabeer G, 1998, METABOLISM, V47, P461, DOI 10.1016/S0026-0495(98)90060-9

Smith CJ, 1998, P NATL ACAD SCI USA, V95, P13313, DOI 10.1073/pnas.95.22.13313

Smith WL, 1996, J BIOL CHEM, V271, P33157, DOI 10.1074/jbc.271.52.33157

Streit WJ, 1999, PROG NEUROBIOL, V57, P563, DOI 10.1016/S0301-0082(98)00069-0

Taoka Y, 1998, PROG NEUROBIOL, V56, P341, DOI 10.1016/S0301-0082(98)00049-5

Tsujii M, 1998, CELL, V93, P705, DOI 10.1016/S0092-8674(00)81433-6

WILBERGER JE, 1996, NEUROTRAUMA, P1219

Williams CS, 1996, AM J PHYSIOL, V270, P393

Willoughby DA, 2000, LANCET, V355, P646, DOI 10.1016/S0140-6736(99)12031-2

YAMAGATA K, 1993, NEURON, V11, P371, DOI 10.1016/0896-6273(93)90192-T

Yasojima K, 1999, BRAIN RES, V830, P226, DOI 10.1016/S0006-8993(99)01389-X

Yermakova AV, 1999, J NEUROPATH EXP NEUR, V58, P1135, DOI 10.1097/00005072-199911000-00003

NR 48

TC 44

Z9 47

U1 0

U2 0

PU MARY ANN LIEBERT INC PUBL

PI LARCHMONT

PA 2 MADISON AVENUE, LARCHMONT, NY 10538 USA

SN 0897-7151

J9 J NEUROTRAUM

JI J. Neurotrauma

PD SEP

PY 2001

VL 18

IS 9

BP 881

EP 890

DI 10.1089/089771501750451802

PG 10

WC Critical Care Medicine; Clinical Neurology; Neurosciences

WE Science Citation Index Expanded (SCI-EXPANDED)

SC General & Internal Medicine; Neurosciences & Neurology

GA 471TP

UT WOS:000170945000004

PM 11565600

DA 2024-03-03

ER

PT J

AU Kyrkanides, S

O'Banion, MK

Whiteley, PE

Daeschner, JC

Olschowka, JA

AF Kyrkanides, S

O'Banion, MK

Whiteley, PE

Daeschner, JC

Olschowka, JA

TI Enhanced glial activation and expression of specific CNS

inflammation-related molecules in aged versus young rats following

cortical stab injury

SO JOURNAL OF NEUROIMMUNOLOGY

LA English

DT Article

DE microglia; astrocytes; aging; brain trauma; neurodegenerative disease

ID BLOOD-BRAIN-BARRIER; EXPERIMENTAL AUTOIMMUNE ENCEPHALOMYELITIS; NF-KAPPA

B; MONONUCLEAR PHAGOCYTES; NERVOUS-SYSTEM; CYTOKINES; ASTROCYTES; ADULT;

INTERLEUKIN-10; MICROGLIA

AB Aging is associated with increased glial responsiveness that may enhance the brain's susceptibility to injury and disease. To determine whether unique age-related molecular responses occur in brain injury, we assessed m-RNA levels of representative central nervous system (CNS) inflammation-related molecules in young (3 months) and aged (36 months) Fisher 344/Brown Norwegian F1 hybrid rats following cortical stab. Enhanced glial activation in older animals was accompanied by increased expression of a subset of inflammation-related mRNAs, including IL-1 beta, TNF alpha, IL-6, ICAM-1, inducible nitric oxide synthase (iNOS), metalloprotednase-9 (NEAP-9), and complement 3 alpha -chain 1 (C3 alpha1). Recognition of these age-specific differences may guide development of novel treatment regimes for older individuals. (C) 2001 Elsevier Science BY. All rights reserved.

C1 Univ Rochester, Dept Neurobiol & Anat, Rochester, NY 14642 USA.

Univ Rochester, Sch Med & Dent, Dept Neurol, Rochester, NY 14642 USA.

Roche Biosci, Dept Inflammatory Dis, Palo Alto, CA 94394 USA.

C3 University of Rochester; University of Rochester; Roche Holding

RP Olschowka, JA (corresponding author), Univ Rochester, Dept Neurobiol & Anat, 601 Elmwood Ave,Box 603, Rochester, NY 14642 USA.

EM John_Olschowka@urmc.rochester.edu

OI O'Banion, M. Kerry/0000-0003-1246-3363

FU NIA NIH HHS [P60 AG10463] Funding Source: Medline; NINDS NIH HHS

[NS33553] Funding Source: Medline

CR Amat JA, 1996, GLIA, V16, P368, DOI 10.1002/(SICI)1098-1136(199604)16:4<368::AID-GLIA9>3.0.CO;2-W

BALASINGAM V, 1994, J NEUROSCI, V14, P846

BORRIELLO F, 1995, BIOTECHNIQUES, V19, P580

Dawson VL, 1998, PROG BRAIN RES, V118, P215

DIJKSTRA CD, 1994, J IMMUNOL METHODS, V174, P21, DOI 10.1016/0022-1759(94)90006-X

DIJKSTRA CD, 1985, IMMUNOLOGY, V54, P589

DiSanto E, 1997, EUR J PHARMACOL, V336, P197, DOI 10.1016/S0014-2999(97)01225-9

ENG LF, 1985, J NEUROIMMUNOL, V8, P203, DOI 10.1016/S0165-5728(85)80063-1

ENGELHARDT B, 1994, J NEUROIMMUNOL, V51, P199, DOI 10.1016/0165-5728(94)90082-5

Eralinna JP, 1996, J NEUROIMMUNOL, V66, P103, DOI 10.1016/0165-5728(96)00031-8

FERNAUDESPINOSA I, 1993, GLIA, V8, P277, DOI 10.1002/glia.440080408

Feuerstein GZ, 1997, ANN NY ACAD SCI, V825, P179, DOI 10.1111/j.1749-6632.1997.tb48428.x

Ghirnikar RS, 1998, NEUROCHEM RES, V23, P329, DOI 10.1023/A:1022453332560

GIULIAN D, 1989, J NEUROSCI, V9, P4416

GIULIAN D, 1988, J NEUROSCI, V8, P2485

Glabinski AR, 1996, J IMMUNOL, V156, P4363

Gordon MN, 1997, J COMP NEUROL, V388, P106

HAMM RJ, 1991, J NEUROSURG, V75, P916, DOI 10.3171/jns.1991.75.6.0916

Hisahara S, 1997, J NEUROCHEM, V69, P10

HOPKINS SJ, 1995, TRENDS NEUROSCI, V18, P83

HOZUMI I, 1990, BRAIN RES, V534, P291, DOI 10.1016/0006-8993(90)90142-X

Karpus WJ, 1998, J IMMUNOL, V161, P2667

Knoblach SM, 1998, EXP NEUROL, V153, P143, DOI 10.1006/exnr.1998.6877

Kogure K, 1996, ACT NEUR S, V66, P40

Korhonen P, 1997, NEUROSCI LETT, V225, P61, DOI 10.1016/S0304-3940(97)00190-0

Kyrkanides S, 1999, J NEUROIMMUNOL, V95, P95, DOI 10.1016/S0165-5728(98)00270-7

LOSSINSKY AS, 1995, BRAIN PATHOL, V5, P339, DOI 10.1111/j.1750-3639.1995.tb00614.x

LUERSSEN TG, 1988, J NEUROSURG, V68, P409, DOI 10.3171/jns.1988.68.3.0409

MATHEWSON AJ, 1985, BRAIN RES, V327, P61, DOI 10.1016/0006-8993(85)91499-4

MonteroMenei CN, 1996, BRAIN RES, V724, P55, DOI 10.1016/0006-8993(96)00268-5

MORGANTIKOSSMANN MC, 1992, TRENDS PHARMACOL SCI, V13, P286, DOI 10.1016/0165-6147(92)90087-M

Mun-Bryce S, 1998, AM J PHYSIOL-REG I, V274, pR1203, DOI 10.1152/ajpregu.1998.274.5.R1203

NAKAYAMA H, 1994, STROKE, V25, P808, DOI 10.1161/01.STR.25.4.808

NICHOLS NR, 1993, NEUROBIOL AGING, V14, P421, DOI 10.1016/0197-4580(93)90100-P

NORTON WT, 1992, NEUROCHEM RES, V17, P877, DOI 10.1007/BF00993263

Olschowka JA, 1997, BRAIN BEHAV IMMUN, V11, P273, DOI 10.1006/brbi.1997.0506

PENNINGS JL, 1993, ARCH SURG-CHICAGO, V128, P787

PERRY VH, 1993, GLIA, V7, P60, DOI 10.1002/glia.440070111

PODOR TJ, 1992, ANN NY ACAD SCI, V667, P173, DOI 10.1111/j.1749-6632.1992.tb51609.x

Popovich PG, 1997, J COMP NEUROL, V377, P443, DOI 10.1002/(SICI)1096-9861(19970120)377:3<443::AID-CNE10>3.0.CO;2-S

Poynter ME, 1998, J BIOL CHEM, V273, P32833, DOI 10.1074/jbc.273.49.32833

ROTHWELL NJ, 1995, TRENDS NEUROSCI, V18, P130, DOI 10.1016/0166-2236(95)93890-A

Samoilova EB, 1998, CELL IMMUNOL, V188, P118, DOI 10.1006/cimm.1998.1365

Saurwein-Teissl M, 2000, CYTOKINE, V12, P1160, DOI 10.1006/cyto.2000.0679

Shibayama M, 1996, J NEUROTRAUM, V13, P801, DOI 10.1089/neu.1996.13.801

Slepko N, 1996, GLIA, V16, P241

Spencer NFL, 1997, INT IMMUNOL, V9, P1581, DOI 10.1093/intimm/9.10.1581

Spera PA, 1998, NEUROSCI LETT, V251, P189, DOI 10.1016/S0304-3940(98)00537-0

Stahel PF, 1998, BRAIN RES REV, V27, P243, DOI 10.1016/S0165-0173(98)00015-0

Sutherland GR, 1996, STROKE, V27, P1663, DOI 10.1161/01.STR.27.9.1663

Teasdale G, 1979, Acta Neurochir Suppl (Wien), V28, P140

TOPP KS, 1989, GLIA, V2, P201, DOI 10.1002/glia.440020309

VOLLMER DG, 1991, J NEUROSURG, V75, pS37, DOI 10.3171/sup.1991.75.1s.0s37

Yong VW, 1998, TRENDS NEUROSCI, V21, P75

NR 54

TC 99

Z9 119

U1 0

U2 5

PU ELSEVIER

PI AMSTERDAM

PA RADARWEG 29, 1043 NX AMSTERDAM, NETHERLANDS

SN 0165-5728

EI 1872-8421

J9 J NEUROIMMUNOL

JI J. Neuroimmunol.

PD OCT 1

PY 2001

VL 119

IS 2

BP 269

EP 277

DI 10.1016/S0165-5728(01)00404-0

PG 9

WC Immunology; Neurosciences

WE Science Citation Index Expanded (SCI-EXPANDED)

SC Immunology; Neurosciences & Neurology

GA 483RX

UT WOS:000171650600014

PM 11585630

DA 2024-03-03

ER

PT J

AU Orihara, Y

Ikematsu, K

Tsuda, R

Nakasono, I

AF Orihara, Y

Ikematsu, K

Tsuda, R

Nakasono, I

TI Induction of nitric oxide synthase by traumatic brain injury

SO FORENSIC SCIENCE INTERNATIONAL

LA English

DT Article

DE inducible nitric oxide synthase; cerebrovascular smooth muscle cell;

neutrophil; microglia; traumatic brain injury

ID SMOOTH-MUSCLE CELLS; CEREBRAL BLOOD-FLOW; HEAD-INJURY;

HUMAN-NEUTROPHILS; L-ARGININE; RATS; EXPRESSION; IMMATURE

AB We investigated the dynamic induction/expression of inducible nitric oxide synthase (iNOS) using human brains made available through death by traumatic brain injury (TBI). Astrocytes. micro.-lia. and neutrophils were identified in tissue using immunohistochemical staining with antibodies against glial fibrillary acidic protein (GFAP). MHC class II antigen, and neutrophil elastase. respectively. The localization of iNOS protein in each of these cell types was evaluated using immunohistochemistry.

Within 2 days of injury, iNOS immunoreactivity was not detected. However, after 2 days, immunoreactivity was detected in the traumatized brain. The iNOS immunoreactivity was localized on neutrophils and microglia/macrophages in the areas around the tissue necrosis in the traumatized cortical hemisphere, in the deep part of the cortex and the dentate gyri of the hippocampi adjacent to the hemorrhage, and within the cytoplasm of vascular smooth muscle cell of a small artery or arteriole surrounding the injured region. This reactivity was absent after 8 days post-injury,

These observations confirmed the prolonged induction of iNOS within various cells in the injured brain. These responses suggest that iNOS plays a crucial role in cerebrovascular damage and/or secondary brain damage subsequent to traumatic brain injury. Furthermore. the dense nitric oxide (NO) generated by iNOS may play a role in neuronal cell death after injury. (C) 2001 Elsevier Science Ireland Ltd. All rights reserved.

C1 Nagasaki Univ, Sch Med, Dept Legal Med, Nagasaki 8528523, Japan.

C3 Nagasaki University

RP Orihara, Y (corresponding author), Nagasaki Univ, Sch Med, Dept Legal Med, 1-12-4 Sakamoto, Nagasaki 8528523, Japan.

CR Akaike T, 1996, METHOD ENZYMOL, V268, P211

BEASLEY D, 1991, J CLIN INVEST, V87, P602, DOI 10.1172/JCI115036

BOJE KM, 1992, BRAIN RES, V587, P250, DOI 10.1016/0006-8993(92)91004-X

CHAO CC, 1992, J IMMUNOL, V149, P2736

Clark RSB, 1996, PEDIATR RES, V39, P784, DOI 10.1203/00006450-199605000-00007

CLARK RSB, 1994, J NEUROTRAUM, V11, P499, DOI 10.1089/neu.1994.11.499

DAWSON VL, 1994, NEUROPHARMACOLOGY, V33, P1425, DOI 10.1016/0028-3908(94)90045-0

DENIS M, 1994, J LEUKOCYTE BIOL, V55, P682, DOI 10.1002/jlb.55.5.682

DING AH, 1988, J IMMUNOL, V141, P2407

FORSTERMANN U, 1991, BIOCHEM PHARMACOL, V42, P1849, DOI 10.1016/0006-2952(91)90581-O

GIULIAN D, 1989, J NEUROSCI, V9, P4416

GRUNDL PD, 1994, J NEUROTRAUM, V11, P135, DOI 10.1089/neu.1994.11.135

HEWETT SJ, 1994, NEURON, V13, P487, DOI 10.1016/0896-6273(94)90362-X

IADECOLA C, 1995, J CEREBR BLOOD F MET, V15, P378, DOI 10.1038/jcbfm.1995.47

IADECOLA C, 1994, J CEREBR BLOOD F MET, V14, P175, DOI 10.1038/jcbfm.1994.25

KANNO K, 1993, HYPERTENSION, V22, P34, DOI 10.1161/01.HYP.22.1.34

KOBZIK L, 1993, AM J RESP CELL MOL, V9, P371, DOI 10.1165/ajrcmb/9.4.371

Marletta MA, 1998, CURR OPIN CHEM BIOL, V2, P656, DOI 10.1016/S1367-5931(98)80098-7

MCCALL TB, 1991, EUR J IMMUNOL, V21, P2523, DOI 10.1002/eji.1830211032

MONCADA S, 1992, ACTA PHYSIOL SCAND, V145, P201, DOI 10.1111/j.1748-1716.1992.tb09359.x

NATHAN C, 1994, CELL, V78, P915, DOI 10.1016/0092-8674(94)90266-6

OBRIST WD, 1984, J NEUROSURG, V61, P241, DOI 10.3171/jns.1984.61.2.0241

Schoettle R J, 1990, J Neurotrauma, V7, P207, DOI 10.1089/neu.1990.7.207

Stuehr DJ, 1999, BBA-BIOENERGETICS, V1411, P217, DOI 10.1016/S0005-2728(99)00016-X

Stuehr DJ, 1997, ANNU REV PHARMACOL, V37, P339, DOI 10.1146/annurev.pharmtox.37.1.339

THOMAS WE, 1992, BRAIN RES REV, V17, P116

UHL MW, 1994, J NEUROTRAUM, V11, P303, DOI 10.1089/neu.1994.11.303

UZZELL BP, 1986, J NEUROSURG, V65, P630, DOI 10.3171/jns.1986.65.5.0630

VANDERVORT AL, 1994, J IMMUNOL, V152, P4102

WANG XK, 1995, STROKE, V26, P661, DOI 10.1161/01.STR.26.4.661

WRIGHT CD, 1989, BIOCHEM BIOPH RES CO, V160, P813, DOI 10.1016/0006-291X(89)92506-0

YAN L, 1994, J IMMUNOL, V153, P1825

NR 32

TC 62

Z9 67

U1 0

U2 3

PU ELSEVIER SCI IRELAND LTD

PI CLARE

PA CUSTOMER RELATIONS MANAGER, BAY 15, SHANNON INDUSTRIAL ESTATE CO, CLARE,

IRELAND

SN 0379-0738

J9 FORENSIC SCI INT

JI Forensic Sci.Int.

PD DEC 1

PY 2001

VL 123

IS 2-3

BP 142

EP 149

DI 10.1016/S0379-0738(01)00537-0

PG 8

WC Medicine, Legal

WE Science Citation Index Expanded (SCI-EXPANDED)

SC Legal Medicine

GA 504ZW

UT WOS:000172887100009

PM 11728740

DA 2024-03-03

ER

PT J

AU Hutchison, JS

Derrane, RE

Johnston, DL

Gendron, N

Barnes, D

Fliss, H

King, WJ

Rasquinha, I

MacManus, J

Robertson, GS

MacKenzie, AE

AF Hutchison, JS

Derrane, RE

Johnston, DL

Gendron, N

Barnes, D

Fliss, H

King, WJ

Rasquinha, I

MacManus, J

Robertson, GS

MacKenzie, AE

TI Neuronal apoptosis inhibitory protein expression after traumatic brain

injury in the mouse

SO JOURNAL OF NEUROTRAUMA

LA English

DT Article

DE apoptosis; caspase; microglia; neuronal apoptosis inhibitor protein

(NAIP); tumor necrosis factor-alpha (TNF alpha); immunofluorescence;

Western blot

ID NECROSIS-FACTOR-ALPHA; PROGRAMMED CELL-DEATH; SPINAL-CORD INJURY;

CLOSED-HEAD INJURY; DNA FRAGMENTATION; POLY(ADP-RIBOSE) POLYMERASE;

COGNITIVE DEFICITS; RAT-BRAIN; TNF-ALPHA; IN-VIVO

AB Apoptosis of brain cells is triggered by traumatic brain injury (TBI) and is blocked by caspase inhibitors. The neuronal apoptosis inhibitor protein (NAIP), which has been shown to inhibit apoptosis by both caspase-dependant and caspase-independent mechanisms, is neuroprotective in rat models of cerebral ischemia and axotomy. In order to gain a better appreciation of CNS apoptosis following head injury in general and the possible involvement of NAIP specifically, we have configured a mouse model of TBI. In addition to demonstrating apoptosis, the spatiotemporal expression or levels of a number of proteins with apoptosis modulating effects have been determined. Apoptosis of neurons and oligodendrocytes following TBI was observed in brain sections which were triple-stained with in situ end labeling, bisbenzimide and immunofluorescent stain for neuron specific nuclear protein and myelin-associated glycoprotein, respectively. Further evidence for apoptosis following TBI in this model was obtained in brain samples using ligation-mediated PCR amplification of DNA fragments and gel electrophoresis. The temporal profile of apoptosis was similar to the temporal profile of microglial activation determined by CD11b staining and TNF alpha expression induced by TBI. NAIP staining in sections of cerebral cortex and subcortical white matter increased at 6 h and decreased towards control levels at 24 h post-TBI. Temporal changes in the expression of NAIP were also observed using Western blot analysis of brain samples removed from injured cortex and sub-cortical white matter. At the time that NAIP expression decreased markedly (24 h post-TBI), procaspase-3 levels also decreased, PARP cleavage increased, and the highest levels of apoptosis were observed. These findings have implications in our understanding of traumatically induced programmed cell death and may be useful in the configuration of therapies for this common injury state.

C1 Univ Ottawa, Fac Med, Dept Pediat, Div Pediat Intens Care, Ottawa, ON, Canada.

Univ Ottawa, Fac Med, Dept Cellular & Mol Med, Ottawa, ON, Canada.

Childrens Hosp Eastern Ontario, Inst Res, Genet Mol Lab, Ottawa, ON K1H 8L1, Canada.

Univ Ottawa, Fac Med, Dept Pediat, Ottawa, ON, Canada.

Natl Res Council Canada, Inst Biol Sci, Apoptosis Res Grp, Ottawa, ON, Canada.

Univ Ottawa, Fac Med, Dept Biochem, Ottawa, ON, Canada.

C3 University of Ottawa; University of Ottawa; University of Ottawa;

Children's Hospital of Eastern Ontario; University of Ottawa; National

Research Council Canada; University of Ottawa

RP Hutchison, JS (corresponding author), Univ Ottawa, Fac Med, Dept Pediat, Div Pediat Intens Care, Ottawa, ON, Canada.

RI Fliss, Henry/ABG-2561-2020

OI Gendron, Nathalie/0000-0001-5636-7408; Hutchison,

James/0000-0002-5850-6667; Robertson, George/0000-0001-8411-7721

CR ALTHAUS JS, 1993, MOL CHEM NEUROPATHOL, V20, P147, DOI 10.1007/BF02815368

ANSARI B, 1993, J PATHOL, V170, P1, DOI 10.1002/path.1711700102

Beer R, 2000, J CEREBR BLOOD F MET, V20, P669, DOI 10.1097/00004647-200004000-00004

Boldin MP, 1996, CELL, V85, P803, DOI 10.1016/S0092-8674(00)81265-9

Clark D, 1999, IEEE INTERNET COMPUT, V3, P13

Clark RSB, 2000, J NEUROCHEM, V74, P740, DOI 10.1046/j.1471-4159.2000.740740.x

Clark RSB, 1997, J NEUROSCI, V17, P9172

Clark RSB, 2000, J PEDIATR-US, V137, P197, DOI 10.1067/mpd.2000.106903

Conti AC, 1998, J NEUROSCI, V18, P5663

Crowe MJ, 1997, NAT MED, V3, P73, DOI 10.1038/nm0197-73

Diez E, 2000, J IMMUNOL, V164, P1470, DOI 10.4049/jimmunol.164.3.1470

Fox GB, 1998, J NEUROTRAUM, V15, P599, DOI 10.1089/neu.1998.15.599

Gourin CG, 1997, J TRAUMA, V42, P1101, DOI 10.1097/00005373-199706000-00020

GRASLKRAUPP B, 1995, HEPATOLOGY, V21, P1465, DOI 10.1016/0270-9139(95)90071-3

HALL ED, 1985, J NEUROSURG, V62, P882, DOI 10.3171/jns.1985.62.6.0882

HALL ED, 1988, J NEUROSURG, V68, P456, DOI 10.3171/jns.1988.68.3.0456

HILL IE, 1995, BRAIN RES, V676, P398, DOI 10.1016/0006-8993(95)00145-G

Holcik M, 2000, P NATL ACAD SCI USA, V97, P2286, DOI 10.1073/pnas.040469797

Hsu HL, 1996, IMMUNITY, V4, P387, DOI 10.1016/S1074-7613(00)80252-6

Hsu HL, 1996, CELL, V84, P299, DOI 10.1016/S0092-8674(00)80984-8

Knoblach SM, 1999, J NEUROIMMUNOL, V95, P115, DOI 10.1016/S0165-5728(98)00273-2

Knoblach SM, 1998, EXP NEUROL, V153, P143, DOI 10.1006/exnr.1998.6877

Kreider BQ, 1996, J NEUROSCI RES, V44, P459

LaCasse EC, 1998, ONCOGENE, V17, P3247, DOI 10.1038/sj.onc.1202569

LINNIK MD, 1993, STROKE, V24, P2002, DOI 10.1161/01.STR.24.12.2002

Liston P, 1996, NATURE, V379, P349, DOI 10.1038/379349a0

LIU MY, 1995, J FORMOS MED ASSOC, V94, P386

Liu XZ, 1997, J NEUROSCI, V17, P5395

MACMANUS JP, 1995, J CEREBR BLOOD F MET, V15, P728, DOI 10.1038/jcbfm.1995.93

MacManus JP, 1999, J CEREBR BLOOD F MET, V19, P502, DOI 10.1097/00004647-199905000-00004

MACMANUS JP, 1993, NEUROSCI LETT, V164, P89, DOI 10.1016/0304-3940(93)90864-H

MACMANUS JP, 1994, NEUROREPORT, V5, P493, DOI 10.1097/00001756-199401120-00031

Mercer EA, 2000, EMBO J, V19, P3597, DOI 10.1093/emboj/19.14.3597

MULLEN RJ, 1992, DEVELOPMENT, V116, P201

O'Dell DM, 2000, J NEUROSCI, V20, P4821

Oliver FJ, 1998, J BIOL CHEM, V273, P33533, DOI 10.1074/jbc.273.50.33533

Perrelet D, 2000, EUR J NEUROSCI, V12, P2059, DOI 10.1046/j.1460-9568.2000.00098.x

Pravdenkova SV, 1996, BRAIN RES, V729, P151, DOI 10.1016/0006-8993(96)00222-3

Raghupathi R, 1998, J CEREBR BLOOD F MET, V18, P1259, DOI 10.1097/00004647-199811000-00013

Raghupathi R, 2000, J NEUROTRAUM, V17, P927, DOI 10.1089/neu.2000.17.927

RINK A, 1995, AM J PATHOL, V147, P1575

Scherbel U, 1999, P NATL ACAD SCI USA, V96, P8721, DOI 10.1073/pnas.96.15.8721

Shah GM, 1995, ANAL BIOCHEM, V232, P251, DOI 10.1006/abio.1995.0016

Shohami E, 1997, J NEUROIMMUNOL, V72, P169, DOI 10.1016/S0165-5728(96)00181-6

Sinson G, 1997, J NEUROSURG, V86, P511, DOI 10.3171/jns.1997.86.3.0511

Westmoreland SV, 1996, J NEUROVIROL, V2, P118, DOI 10.3109/13550289609146545

Xu DG, 1997, NAT MED, V3, P997, DOI 10.1038/nm0997-997

Yakovlev AG, 1997, J NEUROSCI, V17, P7415

Yaraghi Z, 1998, GENOMICS, V51, P107, DOI 10.1006/geno.1998.5378

Ye P, 1999, ENDOCRINOLOGY, V140, P3063, DOI 10.1210/en.140.7.3063

NR 50

TC 36

Z9 42

U1 0

U2 4

PU MARY ANN LIEBERT INC PUBL

PI LARCHMONT

PA 2 MADISON AVENUE, LARCHMONT, NY 10538 USA

SN 0897-7151

J9 J NEUROTRAUM

JI J. Neurotrauma

PD DEC

PY 2001

VL 18

IS 12

BP 1333

EP 1347

DI 10.1089/08977150152725632

PG 15

WC Critical Care Medicine; Clinical Neurology; Neurosciences

WE Science Citation Index Expanded (SCI-EXPANDED)

SC General & Internal Medicine; Neurosciences & Neurology

GA 504RE

UT WOS:000172869000004

PM 11780864

DA 2024-03-03

ER

PT J

AU Sanz, O

Acarin, L

González, B

Castellano, B

AF Sanz, O

Acarin, L

González, B

Castellano, B

TI NF-κB and IκBα expression following traumatic brain injury to the

immature rat brain

SO JOURNAL OF NEUROSCIENCE RESEARCH

LA English

DT Article

DE astrocyte; microglia; glial response; developing brain; transcription

factor; inflammation

ID THALAMIC GLIAL RESPONSE; INDUCED APOPTOSIS; TRANSCRIPTION FACTOR;

HIPPOCAMPAL-NEURONS; CYTOKINE EXPRESSION; EXCITOTOXIC LESION; YOUNG

BRAIN; ACTIVATION; PROTEINS; SUPPRESSION

AB NF-kappaB is one of the most important modulators of stress and inflammatory gene expression in the nervous system. In the adult brain, NF-kappaB upregulation has been demonstrated in neurons and glial cells in response to experimental injury and neuropathological disorders, where it has been related to both neurodegenerative and neuroprotective activities. Accordingly, the aim of this study was to evaluate the cellular and temporal patterns of NF-kappaB activation and the expression of its endogenous inhibitor IkappaBalpha following traumatic brain injury (TBI) during the early postnatal weeks, when the brain presents elevated levels of plasticity and neuroprotection. Our results showed that cortical trauma to the 9-day-old rat brain induced a very fast upregulation of NF-kappaB, which was maximal within the first 24 hours after injury. NF-kappaB was mainly observed in neuronal cells of the degenerating cortex as well as in astrocytes located in the corpus callosum adjacent to the injury, where a pulse-like pattern of microglial NF-kappaB activation was also found. In addition, astrocytes of the corpus callosum, and microglial cells to a lower extent, also showed de novo expression Of IkappaBalpha within the time of NF-kappaB activation. This study suggests an important role of NF-kappaB activation in the early mechanisms of neuronal death or survival, as well as in the development of the glial and inflammatory responses following traumatic injury to the immature rat brain. (C) 2002 Wiley-Liss, Inc.

C1 Autonomous Univ Barcelona, Dept Cell Biol Physiol & Immunol, Fac Med, Unit Histol, Bellaterra 08193, Spain.

C3 Autonomous University of Barcelona

RP Acarin, L (corresponding author), Autonomous Univ Barcelona, Dept Cell Biol Physiol & Immunol, Fac Med, Unit Histol, Bellaterra 08193, Spain.

EM laia.acarin@uab.es

RI Castellano, Bernardo/G-1428-2010; González, Berta/G-6250-2010; Acarin,

Laia/G-2620-2010; Gonzalez, Berta/G-1428-2010

OI Castellano, Bernardo/0000-0003-1976-971X; Gonzalez,

Berta/0000-0002-1860-3980

CR Acarin L, 1999, NEUROSCIENCE, V89, P549, DOI 10.1016/S0306-4522(98)00331-5

Acarin L, 1999, NEUROSCIENCE, V92, P827, DOI 10.1016/S0306-4522(99)00022-6

Acarin L, 2000, EUR J NEUROSCI, V12, P3505, DOI 10.1046/j.1460-9568.2000.00226.x

Acarin L, 2000, J NEUROPATH EXP NEUR, V59, P151, DOI 10.1093/jnen/59.2.151

Acarin L, 1998, NEUROREPORT, V9, P2869, DOI 10.1097/00001756-199808240-00035

Acarin L, 1997, EXP NEUROL, V147, P410, DOI 10.1006/exnr.1997.6593

Acarin L, 2001, STROKE, V32, P2394, DOI 10.1161/hs1001.097243

Baeuerle PA, 1998, CURR BIOL, V8, pR19, DOI 10.1016/S0960-9822(98)70010-7

Baldwin AS, 1996, ANNU REV IMMUNOL, V14, P649, DOI 10.1146/annurev.immunol.14.1.649

Bales KR, 1998, MOL BRAIN RES, V57, P63, DOI 10.1016/S0169-328X(98)00066-7

Bancroft J., 1996, THEORY PRACTICE HIST, P735

Bethea JR, 1998, J NEUROSCI, V18, P3251

Bonetti B, 1999, AM J PATHOL, V155, P1433, DOI 10.1016/S0002-9440(10)65456-9

Chu ZL, 1997, P NATL ACAD SCI USA, V94, P10057, DOI 10.1073/pnas.94.19.10057

Dalmau I, 1997, J COMP NEUROL, V377, P70

Gabriel C, 1999, MOL BRAIN RES, V65, P61, DOI 10.1016/S0169-328X(98)00330-1

Grilli M, 1999, BIOCHEM PHARMACOL, V57, P1, DOI 10.1016/S0006-2952(98)00214-7

Holmin S, 1997, J NEUROSURG, V86, P493, DOI 10.3171/jns.1997.86.3.0493

Kaltschmidt B, 1999, P NATL ACAD SCI USA, V96, P9409, DOI 10.1073/pnas.96.16.9409

KALTSCHMIDT C, 1994, J NEUROIMMUNOL, V55, P99, DOI 10.1016/0165-5728(94)90151-1

Kolb B, 1996, BEHAV BRAIN RES, V79, P1, DOI 10.1016/0166-4328(95)00254-5

KOLB B, 1994, BRAIN RES, V645, P85, DOI 10.1016/0006-8993(94)91641-1

Lipton SA, 1997, NAT MED, V3, P20, DOI 10.1038/nm0197-20

Mattson MP, 1997, J NEUROSCI RES, V49, P681, DOI 10.1002/(SICI)1097-4547(19970915)49:6<681::AID-JNR3>3.0.CO;2-3

Mattson MP, 1998, INT REV NEUROBIOL, V42, P103, DOI 10.1016/S0074-7742(08)60609-1

Mattson MP, 2000, J NEUROCHEM, V74, P443, DOI 10.1046/j.1471-4159.2000.740443.x

MORGANTIKOSSMAN.MC, 1995, IMMUNE RESPONSES NER, P159

Nakai M, 2000, J NEUROCHEM, V74, P647, DOI 10.1046/j.1471-4159.2000.740647.x

Nonaka M, 1999, J NEUROTRAUM, V16, P1023, DOI 10.1089/neu.1999.16.1023

ONeill LAJ, 1997, TRENDS NEUROSCI, V20, P252, DOI 10.1016/S0166-2236(96)01035-1

Qin ZH, 1998, MOL PHARMACOL, V53, P33, DOI 10.1124/mol.53.1.33

Saccani S, 2001, J EXP MED, V193, P1351, DOI 10.1084/jem.193.12.1351

Sanz O, 2001, GLIA, V36, P259, DOI 10.1002/glia.1114

Simeonidis S, 1999, P NATL ACAD SCI USA, V96, P49, DOI 10.1073/pnas.96.1.49

Stoll G, 1998, PROG NEUROBIOL, V56, P149, DOI 10.1016/S0301-0082(98)00034-3

Warr WB, 1981, NEUROANATOMICAL TRAC, P207

YANG KY, 1995, NEUROSCI LETT, V197, P101, DOI 10.1016/0304-3940(95)11919-N

Yu ZF, 1999, J NEUROSCI, V19, P8856, DOI 10.1523/JNEUROSCI.19-20-08856.1999

NR 38

TC 48

Z9 54

U1 1

U2 4

PU WILEY

PI HOBOKEN

PA 111 RIVER ST, HOBOKEN 07030-5774, NJ USA

SN 0360-4012

EI 1097-4547

J9 J NEUROSCI RES

JI J. Neurosci. Res.

PD MAR 15

PY 2002

VL 67

IS 6

BP 772

EP 780

DI 10.1002/jnr.10140

PG 9

WC Neurosciences

WE Science Citation Index Expanded (SCI-EXPANDED)

SC Neurosciences & Neurology

GA 526ZC

UT WOS:000174158700009

PM 11891791

OA Bronze

DA 2024-03-03

ER

PT J

AU Beschorner, R

Nguyen, TD

Gözalan, F

Pedal, I

Mattern, R

Schluesener, HJ

Meyermann, R

Schwab, JM

AF Beschorner, R

Nguyen, TD

Gözalan, F

Pedal, I

Mattern, R

Schluesener, HJ

Meyermann, R

Schwab, JM

TI CD14 expression by activated parenchymal microglia/macrophages and

infiltrating monocytes following human traumatic brain injury

SO ACTA NEUROPATHOLOGICA

LA English

DT Article

DE traumatic brain injury; inflammation; immune response; CD14

ID TUMOR-NECROSIS-FACTOR; MICROGLIAL CELLS; DEPENDENT MECHANISMS;

MONONUCLEAR-CELLS; NEGATIVE BACTERIA; GENE-EXPRESSION; UP-REGULATION;

SOLUBLE CD14; EX-VIVO; LIPOPOLYSACCHARIDE

AB The immune response in the central nervous system (CNS) is under tight control of regulatory mechanisms, resulting in the establishment of immune privilege. CNS injury induces an acute inflammatory reaction, composed mainly of invading leukocytes and activated microglial cells/macrophages. The generation of this robust immune response requires binding of receptors such as CD14, a pattern recognition receptor of the immune system. CD14, a surface molecule of monocytic cells, is up-regulated after monocyte stimulation and is involved in cellular activation. To examine CD14 expression in human brain lesions we investigated sections of brains obtained at autopsy from 25 cases following closed traumatic brain injury (TBI) and 5 control brains by immunohistochemistry. Detection of CD14 in controls demonstrated constitutive expression by perivascular cells, but not in parenchymal microglial cells, equivalent to known expression pattern of ED2 in rats. Following TBI, numbers of CD14(+) cells in perivascular spaces and in the brain parenchyma increased in parallel within 1-2 days, both at the lesion and in adjacent perilesional areas. The number of CD14(+) cells in perivascular spaces and in the brain parenchyma reached maximum levels within 4-8 days and remained elevated until weeks after trauma. In contrast to activated parenchymal microglia/macrophages, resting parenchymal microglial cells lacked CD14. Thus, early CD14 expression constitutes an essential part of the acute inflammatory CNS response following trauma.

C1 Heidelberg Univ, Inst Legal Med, Heidelberg, Germany.

Univ Tubingen, Brain Res Inst, Sch Med, D-72076 Tubingen, Germany.

C3 Ruprecht Karls University Heidelberg; Eberhard Karls University of

Tubingen

RP Beschorner, R (corresponding author), Univ Tubingen, Brain Res Inst, Sch Med, Calwerstr 3, D-72076 Tubingen, Germany.

EM rudi.beschorner@med.uni-tuebingen.de

RI Beschorner, Rudi/M-6397-2014

OI Schwab, Jan/0000-0001-6784-4919; Beschorner, Rudi/0000-0003-1109-915X

CR Abe M, 1999, J NEUROL, V246, P358, DOI 10.1007/s004150050363

Aderem A, 1999, ANNU REV IMMUNOL, V17, P593, DOI 10.1146/annurev.immunol.17.1.593

Antal-Szalmás P, 2000, CYTOMETRY, V41, P279, DOI 10.1002/1097-0320(20001201)41:4<279::AID-CYTO6>3.0.CO;2-B

ARDITI M, 1995, J IMMUNOL, V155, P3994

Asea A, 2000, NAT MED, V6, P435, DOI 10.1038/74697

Bauer J, 1996, HISTOCHEM J, V28, P83, DOI 10.1007/BF02331413

Becher B, 1996, GLIA, V18, P1

Becher B, 1996, J NEUROSCI RES, V45, P375

Beschorner R, 2000, ACTA NEUROPATHOL, V100, P627, DOI 10.1007/s004010000232

Beschorner R, 2000, ACTA NEUROPATHOL, V100, P377, DOI 10.1007/s004010000202

Beyer M, 2000, GLIA, V31, P262, DOI 10.1002/1098-1136(200009)31:3<262::AID-GLIA70>3.0.CO;2-2

Bosco MC, 1997, J IMMUNOL, V159, P2922

Cauwels A, 1999, J IMMUNOL, V162, P4762

Chen KC, 1999, WIREL NETW, V5, P1, DOI 10.1023/A:1019157818476

DENTENER MA, 1993, J IMMUNOL, V150, P2885

Devitt A, 1998, NATURE, V392, P505, DOI 10.1038/33169

Dick AD, 1997, AIDS, V11, P1699, DOI 10.1097/00002030-199714000-00006

DICKSON DW, 1991, LAB INVEST, V64, P135

Dufour A, 1996, J NEUROL, V243, P666, DOI 10.1007/BF00878667

Fearns C, 1998, SHOCK, V9, P157, DOI 10.1097/00024382-199803000-00001

Frevert CW, 2000, J IMMUNOL, V164, P5439, DOI 10.4049/jimmunol.164.10.5439

Galea E, 1996, J NEUROIMMUNOL, V64, P19, DOI 10.1016/0165-5728(95)00143-3

GRAEBER MB, 1990, TRENDS NEUROSCI, V13, P366, DOI 10.1016/0166-2236(90)90020-B

Graham DI, 1997, GREENFIELDS NEUROPAT, P197

Guha M, 2001, CELL SIGNAL, V13, P85, DOI 10.1016/S0898-6568(00)00149-2

Guillemin G, 1997, J NEUROSCI RES, V49, P576, DOI 10.1002/(SICI)1097-4547(19970901)49:5<576::AID-JNR8>3.3.CO;2-O

Han JH, 1998, PROG CLIN BIOL RES, V397, P157

HICKEY WF, 1987, P NATL ACAD SCI USA, V84, P2082, DOI 10.1073/pnas.84.7.2082

HICKEY WF, 1988, SCIENCE, V239, P290, DOI 10.1126/science.3276004

Jersmann HPA, 2001, INFECT IMMUN, V69, P479, DOI 10.1128/IAI.69.1.479-485.2001

JORDAN FL, 1988, BRAIN RES REV, V13, P165, DOI 10.1016/0165-0173(88)90019-7

Kane JP, 1999, CIRCULATION, V99, P3210, DOI 10.1161/01.CIR.99.25.3210

Kennedy DW, 1997, BLOOD, V90, P986, DOI 10.1182/blood.V90.3.986.986_986_993

Kennedy DW, 1998, P NATL ACAD SCI USA, V95, P14944, DOI 10.1073/pnas.95.25.14944

Kirschning CJ, 1998, J EXP MED, V188, P2091, DOI 10.1084/jem.188.11.2091

Kol A, 2000, J IMMUNOL, V164, P13, DOI 10.4049/jimmunol.164.1.13

Kreutzberg GW, 1996, TRENDS NEUROSCI, V19, P312, DOI 10.1016/0166-2236(96)10049-7

KRUGER C, 1991, CLIN EXP IMMUNOL, V85, P297

Kurt-Jones EA, 2000, NAT IMMUNOL, V1, P398, DOI 10.1038/80833

KUSUNOKI T, 1995, J EXP MED, V182, P1673, DOI 10.1084/jem.182.6.1673

LASSMANN H, 1991, J NEUROSCI RES, V28, P236, DOI 10.1002/jnr.490280211

LASSMANN H, 1993, GLIA, V7, P19, DOI 10.1002/glia.440070106

LING EA, 1993, GLIA, V7, P9, DOI 10.1002/glia.440070105

LOPPNOW H, 1995, INFECT IMMUN, V63, P1020, DOI 10.1128/IAI.63.3.1020-1026.1995

Lucas R, 1997, J LEUKOCYTE BIOL, V61, P551, DOI 10.1002/jlb.61.5.551

Manigold T, 2000, CYTOKINE, V12, P1788, DOI 10.1006/cyto.2000.0783

MATO M, 1986, ACTA NEUROPATHOL, V72, P117, DOI 10.1007/BF00685972

MCGEER PL, 1993, GLIA, V7, P84, DOI 10.1002/glia.440070114

Meng FY, 1997, J EXP MED, V185, P1661, DOI 10.1084/jem.185.9.1661

Mittelbronn M, 2001, ACTA NEUROPATHOL, V101, P249

Nadeau S, 2000, J NEUROSCI, V20, P3456, DOI 10.1523/JNEUROSCI.20-09-03456.2000

Nockher WA, 1999, J NEUROIMMUNOL, V101, P161, DOI 10.1016/S0165-5728(99)00141-1

PERRY VH, 1993, TRENDS NEUROSCI, V16, P268, DOI 10.1016/0166-2236(93)90180-T

PEUDENIER S, 1991, ANN NEUROL, V29, P152, DOI 10.1002/ana.410290207

Postler E, 2000, J NEUROIMMUNOL, V108, P244, DOI 10.1016/S0165-5728(00)00283-6

Prinz M, 1999, J NEUROPATH EXP NEUR, V58, P1078, DOI 10.1097/00005072-199910000-00006

PUGIN J, 1994, IMMUNITY, V1, P509, DOI 10.1016/1074-7613(94)90093-0

Saito S, 2000, EUR J BIOCHEM, V267, P37, DOI 10.1046/j.1432-1327.2000.00956.x

Schimke J, 1998, P NATL ACAD SCI USA, V95, P13875, DOI 10.1073/pnas.95.23.13875

Schletter J, 1995, ARCH MICROBIOL, V164, P383, DOI 10.1007/BF02529735

Schluesener HJ, 1998, ACTA NEUROPATHOL, V96, P575, DOI 10.1007/s004010050938

Schluesener HJ, 1997, GLIA, V20, P365, DOI 10.1002/(SICI)1098-1136(199708)20:4<365::AID-GLIA8>3.0.CO;2-4

Selvi E, 2000, ANN RHEUM DIS, V59, P399, DOI 10.1136/ard.59.5.399

STREIT WJ, 1987, J NEUROCYTOL, V16, P249, DOI 10.1007/BF01795308

Streit Wolfgang J., 1995, P85

Su GL, 1999, J HEPATOL, V31, P435, DOI 10.1016/S0168-8278(99)80034-8

Su GL, 1995, CRIT REV IMMUNOL, V15, P201, DOI 10.1615/CritRevImmunol.v15.i3-4.10

Takeshita S, 1999, J INFECT DIS, V179, P508, DOI 10.1086/314600

THEELE DP, 1993, GLIA, V7, P5, DOI 10.1002/glia.440070104

Ulevitch RJ, 1999, NAT MED, V5, P144, DOI 10.1038/5504

ULEVITCH RJ, 1995, ANNU REV IMMUNOL, V13, P437, DOI 10.1146/annurev.iy.13.040195.002253

Ulevitch RJ, 1999, CURR OPIN IMMUNOL, V11, P19, DOI 10.1016/S0952-7915(99)80004-1

ULVESTAD E, 1994, J NEUROPATH EXP NEUR, V53, P492, DOI 10.1097/00005072-199409000-00008

VASS K, 1986, ACTA NEUROPATHOL, V70, P149, DOI 10.1007/BF00691433

Verbon A, 2001, J IMMUNOL, V166, P3599, DOI 10.4049/jimmunol.166.5.3599

Wang PY, 1999, J BIOL CHEM, V274, P23235, DOI 10.1074/jbc.274.33.23235

Wang PY, 1998, J BIOL CHEM, V273, P24309, DOI 10.1074/jbc.273.38.24309

WILLIAMS K, 1992, J NEUROPATH EXP NEUR, V51, P538, DOI 10.1097/00005072-199209000-00009

WRIGHT SD, 1990, SCIENCE, V249, P1431, DOI 10.1126/science.1698311

ZIEGLERHEITBROCK HWL, 1994, EUR J IMMUNOL, V24, P1937, DOI 10.1002/eji.1830240835

NR 80

TC 105

Z9 117

U1 0

U2 13

PU SPRINGER

PI NEW YORK

PA ONE NEW YORK PLAZA, SUITE 4600, NEW YORK, NY, UNITED STATES

SN 0001-6322

EI 1432-0533

J9 ACTA NEUROPATHOL

JI Acta Neuropathol.

PD JUN

PY 2002

VL 103

IS 6

BP 541

EP 549

DI 10.1007/s00401-001-0503-7

PG 9

WC Clinical Neurology; Neurosciences; Pathology

WE Science Citation Index Expanded (SCI-EXPANDED)

SC Neurosciences & Neurology; Pathology

GA 557EE

UT WOS:000175894500002

PM 12012085

OA Bronze

DA 2024-03-03

ER

PT J

AU Basu, A

Krady, JK

O'Malley, M

Styren, SD

DeKosky, ST

Levison, SW

AF Basu, A

Krady, JK

O'Malley, M

Styren, SD

DeKosky, ST

Levison, SW

TI The type 1 interleukin-1 receptor is essential for the efficient

activation of microglia and the induction of multiple proinflammatory

mediators in response to brain injury

SO JOURNAL OF NEUROSCIENCE

LA English

DT Article

DE cytokines; IL-1; IL-6; TNF-alpha; traumatic brain injury;

prostaglandins; astrocytes; null mutant mice

ID NECROSIS-FACTOR-ALPHA; FACTOR MESSENGER-RNA; EXCITOTOXIC NEURONAL

DAMAGE; NF-KAPPA-B; GROWTH-FACTOR; TRANSGENIC MICE; RAT-BRAIN;

ALZHEIMERS-DISEASE; INFLAMMATORY RESPONSE; CONVERTING-ENZYME

AB Interleukin-1 (IL-1) is induced immediately after insults to the brain, and elevated levels of IL-1 have been strongly implicated in the neurodegeneration that accompanies stroke, Alzheimer's disease, and multiple sclerosis. In animal models, antagonizing IL-1 has been shown to reduce cell death; however, the basis for this protection has not been elucidated. Here we analyzed the response to penetrating brain injury in mice lacking the type 1 IL-1 receptor (IL-1R1) to determine which cellular and molecular mediators of tissue damage require IL-1 signaling. At the cellular level, fewer amoeboid microglia/macrophages appeared adjacent to the injured brain tissue in IL-1R1 null mice, and those microglia present at early postinjury intervals retained their resting morphology. Astrogliosis also was mildly abrogated. At the molecular level, cyclooxygenase-2 (Cox-2) and IL-6 expression were depressed and delayed. Interestingly, basal levels of Cox-2, IL-1, and IL-6 were significantly lower in the IL-1R1 null mice. In addition, stimulation of vascular cell adhesion molecule-1 mRNA was depressed in the IL-1R1 null mice, and correspondingly, there was reduced diapedesis of peripheral macrophages in the IL-1R1 null brain after injury. This observation correlated with a reduced number of Cox-2(+) amoeboid phagocytes adjacent to the injury. In contrast, several molecular aspects of the injury response were normal, including expression of tumor necrosis factor-alpha and the production of nerve growth factor. Because antagonizing IL-1 protects neural cells in experimental models of stroke and multiple sclerosis, our data suggest that cell preservation is achieved by abrogating microglial/macrophage activation and the subsequent self-propagating cycle of inflammation.

C1 Penn State Univ, Dept Anat & Neurosci, Coll Med, Hershey, PA 17033 USA.

Univ Pittsburgh, Dept Neurol, Pittsburgh, PA 15213 USA.

C3 Pennsylvania Commonwealth System of Higher Education (PCSHE);

Pennsylvania State University; Penn State Health; Pennsylvania

Commonwealth System of Higher Education (PCSHE); University of

Pittsburgh

RP Levison, SW (corresponding author), Penn State Univ, Dept Anat & Neurosci, Coll Med, H109,POB 850, Hershey, PA 17033 USA.

RI BASU, ANIRBAN/C-1166-2009; Levison, Steven W./Q-6903-2019

OI Levison, Steven W./0000-0002-1264-7309; DeKosky,

Steven/0000-0003-3743-2758; Basu, Anirban/0000-0002-5200-2054

CR ACARIN L, 1994, J HISTOCHEM CYTOCHEM, V42, P1033, DOI 10.1177/42.8.8027523

Akiyama H, 2000, NEUROBIOL AGING, V21, P383, DOI 10.1016/S0197-4580(00)00124-X

Albrecht PJ, 2002, EXP NEUROL, V173, P46, DOI 10.1006/exnr.2001.7834

ALOISI F, 1992, J IMMUNOL, V149, P2358

ARAUJO DM, 1992, J NEUROSCI, V12, P1668

BANDTLOW CE, 1990, J CELL BIOL, V111, P1701, DOI 10.1083/jcb.111.4.1701

BASU A, 2002, IN PRESS GLIA

BASU A, 2001, J NEUROIMMUNOL, V118, P94

BOELENS JJ, 2000, INFECT IMMUN, V68, P6929

Bonventre JV, 1997, NATURE, V390, P622, DOI 10.1038/37635

Boutin H, 2001, J NEUROSCI, V21, P5528, DOI 10.1523/JNEUROSCI.21-15-05528.2001

CAMPBELL IL, 1993, P NATL ACAD SCI USA, V90, P10061, DOI 10.1073/pnas.90.21.10061

CHOPP M, 1994, STROKE, V25, P869, DOI 10.1161/01.STR.25.4.869

CHUNG IY, 1990, J IMMUNOL, V144, P2999

DACUNHA A, 1992, J NEUROIMMUNOL, V36, P157

DECKERTSCHLUTER M, 1992, J NEUROL SCI, V113, P50, DOI 10.1016/0022-510X(92)90264-L

DEKOSKY ST, 1994, EXP NEUROL, V130, P173, DOI 10.1006/exnr.1994.1196

Friedlander RM, 1997, J EXP MED, V185, P933, DOI 10.1084/jem.185.5.933

Friedman WJ, 1996, J BIOL CHEM, V271, P31115, DOI 10.1074/jbc.271.49.31115

FRIEDMAN WJ, 1990, J NEUROSCI RES, V27, P374, DOI 10.1002/jnr.490270316

GADIENT RA, 1990, NEUROSCI LETT, V117, P335, DOI 10.1016/0304-3940(90)90687-5

GIULIAN D, 1988, J NEUROSCI, V8, P2485

Glaccum MB, 1997, J IMMUNOL, V159, P3364

Griffin WST, 2000, EXP GERONTOL, V35, P481, DOI 10.1016/S0531-5565(00)00110-8

GRIFFIN WST, 1989, P NATL ACAD SCI USA, V86, P7611

HAHN M, 1994, GLIA, V10, P286, DOI 10.1002/glia.440100407

Hallenbeck JM, 1996, ACT NEUR S, V66, P27

Hara H, 1997, P NATL ACAD SCI USA, V94, P2007, DOI 10.1073/pnas.94.5.2007

Heyser CJ, 1997, P NATL ACAD SCI USA, V94, P1500, DOI 10.1073/pnas.94.4.1500

HOFMAN FM, 1986, J IMMUNOL, V136, P3239

JACOBS CA, 1991, J IMMUNOL, V146, P2983

Kelley KA, 1999, AM J PATHOL, V155, P995, DOI 10.1016/S0002-9440(10)65199-1

Klein MA, 1997, GLIA, V19, P227, DOI 10.1002/(SICI)1098-1136(199703)19:3<227::AID-GLIA5>3.0.CO;2-W

Legos JJ, 2000, NEUROSCI LETT, V282, P189, DOI 10.1016/S0304-3940(00)00907-1

Levison SW, 1996, EXP NEUROL, V141, P256, DOI 10.1006/exnr.1996.0160

LING EA, 1980, J COMP NEUROL, V193, P631, DOI 10.1002/cne.901930304

Loddick SA, 1996, J CEREBR BLOOD F MET, V16, P932, DOI 10.1097/00004647-199609000-00017

Luheshi GN, 1999, P NATL ACAD SCI USA, V96, P7047, DOI 10.1073/pnas.96.12.7047

McGuinness MC, 1997, J NEUROIMMUNOL, V75, P174, DOI 10.1016/S0165-5728(97)00020-9

MINAMI M, 1992, J NEUROCHEM, V58, P390, DOI 10.1111/j.1471-4159.1992.tb09324.x

Minghetti L, 1999, J NEUROPATH EXP NEUR, V58, P1184, DOI 10.1097/00005072-199911000-00008

NORRIS JG, 1994, J IMMUNOL, V152, P841

Pasinetti GM, 1998, NEUROSCIENCE, V87, P319, DOI 10.1016/S0306-4522(98)00218-8

Penkowa M, 1999, GLIA, V25, P343

RELTON JK, 1992, BRAIN RES BULL, V29, P243, DOI 10.1016/0361-9230(92)90033-T

Rothwell NJ, 2000, TRENDS NEUROSCI, V23, P618, DOI 10.1016/S0166-2236(00)01661-1

Schielke GP, 1998, J CEREBR BLOOD F MET, V18, P180, DOI 10.1097/00004647-199802000-00009

SHENG JG, 1995, NEUROPATH APPL NEURO, V21, P290, DOI 10.1111/j.1365-2990.1995.tb01063.x

Silverstein Arthur M, 1989, HIST IMMUNOLOGY

SPARACIO SM, 1992, J NEUROIMMUNOL, V39, P231, DOI 10.1016/0165-5728(92)90257-L

Streit WJ, 2000, J NEUROSCI RES, V61, P10, DOI 10.1002/1097-4547(20000701)61:1<10::AID-JNR2>3.0.CO;2-E

Stroemer RP, 1998, J CEREBR BLOOD F MET, V18, P833, DOI 10.1097/00004647-199808000-00003

VIGE X, 1991, MOL PHARMACOL, V40, P186

YAMASAKI Y, 1995, STROKE, V26, P676, DOI 10.1161/01.STR.26.4.676

NR 54

TC 136

Z9 158

U1 0

U2 11

PU SOC NEUROSCIENCE

PI WASHINGTON

PA 11 DUPONT CIRCLE, NW, STE 500, WASHINGTON, DC 20036 USA

SN 0270-6474

J9 J NEUROSCI

JI J. Neurosci.

PD JUL 15

PY 2002

VL 22

IS 14

BP 6071

EP 6082

PG 12

WC Neurosciences

WE Science Citation Index Expanded (SCI-EXPANDED)

SC Neurosciences & Neurology

GA 573PR

UT WOS:000176840500031

PM 12122068

DA 2024-03-03

ER

PT J

AU Liu, PH

Wang, YJ

Tseng, GF

AF Liu, PH

Wang, YJ

Tseng, GF

TI Close axonal injury of rubrospinal neurons induced transient

perineuronal astrocytic and microglial reaction that coincided with

their massive degeneration

SO EXPERIMENTAL NEUROLOGY

LA English

DT Article

DE axotomy; trauma; spinal cord injury; brain stem; red nucleus; CNS

ID RAT CORTICOSPINAL NEURONS; NEUROTROPHIC FACTORS; SPINAL MOTONEURONS;

DISTAL AXOTOMY; RED NUCLEUS; ADULT-RAT; CORD; REGENERATION; TERMINALS;

NERVE

AB To learn more about the pathophysiology of axonal injury and the significance of axon collaterals on the survival of axotomized cord-projection central neurons, we studied the survival rate, surrounding astrocytic and microglial reactions, and bouton coverage on rat rubrospinal cell bodies following their axonal lesion at the brain stem and upper cervical level. T e brain stem lesion disconnected most rubrospinal neurons from all their targets, while the upper cervical lesion spared their supraspinal collaterals. Much higher cell loss accompanied by robust astrocytic and microglial reaction was found following brain stem than upper cervical lesion starting 4 days postaxotomy. The reaction of astrocytes had subsided while microglial reaction remained relatively robust by 10 weeks postaxotomy when the cell loss had slowed down. Ultrastructural observation revealed that reactive astrocytes covered 40%, an increase from the 20% of control, of brain stem-axotomized rubrospinal cell body surface at 4 days and 2 weeks and returned to normal levels by 10 weeks postlesion. An increase of apposition by axons and dendrites and a moderate decrease of round and flattened vesicle-containing bouton contacts at 4 days and 2 weeks and returning to normal levels at 10 weeks postaxotomy accompanied this. It appears that although axotomy induced robust astrocytic reaction around cord-projection central neurons, this, unlike their periphery-projection counterparts, failed to effectively strip their somatic synapses. In effect, this might in part determine neuronal fate following axonal injury. (C) 2002 Elsevier Science (USA).

C1 Natl Taiwan Univ, Coll Med, Dept Anat & Cell Biol, Taipei, Taiwan.

Tzuchi Univ, Coll Med, Dept Anat, Hualien, Taiwan.

C3 National Taiwan University; Tzu Chi University

RP Liu, PH (corresponding author), Natl Taiwan Univ, Coll Med, Dept Anat & Cell Biol, Taipei, Taiwan.

OI Liu, Pei-Hsin/0000-0002-1398-7006

CR BENVENISTE EN, 1993, ASTROCYTES PHARM FUN, P355

BLINZING.K, 1968, Z ZELLFORSCH MIK ANA, V85, P145, DOI 10.1007/BF00325030

Cajal RS., 1928, DEGENERATION REGENER

Chen JR, 1997, NEUROSCIENCE, V79, P449, DOI 10.1016/S0306-4522(96)00704-X

Cross AK, 2001, MICROSC RES TECHNIQ, V54, P10, DOI 10.1002/jemt.1115

DIENER PS, 1994, NEUROREPORT, V5, P1913, DOI 10.1097/00001756-199410000-00018

EGAN DA, 1977, ACTA NEUROPATHOL, V37, P13, DOI 10.1007/BF00684534

Fernandes KJL, 1999, J COMP NEUROL, V414, P495, DOI 10.1002/(SICI)1096-9861(19991129)414:4<495::AID-CNE6>3.0.CO;2-S

Fu YS, 1996, EXP NEUROL, V137, P142, DOI 10.1006/exnr.1996.0014

Giehl KM, 1996, EUR J NEUROSCI, V8, P1167, DOI 10.1111/j.1460-9568.1996.tb01284.x

GIULIAN D, 1993, GLIA, V7, P102, DOI 10.1002/glia.440070116

GRAEBER MB, 1988, J NEUROCYTOL, V17, P209, DOI 10.1007/BF01674208

HALL GF, 1983, SCIENCE, V222, P518, DOI 10.1126/science.6623092

Kobayashi H, 1997, BRAIN RES BULL, V43, P17, DOI 10.1016/S0361-9230(96)00343-7

Lindå H, 2000, J COMP NEUROL, V425, P10

LING EA, 1993, GLIA, V7, P9, DOI 10.1002/glia.440070105

Liu CL, 2002, ANAT EMBRYOL, V205, P245, DOI 10.1007/s00429-002-0250-0

MARTY S, 1994, NEUROSCIENCE, V62, P1121, DOI 10.1016/0306-4522(94)90348-4

MARTY S, 1994, J NEUROSCI, V14, P5257

NJA A, 1978, J PHYSIOL-LONDON, V277, P55

Novikova LN, 2000, EUR J NEUROSCI, V12, P776, DOI 10.1046/j.1460-9568.2000.00978.x

PEARSON HE, 1992, J COMP NEUROL, V315, P333, DOI 10.1002/cne.903150308

PRENDERGAST J, 1976, J COMP NEUROL, V166, P163, DOI 10.1002/cne.901660204

RANSOHOFF RM, 1996, CYTOKINES CNS, P309

REID JM, 1975, J COMP NEUROL, V162, P363, DOI 10.1002/cne.901620306

RICHARDSON PM, 1984, J NEUROCYTOL, V13, P165, DOI 10.1007/BF01148324

ROSSI F, 1993, NEUROSCIENCE, V53, P759, DOI 10.1016/0306-4522(93)90622-M

Rossi F, 1997, PROG BRAIN RES, V114, P283

Streit WJ, 1999, PROG NEUROBIOL, V57, P563, DOI 10.1016/S0301-0082(98)00069-0

SVENSSON M, 1994, J ANAT, V185, P537

Tseng GF, 1996, J NEUROPHYSIOL, V75, P248, DOI 10.1152/jn.1996.75.1.248

Tseng GF, 1996, BRAIN RES, V715, P32, DOI 10.1016/0006-8993(95)01418-7

TSENG GF, 1995, ANAT EMBRYOL, V191, P243

Tseng GF, 1996, BRAIN RES, V742, P115, DOI 10.1016/S0006-8993(96)00972-9

Tseng GF, 1996, ANAT EMBRYOL, V194, P457

TSENG GF, 1993, J COMP NEUROL, V335, P92, DOI 10.1002/cne.903350107

VILLEGASPEREZ MP, 1993, J NEUROBIOL, V24, P23, DOI 10.1002/neu.480240103

Wang YJ, 2000, J NEUROTRAUM, V17, P231, DOI 10.1089/neu.2000.17.231

Wang YJ, 1996, NEUROSCIENCE, V74, P427, DOI 10.1016/0306-4522(96)00154-6

WANG YJ, IN PRESS J NEUROTRAU

Ye JH, 1997, EXP NEUROL, V143, P70, DOI 10.1006/exnr.1996.6353

NR 41

TC 13

Z9 17

U1 0

U2 1

PU ACADEMIC PRESS INC ELSEVIER SCIENCE

PI SAN DIEGO

PA 525 B ST, STE 1900, SAN DIEGO, CA 92101-4495 USA

SN 0014-4886

J9 EXP NEUROL

JI Exp. Neurol.

PD JAN

PY 2003

VL 179

IS 1

BP 111

EP 126

DI 10.1006/exnr.2002.8057

PG 16

WC Neurosciences

WE Science Citation Index Expanded (SCI-EXPANDED)

SC Neurosciences & Neurology

GA 634RU

UT WOS:000180355100013

PM 12504873

DA 2024-03-03

ER

PT J

AU Mueller, CA

Schluesener, HJ

Conrad, S

Meyermann, R

Schwab, JM

AF Mueller, CA

Schluesener, HJ

Conrad, S

Meyermann, R

Schwab, JM

TI Lesional expression of a proinflammatory and antiangiogenic cytokine

EMAP II confined to endothelium and microglia/macrophages during

secondary damage following experimental traumatic brain injury

SO JOURNAL OF NEUROIMMUNOLOGY

LA English

DT Article

DE EMAP II; secondary brain damage; traumatic brain injury; inflammation;

antiangiogenic

ID ACTIVATING POLYPEPTIDE-II; TUMOR-NECROSIS-FACTOR; SPINAL-CORD;

INFLAMMATORY RESPONSE; VASCULAR ARCHITECTURE; MICROGLIAL CELLS; CORTICAL

IMPACT; MACROPHAGES; RAT; ENCEPHALOMYELITIS

AB We analyzed expression of Endothelial Monocyte-Activating Polypeptide II (EMAP II), a proinflammatory, antiangiogenic cytokine in rat brains after stab wound injury and observed a highly significant (p<0.0001) lesional accumulation confined to microglia/macrophages. Maximum numbers were seen at day 5 declining until 21 days after injury. Further, EMAP II+ microglia/macrophages formed clusters in perivascular Virchow-Robin spaces. Prolonged accumulation of EMAP II+, EDI+ microglia/macrophages and increased lesional numbers of EMAP II+ endothelial/smooth muscle cells during the acute postinjury period might indicate that EMAP II enrich the proinflammatory and antiangiogenic repertoire of effector molecules expressed by activated microglia/macrophages during secondary damage. (C) 2002 Elsevier Science B.V. All rights reserved.

C1 Univ Tubingen, Sch Med, Inst Brain Res, D-72076 Tubingen, Germany.

C3 Eberhard Karls University of Tubingen

RP Mueller, CA (corresponding author), Univ Tubingen, Sch Med, Inst Brain Res, Calwerstr 2, D-72076 Tubingen, Germany.

EM Christian-Andreas.Mueller@med.uni-tuebingen.de

OI Schwab, Jan/0000-0001-6784-4919

CR Adelson PD, 1998, ACT NEUR S, V71, P104

AIHARA N, 1995, J NEUROTRAUM, V12, P53, DOI 10.1089/neu.1995.12.53

BAETHMANN A, 1988, CRIT CARE MED, V16, P972, DOI 10.1097/00003246-198810000-00008

BANATI RB, 1993, GLIA, V7, P111, DOI 10.1002/glia.440070117

Bartholdi D, 1997, EUR J NEUROSCI, V9, P2549, DOI 10.1111/j.1460-9568.1997.tb01684.x

Berger AC, 2000, MICROVASC RES, V60, P70, DOI 10.1006/mvre.2000.2249

CARLOS TM, 1994, BLOOD, V84, P2068

Chang SY, 2002, J BIOL CHEM, V277, P8388, DOI 10.1074/jbc.M108792200

CHAO CC, 1992, J IMMUNOL, V149, P2736

COFFEY PJ, 1990, NEUROSCIENCE, V35, P121, DOI 10.1016/0306-4522(90)90126-O

COLTON CA, 1987, FEBS LETT, V223, P284, DOI 10.1016/0014-5793(87)80305-8

DAVID S, 1990, NEURON, V5, P463, DOI 10.1016/0896-6273(90)90085-T

DUSART I, 1994, EUR J NEUROSCI, V6, P712, DOI 10.1111/j.1460-9568.1994.tb00983.x

ESIRI MM, 1990, J NEUROL SCI, V100, P3, DOI 10.1016/0022-510X(90)90004-7

Fujita T, 1998, ACTA NEUROCHIR, V140, P275, DOI 10.1007/s007010050095

Gerlach C, 1997, LAB INVEST, V77, P697

GIULIAN D, 1989, J NEUROSCI, V9, P4416

GIULIAN D, 1993, GLIA, V7, P102, DOI 10.1002/glia.440070116

GOODMAN JC, 1990, J NEUROIMMUNOL, V30, P213

Holmin S, 2000, J NEUROSURG, V92, P108, DOI 10.3171/jns.2000.92.1.0108

HOLMIN S, 1995, NEUROSCI LETT, V194, P97, DOI 10.1016/0304-3940(95)11737-H

HOLMIN S, 1995, ACTA NEUROCHIR, V132, P110, DOI 10.1007/BF01404857

Hsu CY, 1996, NEUROTRAUMA, P1433

HUITINGA I, 1990, J EXP MED, V172, P1025, DOI 10.1084/jem.172.4.1025

ImperatoKalmar EL, 1997, EXP NEUROL, V145, P322, DOI 10.1006/exnr.1997.6449

KAO J, 1992, J BIOL CHEM, V267, P20239

KAO J, 1994, J BIOL CHEM, V269, P25106

KAO J, 1994, J BIOL CHEM, V269, P9774

Kaya SS, 1999, BRAIN RES, V818, P23, DOI 10.1016/S0006-8993(98)01204-9

Knies UE, 1998, P NATL ACAD SCI USA, V95, P12322, DOI 10.1073/pnas.95.21.12322

LOTAN M, 1994, FASEB J, V8, P1026, DOI 10.1096/fasebj.8.13.7926367

LU X, 1991, J NEUROSCI, V11, P972

MALLAT M, 1994, J LEUKOCYTE BIOL, V56, P416, DOI 10.1002/jlb.56.3.416

MAXWELL WL, 1990, PHILOS T R SOC B, V328, P479, DOI 10.1098/rstb.1990.0121

Maxwell WL, 1997, J NEUROTRAUM, V14, P419, DOI 10.1089/neu.1997.14.419

MCCLAIN C, 1991, J LAB CLIN MED, V118, P225

McIntosh TK, 1996, LAB INVEST, V74, P315

MORGANTIKOSSMANN MC, 1992, TRENDS PHARMACOL SCI, V13, P286, DOI 10.1016/0165-6147(92)90087-M

MORI E, 1992, STROKE, V23, P712, DOI 10.1161/01.STR.23.5.712

MULLIN JM, 1990, CANCER RES, V50, P2172

Murray JC, 2000, AM J PATHOL, V157, P2045, DOI 10.1016/S0002-9440(10)64843-2

Nag S, 1997, J NEUROPATH EXP NEUR, V56, P912, DOI 10.1097/00005072-199708000-00009

Newcomb JK, 1999, EXP NEUROL, V158, P76, DOI 10.1006/exnr.1999.7071

Orihara Y, 2001, FORENSIC SCI INT, V123, P142, DOI 10.1016/S0379-0738(01)00537-0

OTT L, 1994, J NEUROTRAUM, V11, P447, DOI 10.1089/neu.1994.11.447

PIANI D, 1991, NEUROSCI LETT, V133, P159, DOI 10.1016/0304-3940(91)90559-C

Popovich PG, 1997, J COMP NEUROL, V377, P443, DOI 10.1002/(SICI)1096-9861(19970120)377:3<443::AID-CNE10>3.0.CO;2-S

QUAGLIARELLO VJ, 1991, J CLIN INVEST, V87, P1360, DOI 10.1172/JCI115140

Rabchevsky AG, 1997, J NEUROSCI RES, V47, P34

Raivich G, 1999, BRAIN RES REV, V30, P77, DOI 10.1016/S0165-0173(99)00007-7

Rapalino O, 1998, NAT MED, V4, P814, DOI 10.1038/nm0798-814

RINK A, 1995, AM J PATHOL, V147, P1575

RIVADEPATY I, 1994, EXP NEUROL, V128, P77, DOI 10.1006/exnr.1994.1114

Schluesener HJ, 1997, GLIA, V20, P365, DOI 10.1002/(SICI)1098-1136(199708)20:4<365::AID-GLIA8>3.0.CO;2-4

Schluesener HJ, 1998, GLIA, V24, P244, DOI 10.1002/(SICI)1098-1136(199810)24:2<244::AID-GLIA9>3.0.CO;2-3

Schwab JM, 2001, J NEUROTRAUM, V18, P881, DOI 10.1089/089771501750451802

Schwab ME, 1996, PHYSIOL REV, V76, P319, DOI 10.1152/physrev.1996.76.2.319

Schwartz M, 1999, TRENDS NEUROSCI, V22, P295, DOI 10.1016/S0166-2236(99)01405-8

Stoll G, 1999, PROG NEUROBIOL, V58, P233, DOI 10.1016/S0301-0082(98)00083-5

STREIT WJ, 1993, J CHEM NEUROANAT, V6, P261, DOI 10.1016/0891-0618(93)90047-8

Streit WJ, 1999, PROG NEUROBIOL, V57, P563, DOI 10.1016/S0301-0082(98)00069-0

STREIT WJ, 1988, J COMP NEUROL, V268, P248, DOI 10.1002/cne.902680209

Tas MPR, 1996, INT J BIOCHEM CELL B, V28, P837, DOI 10.1016/1357-2725(96)00038-6

TERADA LS, 1992, INFLAMMATION, V16, P13, DOI 10.1007/BF00917511

Wakasugi K, 1999, SCIENCE, V284, P147, DOI 10.1126/science.284.5411.147

WOODROOFE MN, 1991, J NEUROIMMUNOL, V33, P227, DOI 10.1016/0165-5728(91)90110-S

Zheng M, 2001, AM J PATHOL, V159, P1021, DOI 10.1016/S0002-9440(10)61777-4

NR 67

TC 31

Z9 33

U1 0

U2 1

PU ELSEVIER

PI AMSTERDAM

PA RADARWEG 29, 1043 NX AMSTERDAM, NETHERLANDS

SN 0165-5728

EI 1872-8421

J9 J NEUROIMMUNOL

JI J. Neuroimmunol.

PD FEB

PY 2003

VL 135

IS 1-2

BP 1

EP 9

DI 10.1016/S0165-5728(02)00427-7

PG 9

WC Immunology; Neurosciences

WE Science Citation Index Expanded (SCI-EXPANDED)

SC Immunology; Neurosciences & Neurology

GA 651XW

UT WOS:000181349500001

PM 12576219

DA 2024-03-03

ER

PT J

AU Delgado, M

Ganea, D

AF Delgado, M

Ganea, D

TI Vasoactive intestinal peptide prevents activated microglia-induced

neurodegeneration under inflammatory conditions: potential therapeutic

role in brain trauma

SO FASEB JOURNAL

LA English

DT Article

DE VIP; inflammation; endotoxin; cytokines; neuropeptides

ID TUMOR-NECROSIS-FACTOR; SPINAL-CORD-INJURY; MESSENGER-RNA; FACTOR-ALPHA;

RAT-BRAIN; CHEMOKINE PRODUCTION; TNF-ALPHA; IN-VITRO; KAPPA-B; PITUITARY

AB In most neurodegenerative disorders, including multiple sclerosis, Parkinson's disease, and Alzheimer's disease, a massive neuronal cell death occurs as a consequence of an uncontrolled inflammatory response, where activated microglia and its cytotoxic agents play a crucial pathologic role. Because current treatments for these diseases are not effective, several regulatory molecules termed "microglia-deactivating factors" recently have been the focus of considerable research. Vasoactive intestinal peptide ( VIP) is a neuropeptide with a potent antiinflammatory effect, which has been found to protect from other inflammatory disorders, such as endotoxic shock and rheumatoid arthritis. In the present study, we investigate the effect of VIP on inflammation-mediated neurodegeneration in vitro and in vivo as well as on the putative neuroprotective effect of VIP on experimental pathological conditions in which central nervous system (CNS) inflammation is involved, such as brain trauma. The involvement of activated microglia and their derived cytotoxic products is also studied. VIP has a clear neuroprotective effect on inflammatory conditions by inhibiting the production of microglia-derived proinflammatory factors (tumor necrosis factor alpha, interleukin-1beta, nitric oxide). In this sense, VIP prevents neuronal cell death following brain trauma by reducing the inflammatory response of neighboring microglia. Therefore, VIP emerges as a valuable neuroprotective agent for the treatment of pathologic conditions of the CNS where inflammation-induced neurodegeneration occurs.

C1 CSIC, Inst Parasitol & Biomed Lopez Neyra, Granada 18001, Spain.

Rutgers State Univ, Dept Biol Sci, Newark, NJ 07102 USA.

C3 Consejo Superior de Investigaciones Cientificas (CSIC); CSIC - Instituto

de Parasitologia y Biomedicina Lopez-Neyra (IPBLN); Rutgers University

System; Rutgers University Newark; Rutgers University New Brunswick

RP Delgado, M (corresponding author), CSIC, Inst Parasitol & Biomed Lopez Neyra, Granada 18001, Spain.

EM mdelgado@ipb.csic.es

RI Delgado, Mario/P-1524-2016

OI Delgado, Mario/0000-0003-1893-5982

CR Bartholdi D, 1997, EUR J NEUROSCI, V9, P1422, DOI 10.1111/j.1460-9568.1997.tb01497.x

Carlson SL, 1998, EXP NEUROL, V151, P77, DOI 10.1006/exnr.1998.6785

CHAO CC, 1992, J IMMUNOL, V149, P2736

DAVID S, 1990, NEURON, V5, P463, DOI 10.1016/0896-6273(90)90085-T

de Yébenes EG, 1999, J NEUROCHEM, V73, P812, DOI 10.1046/j.1471-4159.1999.0730812.x

Delgado M, 1998, J BIOL CHEM, V273, P31427, DOI 10.1074/jbc.273.47.31427

Delgado M, 1999, J IMMUNOL, V162, P4685

Delgado M, 1999, J IMMUNOL, V162, P2358

Delgado M, 1999, J NEUROIMMUNOL, V96, P167, DOI 10.1016/S0165-5728(99)00023-5

Delgado M, 2003, FASEB J, V17, P944, DOI 10.1096/fj.02-0799fje

Delgado M, 2003, J LEUKOCYTE BIOL, V73, P155, DOI 10.1189/jlb.0702372

Delgado M, 1999, J IMMUNOL, V162, P1200

Delgado M, 2002, GLIA, V39, P148, DOI 10.1002/glia.10098

Delgado M, 2001, J IMMUNOL, V167, P966, DOI 10.4049/jimmunol.167.2.966

FRANK M, 1996, GLIA, V7, P146

Ganea D, 2001, MICROBES INFECT, V3, P141, DOI 10.1016/S1286-4579(00)01361-7

GIULIAN D, 1989, J NEUROSCI, V9, P4416

Gomariz RP, 2001, CURR PHARM DESIGN, V7, P89, DOI 10.2174/1381612013398374

González-Scarano F, 1999, ANNU REV NEUROSCI, V22, P219, DOI 10.1146/annurev.neuro.22.1.219

Gourlet P, 1997, PEPTIDES, V18, P1539, DOI 10.1016/S0196-9781(97)00228-3

Gressens P, 1997, J CLIN INVEST, V100, P390, DOI 10.1172/JCI119545

HALL ED, 1982, SURG NEUROL, V18, P320, DOI 10.1016/0090-3019(82)90140-9

Harmar AJ, 1998, PHARMACOL REV, V50, P265

HIRSCHBERG DL, 1994, J NEUROIMMUNOL, V50, P9, DOI 10.1016/0165-5728(94)90209-7

Kim WG, 2000, J NEUROSCI, V20, P6309, DOI 10.1523/JNEUROSCI.20-16-06309.2000

KNO JY, 1987, J NEUROSCI, V20, P83

Lawrence CB, 1998, EUR J NEUROSCI, V10, P1188, DOI 10.1046/j.1460-9568.1998.00136.x

LAWSON LJ, 1990, NEUROSCIENCE, V39, P151, DOI 10.1016/0306-4522(90)90229-W

LIPTON JM, 1993, NEUROBIOLOGY CYTOK B, P61

MORENOFLORES MT, 1993, GLIA, V7, P146, DOI 10.1002/glia.440070204

Moro O, 1997, J BIOL CHEM, V272, P966, DOI 10.1074/jbc.272.2.966

Nadeau S, 2000, J NEUROSCI, V20, P3456, DOI 10.1523/JNEUROSCI.20-09-03456.2000

Offen D, 2000, BRAIN RES, V854, P257, DOI 10.1016/S0006-8993(99)02375-6

Pozo D, 2000, IMMUNOL TODAY, V21, P7, DOI 10.1016/S0167-5699(99)01525-X

Quan N, 1998, NEUROSCIENCE, V83, P281, DOI 10.1016/S0306-4522(97)00350-3

Rajora N, 1997, J NEUROSCI, V17, P2181

SAID SI, 1991, AM REV RESPIR DIS, V143, pS22, DOI 10.1164/ajrccm/143.3_Pt_2.S22

SAWADA M, 1990, BRAIN RES, V509, P119, DOI 10.1016/0006-8993(90)90317-5

SAWADA M, 1989, BRAIN RES, V491, P394, DOI 10.1016/0006-8993(89)90078-4

Schwartz M, 1999, TRENDS NEUROSCI, V22, P295, DOI 10.1016/S0166-2236(99)01405-8

SHER PK, 1990, GLIA, V3, P350, DOI 10.1002/glia.440030506

Streit WJ, 1998, EXP NEUROL, V152, P74, DOI 10.1006/exnr.1998.6835

TAUPIN V, 1993, J NEUROIMMUNOL, V42, P177, DOI 10.1016/0165-5728(93)90008-M

WHITFIELD HJ, 1990, CELL MOL NEUROBIOL, V10, P145, DOI 10.1007/BF00733641

Xia MG, 1997, J PHARMACOL EXP THER, V281, P629

[No title captured]

NR 46

TC 97

Z9 108

U1 0

U2 4

PU WILEY

PI HOBOKEN

PA 111 RIVER ST, HOBOKEN 07030-5774, NJ USA

SN 0892-6638

EI 1530-6860

J9 FASEB J

JI Faseb J.

PD AUG

PY 2003

VL 17

IS 11

BP 1922

EP +

DI 10.1096/fj.02-1029fje

PG 17

WC Biochemistry & Molecular Biology; Biology; Cell Biology

WE Science Citation Index Expanded (SCI-EXPANDED)

SC Biochemistry & Molecular Biology; Life Sciences & Biomedicine - Other

Topics; Cell Biology

GA 721YB

UT WOS:000185345100006

PM 12923064

DA 2024-03-03

ER

PT J

AU Wilson, S

Raghupathi, R

Saatman, KE

Mackinnon, MA

McIntosh, TK

Graham, DI

AF Wilson, S

Raghupathi, R

Saatman, KE

Mackinnon, MA

McIntosh, TK

Graham, DI

TI Continued <i>in situ</i> DNA fragmentation of microglia/macrophages in

white matter weeks and months after traumatic brain injury

SO JOURNAL OF NEUROTRAUMA

LA English

DT Article

DE human traumatic brain injury; TUNEL staining; Wallerian degeneration

ID SPINAL-CORD-INJURY; DIFFUSE AXONAL INJURY; MISSILE HEAD-INJURY;

CONTROLLED CORTICAL IMPACT; VEGETATIVE STATE; CELL-DEATH; APOPTOSIS;

RATS; NEUROPATHOLOGY; DAMAGE

AB Paraffin-embedded material from the pons of head-injured patients whose disability could be attributed to diffuse traumatic axonal injury, and controls, was identified from the department's archive. The cases were divided into three groups based on survival, viz Group I (n = 5) who survived for between 4 and 8 weeks, Group 2 (n = 5) for between 3 and 9 months, and Group 3 (n = 5) who survived for more that 12 months. Sections were stained by the TUNEL (TdT-mediated UTP nick end labelling) technique, and by H&E, LFB/CV and immunohistochemically for astrocytes (GFAP) and microglia/macrophages (CD68). Microscopic abnormalities were mapped onto line diagrams of two levels of the pons and quantitation of the response determined by an eye-piece graticule placed over the medial lemmisci, cortico-spinal and transverse fiber tracts. Data were pooled by region of interest. In the H&E and LFB/CV stained sections, there was variable pallor of staining in ascending and descending fiber tracts due to loss of myelin: within these same tracts there was an astrocytosis and increased numbers of microglia/macrophages compared with controls. In the white matter tracts of the controls, there was on average 1-2 TUNEL+ cells per unit area. In contrast, there were on average 2-16 TUNEL+ cells in the cortico-spinal tracts and in the medial lemnisci of all groups of head-injured patients. CD68(+) cells co-located with the TUNEL+, and their number mirrored the TUNEL I staining with on average 16-30 cells per unit area in Group 1, 14-27 cells per unit area in Group 2, and 12-14 cells per unit area in Group 3. There was a statistical association between the TUNEL+ and CD68(+) cells. Few changes were seen in the transverse fiber tracts of the pons. These findings indicate that most of the in situ DNA fragmentation occurred in microglia/macrophages in ascending and descending fiber tracts of the brain stem in which by conventional light microscopy there is Wallerian degeneration. However, in addition, a few TUNEL+ oligodendrocyte-like cells were also seen.

C1 Univ Glasgow, Dept Neuropathol, Glasgow G12 8QQ, Lanark, Scotland.

Univ Penn, Sch Med, Dept Neurosurg, Philadelphia, PA 19104 USA.

C3 University of Glasgow; University of Pennsylvania

RP Graham, DI (corresponding author), S Glasgow Hosp NHS Trust, So Gen Hosp, Acad Unit Neuropathol, Inst Neurol Sci, 1345 Govan Rd, Glasgow G51 4TF, Lanark, Scotland.

EM D.Graham@clinmed.gla.ac.uk

FU NINDS NIH HHS [P50-NS08803] Funding Source: Medline; PHS HHS

[R01-GN34690] Funding Source: Medline

CR Abe Y, 1999, J NEUROTRAUM, V16, P945, DOI 10.1089/neu.1999.16.945

ADAMS JH, 1976, NEUROPATH APPL NEURO, V2, P323, DOI 10.1111/j.1365-2990.1976.tb00506.x

Adams JH, 1999, J CLIN PATHOL, V52, P804, DOI 10.1136/jcp.52.11.804

ADAMS JH, 1977, BRAIN, V100, P489, DOI 10.1093/brain/100.3.489

ADAMS JH, 1989, HISTOPATHOLOGY, V15, P49, DOI 10.1111/j.1365-2559.1989.tb03040.x

ADAMS JH, 1982, ANN NEUROL, V12, P557, DOI 10.1002/ana.410120610

Adams JH, 2000, BRAIN, V123, P1327, DOI 10.1093/brain/123.7.1327

ADAMS JH, 1980, J CLIN PATHOL, V33, P1132, DOI 10.1136/jcp.33.12.1132

ADAMS JH, 1985, NEUROPATH APPL NEURO, V11, P299, DOI 10.1111/j.1365-2990.1985.tb00027.x

BARRES BA, 1993, DEVELOPMENT, V118, P283

Beattie MS, 1998, NEUROSCIENTIST, V4, P163, DOI 10.1177/107385849800400312

Beattie MS, 2000, J NEUROTRAUM, V17, P915, DOI 10.1089/neu.2000.17.915

Bramlett HM, 1997, ACTA NEUROPATHOL, V93, P190, DOI 10.1007/s004010050602

BUNGE RP, 1998, ADV NEUROL, V59, P75

Clark D, 1999, IEEE INTERNET COMPUT, V3, P13

Clark RSB, 1997, J NEUROSCI, V17, P9172

Colicos MA, 1996, BRAIN RES, V739, P111, DOI 10.1016/S0006-8993(96)00819-0

Conti AC, 1998, J NEUROSCI, V18, P5663

Crowe MJ, 1997, NAT MED, V3, P73, DOI 10.1038/nm0197-73

Dixon CE, 1999, J NEUROTRAUM, V16, P109, DOI 10.1089/neu.1999.16.109

Emery E, 1998, J NEUROSURG, V89, P911, DOI 10.3171/jns.1998.89.6.0911

Fowler J, 2002, CLIN NEUROPATHOL, V21, P156

GAVRIELI Y, 1992, J CELL BIOL, V119, P493, DOI 10.1083/jcb.119.3.493

Geddes JF, 2000, NEUROPATH APPL NEURO, V26, P105, DOI 10.1046/j.1365-2990.2000.026002105.x

GEHRMANN J, 1991, ACTA NEUROPATHOL, V82, P442, DOI 10.1007/BF00293378

GEHRMANN J, 1992, LAB INVEST, V67, P100

Gennarelli TA, 1998, NEUROSCIENTIST, V4, P202, DOI 10.1177/107385849800400316

GOLDSTEIN M, 1990, ANN NEUROL, V27, P327, DOI 10.1002/ana.410270315

GRAHAM DI, 1989, J NEUROL NEUROSUR PS, V52, P346, DOI 10.1136/jnnp.52.3.346

Graham DI, 2000, J NEUROPATH EXP NEUR, V59, P641, DOI 10.1093/jnen/59.8.641

Graham DI., 2002, Greenfieldfs Neuropathology, V7, P823

GRCEVIC H, 1977, RAD JUN AKAD ZNS VMJ, V402, P265

Hayes KC, 1997, J NEUROTRAUM, V14, P235, DOI 10.1089/neu.1997.14.235

KERR JFR, 1972, J PATHOL, V106, pPR11

Li GL, 1996, J NEUROPATH EXP NEUR, V55, P280, DOI 10.1097/00005072-199603000-00003

Liu XZ, 1997, J NEUROSCI, V17, P5395

McIntosh TK, 1998, NEUROPATH APPL NEURO, V24, P251

Murray GD, 1999, ACTA NEUROCHIR, V141, P223, DOI 10.1007/s007010050292

Newcomb JK, 1999, EXP NEUROL, V158, P76, DOI 10.1006/exnr.1999.7071

PEERLESS SJ, 1967, CAN MED ASSOC J, V96, P577

PETITO CK, 1995, J NEUROPATH EXP NEUR, V54, P761, DOI 10.1097/00005072-199511000-00002

Pierce JES, 1998, NEUROSCIENCE, V87, P359, DOI 10.1016/S0306-4522(98)00142-0

RINK A, 1995, AM J PATHOL, V147, P1575

Shaw K, 2001, CLIN NEUROPATHOL, V20, P106

Shuman SL, 1997, J NEUROSCI RES, V50, P798, DOI 10.1002/(SICI)1097-4547(19971201)50:5<798::AID-JNR16>3.0.CO;2-Y

Smith DH, 1997, J NEUROTRAUM, V14, P715, DOI 10.1089/neu.1997.14.715

Smith FM, 2000, ACTA NEUROPATHOL, V100, P537, DOI 10.1007/s004010000222

STRICH SJ, 1956, J NEUROL NEUROSUR PS, V19, P163, DOI 10.1136/jnnp.19.3.163

Williams S, 2001, ACTA NEUROPATHOL, V102, P581, DOI 10.1007/s004010100410

WYLLIE AH, 1993, BRIT J CANCER, V67, P205, DOI 10.1038/bjc.1993.40

Yakovlev AG, 1997, J NEUROSCI, V17, P7415

Yong C, 1998, J NEUROTRAUM, V15, P459, DOI 10.1089/neu.1998.15.459

ZIMMERMAN RA, 1978, RADIOLOGY, V127, P393, DOI 10.1148/127.2.393

[No title captured]

NR 54

TC 33

Z9 35

U1 0

U2 1

PU MARY ANN LIEBERT, INC

PI NEW ROCHELLE

PA 140 HUGUENOT STREET, 3RD FL, NEW ROCHELLE, NY 10801 USA

SN 0897-7151

EI 1557-9042

J9 J NEUROTRAUM

JI J. Neurotrauma

PD MAR

PY 2004

VL 21

IS 3

BP 239

EP 250

DI 10.1089/089771504322972031

PG 12

WC Critical Care Medicine; Clinical Neurology; Neurosciences

WE Science Citation Index Expanded (SCI-EXPANDED)

SC General & Internal Medicine; Neurosciences & Neurology

GA 806CI

UT WOS:000220411600002

PM 15115599

DA 2024-03-03

ER

PT J

AU Bellander, BM

Bendel, O

Von Euler, G

Ohlsson, M

Svensson, M

AF Bellander, BM

Bendel, O

Von Euler, G

Ohlsson, M

Svensson, M

TI Activation of microglial cells and complement following traumatic injury

in rat entorhinal-hippocampal slice cultures

SO JOURNAL OF NEUROTRAUMA

LA English

DT Article

DE complement; slice cultures; traumatic brain injury

ID SULFATED GLYCOPROTEIN-2; BRAIN MACROPHAGES; NERVOUS-SYSTEM; MYELIN;

ATTACK; PROTEIN; DEGENERATION; RECRUITMENT; INCREASE; NUCLEUS

AB The complement cascade has been suggested to be involved in development of secondary brain damage following traumatic brain injury (TBI). Previous studies have shown that reactive microglia are involved in activation of the complement cascade following various injuries to the nervous system. Macrophages seem to have a significant role in this process, but it is still unclear whether these cells, as well as the complement components, are derived from reactive microglia or if these biological events only can occur as a result from the influx of plasma and monocytes via a disrupted blood-brain barrier (BBB). The aim of this study was to investigate the response of microglial cells and the complement system in the absence of plasma/blood components following a standardized crush injury in an entorhinal-hippocampal slice culture. There was a clear increase in complement component C1q and C5b-9-IR (Membrane Attack Complex, MAC) in the area near the crush injury. MAC-IR appeared as numerous dots in clusters which co-localized with anti-NeuN labelled neurons in the injury border zone. Complement C1q-IR co-localized with reactive microglia, co-labelled with OX42 antisera. These findings show activation of the complement cascade near the injury zone and in particular, formation of MAC at the surface of neurons in this area. There was a distinct activation of microglial cells (OX42-IR) near the site of injury, as well as an increase in ED-1 expressing macrophages. In the absence of blood and plasma components it is likely that ED-1-labelled cells represent reactive microglia transformed into macrophages. In addition, Neurons (Neun-IR) near the injury were found to co-localize with clusterin-IR indicating upregulation of a defense system to the endogenous complement attack. The present study provides evidence that microglia and complement is activated in the injury border zone of the tissue slice in a similar fashion as in vivo following TBI, despite the absence of plasma/blood products and cells. These findings support the hypothesis that reactive microglia have a key role in complement activation following TBI by local synthesis of complement with a potential impact on development of secondary neuronal insults.

C1 Karolinska Hosp, Dept Clin Neurosci, Neurosurg Sect, S-17176 Stockholm, Sweden.

C3 Karolinska Institutet; Karolinska University Hospital

RP Bellander, BM (corresponding author), Karolinska Hosp, Dept Clin Neurosci, Neurosurg Sect, R3-02 Karolinska Vagen, S-17176 Stockholm, Sweden.

EM bob@ks.se

RI Ohlsson, Marcus R/H-1395-2011; Svensson, Mikael/F-8662-2012

OI Svensson, Mikael/0000-0003-1179-7003

CR AGOSTONI A, 1992, INT J IMMUNOPATH PH, V5, P123, DOI 10.1177/039463209200500207

[Anonymous], HEAD INJURY

Bellander BM, 1996, J NEUROSURG, V85, P468, DOI 10.3171/jns.1996.85.3.0468

Bellander BM, 2001, J NEUROTRAUM, V18, P1295, DOI 10.1089/08977150152725605

BHAKDI S, 1991, IMMUNOL TODAY, V12, P318, DOI 10.1016/0167-5699(91)90007-G

Blumbergs P. C., 1997, HEAD INJURY PATHOPHY, P39

BOJE KM, 1992, BRAIN RES, V587, P250, DOI 10.1016/0006-8993(92)91004-X

BROWN EJ, 1991, CURR OPIN IMMUNOL, V3, P76, DOI 10.1016/0952-7915(91)90081-B

BRUCK W, 1991, J NEUROL SCI, V103, P182, DOI 10.1016/0022-510X(91)90162-Z

CAMPBELL AK, 1981, BIOCHEM J, V194, P551, DOI 10.1042/bj1940551

CHESNUT RM, 1993, J TRAUMA, V34, P216, DOI 10.1097/00005373-199302000-00006

ESSER AF, 1991, IMMUNOL TODAY, V12, P316, DOI 10.1016/0167-5699(91)90006-F

FRANK MM, 1991, IMMUNOL TODAY, V12, P322, DOI 10.1016/0167-5699(91)90009-I

GIULIAN D, 1993, J NEUROSCI, V13, P29

GRAHAM DI, 1971, LANCET, V1, P265

HANSCH GM, 1992, IMMUNOPHARMACOLOGY, V24, P107, DOI 10.1016/0162-3109(92)90017-7

HOLMIN S, 1995, ACTA NEUROCHIR, V132, P110, DOI 10.1007/BF01404857

JENNE DE, 1989, P NATL ACAD SCI USA, V86, P7123, DOI 10.1073/pnas.86.18.7123

KATAYAMA Y, 1995, BRAIN PATHOL, V5, P427, DOI 10.1111/j.1750-3639.1995.tb00621.x

KINOSHITA T, 1991, IMMUNOL TODAY, V12, P291, DOI 10.1016/0167-5699(91)90001-A

Koshinaga M, 2000, J NEUROTRAUM, V17, P185, DOI 10.1089/neu.2000.17.185

LACHMANN PJ, 1991, IMMUNOL TODAY, V12, P312, DOI 10.1016/0167-5699(91)90005-E

MAY PC, 1992, TRENDS NEUROSCI, V15, P391, DOI 10.1016/0166-2236(92)90190-J

MCGEER PL, 1989, CAN J NEUROL SCI, V16, P516, DOI 10.1017/S0317167100029863

MCGEER PL, 1993, GLIA, V7, P84, DOI 10.1002/glia.440070114

MICHEL D, 1992, SYNAPSE, V11, P105, DOI 10.1002/syn.890110203

MILLER JD, 1977, J NEUROSURG, V47, P503, DOI 10.3171/jns.1977.47.4.0503

Morgan BP, 1999, CRIT REV IMMUNOL, V19, P173

MORGAN BP, 1994, SPRINGER SEMIN IMMUN, V15, P369, DOI 10.1007/BF01837366

MORGAN BP, 1992, CURR TOP MICROBIOL, V178, P115

MULLEREBERHARD HJ, 1986, ANNU REV IMMUNOL, V4, P503, DOI 10.1146/annurev.iy.04.040186.002443

MURPHY BF, 1988, J CLIN INVEST, V81, P1858, DOI 10.1172/JCI113531

OKUSAWA S, 1987, J IMMUNOL, V139, P2635

OKUSAWA S, 1988, J EXP MED, V168, P443, DOI 10.1084/jem.168.1.443

OLSSON T, 1992, AUTOIMMUNITY, V13, P117, DOI 10.3109/08916939209001912

PIANI D, 1991, NEUROSCI LETT, V133, P159, DOI 10.1016/0304-3940(91)90559-C

Raivich G, 1998, J NEUROSCI, V18, P5804

ROSEN H, 1990, J LEUKOCYTE BIOL, V48, P465, DOI 10.1002/jlb.48.5.465

SCHILLING MT, 1990, IEEE T POWER SYST, V5, P1, DOI 10.1109/59.49079

Soares HD, 1995, J NEUROSCI, V15, P8223

STOLL G, 1991, ANN NEUROL, V30, P147, DOI 10.1002/ana.410300205

STOPPINI L, 1991, J NEUROSCI METH, V37, P173, DOI 10.1016/0165-0270(91)90128-M

STREIT WJ, 1988, J COMP NEUROL, V268, P248, DOI 10.1002/cne.902680209

SVENSSON M, 1992, J NEUROIMMUNOL, V40, P99, DOI 10.1016/0165-5728(92)90217-9

Takayama A, 1996, PHYS PLASMAS, V3, P3, DOI 10.1063/1.871832

THERY C, 1991, EUR J NEUROSCI, V3, P1155, DOI 10.1111/j.1460-9568.1991.tb00050.x

TODD NV, 1990, ACT NEUR S, V51, P296

VACA K, 1992, EXP NEUROL, V118, P62, DOI 10.1016/0014-4886(92)90023-J

VANGURI P, 1988, J BIOL CHEM, V263, P7228

WETSEL RA, 1995, CURR OPIN IMMUNOL, V7, P48, DOI 10.1016/0952-7915(95)80028-X

YAO J, 1990, J NEUROSCI RES, V27, P36, DOI 10.1002/jnr.490270106

NR 51

TC 28

Z9 34

U1 0

U2 2

PU MARY ANN LIEBERT, INC

PI NEW ROCHELLE

PA 140 HUGUENOT STREET, 3RD FL, NEW ROCHELLE, NY 10801 USA

SN 0897-7151

EI 1557-9042

J9 J NEUROTRAUM

JI J. Neurotrauma

PD MAY

PY 2004

VL 21

IS 5

BP 605

EP 615

DI 10.1089/089771504774129937

PG 11

WC Critical Care Medicine; Clinical Neurology; Neurosciences

WE Science Citation Index Expanded (SCI-EXPANDED)

SC General & Internal Medicine; Neurosciences & Neurology

GA 821LK

UT WOS:000221461900011

PM 15165368

DA 2024-03-03

ER

PT J

AU Grossman, KJ

Goss, CW

Stein, DG

AF Grossman, KJ

Goss, CW

Stein, DG

TI Effects of progesterone on the inflammatory response to brain injury in

the rat

SO BRAIN RESEARCH

LA English

DT Article

DE progesterone; traumatic brain injury; microglia; astrocyte; edema;

neuronal survival

ID CEREBRAL-ARTERY OCCLUSION; FIBRILLARY ACIDIC PROTEIN; NITRIC-OXIDE

SYNTHASE; CONTUSION INJURY; SPINAL-CORD; CORTICAL CONTUSION; REACTIVE

GLIOSIS; CORTEX ABLATION; MICROGLIA; CNS

AB The effects of progesterone on the cellular inflammatory response to frontal cortex in jury were examined on Postsurgical days 1, 3 5, 7 and 9 in male rats treated with progesterone (4 mg/kg) and/or vehicle. Rats with bilateral contusions showed increased levels of edema on days I, 3 and 5, more reactive astrocytes on days 3, 5, 7 and 9, and more macrophages/activated microglia on days 1, 3, 5 and 9 compared to shams. The number of neurons in the medial dorsal nucleus (MDN) of the thalamus reduced on days 5 and 9 after injury compared to shams. Progesterone reduced edema levels and increased the accumulation of macrophages/activated microglia compared to vehicle controls (p<0.025); however, these changes in the inflammatory response were not related to MDN neuronal Survival. Our results Confirm the possibility that one way progesterone mediates its neuroprotective effects following injury is through its actions on the inflammatory response. (C) 2004 Elsevier B.V. All rights reserved.

C1 Emory Univ, Dept Psychol, Atlanta, GA 30322 USA.

Emory Univ, Sch Med, Dept Emergency Med, Atlanta, GA 30322 USA.

Emory Univ, Sch Med, Dept Neurol, Atlanta, GA 30322 USA.

C3 Emory University; Emory University; Emory University

RP Grossman, KJ (corresponding author), Univ Chicago, Dept Neurobiol Pharmacol & Physiol, 947 E 58th St,MC0926, Chicago, IL 60637 USA.

EM kgrossm@uchicago.edu

RI Stein, Donald/AAJ-5139-2020

FU NINDS NIH HHS [R01-NS3866401A2] Funding Source: Medline

CR Arvin B, 1996, NEUROSCI BIOBEHAV R, V20, P445, DOI 10.1016/0149-7634(95)00026-7

Asbury ET, 1998, BEHAV BRAIN RES, V97, P99, DOI 10.1016/S0166-4328(98)00031-X

Aschner M, 1998, NEUROTOXICOLOGY, V19, P269

BANATI RB, 1993, GLIA, V7, P111, DOI 10.1002/glia.440070117

BENVENISTE EN, 1990, J NEUROIMMUNOL, V30, P201, DOI 10.1016/0165-5728(90)90104-U

BOLOVENTA B, 1992, PROG BRAIN RES, V94, P367

BOUVIER M, 1992, NATURE, V360, P471, DOI 10.1038/360471a0

Bruce-Keller AJ, 1999, J NEUROSCI RES, V58, P191, DOI 10.1002/(SICI)1097-4547(19991001)58:1<191::AID-JNR17>3.0.CO;2-E

Carlos TM, 1997, J LEUKOCYTE BIOL, V61, P279, DOI 10.1002/jlb.61.3.279

Cervantes M, 2002, ARCH MED RES, V33, P6, DOI 10.1016/S0188-4409(01)00347-2

CERVOSNAVARRO J, 1991, J NEUROL SCI, V103, pS3

CHAO CC, 1992, J IMMUNOL, V149, P2736

Chen JL, 1999, J NEUROL SCI, V171, P24, DOI 10.1016/S0022-510X(99)00247-6

CHOI DW, 1992, J NEUROBIOL, V23, P1261, DOI 10.1002/neu.480230915

Coggeshall RE, 1996, J COMP NEUROL, V364, P6, DOI 10.1002/(SICI)1096-9861(19960101)364:1<6::AID-CNE2>3.0.CO;2-9

COLTON CA, 1987, FEBS LETT, V223, P284, DOI 10.1016/0014-5793(87)80305-8

DAMOISEAUX JGMC, 1994, IMMUNOLOGY, V83, P140

ENG LF, 1985, J NEUROIMMUNOL, V8, P203, DOI 10.1016/S0165-5728(85)80063-1

FEUERSTEIN GZ, 1994, CEREBROVAS BRAIN MET, V6, P341

Fujita T, 1998, ACTA NEUROCHIR, V140, P275, DOI 10.1007/s007010050095

Galani R, 2001, RESTOR NEUROL NEUROS, V18, P161

GALEA E, 1992, P NATL ACAD SCI USA, V89, P10945, DOI 10.1073/pnas.89.22.10945

GANTER S, 1992, J NEUROSCI RES, V33, P218, DOI 10.1002/jnr.490330205

García-Estrada J, 1999, INT J DEV NEUROSCI, V17, P145, DOI 10.1016/S0736-5748(98)00065-3

GARCIAESTRADA J, 1993, BRAIN RES, V628, P271, DOI 10.1016/0006-8993(93)90964-O

Ghirnikar RS, 1998, NEUROCHEM RES, V23, P329, DOI 10.1023/A:1022453332560

GIULIAN D, 1986, J EXP MED, V164, P594, DOI 10.1084/jem.164.2.594

GIULIAN D, 1985, SCIENCE, V228, P497, DOI 10.1126/science.3872478

GIULIAN D, 1990, ANN NEUROL, V27, P33, DOI 10.1002/ana.410270107

Goss CW, 2003, PHARMACOL BIOCHEM BE, V76, P231, DOI 10.1016/j.pbb.2003.07.003

HE J, 2001, J NEUROTRAUM, V18, P1140

HOFFMAN SW, 1994, J NEUROTRAUM, V11, P417, DOI 10.1089/neu.1994.11.417

HOLMIN S, 1995, ACTA NEUROCHIR, V132, P110, DOI 10.1007/BF01404857

Jiang N, 1996, BRAIN RES, V735, P101, DOI 10.1016/0006-8993(96)00605-1

KEMPSKI OS, 1994, ACTA NEUROCHIR, P7

Keppel G, 1991, DESIGN ANAL RES HDB, P169

KESSLAK JP, 1986, EXP NEUROL, V92, P377, DOI 10.1016/0014-4886(86)90089-0

KIMELBERG HK, 1995, J NEUROSURG, V83, P1051, DOI 10.3171/jns.1995.83.6.1051

Kuebler JF, 2003, ARCH SURG-CHICAGO, V138, P727, DOI 10.1001/archsurg.138.7.727

Kumon Y, 2000, J NEUROSURG, V92, P848, DOI 10.3171/jns.2000.92.5.0848

Labombarda F, 2000, J STEROID BIOCHEM, V73, P159, DOI 10.1016/S0960-0760(00)00064-9

LIEW FY, 1991, IMMUNOPARASITOL TOD, pA17, DOI 10.1016/S0167-5699(05)80006-4

MANNEL DN, 1990, IMMUNOL LETT, V26, P89, DOI 10.1016/0165-2478(90)90181-O

MILLIGAN CE, 1991, J COMP NEUROL, V314, P125, DOI 10.1002/cne.903140112

MORALI G, 2002, ABSTR SOC NEUR

Murphy SJ, 2000, STROKE, V31, P1173, DOI 10.1161/01.STR.31.5.1173

NIETOSAMPEDRO M, 1988, NEUROSCI LETT, V86, P361, DOI 10.1016/0304-3940(88)90511-3

NORENBERG MD, 1994, J NEUROPATH EXP NEUR, V53, P213, DOI 10.1097/00005072-199405000-00001

Paxinos G., 1982, RAT BRAIN

PIANI D, 1991, NEUROSCI LETT, V133, P159, DOI 10.1016/0304-3940(91)90559-C

Ramos-Remus C, 2002, J INVEST MED, V50, P458, DOI 10.1136/jim-50-06-06

Ransohoff RM, 1998, TRENDS NEUROSCI, V21, P154, DOI 10.1016/S0166-2236(97)01198-3

ROOF RL, 1992, RESTOR NEUROL NEUROS, V4, P425, DOI 10.3233/RNN-1992-4608

Roof RL, 1997, MOL CHEM NEUROPATHOL, V31, P1, DOI 10.1007/BF02815156

ROOF RL, 1994, EXP NEUROL, V129, P64, DOI 10.1006/exnr.1994.1147

Schwartz M, 1999, TRENDS NEUROSCI, V22, P295, DOI 10.1016/S0166-2236(99)01405-8

Shear DA, 2002, EXP NEUROL, V178, P59, DOI 10.1006/exnr.2002.8020

SIMMONS ML, 1992, J NEUROCHEM, V59, P897, DOI 10.1111/j.1471-4159.1992.tb08328.x

Smith JS, 2000, NEURAL PLAST, V7, P73, DOI 10.1155/NP.2000.73

SMITH JS, 1998, J NEUROTRAUM, V15, P905

Streit WJ, 1999, PROG NEUROBIOL, V57, P563, DOI 10.1016/S0301-0082(98)00069-0

Thomas AJ, 1999, SPINE, V24, P2134, DOI 10.1097/00007632-199910150-00013

Wright DW, 2001, J NEUROTRAUM, V18, P901, DOI 10.1089/089771501750451820

Zink Brian J., 1996, Emergency Medicine Clinics of North America, V14, P115

NR 64

TC 96

Z9 109

U1 0

U2 6

PU ELSEVIER SCIENCE BV

PI AMSTERDAM

PA PO BOX 211, 1000 AE AMSTERDAM, NETHERLANDS

SN 0006-8993

EI 1872-6240

J9 BRAIN RES

JI Brain Res.

PD MAY 15

PY 2004

VL 1008

IS 1

BP 29

EP 39

DI 10.1016/j.brainres.2004.02.022

PG 11

WC Neurosciences

WE Science Citation Index Expanded (SCI-EXPANDED)

SC Neurosciences & Neurology

GA 817YD

UT WOS:000221211900004

PM 15081379

DA 2024-03-03

ER

PT J

AU Rodriguez-Paez, AC

Brunschwig, JP

Bramlett, HM

AF Rodriguez-Paez, AC

Brunschwig, JP

Bramlett, HM

TI Light and electron microscopic assessment of progressive atrophy

following moderate traumatic brain injury in the rat

SO ACTA NEUROPATHOLOGICA

LA English

DT Article

DE progressive atrophy; inflammation; macrophages/microglia; traumatic

brain injury; white matter

ID AMYLOID PRECURSOR PROTEIN; ENDOTHELIAL GROWTH-FACTOR; NONDISRUPTIVE

AXONAL INJURY; NITRIC-OXIDE SYNTHASE; CLOSED-HEAD-INJURY; OPTIC-NERVE

FIBERS; INFLAMMATORY RESPONSE; STRETCH-INJURY; WHITE-MATTER;

POSTTRAUMATIC HYPOTHERMIA

AB The presence of progressive white matter atrophy following traumatic brain injury (TBI) has been reported in humans as well as in animal models. However, a quantitative analysis of progressive alterations in myelinated axons and other cellular responses to trauma has not been conducted. This study examined quantitative differences in myelinated axons from several white and gray matter structures between non-traumatized and traumatized areas at several time points up to 1 year. We hypothesize that axonal numbers decrease over time within the structures analyzed, based on our previous work demonstrating shrinkage of tissue in these vulnerable areas. Intubated, anesthetized male Sprague-Dawley rats were subjected to moderate (1.8-2.2 atm) parasagittal fluid-percussion brain injury, and perfused at various intervals after surgery. Sections from the fimbria, external capsule, thalamus and cerebral cortex from the ipsilateral hemisphere of traumatized and sham-operated animals were prepared and. estimated total numbers of myelinated axons were determined by systematic random sampling. Electron micrographs were obtained for ultrastructural analysis. A significant (P < 0.05) reduction in the number of myelinated axons in the traumatized hemisphere compared to control in all structures was observed. In addition, thalamic and cortical axonal counts decreased significantly (P < 0.05) over time. Swollen axons and macrophage/microglia infiltration were present as late as 6 months post-TBI in various structures. This study is the first to describe quantitatively chronic axonal changes in vulnerable brains regions after injury. Based on these data, a time-dependent decrease in the number of myelinated axons is seen to occur in vulnerable gray matter regions including the cerebral cortex and thalamus along with distinct morphological changes within white matter tracts after TBI. Although this progressive axonal response to TBI may include Wallerian degeneration, other potential mechanisms underlying this progressive pathological response within the white matter are discussed.

C1 Univ Miami, Dept Neurol Surg, Miami, FL 33136 USA.

Univ Miami, Miller Sch Med, Dept Neurosurg, Miami Project Cure Paralysis,Neurotrauma Res Ctr, Miami, FL 33152 USA.

C3 University of Miami; University of Miami

RP Bramlett, HM (corresponding author), Univ Miami, Dept Neurol Surg, 1095 NW 14th Terrace, Miami, FL 33136 USA.

EM hbramlett@miami.edu

CR ADAMS JH, 1991, J NEUROL NEUROSUR PS, V54, P481, DOI 10.1136/jnnp.54.6.481

ADAMS JH, 1989, HISTOPATHOLOGY, V15, P49, DOI 10.1111/j.1365-2559.1989.tb03040.x

ANDERSON CV, 1995, J NEUROPSYCH CLIN N, V7, P42

Arvin B, 1996, NEUROSCI BIOBEHAV R, V20, P445, DOI 10.1016/0149-7634(95)00026-7

BIAGAS KV, 1992, J NEUROTRAUM, V9, P363, DOI 10.1089/neu.1992.9.363

BLUMBERGS PC, 1994, LANCET, V344, P1055, DOI 10.1016/S0140-6736(94)91712-4

Bramlett HM, 2004, J CEREBR BLOOD F MET, V24, P133, DOI 10.1097/01.WCB.0000111614.19196.04

Bramlett HM, 1997, J NEUROPATH EXP NEUR, V56, P1132, DOI 10.1097/00005072-199710000-00007

Bramlett HM, 1997, ACTA NEUROPATHOL, V93, P190, DOI 10.1007/s004010050602

Bramlett HM, 2002, ACTA NEUROPATHOL, V103, P607, DOI 10.1007/s00401-001-0510-8

Braun H, 2002, J NEUROTRAUM, V19, P975, DOI 10.1089/089771502320317122

BULLOCK R, 1991, J NEUROL NEUROSUR PS, V54, P427, DOI 10.1136/jnnp.54.5.427

Carlos TM, 1997, J LEUKOCYTE BIOL, V61, P279, DOI 10.1002/jlb.61.3.279

Chatzipanteli K, 2000, J CEREBR BLOOD F MET, V20, P531, DOI 10.1097/00004647-200003000-00012

Chen XH, 2003, J NEUROTRAUM, V20, P623, DOI 10.1089/089771503322144545

Chirumamilla S, 2002, J NEUROTRAUM, V19, P693, DOI 10.1089/08977150260139084

Chodobski A, 2003, NEUROSCIENCE, V122, P853, DOI 10.1016/j.neuroscience.2003.08.055

CRUTCHER KA, 1990, ACTA NEUROBIOL EXP, V50, P115

Csuka E, 2000, NEUROREPORT, V11, P2587, DOI 10.1097/00001756-200008030-00047

CULLUM CM, 1986, J CLIN EXP NEUROPSYC, V8, P437, DOI 10.1080/01688638608401333

Dixon CE, 1999, J NEUROTRAUM, V16, P109, DOI 10.1089/neu.1999.16.109

FEUERSTEIN GZ, 1994, CEREBROVAS BRAIN MET, V6, P341

FURUKAWA S, 1986, BIOCHEM BIOPH RES CO, V136, P57, DOI 10.1016/0006-291X(86)90876-4

Gahm C, 2000, NEUROSURGERY, V46, P169, DOI 10.1093/neurosurgery/46.1.169

Gale S D, 1995, J Int Neuropsychol Soc, V1, P17

GENNARELLI TA, 1982, ANN NEUROL, V12, P564, DOI 10.1002/ana.410120611

Gentleman SM, 2004, FORENSIC SCI INT, V146, P97, DOI 10.1016/j.forsciint.2004.06.027

GENTLEMAN SM, 1995, ACTA NEUROPATHOL, V89, P537

Goss JR, 1998, EXP NEUROL, V149, P301, DOI 10.1006/exnr.1997.6712

Grad S, 1998, CLIN CHEM LAB MED, V36, P379, DOI 10.1515/CCLM.1998.064

GRAHAM DI, 1995, NEUROPATH APPL NEURO, V21, P27, DOI 10.1111/j.1365-2990.1995.tb01025.x

Graham DI, 2000, J NEUROPATH EXP NEUR, V59, P641, DOI 10.1093/jnen/59.8.641

Graham DI, 2000, ACTA NEUROPATHOL, V99, P117, DOI 10.1007/PL00007414

Hamberger A, 2003, J NEUROTRAUM, V20, P169, DOI 10.1089/08977150360547080

Holmin S, 1997, J NEUROSURG, V86, P493, DOI 10.3171/jns.1997.86.3.0493

HUGHES CCW, 1987, J NEUROL SCI, V79, P101, DOI 10.1016/0022-510X(87)90264-4

Jafari SS, 1998, J NEUROTRAUM, V15, P955, DOI 10.1089/neu.1998.15.955

Jafari SS, 1997, J NEUROCYTOL, V26, P207, DOI 10.1023/A:1018588114648

KIMBELBERG HK, 1994, NEUROBIOLOGY CENTRAL, P193

Kinoshita K, 2002, NEUROSURGERY, V51, P195, DOI 10.1097/00006123-200207000-00027

Kunz T, 2002, J NEUROTRAUM, V19, P1051, DOI 10.1089/089771502760341965

Leclercq PD, 2002, J NEUROTRAUM, V19, P1183, DOI 10.1089/08977150260337985

Leclercq PD, 2001, J NEUROTRAUM, V18, P1, DOI 10.1089/089771501750055721

LEWEN A, 1995, NEUROREPORT, V6, P357, DOI 10.1097/00001756-199501000-00032

Li SX, 2000, J NEUROPHYSIOL, V84, P1116

Liberto CM, 2004, J NEUROCHEM, V89, P1092, DOI 10.1111/j.1471-4159.2004.02420.x

Liedtke W, 1996, NEURON, V17, P607, DOI 10.1016/S0896-6273(00)80194-4

Liu D, 1999, MOL BRAIN RES, V68, P29, DOI 10.1016/S0169-328X(99)00063-7

LoPachin RM, 1997, TOXICOL APPL PHARM, V143, P233, DOI 10.1006/taap.1997.8106

Lu J, 2003, NEUROSCI LETT, V339, P147, DOI 10.1016/S0304-3940(03)00003-X

Maxwell WL, 1997, J NEUROTRAUM, V14, P603, DOI 10.1089/neu.1997.14.603

Maxwell WL, 1996, MICROSC RES TECHNIQ, V34, P522, DOI 10.1002/(SICI)1097-0029(19960815)34:6<522::AID-JEMT4>3.0.CO;2-L

MAXWELL WL, 1991, J NEUROCYTOL, V20, P157, DOI 10.1007/BF01186989

Maxwell WL, 1999, J NEUROTRAUM, V16, P273, DOI 10.1089/neu.1999.16.273

Maxwell WL, 2003, J NEUROTRAUM, V20, P151, DOI 10.1089/08977150360547071

MAXWELL WL, 1993, ACTA NEUROPATHOL, V86, P136, DOI 10.1007/BF00334880

Maxwell WL, 1997, J NEUROTRAUM, V14, P419, DOI 10.1089/neu.1997.14.419

McKenzie KJ, 1996, ACTA NEUROPATHOL, V92, P608, DOI 10.1007/s004010050568

Medana IM, 2003, BRAIN, V126, P515, DOI 10.1093/brain/awg061

Morganti-Kossmann Maria Cristina, 2002, Curr Opin Crit Care, V8, P101

MorgantiKossman MC, 1997, MOL PSYCHIATR, V2, P133, DOI 10.1038/sj.mp.4000227

MURPHY S, 1987, NEUROSCIENCE, V22, P381, DOI 10.1016/0306-4522(87)90342-3

Nag S, 2002, J NEUROPATH EXP NEUR, V61, P778, DOI 10.1093/jnen/61.9.778

Nonaka M, 1999, J NEUROTRAUM, V16, P1023, DOI 10.1089/neu.1999.16.1023

Orihara Y, 2001, FORENSIC SCI INT, V123, P142, DOI 10.1016/S0379-0738(01)00537-0

Paxinos G., 1997, RAT BRAIN STEROTAXIC, VThird

Peters A., 1976, FINE STRUCTURE NERVO

Pettus EH, 1996, BRAIN RES, V722, P1, DOI 10.1016/0006-8993(96)00113-8

Pierce JES, 1996, J NEUROSCI, V16, P1083

Pierce JES, 1998, NEUROSCIENCE, V87, P359, DOI 10.1016/S0306-4522(98)00142-0

Povlishock JT, 1996, ACT NEUR S, V66, P81

POVLISHOCK JT, 1995, BRAIN PATHOL, V5, P415, DOI 10.1111/j.1750-3639.1995.tb00620.x

POVLISHOCK JT, 1995, J NEUROTRAUM, V12, P555, DOI 10.1089/neu.1995.12.555

Rosenberg LJ, 1997, J NEUROTRAUM, V14, P823, DOI 10.1089/neu.1997.14.823

Schwab JM, 2001, J NEUROTRAUM, V18, P881, DOI 10.1089/089771501750451802

SHERRIFF FE, 1994, ACTA NEUROPATHOL, V87, P55

SHOHAMI E, 1994, J CEREBR BLOOD F MET, V14, P615, DOI 10.1038/jcbfm.1994.76

Shore PM, 2004, NEUROSURGERY, V54, P605, DOI 10.1227/01.NEU.0000108642.88724.DB

Smith DH, 1997, J NEUROTRAUM, V14, P715, DOI 10.1089/neu.1997.14.715

Soares HD, 1995, J NEUROSCI, V15, P8223

Strauss KI, 2000, J NEUROTRAUM, V17, P695, DOI 10.1089/089771500415436

Tanaka K, 2003, BRAIN RES, V989, P172, DOI 10.1016/S0006-8993(03)03317-1

van der Naalt J, 1999, ANN NEUROL, V46, P70, DOI 10.1002/1531-8249(199907)46:1<70::AID-ANA11>3.0.CO;2-L

VEGA JA, 1994, MECH AGEING DEV, V73, P9, DOI 10.1016/0047-6374(94)90033-7

Wada K, 1998, NEUROSURGERY, V43, P1427, DOI 10.1097/00006123-199812000-00096

WEST RW, 1993, CARCINOGENESIS, V14, P285, DOI 10.1093/carcin/14.2.285

NR 86

TC 72

Z9 84

U1 0

U2 2

PU SPRINGER

PI NEW YORK

PA 233 SPRING ST, NEW YORK, NY 10013 USA

SN 0001-6322

EI 1432-0533

J9 ACTA NEUROPATHOL

JI Acta Neuropathol.

PD JUN

PY 2005

VL 109

IS 6

BP 603

EP 616

DI 10.1007/s00401-005-1010-z

PG 14

WC Clinical Neurology; Neurosciences; Pathology

WE Science Citation Index Expanded (SCI-EXPANDED)

SC Neurosciences & Neurology; Pathology

GA 940KE

UT WOS:000230142600007

PM 15877231

DA 2024-03-03

ER

PT J

AU Cernak, I

Stoica, B

Byrnes, KR

Di Giovanni, S

Faden, AI

AF Cernak, I

Stoica, B

Byrnes, KR

Di Giovanni, S

Faden, AI

TI Role of the cell cycle in the pathobiology of central nervous system

trauma

SO CELL CYCLE

LA English

DT Article

DE cell cycle; apoptosis; neuronal injury; traumatic brain injury;

astrocyte proliferation; microglial proliferation; neuroprotection;

flavopiridol

ID BLOOD-BRAIN-BARRIER; SPINAL-CORD-INJURY; STIMULATED HOMOLOGOUS

MACROPHAGES; KINASE INHIBITOR FLAVOPIRIDOL; IN-VITRO;

ALZHEIMERS-DISEASE; DEPENDENT KINASES; MICROGLIAL ACTIVATION; NEURONAL

APOPTOSIS; PARKINSONS-DISEASE

AB Upregulation of cell cycle proteins occurs in both mitotic and post-mitotic neural cells after central nervous system (CNS) injury in adult animals. In mitotic cells, such as astroglia and microglia, they induce proliferation, whereas in post-mitotic cells such as neurons they initiate caspase-related apoptosis. We recently reported that early central administration of the cell cycle inhibitor flavopiridol after experimental traumatic brain injury (TBI) significantly reduced lesion volume, scar formation and neuronal cell death, while promoting near complete behavioral recovery. Here we show that in primary neuronal or astrocyte cultures structurally different cell cycle inhibitors ( flavopiridol, roscovitine, and olomoucine) significantly reduce upregulation of cell cycle proteins, attenuate neuronal cell death induced by etoposide, and decrease astrocyte proliferation. Flavopiridol, in a concentration dependent manner, also attenuates proliferation/ activation of microglia. In addition, we demonstrate that central administration of flavopiridol improves functional outcome in dose-dependent manner after fluid percussion induced brain injury in rats. Moreover, delayed systemic administration of flavopiridol significantly reduces brain lesion volume and edema development after TBI. These data provide further support for the therapeutic potential of cell cycle inhibitors for the treatment of clinical CNS injury and that protective mechanisms likely include reduction of neuronal cell death, inhibition of glial proliferation and attenuation of microglial activation.

C1 Georgetown Univ, Med Ctr, Dept Neurosci, Lab Study CNS Injury, Washington, DC 20057 USA.

C3 Georgetown University

RP Faden, AI (corresponding author), Georgetown Univ, Med Ctr, Dept Neurosci, Lab Study CNS Injury, 3970 Reservoir Rd NW, Washington, DC 20057 USA.

EM fadena@georgetown.edu

RI Cernak, Ibolja/A-6399-2008; STOICA, BOGDAN/H-9782-2013

OI STOICA, BOGDAN/0000-0002-2501-6434; Ibolja, Cernak/0000-0003-3214-698X;

Byrnes, Kimberly/0000-0002-7501-7734

FU NICHD NIH HHS [HD4-0677] Funding Source: Medline; NINDS NIH HHS

[NS-1-2339] Funding Source: Medline; PHS HHS [36537-03] Funding Source:

Medline

CR ABRAHAM RT, 1995, BIOL CELL, V83, P105, DOI 10.1016/0248-4900(96)81298-6

AKIYAMA H, 1988, J NEUROSCI RES, V20, P147, DOI 10.1002/jnr.490200202

AKUNDI RS, 2005, IN PRESS GLIA

Albensi BC, 2000, EXP NEUROL, V162, P61, DOI 10.1006/exnr.2000.7256

Allan SM, 2003, PHILOS T R SOC B, V358, P1669, DOI 10.1098/rstb.2003.1358

Bellander BM, 2004, J NEUROTRAUM, V21, P605, DOI 10.1089/089771504774129937

Blagosklonny MV, 2004, CELL CYCLE, V3, P1537, DOI 10.4161/cc.3.12.1278

Blamire AM, 2000, J NEUROSCI, V20, P8153

Bonmann E, 1997, NEUROSCI LETT, V230, P109, DOI 10.1016/S0304-3940(97)00485-0

Boonstra J, 2003, J CELL BIOCHEM, V90, P244, DOI 10.1002/jcb.10617

Brar SS, 2002, AM J PHYSIOL-LUNG C, V282, pL782, DOI 10.1152/ajplung.00206.2001

Bruns TJ, 2003, EPILEPSIA, V44, P2, DOI 10.1046/j.1528-1157.44.s10.3.x

Coqueret O, 2003, TRENDS CELL BIOL, V13, P65, DOI 10.1016/S0962-8924(02)00043-0

Dai Yun, 2004, Curr Oncol Rep, V6, P123, DOI 10.1007/s11912-004-0024-3

Daly J, 1998, NEUROBIOL AGING, V19, P619, DOI 10.1016/S0197-4580(98)00100-6

Di Giovanni S, 2005, P NATL ACAD SCI USA, V102, P8333, DOI 10.1073/pnas.0500989102

Di Giovanni S, 2003, ANN NEUROL, V53, P454, DOI 10.1002/ana.10472

DUSART I, 1994, EUR J NEUROSCI, V6, P712, DOI 10.1111/j.1460-9568.1994.tb00983.x

Eikelenboom P, 2002, GLIA, V40, P232, DOI 10.1002/glia.10146

Faden AI, 2003, J CEREBR BLOOD F MET, V23, P342, DOI 10.1097/01.WCB.0000046143.31247.FD

Faden AI, 2002, CURR OPIN NEUROL, V15, P707, DOI 10.1097/00019052-200212000-00008

FADEN AI, 1989, SCIENCE, V244, P798, DOI 10.1126/science.2567056

Fischer Peter M, 2003, Prog Cell Cycle Res, V5, P235

Fitch MT, 1999, J NEUROSCI, V19, P8182

Fitch MT, 1997, EXP NEUROL, V148, P587, DOI 10.1006/exnr.1997.6701

GALLO V, 1995, J NEUROSCI, V15, P394

Gao HM, 2002, J NEUROCHEM, V81, P1285, DOI 10.1046/j.1471-4159.2002.00928.x

GIULIAN D, 1988, Brain Behavior and Immunity, V2, P352, DOI 10.1016/0889-1591(88)90040-2

GIULIAN D, 1986, J NEUROSCI, V6, P2163

Grewal RP, 1999, NEUROSCI LETT, V271, P65, DOI 10.1016/S0304-3940(99)00496-6

Hains BC, 2001, J NEUROTRAUM, V18, P409, DOI 10.1089/089771501750170994

HANSTOCK CC, 1994, STROKE, V25, P843, DOI 10.1161/01.STR.25.4.843

Harris FM, 2004, J BIOL CHEM, V279, P3862, DOI 10.1074/jbc.M309475200

Hashimoto M, 2005, NEUROREPORT, V16, P99, DOI 10.1097/00001756-200502080-00004

HOEPPNER TJ, 1986, EXP NEUROL, V94, P519, DOI 10.1016/0014-4886(86)90235-9

Hunot S, 2003, ANN NEUROL, V53, pS49, DOI 10.1002/ana.10481

Kato H, 2003, BRAIN RES BULL, V60, P215, DOI 10.1016/S0361-9230(03)00036-4

Kim HRC, 1999, CANCER RES, V59, P4148

Kim SY, 2002, MOL CELLS, V13, P429

KIMELBERG HK, 1989, SCI AM, V260, P66, DOI 10.1038/scientificamerican0489-66

Lee SM, 2003, J NEUROTRAUM, V20, P1017, DOI 10.1089/089771503770195867

Lynch NJ, 2004, MOL IMMUNOL, V40, P709, DOI 10.1016/j.molimm.2003.08.009

McGeer PL, 2004, ANN NY ACAD SCI, V1035, P104, DOI 10.1196/annals.1332.007

McGraw J, 2001, J NEUROSCI RES, V63, P109, DOI 10.1002/1097-4547(20010115)63:2<109::AID-JNR1002>3.0.CO;2-J

MCINTOSH TK, 1989, NEUROSCIENCE, V28, P233, DOI 10.1016/0306-4522(89)90247-9

Meijer L, 2003, ACCOUNTS CHEM RES, V36, P417, DOI 10.1021/ar0201198

Moore S, 1996, PROG NEUROBIOL, V48, P441, DOI 10.1016/0301-0082(95)00051-8

Movsesyan VA, 2001, EXP NEUROL, V167, P366, DOI 10.1006/exnr.2000.7567

Muessel MJ, 2000, BRAIN RES, V870, P211, DOI 10.1016/S0006-8993(00)02450-1

Mukhin AG, 1997, J NEUROTRAUM, V14, P651, DOI 10.1089/neu.1997.14.651

Natale JE, 2003, J NEUROTRAUM, V20, P907, DOI 10.1089/089771503770195777

Nguyen MD, 2002, CELL DEATH DIFFER, V9, P1294, DOI 10.1038/sj.cdd.4401108

Nishitani H, 2002, GENES CELLS, V7, P523, DOI 10.1046/j.1365-2443.2002.00544.x

Obaya AJ, 2002, CELL MOL LIFE SCI, V59, P126, DOI 10.1007/s00018-002-8410-1

OKANO HJ, 1993, J NEUROSCI, V13, P2930

Osuga H, 2000, P NATL ACAD SCI USA, V97, P10254, DOI 10.1073/pnas.170144197

Prewitt CMF, 1997, EXP NEUROL, V148, P433, DOI 10.1006/exnr.1997.6694

QUAGLIARELLO VJ, 1991, J CLIN INVEST, V87, P1360, DOI 10.1172/JCI115140

Rabchevsky AG, 1997, J NEUROSCI RES, V47, P34

Rapalino O, 1998, NAT MED, V4, P814, DOI 10.1038/nm0798-814

Schultz C, 2004, NEUROBIOL AGING, V25, P397, DOI 10.1016/S0197-4580(03)00113-1

Schwartz GK, 2001, J CLIN ONCOL, V19, P1985, DOI 10.1200/JCO.2001.19.7.1985

Schwartz M, 1999, NEUROSURGERY, V44, P1041, DOI 10.1097/00006123-199905000-00057

Senderowicz AM, 2003, ONCOGENE, V22, P6609, DOI 10.1038/sj.onc.1206954

Shaked I, 2005, J NEUROCHEM, V92, P997, DOI 10.1111/j.1471-4159.2004.02954.x

Shapiro GI, 2001, CLIN CANCER RES, V7, P1590

SHERR CJ, 1993, CELL, V73, P1059, DOI 10.1016/0092-8674(93)90636-5

Silver J, 2004, NAT REV NEUROSCI, V5, P146, DOI 10.1038/nrn1326

SINENSKY MC, 1995, TOXICOL LETT, V75, P101

Sugita Y, 1997, J NEUROSCI RES, V49, P710, DOI 10.1002/(SICI)1097-4547(19970915)49:6<710::AID-JNR5>3.0.CO;2-A

Swanson RA, 2004, CURR MOL MED, V4, P193, DOI 10.2174/1566524043479185

Swanton C, 2004, LANCET ONCOL, V5, P27, DOI 10.1016/S1470-2045(03)01321-4

Tolias Christos M, 2004, NeuroRx, V1, P71, DOI 10.1602/neurorx.1.1.71

Tsan MF, 2001, AM J PHYSIOL-CELL PH, V280, pC1422

Verdaguer E, 2002, NEUROREPORT, V13, P413, DOI 10.1097/00001756-200203250-00010

Vink R, 2004, EXPERT OPIN INV DRUG, V13, P1263, DOI 10.1517/13543784.13.10.1263

VINK R, 1988, J BIOL CHEM, V263, P757

Waetzig V, 2005, GLIA, V50, P235, DOI 10.1002/glia.20173

Wang FH, 2002, J CEREBR BLOOD F MET, V22, P171, DOI 10.1097/00004647-200202000-00005

Wartiovaara K, 2002, J NEUROSCI, V22, P815, DOI 10.1523/JNEUROSCI.22-03-00815.2002

Wiessner C, 1996, NEUROSCIENCE, V72, P947, DOI 10.1016/0306-4522(95)00601-X

XU J, 1992, J NEUROTRAUM, V9, P245, DOI 10.1089/neu.1992.9.245

Yakovlev AG, 1997, J NEUROSCI, V17, P7415

Zhang ZG, 1997, BRAIN RES, V744, P189, DOI 10.1016/S0006-8993(96)01085-2

NR 84

TC 98

Z9 108

U1 0

U2 7

PU TAYLOR & FRANCIS INC

PI PHILADELPHIA

PA 530 WALNUT STREET, STE 850, PHILADELPHIA, PA 19106 USA

SN 1538-4101

EI 1551-4005

J9 CELL CYCLE

JI Cell Cycle

PD SEP

PY 2005

VL 4

IS 9

BP 1286

EP 1293

DI 10.4161/cc.4.9.1996

PG 8

WC Cell Biology

WE Science Citation Index Expanded (SCI-EXPANDED)

SC Cell Biology

GA 959JB

UT WOS:000231512900030

PM 16082214

OA Bronze

DA 2024-03-03

ER

PT J

AU Zhang, ZR

Artelt, M

Bernet, M

Trautmann, K

Schluesener, HJ

AF Zhang, ZR

Artelt, M

Bernet, M

Trautmann, K

Schluesener, HJ

TI Lesional accumulation of P2X<sub>4</sub> receptor<SUP>+</SUP> monocytes

following experimental traumatic brain injury

SO EXPERIMENTAL NEUROLOGY

LA English

DT Article

DE P2X(4) receptor; traumatic brain injury; microglia/macrophages

ID SPINAL MICROGLIA; UP-REGULATION; CELL-DEATH; ATP; ACTIVATION;

MACROPHAGES; MECHANISMS; EXPRESSION; STRIATUM; RELEASE

AB P2X(4) receptor (P2X(4)R) is an ATP-gated ion channel. ATP is an important messenger in traumatic brain injury. Here, we report expression of P2X(4)R in rat traumatic brain injury with focus on the early phase, most amenable to therapy. Accumulation of P2X(4)R(+) cells was observed as early as 6 h after injury and continued to increase 4 days post-injury at the lesion and remote areas. Double staining revealed that most P2X(4)R(+) cells coexpressed ED-1, a marker for reactive microglia/macrophages, but not nestin or W3/13. Our data suggest that P2X(4)R expression defines a Subtype of activated microglia/macrophages involved in the early processes following traumatic brain injury. (c) 2005 Elsevier Inc. All rights reserved.

C1 Univ Tubingen, Inst Brain Res, D-72076 Tubingen, Germany.

Synovo GmbH, D-72076 Tubingen, Germany.

C3 Eberhard Karls University of Tubingen; Synovo GmbH

RP Zhang, ZR (corresponding author), Univ Tubingen, Inst Brain Res, Calwer Str 3, D-72076 Tubingen, Germany.

EM zhangzhiren@yahoo.com

RI Zhang, Zhiren/O-1012-2019

OI Zhang, Zhiren/0000-0002-5238-2835; Burnet, Michael/0000-0003-4311-5441

CR BO XN, 1995, FEBS LETT, V375, P129, DOI 10.1016/0014-5793(95)01203-Q

Braun N, 1998, J NEUROSCI, V18, P4891

Brough D, 2002, MOL CELL NEUROSCI, V19, P272, DOI 10.1006/mcne.2001.1054

Burnstock G, 2004, CURR TOP MED CHEM, V4, P793, DOI 10.2174/1568026043451014

Burnstock G, 2003, CURR TOP MEMBR, V54, P307, DOI 10.1016/S1063-5823(03)01010-X

BURNSTOCK G, 2004, INT REV CYTOL, V240, P304

Cavaliere F, 2003, NEUROSCIENCE, V120, P85, DOI 10.1016/S0306-4522(03)00228-8

DUBYAK GR, 1993, AM J PHYSIOL, V265, pC577, DOI 10.1152/ajpcell.1993.265.3.C577

EDWARDS FA, 1993, FEBS LETT, V325, P86, DOI 10.1016/0014-5793(93)81419-Z

FEENEY DM, 1981, BRAIN RES, V211, P67, DOI 10.1016/0006-8993(81)90067-6

Ferrari D, 1997, J EXP MED, V185, P579, DOI 10.1084/jem.185.3.579

Gendron FP, 2003, J NEUROCHEM, V87, P344, DOI 10.1046/j.1471-4159.2003.01995.x

Golarai G, 2001, J NEUROSCI, V21, P8523, DOI 10.1523/JNEUROSCI.21-21-08523.2001

GREGERSEN R, 2002, J CEREB BLOOD FLOW M, V20, P53

Guo LH, 2005, NEUROSCIENCE, V134, P199, DOI 10.1016/j.neuroscience.2005.04.026

Guo LH, 2005, J NEUROIMMUNOL, V163, P120, DOI 10.1016/j.jneuroim.2005.03.007

Guo LH, 2004, J NEUROIMMUNOL, V152, P67, DOI 10.1016/j.jneuroim.2004.04.005

Kennedy C, 2000, J AUTONOM NERV SYST, V81, P158, DOI 10.1016/S0165-1838(00)00133-8

Kreutzberg GW, 1996, TRENDS NEUROSCI, V19, P312, DOI 10.1016/0166-2236(96)10049-7

Ladeby R, 2005, BRAIN RES REV, V48, P196, DOI 10.1016/j.brainresrev.2004.12.009

Ladeby R, 2005, GLIA, V50, P121, DOI 10.1002/glia.20159

Lehrmann E, 1997, J COMP NEUROL, V386, P461

McIntosh TK, 1996, LAB INVEST, V74, P315

McIntosh TK, 1998, J NEUROTRAUM, V15, P731, DOI 10.1089/neu.1998.15.731

Nieber K, 1999, PROG BRAIN RES, V120, P287

Nörenberg W, 1999, PROG BRAIN RES, V120, P209

North RA, 2002, PHYSIOL REV, V82, P1013, DOI 10.1152/physrev.00015.2002

Raivich G, 1999, BRAIN RES REV, V30, P77, DOI 10.1016/S0165-0173(99)00007-7

Robson SC, 1997, J EXP MED, V185, P153, DOI 10.1084/jem.185.1.153

Rothermel Annette L., 2004, BMC Immunology, V5, P1

Ryu JK, 2002, NEUROREPORT, V13, P1611, DOI 10.1097/00001756-200209160-00008

Schwab JM, 2005, J NEUROIMMUNOL, V163, P185, DOI 10.1016/j.jneuroim.2005.02.016

Soto F, 1996, P NATL ACAD SCI USA, V93, P3684, DOI 10.1073/pnas.93.8.3684

Tsuda M, 2003, NATURE, V424, P778, DOI 10.1038/nature01786

Tsuda M, 2005, TRENDS NEUROSCI, V28, P101, DOI 10.1016/j.tins.2004.12.002

WIERASZKO A, 1989, BRAIN RES, V485, P244, DOI 10.1016/0006-8993(89)90567-2

Yamamoto K, 2000, AM J PHYSIOL-HEART C, V279, pH285, DOI 10.1152/ajpheart.2000.279.1.H285

Zimmermann H, 1996, PROG NEUROBIOL, V49, P589, DOI 10.1016/0301-0082(96)00026-3

NR 38

TC 35

Z9 42

U1 0

U2 1

PU ACADEMIC PRESS INC ELSEVIER SCIENCE

PI SAN DIEGO

PA 525 B ST, STE 1900, SAN DIEGO, CA 92101-4495 USA

SN 0014-4886

EI 1090-2430

J9 EXP NEUROL

JI Exp. Neurol.

PD JAN

PY 2006

VL 197

IS 1

BP 252

EP 257

DI 10.1016/j.expneurol.2005.09.015

PG 6

WC Neurosciences

WE Science Citation Index Expanded (SCI-EXPANDED)

SC Neurosciences & Neurology

GA 001LI

UT WOS:000234534200026

PM 16259982

DA 2024-03-03

ER

PT J

AU Zhang, Z

Artelt, M

Burnet, M

Trautmann, K

Schluesener, HJ

AF Zhang, Z.

Artelt, M.

Burnet, M.

Trautmann, K.

Schluesener, H. J.

TI Early infiltration of CD8<SUP>+</SUP> macrophages/microglia to lesions

of rat traumatic brain injury

SO NEUROSCIENCE

LA English

DT Article

DE traumatic brain injury; microglia; macrophages; weight-drop model;

endothelial monocyte-activating polypeptide II

ID EXPERIMENTAL AUTOIMMUNE ENCEPHALOMYELITIS; HEMATOGENOUS MACROPHAGES;

P2X(4) RECEPTOR; EMAP-II; EXPRESSION; MICROGLIA; MONOCYTES; ACTIVATION;

ACCUMULATION; RECRUITMENT

AB Local inflammatory responses play an important role in mediating secondary tissue damage in traumatic brain injury. Characterization of leukocytic subpopulations contributing to the early infiltration of the damaged tissue might aid in further understanding of lesion development and contribute to definition of cellular targets for selective immunotherapy. In a rat traumatic brain injury model, significant CD8(+) cell accumulation was observed 3 days post-injury. The CD8(+) cells were strictly distributed to the pannecrotic areas and around the pannecrotic perimeter. The morphology, time course of accumulation and distribution of CD8(+) cells were similar to that of reactive ED1(+) and enclothelial monocyteactivating polypepticle II+ microglia/macrophages, but different from W3/13(+) T cells. Further double-labeling experiments confirmed that the major cellular sources of CD8 were reactive macrophages/microglia. Both the location of these CD8(+) macrophages/microglia to the border of the pannecrosis and their co-expression of endothelial monocyte-activating polypeptide II and P2X(4) receptor suggest they might have a central role in lesion development and might thus be candidates fordevelopment of immunotherapeutic, anti-inflammatory strategies. (c) 2006 IBRO. Published by Elsevier Ltd. All rights reserved.

C1 Univ Tubingen, Inst Brain Res, D-72076 Tubingen, Germany.

Synovo GmbH, D-72076 Tubingen, Germany.

C3 Eberhard Karls University of Tubingen; Synovo GmbH

RP Zhang, Z (corresponding author), Univ Tubingen, Inst Brain Res, Calwer Str 3, D-72076 Tubingen, Germany.

EM zhangzhiren@yahoo.com

RI Burnet, Michael/AAB-2958-2019; Zhang, Zhiren/O-1012-2019

OI Zhang, Zhiren/0000-0002-5238-2835; Burnet, Michael/0000-0003-4311-5441

CR AIHARA N, 1995, J NEUROTRAUM, V12, P53, DOI 10.1089/neu.1995.12.53

Brabeck C, 2002, EXP NEUROL, V177, P341, DOI 10.1006/exnr.2002.7985

Cavaliere F, 2003, NEUROSCIENCE, V120, P85, DOI 10.1016/S0306-4522(03)00228-8

CLARK RSB, 1994, J NEUROTRAUM, V11, P499, DOI 10.1089/neu.1994.11.499

DAMOISEAUX JGMC, 1994, IMMUNOLOGY, V83, P140

DAVID S, 1990, NEURON, V5, P463, DOI 10.1016/0896-6273(90)90085-T

FEENEY DM, 1981, BRAIN RES, V211, P67, DOI 10.1016/0006-8993(81)90067-6

Golarai G, 2001, J NEUROSCI, V21, P8523, DOI 10.1523/JNEUROSCI.21-21-08523.2001

Guo LH, 2005, NEUROSCIENCE, V134, P199, DOI 10.1016/j.neuroscience.2005.04.026

Hirji N, 1997, J IMMUNOL, V158, P1833

Hirji N, 1998, J IMMUNOL, V160, P6004

Hirji NS, 1999, INT ARCH ALLERGY IMM, V118, P180, DOI 10.1159/000024060

HOLMIN S, 1995, NEUROSCI LETT, V194, P97, DOI 10.1016/0304-3940(95)11737-H

Jander S, 2001, BRAIN PATHOL, V11, P27

KAFFENBERGER W, 1987, J LEUKOCYTE BIOL, V42, P181, DOI 10.1002/jlb.42.3.181

Kato H, 2000, BRAIN PATHOL, V10, P137

Kempermann G, 2003, SCIENCE, V302, P1689, DOI 10.1126/science.1092864

Kostulas N, 2002, STROKE, V33, P1129, DOI 10.1161/hs0402.105379

Kreutzberg GW, 1996, TRENDS NEUROSCI, V19, P312, DOI 10.1016/0166-2236(96)10049-7

Kubes P, 2000, BRAIN PATHOL, V10, P127

Ladeby R, 2005, BRAIN RES REV, V48, P196, DOI 10.1016/j.brainresrev.2004.12.009

Ladeby R, 2005, GLIA, V50, P121, DOI 10.1002/glia.20159

Lehrmann E, 1997, J COMP NEUROL, V386, P461

Lin TJ, 2000, J IMMUNOL, V164, P1783, DOI 10.4049/jimmunol.164.4.1783

MCCALL MN, 1992, IMMUNOLOGY, V76, P310

McIntosh TK, 1996, LAB INVEST, V74, P315

McIntosh TK, 1998, J NEUROTRAUM, V15, P731, DOI 10.1089/neu.1998.15.731

MorgantiKossman MC, 1997, MOL PSYCHIATR, V2, P133, DOI 10.1038/sj.mp.4000227

Mueller CA, 2003, J NEUROTRAUM, V20, P1007, DOI 10.1089/089771503770195858

Mueller CA, 2003, J NEUROIMMUNOL, V135, P1, DOI 10.1016/S0165-5728(02)00427-7

North RA, 2002, PHYSIOL REV, V82, P1013, DOI 10.1152/physrev.00015.2002

Popovich PG, 2003, EXP NEUROL, V182, P275, DOI 10.1016/S0014-4886(03)00120-1

Raivich G, 1999, BRAIN RES REV, V30, P77, DOI 10.1016/S0165-0173(99)00007-7

Schluesener HJ, 1997, GLIA, V20, P365, DOI 10.1002/(SICI)1098-1136(199708)20:4<365::AID-GLIA8>3.0.CO;2-4

Schroeter M, 2003, AM J PATHOL, V163, P1517, DOI 10.1016/S0002-9440(10)63508-0

Schroeter M, 2001, ACTA NEUROPATHOL, V101, P440

Schwab JM, 2005, J NEUROIMMUNOL, V163, P185, DOI 10.1016/j.jneuroim.2005.02.016

Scriba A, 1997, J LEUKOCYTE BIOL, V62, P741, DOI 10.1002/jlb.62.6.741

Scriba A, 1998, SCAND J IMMUNOL, V47, P332

Stoll G, 1999, PROG NEUROBIOL, V58, P233, DOI 10.1016/S0301-0082(98)00083-5

Streit WJ, 2002, GLIA, V40, P133, DOI 10.1002/glia.10154

Tam FWK, 1999, NEPHROL DIAL TRANSPL, V14, P1658, DOI 10.1093/ndt/14.7.1658

TORRESNAGEL N, 1992, EUR J IMMUNOL, V22, P2841, DOI 10.1002/eji.1830221113

WALKER DG, 1998, CONTEMP NEUROSCI, P61

Weissenböck H, 2000, BRAIN PATHOL, V10, P260, DOI 10.1111/j.1750-3639.2000.tb00259.x

Wu YP, 2001, J NEUROPATH EXP NEUR, V60, P1062, DOI 10.1093/jnen/60.11.1062

Zhang ZR, 2006, EXP NEUROL, V197, P252, DOI 10.1016/j.expneurol.2005.09.015

NR 47

TC 47

Z9 55

U1 0

U2 3

PU PERGAMON-ELSEVIER SCIENCE LTD

PI OXFORD

PA THE BOULEVARD, LANGFORD LANE, KIDLINGTON, OXFORD OX5 1GB, ENGLAND

SN 0306-4522

EI 1873-7544

J9 NEUROSCIENCE

JI Neuroscience

PY 2006

VL 141

IS 2

BP 637

EP 644

DI 10.1016/j.neuroscience.2006.04.027

PG 8

WC Neurosciences

WE Science Citation Index Expanded (SCI-EXPANDED)

SC Neurosciences & Neurology

GA 072JA

UT WOS:000239668500008

PM 16725271

DA 2024-03-03

ER

PT J

AU Lünemann, A

Ullrich, O

Diestel, A

Jöns, T

Ninnemann, O

Kovac, A

Pohl, EE

Hass, R

Nitsch, R

Hendrix, S

AF Lünemann, A

Ullrich, O

Diestel, A

Jöns, T

Ninnemann, O

Kovac, A

Pohl, EE

Hass, R

Nitsch, R

Hendrix, S

TI Macrophage/microglia activation factor expression is restricted to

lesion-associated microglial cells after brain trauma

SO GLIA

LA English

DT Article

DE entorhinal cortex lesion; deafferentiation; CD11b; 1B4

ID ENTORHINAL CORTEX LESION; DENTATE GYRUS; MULTIPLE-SCLEROSIS; ATHYMIC

MICE; INJURY; CNS; IDENTIFICATION; MACROPHAGES; ASTROCYTES; CLONING

AB After traumatic brain lesion, microglial cells are rapidly activated, migrate toward the sites of injury, and cause secondary damage that accounts for most of the loss of brain function. In the present study, we have characterized a new macrophage/microglia activation factor (MA-F). Using the monocytic cell line U937, we were able to demonstrate that MAF is upregulated after TPA-induced differentiation into macrophages. We have generated a specific antibody against MAY In BV-2 microglial cells, MAF is partially co-localized with 1134, a classical microglial marker. In addition, we have analyzed the in vivo expression patterns of MAF after entorhinal cortex lesion. We were able to show a substantial upregulation of MAF on selected CD11b(+) and IB4(+) macrophages/microglial cells in the deafferented hippocampus and in the perilesional region, while no MAF expression was detectable on the contralateral side. Confocal microscopy revealed a lysosome-like expression pattern in BV-2 cells, as well as in ECL-associated macrophages/microglial cells in vivo. Furthermore, we were able to demonstrate that U937 cells with downregulated MAF converted slower and to a significantly reduced extent to the macrophageal phenotype after TPA treatment. In addition, MAF downregulation in BV-2 microglial cells substantially reduced the phagocytotic uptake of dextran beads. Our data indicate that MAF is expressed in selected macrophages/microglial cells around the lesion and in the degenerating hippocampus after ECL. Furthermore, MAF expression in monocytic cells seems to play a functional role in the differentiation to a phagocytosing phenotype and may be, at least partially, required for phagocytotic activity, specifically in lesioned tissue after brain trauma. (c) 2005 Wiley-Liss, Inc.

C1 Humboldt Univ, Charite Univ Med Berlin, Inst Cell Biol & Neurobiol, Ctr Anat, D-10098 Berlin, Germany.

Otto Von Guericke Univ, Inst Immunol, Dept Mol Immunol & Neuroimmunol, Magdeburg, Germany.

Humboldt Univ, Charite Univ Med Berlin, Ctr Anat, Inst Electron Microscope & Mol Neuroanat, D-10098 Berlin, Germany.

Hannover Med Sch, Dept Biochem & Tumor Biol, Clin Obstet & Gynecol, D-3000 Hannover, Germany.

C3 Free University of Berlin; Humboldt University of Berlin; Charite

Universitatsmedizin Berlin; Otto von Guericke University; Free

University of Berlin; Humboldt University of Berlin; Charite

Universitatsmedizin Berlin; Hannover Medical School

RP Nitsch, R (corresponding author), Humboldt Univ, Charite Univ Med Berlin, Inst Cell Biol & Neurobiol, Ctr Anat, Schumannstr 20-21, D-10098 Berlin, Germany.

EM robert.nitsch@charite.de

RI Hendrix, Sven/F-4059-2010; Pohl, Elena E./A-4025-2009; Hass,

Ralf/F-3197-2012; Nitsch, Robert/AAI-9129-2021

OI Hendrix, Sven/0000-0003-2344-7369; Pohl, Elena E./0000-0002-0604-5950;

Hass, Ralf/0000-0002-2481-7547;

CR BAIDA GE, 1995, BBA-GENE STRUCT EXPR, V1264, P151, DOI 10.1016/0167-4781(95)00150-F

Bechmann I, 1997, GLIA, V20, P145, DOI 10.1002/(SICI)1098-1136(199706)20:2<145::AID-GLIA6>3.0.CO;2-8

Bechmann I, 2000, ANN NY ACAD SCI, V911, P192

BOCCHINI V, 1992, J NEUROSCI RES, V31, P616, DOI 10.1002/jnr.490310405

Bräuer AU, 2004, FEBS LETT, V563, P41, DOI 10.1016/S0014-5793(04)00244-3

DRENCKHAHN D, 1993, METHOD CELL BIOL, V37, P7, DOI 10.1016/S0091-679X(08)60242-3

Eyüpoglu IY, 2003, FASEB J, V17, P1110, DOI 10.1096/fj.02-0825fje

Frotscher M, 1997, TRENDS NEUROSCI, V20, P218, DOI 10.1016/S0166-2236(96)01018-1

GIULIAN D, 1989, J NEUROSCI, V9, P4416

Guillemin GJ, 2004, J LEUKOCYTE BIOL, V75, P388, DOI 10.1189/jlb.0303114

Hailer NP, 1999, EUR J NEUROSCI, V11, P3359, DOI 10.1046/j.1460-9568.1999.00808.x

HASS R, 1989, EUR J CELL BIOL, V48, P282

HTAIN WW, 1994, GLIA, V12, P44, DOI 10.1002/glia.440120106

Htain WW, 1997, BRAIN RES, V755, P63, DOI 10.1016/S0006-8993(97)00108-X

Kreutzberg GW, 1996, TRENDS NEUROSCI, V19, P312, DOI 10.1016/0166-2236(96)10049-7

Kwidzinski E, 2003, J NEURAL TRANSM-SUPP, P29, DOI 10.1007/978-3-7091-0643-3_2

Lehnardt S, 2002, J NEUROSCI, V22, P2478, DOI 10.1523/JNEUROSCI.22-07-02478.2002

LYNCH G, 1974, BRAIN RES, V69, P1, DOI 10.1016/0006-8993(74)90365-5

Mabuchi T, 2000, STROKE, V31, P1735, DOI 10.1161/01.STR.31.7.1735

Prudovsky I, 2002, EUR J CELL BIOL, V81, P36, DOI 10.1078/0171-9335-00219

Raivich G, 2004, BRAIN RES REV, V46, P261, DOI 10.1016/j.brainresrev.2004.06.006

Rehli M, 1995, BIOCHEM BIOPH RES CO, V217, P661, DOI 10.1006/bbrc.1995.2825

SCHOEN SW, 1994, EXP NEUROL, V127, P106, DOI 10.1006/exnr.1994.1084

Stoll G, 1998, PROG NEUROBIOL, V56, P149, DOI 10.1016/S0301-0082(98)00034-3

Streit WJ, 2000, TOXICOL PATHOL, V28, P28, DOI 10.1177/019262330002800104

Tikka TM, 2001, J IMMUNOL, V166, P7527, DOI 10.4049/jimmunol.166.12.7527

Ullrich O, 2001, NAT CELL BIOL, V3, P1035, DOI 10.1038/ncb1201-1035

VARTANIAN T, 1995, MOL MED, V1, P732, DOI 10.1007/BF03401888

NR 28

TC 18

Z9 19

U1 0

U2 10

PU WILEY

PI HOBOKEN

PA 111 RIVER ST, HOBOKEN 07030-5774, NJ USA

SN 0894-1491

EI 1098-1136

J9 GLIA

JI Glia

PD MAR

PY 2006

VL 53

IS 4

BP 412

EP 419

DI 10.1002/glia.20301

PG 8

WC Neurosciences

WE Science Citation Index Expanded (SCI-EXPANDED)

SC Neurosciences & Neurology

GA 020CV

UT WOS:000235886400009

PM 16342171

DA 2024-03-03

ER

PT J

AU Vallès, A

Grijpink-Ongering, L

de Bree, FM

Tuinstra, T

Ronken, E

AF Vallès, A

Grijpink-Ongering, L

de Bree, FM

Tuinstra, T

Ronken, E

TI Differential regulation of the CXCR2 chemokine network in rat brain

trauma:: Implications for neuroimmune interactions and neuronal survival

SO NEUROBIOLOGY OF DISEASE

LA English

DT Article

DE brain injury; CXCL1; CINC-1; KC; CXCL2; MIP-2; CXCR2; leukocytes;

microglia; neuroprotection

ID MACROPHAGE-INFLAMMATORY PROTEIN-2; CONTROLLED CORTICAL IMPACT;

CENTRAL-NERVOUS-SYSTEM; MARROW-DERIVED CELLS; BONE-MARROW; MICROGLIAL

CELLS; CONSTITUTIVE EXPRESSION; NONPEPTIDE ANTAGONIST; INTERNATIONAL

UNION; IDENTIFICATION

AB Chemokine receptors represent promising targets to attenuate inflammatory responses and subsequent secondary damage after brain injury. We studied the response of the chemokines CXCL1/CINC-1 and CXCL2/MIP-2 and their receptors CXCR1 and CXCR2 after controlled cortical impact injury in adult rats. Rapid upregulation of CXCL1/CINC-1 and CXCL2/MIP-2, followed by CXCR2 (but not CXCR1), was observed after injury. Constitutive neuronal CXCR2 immunoreactivity was detected in several brain areas, which rapidly but transiently downregulated upon trauma. A second CXCR2-positive compartment, mainly colocalized with the activated microglia/macrophage marker ED1, was detected rapidly after injury in the ipsilateral cortex, progressively emerging into deeper areas of the brain later in time. It is proposed that CXCR2 has a dual role after brain injury: (i) homologous neuronal CXCR2 downregulation would render neurons more vulnerable to injury, whereas (ii) chemotaxis and subsequent differentiation of blood-borne cells into a microglial-like phenotype would be promoted by the same receptor. (c) 2005 Elsevier Inc. All rights reserved.

C1 Solvay Pharmaceut Res Labs, NL-1381 CP Weesp, Netherlands.

Netherlands Inst Brain Res, NL-1105 AZ Amsterdam, Netherlands.

C3 Solvay SA; Royal Netherlands Academy of Arts & Sciences; Netherlands

Institute for Neuroscience (NIN-KNAW)

RP Ronken, E (corresponding author), Solvay Pharmaceut Res Labs, CJ Van Houtenlaan 36, NL-1381 CP Weesp, Netherlands.

EM eric.ronken@solvay.com

OI Valles, Astrid/0000-0002-9377-2880

CR Ambrosini E, 2004, NEUROCHEM RES, V29, P1017, DOI 10.1023/B:NERE.0000021246.96864.89

Auten RL, 2001, J PHARMACOL EXP THER, V299, P90

Bechmann I, 2005, FASEB J, V19, P647, DOI 10.1096/fj.04-2599fje

BERRY M, 1999, CNS INJURIES CELLULA, P1

Catusse J, 2003, BIOCHEM PHARMACOL, V65, P813, DOI 10.1016/S0006-2952(02)01619-2

Danik M, 2003, J NEUROSCI RES, V74, P286, DOI 10.1002/jnr.10744

Dunstan CAN, 1996, J BIOL CHEM, V271, P32770, DOI 10.1074/jbc.271.51.32770

Enriquez P, 2004, CURR PHARM DESIGN, V10, P2131

Eyüpoglu IY, 2003, FASEB J, V17, P1110, DOI 10.1096/fj.02-0825fje

Fan GH, 2001, BIOCHEMISTRY-US, V40, P791, DOI 10.1021/bi001661b

FEENEY DM, 1981, BRAIN RES, V211, P67, DOI 10.1016/0006-8993(81)90067-6

Filipovic R, 2003, DEV NEUROSCI-BASEL, V25, P279, DOI 10.1159/000072275

Fischer FR, 2000, J NEUROIMMUNOL, V110, P195, DOI 10.1016/S0165-5728(00)00351-9

Flynn G, 2003, J NEUROIMMUNOL, V136, P84, DOI 10.1016/S0165-5728(03)00009-2

GIULIAN D, 1989, J NEUROSCI, V9, P4416

Hall ED, 2005, J NEUROTRAUM, V22, P252, DOI 10.1089/neu.2005.22.252

Horuk R, 1997, J IMMUNOL, V158, P2882

Imai Y, 1996, BIOCHEM BIOPH RES CO, V224, P855, DOI 10.1006/bbrc.1996.1112

Janabi N, 1999, J IMMUNOL, V162, P1701

Kielian T, 2001, J IMMUNOL, V166, P4634, DOI 10.4049/jimmunol.166.7.4634

Koennecke LA, 1999, J NEUROCHEM, V73, P770, DOI 10.1046/j.1471-4159.1999.0730770.x

Lenzlinger PM, 2001, MOL NEUROBIOL, V24, P169

Liberto CM, 2004, J NEUROCHEM, V89, P1092, DOI 10.1111/j.1471-4159.2004.02420.x

Limatola C, 2000, P NATL ACAD SCI USA, V97, P6197, DOI 10.1073/pnas.090105997

Luo QW, 2005, MOL PHARMACOL, V68, P528, DOI 10.1124/mol.105.011197

Luo Y, 2000, J IMMUNOL, V165, P4015, DOI 10.4049/jimmunol.165.7.4015

Malm TM, 2005, NEUROBIOL DIS, V18, P134, DOI 10.1016/j.nbd.2004.09.009

MATSUO Y, 1995, J CEREBR BLOOD F MET, V15, P941, DOI 10.1038/jcbfm.1995.119

Matzer SP, 2004, IMMUNOBIOLOGY, V209, P225, DOI 10.1016/j.imbio.2004.02.009

Matzer SP, 2004, INT IMMUNOL, V16, P1675, DOI 10.1093/intimm/dxh169

Matzer SP, 2001, J IMMUNOL, V167, P4635, DOI 10.4049/jimmunol.167.8.4635

McGraw J, 2001, J NEUROSCI RES, V63, P109, DOI 10.1002/1097-4547(20010115)63:2<109::AID-JNR1002>3.0.CO;2-J

Murphy PM, 2000, PHARMACOL REV, V52, P145

Murphy PM, 2002, PHARMACOL REV, V54, P227, DOI 10.1124/pr.54.2.227

Nguyen D, 2001, DEV BRAIN RES, V128, P77, DOI 10.1016/S0165-3806(01)00128-6

Omari KM, 2006, GLIA, V53, P24, DOI 10.1002/glia.20246

Omari KM, 2005, BRAIN, V128, P1003, DOI 10.1093/brain/awh479

Oynebråten I, 2004, BLOOD, V104, P314, DOI 10.1182/blood-2003-08-2891

PALMER AM, 1993, J NEUROCHEM, V61, P2015, DOI 10.1111/j.1471-4159.1993.tb07437.x

Paxinos G, 1998, RAT BRAIN IN STEREOTAXIC COORDINATES, FOURTH ED., pix

Podolin PL, 2002, J IMMUNOL, V169, P6435, DOI 10.4049/jimmunol.169.11.6435

Popivanova BK, 2003, BRAIN RES, V970, P195, DOI 10.1016/S0006-8993(03)02343-6

Priller J, 2001, NAT MED, V7, P1356, DOI 10.1038/nm1201-1356

Puma C, 2001, J NEUROCHEM, V78, P960, DOI 10.1046/j.1471-4159.2001.00469.x

Raivich G, 1999, BRAIN RES REV, V30, P77, DOI 10.1016/S0165-0173(99)00007-7

Ransohoff RM, 1998, TRENDS NEUROSCI, V21, P154, DOI 10.1016/S0166-2236(97)01198-3

Rose JJ, 2004, J BIOL CHEM, V279, P24372, DOI 10.1074/jbc.M401364200

SCHALL TJ, 1994, CURR OPIN IMMUNOL, V6, P865, DOI 10.1016/0952-7915(94)90006-X

Simard AR, 2004, FASEB J, V18, P998, DOI 10.1096/fj.04-1517fje

Streit WJ, 2002, GLIA, V40, P133, DOI 10.1002/glia.10154

Tani M, 1996, J CLIN INVEST, V98, P529, DOI 10.1172/JCI118821

TEASDALE GM, 1997, HEAD INJURY, P423

Traves SL, 2004, J LEUKOCYTE BIOL, V76, P441, DOI 10.1189/jlb.1003495

Vallières L, 2003, J NEUROSCI, V23, P5197

Vink R, 2002, EXPERT OPIN INV DRUG, V11, P1375, DOI 10.1517/13543784.11.10.1375

Watson K, 2005, MOL PHARMACOL, V67, P757, DOI 10.1124/mol.104.004812

White JR, 1998, J BIOL CHEM, V273, P10095, DOI 10.1074/jbc.273.17.10095

Xia MQ, 1997, AM J PATHOL, V150, P1267

Zhai QW, 2004, GLIA, V48, P327, DOI 10.1002/glia.20087

NR 59

TC 76

Z9 81

U1 0

U2 12

PU ACADEMIC PRESS INC ELSEVIER SCIENCE

PI SAN DIEGO

PA 525 B ST, STE 1900, SAN DIEGO, CA 92101-4495 USA

SN 0969-9961

EI 1095-953X

J9 NEUROBIOL DIS

JI Neurobiol. Dis.

PD MAY

PY 2006

VL 22

IS 2

BP 312

EP 322

DI 10.1016/j.nbd.2005.11.015

PG 11

WC Neurosciences

WE Science Citation Index Expanded (SCI-EXPANDED)

SC Neurosciences & Neurology

GA 040KS

UT WOS:000237378000011

PM 16472549

DA 2024-03-03

ER

PT J

AU Igarashi, T

Potts, MB

Noble-Haeusslein, LJ

AF Igarashi, Takuji

Potts, Matthew B.

Noble-Haeusslein, Linda J.

TI Injury severity determines Purkinje cell loss and microglial activation

in the cerebellum after cortical contusion injury

SO EXPERIMENTAL NEUROLOGY

LA English

DT Article

DE traumatic brain injury; controlled cortical impact; neuronal loss;

cerebellum; activated microglia; Purkinje cells

ID TRAUMATIC BRAIN-INJURY; FLUID PERCUSSION INJURY; MORRIS WATER MAZE;

IMPACT INJURY; COGNITIVE DEFICITS; HEAD-INJURY; NEURONAL INJURY;

TIME-COURSE; RAT; MODEL

AB Clinical evidence suggests that the cerebellum is damaged after traumatic brain injury (TBI) and experimental studies have validated these observations. We have previously shown cerebellar vulnerability, as demonstrated by Purkinje cell loss and microglial activation, after fluid percussion brain injury. In this study, we examine the effect of graded controlled cortical impact (CCI) injury on the cerebellum in the context of physiologic and anatomical parameters that have been shown by others to be sensitive to injury severity. Adult male rats received mild, moderate, or severe CCI and were euthanized 7 days later. We first validated the severity of the initial injury using physiologic criteria, including apnea and blood pressure, during the immediate postinjury period. Increasing injury severity was associated with an increased incidence of apnea and higher mortality. Severe injury also induced transient hypertension followed by hypotension, while lower grade injuries produced an immediate and sustained hypotension. We next evaluated the pattern of subcortical neuronal loss in response to graded injuries. There was significant neuronal loss in the ipsilateral cortex, hippocampal CA2/CA3, and laterodorsal thalamus that was injury severity-dependent and that paralleled microglial activation. Similarly, there was a distinctive pattern of Purkinje cell loss and microglial activation in the cerebellar vermis that varied with injury severity. Together, these findings emphasize the vulnerability of the cerebellum to TBI. That a selective pattern of Purkinje cell loss occurs regardless of the type of injury suggests a generalized response that is a likely determinant of recovery and a target for therapeutic intervention. (c) 2006 Elsevier Inc. All rights reserved.

C1 Univ Calif San Francisco, Dept Neurol Surg, San Francisco, CA 94143 USA.

C3 University of California System; University of California San Francisco

RP Noble-Haeusslein, LJ (corresponding author), Univ Calif San Francisco, Dept Neurol Surg, 521 Parnassus Ave,Room C-224, San Francisco, CA 94143 USA.

EM linda.noble@ucsf.edu

OI Noble-Haeusslein, Linda/0000-0002-2653-7047

FU NINDS NIH HHS [NS050159] Funding Source: Medline

CR Ai JL, 2004, PHARMACOLOGY, V71, P192, DOI 10.1159/000078085

Ai JL, 2002, NEUROSCI LETT, V332, P155, DOI 10.1016/S0304-3940(02)00945-X

Alavi A, 1997, J NUCL MED, V38, P1717

Alessandri B, 2003, J NEUROTRAUM, V20, P1293, DOI 10.1089/089771503322686094

Baldwin SA, 1997, J NEUROTRAUM, V14, P385, DOI 10.1089/neu.1997.14.385

Basford JR, 2003, ARCH PHYS MED REHAB, V84, P343, DOI 10.1053/apmr.2003.50034

BIARY N, 1989, NEUROLOGY, V39, P103, DOI 10.1212/WNL.39.1.103

Chen S, 2003, EXP NEUROL, V182, P87, DOI 10.1016/S0014-4886(03)00002-5

Cherian L, 2000, J NEUROPHYSIOL, V83, P2171, DOI 10.1152/jn.2000.83.4.2171

CHERIAN L, 1994, J NEUROTRAUM, V11, P573, DOI 10.1089/neu.1994.11.573

CLARK RSB, 1994, J NEUROTRAUM, V11, P499, DOI 10.1089/neu.1994.11.499

Colicos MA, 1996, BRAIN RES, V739, P111, DOI 10.1016/S0006-8993(96)00819-0

Conti AC, 1998, J NEUROSCI, V18, P5663

CORTEZ SC, 1989, BRAIN RES, V482, P271, DOI 10.1016/0006-8993(89)91190-6

D'Hooge R, 2001, BRAIN RES REV, V36, P60, DOI 10.1016/S0165-0173(01)00067-4

DAUM I, 1995, BEHAV BRAIN RES, V67, P201, DOI 10.1016/0166-4328(94)00144-5

Di X, 1999, J NEUROTRAUM, V16, P195, DOI 10.1089/neu.1999.16.195

Ding YC, 2001, NEUROL RES, V23, P193, DOI 10.1179/016164101101198334

DIXON CE, 1987, J NEUROSURG, V67, P110, DOI 10.3171/jns.1987.67.1.0110

DIXON CE, 1991, J NEUROSCI METH, V39, P253

FODA MAA, 1994, J NEUROSURG, V80, P301, DOI 10.3171/jns.1994.80.2.0301

Fox GB, 1998, J NEUROTRAUM, V15, P599, DOI 10.1089/neu.1998.15.599

Fujimoto ST, 2004, NEUROSCI BIOBEHAV R, V28, P365, DOI 10.1016/j.neubiorev.2004.06.002

Fukuda K, 1996, J NEUROTRAUM, V13, P255, DOI 10.1089/neu.1996.13.255

Gale SD, 2005, J NEUROL NEUROSUR PS, V76, P984, DOI 10.1136/jnnp.2004.036210

Geddes DM, 2003, EXP NEUROL, V184, P420, DOI 10.1016/S0014-4886(03)00254-1

GOODMAN JC, 1994, J NEUROTRAUM, V11, P587, DOI 10.1089/neu.1994.11.587

Hall ED, 2005, J NEUROTRAUM, V22, P252, DOI 10.1089/neu.2005.22.252

HAMM RJ, 1992, J NEUROTRAUM, V9, P11, DOI 10.1089/neu.1992.9.11

Hicks R, 1996, ACTA NEUROPATHOL, V91, P236, DOI 10.1007/s004010050421

Hoshino S, 2003, NEUROL MED-CHIR, V43, P165, DOI 10.2176/nmc.43.165

IIZUKA H, 1990, STROKE, V21, P790, DOI 10.1161/01.STR.21.5.790

IWADATE Y, 1989, Neurosurgical Review, V12, P500, DOI 10.1007/BF01790695

Kim SU, 2005, J NEUROSCI RES, V81, P302, DOI 10.1002/jnr.20562

KOCHANEK PM, 1995, J NEUROTRAUM, V12, P1015

KRAUSS JK, 1995, NEUROL RES, V17, P409, DOI 10.1080/01616412.1995.11740353

Kuhtz-Buschbeck JP, 2003, DEV MED CHILD NEUROL, V45, P821, DOI 10.1017/S001216220300152X

Lalonde R, 2003, CEREBELLUM, V2, P300, DOI 10.1080/14734220310017456

LIGHTHALL J W, 1990, Journal of Neurotrauma, V7, P65, DOI 10.1089/neu.1990.7.65

Lighthall JW, 1988, J NEUROTRAUM, V5, P1, DOI 10.1089/neu.1988.5.1

Louis ED, 1996, ARCH NEUROL-CHICAGO, V53, P450, DOI 10.1001/archneur.1996.00550050080027

LOWENSTEIN DH, 1992, J NEUROSCI, V12, P4846

LYETH BG, 1988, BRAIN RES, V448, P88, DOI 10.1016/0006-8993(88)91104-3

Manley GT, 2006, J NEUROTRAUM, V23, P128, DOI 10.1089/neu.2006.23.128

Marín-Teva JL, 2004, NEURON, V41, P535, DOI 10.1016/S0896-6273(04)00069-8

MARMAROU A, 1994, J NEUROSURG, V80, P291, DOI 10.3171/jns.1994.80.2.0291

Matthews MA, 1998, BRAIN RES, V794, P1, DOI 10.1016/S0006-8993(98)00107-3

Mautes AEM, 1996, NEUROSCI LETT, V214, P95, DOI 10.1016/0304-3940(96)12916-5

McIntosh T K, 1987, Cent Nerv Syst Trauma, V4, P119

MCINTOSH TK, 1989, NEUROSCIENCE, V28, P233, DOI 10.1016/0306-4522(89)90247-9

Middleton FA, 1998, TRENDS NEUROSCI, V21, P367, DOI 10.1016/S0166-2236(98)01330-7

Morales DM, 2005, NEUROSCIENCE, V136, P971, DOI 10.1016/j.neuroscience.2005.08.030

MULLEN RJ, 1992, DEVELOPMENT, V116, P201

MYSIW W J, 1990, Brain Injury, V4, P247, DOI 10.3109/02699059009026174

NAKKI R, 1995, J NEUROSCI, V15, P2097, DOI 10.1523/JNEUROSCI.15-03-02097.1995

Newcomb JK, 1997, J NEUROTRAUM, V14, P369, DOI 10.1089/neu.1997.14.369

Niimura K, 1999, NEUROLOGY, V52, P792, DOI 10.1212/WNL.52.4.792

OHearn E, 1997, J NEUROSCI, V17, P8828

PALMER AM, 1993, J NEUROCHEM, V61, P2015, DOI 10.1111/j.1471-4159.1993.tb07437.x

Park E, 2006, J NEUROPATH EXP NEUR, V65, P226, DOI 10.1097/01.jnen.0000202888.29705.93

Paxinos G., 2018, RAT NERVOUS SYSTEM

Piot-Grosjean O, 2001, NEUROBIOL DIS, V8, P1082, DOI 10.1006/nbdi.2001.0450

Rapoport M, 2000, J NEUROPSYCH CLIN N, V12, P193, DOI 10.1176/appi.neuropsych.12.2.193

ROSS CA, 1990, TRENDS NEUROSCI, V13, P216, DOI 10.1016/0166-2236(90)90163-5

Sato M, 2001, BRAIN RES, V917, P45, DOI 10.1016/S0006-8993(01)02905-5

Scheff SW, 1997, J NEUROTRAUM, V14, P615, DOI 10.1089/neu.1997.14.615

SCHMIDT RH, 1993, J NEUROTRAUM, V10, P415, DOI 10.1089/neu.1993.10.415

SILVERMAN J, 1993, LAB ANIM SCI, V43, P210

SMITH DH, 1995, J NEUROTRAUM, V12, P169, DOI 10.1089/neu.1995.12.169

Soblosky JS, 1996, BEHAV BRAIN RES, V79, P79, DOI 10.1016/0166-4328(95)00264-2

Soto-Ares G, 2001, CHILD NERV SYST, V17, P263, DOI 10.1007/s003810000411

Strich S J, 1970, J Clin Pathol Suppl (R Coll Pathol), V4, P166

Tang SL, 1997, J CHART INST WATER E, V11, P14

Tang YP, 1997, J NEUROTRAUM, V14, P851, DOI 10.1089/neu.1997.14.851

Tecoult E, 2000, J NEUROSURG ANESTH, V12, P255, DOI 10.1097/00008506-200007000-00010

Thompson HJ, 2005, J NEUROTRAUM, V22, P42, DOI 10.1089/neu.2005.22.42

Toth Z, 1997, J NEUROSCI, V17, P8106

Wixson S.K., 1997, ANESTHESIA ANALGESIA

WOBER C, 1993, ARCH PHYS MED REHAB, V74, P1151

Wolf HK, 1996, J HISTOCHEM CYTOCHEM, V44, P1167, DOI 10.1177/44.10.8813082

YUAN X-Q, 1990, Journal of Neurotrauma, V7, P141, DOI 10.1089/neu.1990.7.141

Zanier ER, 2003, J NEUROTRAUM, V20, P409, DOI 10.1089/089771503765355496

ZIAI MR, 1988, J NEUROCHEM, V51, P1771, DOI 10.1111/j.1471-4159.1988.tb01158.x

ZIAI R, 1986, P NATL ACAD SCI USA, V83, P8420, DOI 10.1073/pnas.83.21.8420

NR 84

TC 53

Z9 68

U1 0

U2 9

PU ACADEMIC PRESS INC ELSEVIER SCIENCE

PI SAN DIEGO

PA 525 B ST, STE 1900, SAN DIEGO, CA 92101-4495 USA

SN 0014-4886

J9 EXP NEUROL

JI Exp. Neurol.

PD JAN

PY 2007

VL 203

IS 1

BP 258

EP 268

DI 10.1016/j.expneurol.2006.08.030

PG 11

WC Neurosciences

WE Science Citation Index Expanded (SCI-EXPANDED)

SC Neurosciences & Neurology

GA 123NU

UT WOS:000243303400028

PM 17045589

DA 2024-03-03

ER

PT J

AU Thomale, UW

Bender, M

Casalis, P

Rupprecht, S

Griebenow, M

Neumann, K

Woiciechowsky, C

Unterberg, AW

Stover, JF

AF Thomale, Ulrich W.

Bender, Marcel

Casalis, Pablo

Rupprecht, Stefan

Griebenow, Martin

Neumann, Konrad

Woiciechowsky, Christian

Unterberg, Andreas W.

Stover, John F.

TI Tacrolimus depresses local immune cell infiltration but fails to reduce

cortical contusion volume in brain-injured rats

SO IMMUNOBIOLOGY

LA English

DT Article

DE traumatic brain injury; ICAM-1; microglia; macrophages; granulocytes

ID NECROSIS-FACTOR-ALPHA; CLOSED-HEAD INJURY; FLUID PERCUSSION INJURY;

NITRIC-OXIDE SYNTHASE; SPINAL-CORD-INJURY; CEREBRAL-ISCHEMIA; NEUTROPHIL

INFILTRATION; NEUROPROTECTIVE ACTION; INFLAMMATORY RESPONSE;

MOLECULAR-MECHANISMS

AB The immunosuppressant drug tacrolimus (FK-506) failed to show an anti-edematous effect despite suppressing pro-inflammatory cytokines in cerebrospinal fluid following focal traumatic brain injury. By questioning the role of the inflammatory response as a pharmacological target, we investigated the effects of FK-506 on immune cell infiltration in brain-injured rats.

Following induction of a cortical contusion, male Sprague-Dawley rats received FK-506 or physiological saline intraperitoneally. Brains were removed at 24 h, 72 h or 7 days, respectively. Frozen brain sections (7 mu m) were stained immunohistologically for markers of endothelial activation (intercellular adhesion molecule-1-ICAM-1), neutrophil infiltration (His-48), and microglial and macrophage activation (Ox-6; ED-1), respectively. Immunopositive cells were counted microscopically. Contusion volume (CV) was quantified morphometrically 7 days after trauma.

Inflammatory response was confined to the ipsilateral cortex and hippocampal formation, predominating in the contusion and pericontusional cortex. Strongest ICAM-1 expression coincided with sustained granulocyte accumulation at 72 h which was suppressed by FK-506. Ox-6 + cells prevailing at 72 h were also significantly reduced by FK-506. ED-1 + cells reaching highest intensity at 7 days were significantly attenuated at 72 h. Cortical CV was not influenced.

FK-506 significantly decreased post-traumatic local inflammation which, however, was not associated with a reduction in cortical CV. These results question the importance of post-traumatic local immune cell infiltration in the secondary growth of a cortical contusion. (c) 2007 Elsevier GmbH. All rights reserved.

C1 Med Univ Berlin, Dept Neurosurg, Campus Virchow Med Ctr, D-13353 Berlin, Germany.

Charite, Dept Biometry & Clin Epidemiol, D-10117 Berlin, Germany.

Heidelberg Univ, Dept Neurosurg, D-69120 Heidelberg, Germany.

Univ Zurich Hosp, Div Surg Intens Care Med, CH-8091 Zurich, Switzerland.

C3 Free University of Berlin; Humboldt University of Berlin; Charite

Universitatsmedizin Berlin; Free University of Berlin; Humboldt

University of Berlin; Charite Universitatsmedizin Berlin; Ruprecht Karls

University Heidelberg; University of Zurich; University Zurich Hospital

RP Thomale, UW (corresponding author), Med Univ Berlin, Dept Neurosurg, Campus Virchow Med Ctr, D-13353 Berlin, Germany.

EM uthomale@charite.de

OI Misch, Martin/0000-0001-5283-5753

CR AIHARA N, 1995, J NEUROTRAUM, V12, P53, DOI 10.1089/neu.1995.12.53

Allan SM, 2003, PHILOS T R SOC B, V358, P1669, DOI 10.1098/rstb.2003.1358

Allan SM, 2001, NAT REV NEUROSCI, V2, P734, DOI 10.1038/35094583

Benetoli A, 2004, PHARMACOL BIOCHEM BE, V77, P607, DOI 10.1016/j.pbb.2003.12.022

Bochelen D, 1999, J PHARMACOL EXP THER, V288, P653

Butcher SP, 1997, J NEUROSCI, V17, P6939

CLARK RK, 1993, BRAIN RES BULL, V31, P565, DOI 10.1016/0361-9230(93)90124-T

CLARK RSB, 1994, J NEUROTRAUM, V11, P499, DOI 10.1089/neu.1994.11.499

Cross AK, 2001, MICROSC RES TECHNIQ, V54, P10, DOI 10.1002/jemt.1115

Darakchiev BJ, 1997, ACT NEUR S, V70, P98

DAWSON TM, 1993, P NATL ACAD SCI USA, V90, P9808, DOI 10.1073/pnas.90.21.9808

Fee D, 2003, J NEUROIMMUNOL, V136, P54, DOI 10.1016/S0165-5728(03)00008-0

Feuerstein GZ, 1997, ANN NY ACAD SCI, V825, P179, DOI 10.1111/j.1749-6632.1997.tb48428.x

Furuichi Y, 2004, BRAIN RES, V1014, P120, DOI 10.1016/j.brainres.2004.04.031

GarciaCriado FJ, 1997, TRANSPLANTATION, V64, P594, DOI 10.1097/00007890-199708270-00008

GIULIAN D, 1989, J NEUROSCI, V9, P4416

Gold BG, 1999, DRUG METAB REV, V31, P649, DOI 10.1081/DMR-100101940

Hartl R, 1997, J CEREBR BLOOD F MET, V17, P1210

Kaminska B, 2004, J CELL MOL MED, V8, P45, DOI 10.1111/j.1582-4934.2004.tb00259.x

Klettner A, 2003, CNS NEUROL DISORD-DR, V2, P153, DOI 10.2174/1568007033482878

Kubes P, 2000, BRAIN PATHOL, V10, P127

Kurz JE, 2005, BRAIN RES, V1048, P153, DOI 10.1016/j.brainres.2005.04.062

Kurz JE, 2005, J NEUROTRAUM, V22, P476, DOI 10.1089/neu.2005.22.476

Lenzlinger PM, 2001, MOL NEUROBIOL, V24, P169

Lucas SM, 2006, BRIT J PHARMACOL, V147, pS232, DOI 10.1038/sj.bjp.0706400

Madsen JR, 1998, EXP NEUROL, V154, P673, DOI 10.1006/exnr.1998.6974

Marmarou CR, 2006, EXP NEUROL, V197, P353, DOI 10.1016/j.expneurol.2005.10.003

Masri MA, 2003, MOL IMMUNOL, V39, P1073, DOI 10.1016/S0161-5890(03)00075-0

Mechoulam R, 2002, TRENDS MOL MED, V8, P58, DOI 10.1016/S1471-4914(02)02276-1

Morganti-Kossmann Maria Cristina, 2002, Curr Opin Crit Care, V8, P101

Morganti-Kossmann MC, 2001, SHOCK, V16, P165, DOI 10.1097/00024382-200116030-00001

Nottingham S, 2002, EXP NEUROL, V177, P242, DOI 10.1006/exnr.2002.7975

Paxinos G., 1996, RAT BRAIN STEREOTAXI

Price CJS, 2003, J NEUROL NEUROSUR PS, V74, P1476, DOI 10.1136/jnnp.74.11.1476

Raivich G, 1999, BRAIN RES REV, V30, P77, DOI 10.1016/S0165-0173(99)00007-7

Scheff SW, 1999, J NEUROTRAUM, V16, P783, DOI 10.1089/neu.1999.16.783

Schöning B, 2002, NEUROL SCI, V23, P211, DOI 10.1007/s100720200043

Shohami E, 1999, CYTOKINE GROWTH F R, V10, P119, DOI 10.1016/S1359-6101(99)00008-8

Shohami E, 1996, J CEREBR BLOOD F MET, V16, P378, DOI 10.1097/00004647-199605000-00004

Smith DH, 1997, J NEUROTRAUM, V14, P715, DOI 10.1089/neu.1997.14.715

Squadrito F, 1999, BRIT J PHARMACOL, V127, P498, DOI 10.1038/sj.bjp.0702528

Stahel PF, 2000, J CEREBR BLOOD F MET, V20, P369, DOI 10.1097/00004647-200002000-00019

Stover JF, 2001, J NEUROSURG, V94, P782, DOI 10.3171/jns.2001.94.5.0782

Suehiro E, 2001, EXP NEUROL, V172, P199, DOI 10.1006/exnr.2001.7765

Tamura F, 1998, J GASTROEN HEPATOL, V13, P703, DOI 10.1111/j.1440-1746.1998.tb00717.x

Tehranian R, 2002, J NEUROTRAUM, V19, P939, DOI 10.1089/089771502320317096

Thomale UW, 2006, INTENS CARE MED, V32, P149, DOI 10.1007/s00134-005-2845-4

Tsujikawa A, 1998, STROKE, V29, P1431, DOI 10.1161/01.STR.29.7.1431

Vela JM, 2002, J NEUROTRAUM, V19, P1503, DOI 10.1089/089771502320914723

Whalen MJ, 2000, CRIT CARE MED, V28, P3710, DOI 10.1097/00003246-200011000-00029

Yoshimoto T, 2002, BRAIN RES, V947, P191, DOI 10.1016/S0006-8993(02)02922-0

ZHANG J, 1995, NEUROL RES, V17, P285, DOI 10.1080/01616412.1995.11740328

NR 52

TC 16

Z9 20

U1 0

U2 0

PU ELSEVIER GMBH, URBAN & FISCHER VERLAG

PI JENA

PA OFFICE JENA, P O BOX 100537, 07705 JENA, GERMANY

SN 0171-2985

J9 IMMUNOBIOLOGY

JI Immunobiology

PY 2007

VL 212

IS 7

BP 567

EP 576

DI 10.1016/j.imbio.2007.01.007

PG 10

WC Immunology

WE Science Citation Index Expanded (SCI-EXPANDED)

SC Immunology

GA 209FV

UT WOS:000249375300004

PM 17678714

DA 2024-03-03

ER

PT J

AU Potter, EG

Cheng, Y

Knight, JB

Gordish-Dressman, H

Natale, JE

AF Potter, Emily G.

Cheng, Ying

Knight, Jay Brandon

Gordish-Dressman, Heather

Natale, Joanne E.

TI Metallothionein I and II attenuate the thalamic microglial response

following traumatic axotomy in the immature brain

SO JOURNAL OF NEUROTRAUMA

LA English

DT Article

DE inflicted childhood neurotrauma; mice; microglial activation; negative

binomial regression; secondary injury

ID FOCAL CEREBRAL-ISCHEMIA; CENTRAL-NERVOUS-SYSTEM; INFLICTED HEAD-INJURY;

CELL-DEATH; RAT-BRAIN; DOPAMINERGIC-NEURONS; CEREBROSPINAL-FLUID;

MOLECULAR-MECHANISMS; SENSORIMOTOR CORTEX; PARKINSONS-DISEASE

AB The clinical manifestations of inflicted traumatic brain injury in infancy most commonly result from intracranial hemorrhage, axonal stretch and disruption, and cerebral edema. Often hypoxia ischemia is superimposed, leading to early forebrain and later thalamic neurodegeneration. Such acute and delayed cellular injury activates microglia in the CNS. Although activated microglia provide important benefits in response to injury, microglial release of reactive oxygen species can be harmful to axotomized neurons. We have previously shown that the antioxidants metallothionein I and II (MT I & II) promote geniculocortical neuronal survival after visual cortex lesioning. The purpose of this investigation was to determine the influence of MT I & II on the density and rate of thalamic microglial activation and accumulation following in vivo axotomy. We ablated the visual cortex of 10-day-old and adult MT I & II knock out (MT-/-) and wild-type mice and then determined the density of microglia in the dorsal lateral geniculate nucleus (dLGN) over time. Compared to the wild-type strain, microglial activation occurred earlier in both young and adult MT-/- mice. Similarly, microglial density was significantly greater in young MT-/- mice 30, 36, and 48 hours after injury, and 3, 4, and 5 days after injury in MT-/- adults. In both younger and older mice, time and MT I & II deficiency each contributed significantly to greater microglial density. Only in younger mice did MT I & II expression significantly slow the rate (density X time) of microglial accumulation. These results suggest that augmentation of NIT I & II expression may provide therapeutic benefits to infants with inflicted brain injury.

C1 Childrens Natl Med Ctr, Med Genet Res Ctr, Washington, DC USA.

George Washington Univ, Inst Biomed Sci, Neurosci Program, Washington, DC 20052 USA.

Childrens Natl Med Ctr, Neurosci Res Ctr, Washington, DC 20010 USA.

C3 Children's National Health System; George Washington University;

Children's National Health System

RP Natale, JE (corresponding author), Univ Calif Davis, Med Ctr, Dept Pediat, 2516 Stockton Blvd, Sacramento, CA 95817 USA.

EM jnatale@cnmcresearch.org

OI Gordish-Dressman, Heather/0000-0002-2330-5427; Knight,

Brandon/0000-0003-4953-7194

FU NINDS NIH HHS [KO8 NS 41273] Funding Source: Medline

CR Acarin L, 1999, NEUROSCIENCE, V89, P549, DOI 10.1016/S0306-4522(98)00331-5

Acarin L, 1997, EXP NEUROL, V147, P410, DOI 10.1006/exnr.1997.6593

Agar J, 2003, AMYOTROPH LATERAL SC, V4, P232, DOI 10.1080/14660820310011278

Asanuma M, 2002, NEUROSCI LETT, V327, P61, DOI 10.1016/S0304-3940(02)00346-4

Batchelor PE, 1999, J NEUROSCI, V19, P1708

Bayir H, 2002, PEDIATR RES, V51, P571, DOI 10.1203/00006450-200205000-00005

Bell MJ, 1999, CRIT CARE MED, V27, P493, DOI 10.1097/00003246-199903000-00023

Berger RP, 2004, J NEUROTRAUM, V21, P1123

Block ML, 2004, FASEB J, V18, P1618, DOI 10.1096/fj.04-1945fje

Campagne MV, 2000, J NEUROSCI, V20, P5200, DOI 10.1523/JNEUROSCI.20-14-05200.2000

Cerutti SM, 2000, CELL BIOL INT, V24, P35, DOI 10.1006/cbir.1999.0451

Chung RS, 2004, NEUROSCIENCE, V123, P595, DOI 10.1016/j.neuroscience.2003.10.019

Dailey ME, 1999, METHODS, V18, P222, DOI 10.1006/meth.1999.0775

Dommergues MA, 2003, NEUROSCIENCE, V121, P619, DOI 10.1016/S0306-4522(03)00558-X

Ebadi M, 2005, METHOD ENZYMOL, V396, P276, DOI 10.1016/S0076-6879(05)96024-2

Ewing-Cobbs L, 2000, CHILD NERV SYST, V16, P25, DOI 10.1007/s003810050006

Fan P, 2003, J NEUROTRAUM, V20, P437, DOI 10.1089/089771503765355513

Gao HM, 2003, J NEUROSCI, V23, P6181

Geddes JF, 2001, BRAIN, V124, P1299, DOI 10.1093/brain/124.7.1299

GRUNDL PD, 1994, J NEUROTRAUM, V11, P135, DOI 10.1089/neu.1994.11.135

Hagberg H, 2004, J NEUROCHEM, V90, P1068, DOI 10.1111/j.1471-4159.2004.02547.x

He Y, 2001, BRAIN RES, V909, P187, DOI 10.1016/S0006-8993(01)02681-6

Heese K, 1998, J NEUROCHEM, V70, P699

Heppner FL, 2005, NAT MED, V11, P146, DOI 10.1038/nm1177

Hermann DM, 2000, ACTA NEUROPATHOL, V99, P147, DOI 10.1007/PL00007418

Hochman A, 2000, CELL MOL BIOL, V46, P41

Ito D, 2001, STROKE, V32, P1208, DOI 10.1161/01.STR.32.5.1208

Ito D, 1998, MOL BRAIN RES, V57, P1, DOI 10.1016/S0169-328X(98)00040-0

Jensen MB, 1999, NEUROSCIENCE, V93, P507, DOI 10.1016/S0306-4522(99)00139-6

Keenan HT, 2006, PEDIATRICS, V117, P317, DOI 10.1542/peds.2005-0979

Keenan HT, 2004, PEDIATRICS, V114, P633, DOI 10.1542/peds.2003-1020-L

Kim D, 2003, NEUROREPORT, V14, P679, DOI 10.1097/00001756-200304150-00004

Kohler LB, 2003, BRAIN RES, V992, P128, DOI 10.1016/j.brainres.2003.08.049

Kreutzberg GW, 1996, TRENDS NEUROSCI, V19, P312, DOI 10.1016/0166-2236(96)10049-7

Ladeby R, 2005, BRAIN RES REV, V48, P196, DOI 10.1016/j.brainresrev.2004.12.009

Lehnardt S, 2003, P NATL ACAD SCI USA, V100, P8514, DOI 10.1073/pnas.1432609100

LEIBBRANDT MEI, 1994, TOXICOL APPL PHARM, V124, P72, DOI 10.1006/taap.1994.1010

Lu YZ, 2005, NEUROSCI LETT, V373, P159, DOI 10.1016/j.neulet.2004.10.004

Maxwell WL, 2004, BRAIN, V127, P2470, DOI 10.1093/brain/awh294

MILLIGAN CE, 1991, J COMP NEUROL, V314, P136, DOI 10.1002/cne.903140113

Mischel RE, 1997, NEUROSCI LETT, V231, P17, DOI 10.1016/S0304-3940(97)00531-4

Morino T, 2003, NEUROSCI RES, V46, P309, DOI 10.1016/S0168-0102(03)00095-6

Muessel MJ, 2002, MOL BRAIN RES, V103, P12, DOI 10.1016/S0169-328X(02)00158-4

Münch G, 2003, EXP BRAIN RES, V150, P1, DOI 10.1007/s00221-003-1389-5

Natale JE, 2004, J NEUROSCI RES, V78, P303, DOI 10.1002/jnr.20265

Natale JE, 2002, NEUROSCIENCE, V112, P665, DOI 10.1016/S0306-4522(02)00098-2

Nimmerjahn A, 2005, SCIENCE, V308, P1314, DOI 10.1126/science.1110647

Northington FJ, 2001, J NEUROSCI, V21, P1931, DOI 10.1523/JNEUROSCI.21-06-01931.2001

Penkowa M, 2005, J NEUROSCI RES, V79, P522, DOI 10.1002/jnr.20387

Penkowa M, 2001, J NEUROTRAUM, V18, P447, DOI 10.1089/089771501750171056

Popovich PG, 2002, J NEUROPATH EXP NEUR, V61, P623, DOI 10.1093/jnen/61.7.623

Potts Mathew B, 2006, NeuroRx, V3, P143

Raivich G, 1999, BRAIN RES REV, V30, P77, DOI 10.1016/S0165-0173(99)00007-7

Rogove AD, 2002, CELL DEATH DIFFER, V9, P801, DOI 10.1038/sj.cdd.4401041

Ruppel RA, 2002, NEUROSURG CLIN N AM, V13, P169, DOI 10.1016/S1042-3680(01)00005-5

Soltys Z, 2005, J NEUROSCI METH, V146, P50, DOI 10.1016/j.jneumeth.2005.01.009

Sorensen JC, 1996, EXP BRAIN RES, V112, P203

Stence N, 2001, GLIA, V33, P256, DOI 10.1002/1098-1136(200103)33:3<256::AID-GLIA1024>3.0.CO;2-J

Streit WJ, 2000, TOXICOL PATHOL, V28, P28, DOI 10.1177/019262330002800104

STREIT WJ, 1987, J NEUROCYTOL, V16, P249, DOI 10.1007/BF01795308

Streit WJ, 1999, PROG NEUROBIOL, V57, P563, DOI 10.1016/S0301-0082(98)00069-0

Teismann P, 2004, CELL TISSUE RES, V318, P149, DOI 10.1007/s00441-004-0944-0

Trendelenburg G, 2002, J NEUROSCI, V22, P5879

Vela JM, 2002, J NEUROTRAUM, V19, P1503, DOI 10.1089/089771502320914723

VERITY MA, 1994, NEUROTOXICOLOGY, V15, P81

Walsh DT, 2000, GLIA, V29, P392, DOI 10.1002/(SICI)1098-1136(20000215)29:4<392::AID-GLIA10>3.0.CO;2-C

Wang TG, 2004, J NEUROCHEM, V88, P939, DOI 10.1046/j.1471-4159.2003.02242.x

Wang XJ, 2005, NEUROREPORT, V16, P267, DOI 10.1097/00001756-200502280-00013

West AK, 2004, REV NEUROSCIENCE, V15, P157

Yrjänheikki J, 1999, P NATL ACAD SCI USA, V96, P13496, DOI 10.1073/pnas.96.23.13496

Zhu C, 2005, CELL DEATH DIFFER, V12, P162, DOI 10.1038/sj.cdd.4401545

NR 71

TC 15

Z9 15

U1 0

U2 0

PU MARY ANN LIEBERT, INC

PI NEW ROCHELLE

PA 140 HUGUENOT STREET, 3RD FL, NEW ROCHELLE, NY 10801 USA

SN 0897-7151

EI 1557-9042

J9 J NEUROTRAUM

JI J. Neurotrauma

PD JAN

PY 2007

VL 24

IS 1

BP 28

EP 42

DI 10.1089/neu.2006.0056.R1

PG 15

WC Critical Care Medicine; Clinical Neurology; Neurosciences

WE Science Citation Index Expanded (SCI-EXPANDED)

SC General & Internal Medicine; Neurosciences & Neurology

GA 131GX

UT WOS:000243858100004

PM 17263668

DA 2024-03-03

ER

PT J

AU Koshinaga, M

Suma, T

Fukushima, M

Tsuboi, I

Aizawa, S

Katayama, Y

AF Koshinaga, M.

Suma, T.

Fukushima, M.

Tsuboi, I.

Aizawa, S.

Katayama, Y.

TI Rapid microglial activation induced by traumatic brain injury is

independent of blood brain barrier disruption

SO HISTOLOGY AND HISTOPATHOLOGY

LA English

DT Article

DE microglia; blood brain barrier; brain injury; CR3

ID SPREADING DEPRESSION; RAT; CELLS; DAMAGE; ASTROCYTES; EXPRESSION;

ISCHEMIA; RELEASE

AB Following CNS injury, microglia respond and transform into reactive species exhibiting characteristic morphological changes that have been termed "activated" or "ameboid" microglia. In an attempt to establish that microglial reactions induced immediately after injury are caused by intrinsic mechanisms rather than infiltration of blood and its constituents, oxygenized Ringer's solution was perfused into the cerebral circulation of rats so that the circulating blood could be eliminated prior to injury induction. Under artificial respiration, a catheter was inserted from the cardiac apex into the ascending aorta, and oxygenized Ringer's solution was immediately perfused with a pulsatile blood pump, resulting in wash out of the circulating blood from the brain within 1 min. Subsequently, a cortical contusion was induced in the unilateral parietal cortex using a controlled cortical impact (CCI) device. At 5 min following the injury, the brain was fixed by perfusion of fixative through the catheter and removed. Coronal vibratome sections were then processed for CR3 immunohistochemistry to examine the microglial activation. It appeared that microglial activation with both morphological transformation and an increase in CR3 immunoreactivity was induced throughout the hemisphere ipsilateral to the injury side exclusively, even in rats with elimination of circulating blood. The microglial reactions did not differ substantially from those observed in the control rats with extensive BBB disruption. The present results thus provide direct evidence that the microglial activation induced immediately after injury is independent of infiltration of circulating blood induced by concurrent BBB disruption.

C1 Nihon Univ, Sch Med, Dept Neurol Surg, Tokyo 1738610, Japan.

Nihon Univ, Sch Med, Dept Anat, Tokyo 1738610, Japan.

C3 Nihon University; Nihon University

RP Koshinaga, M (corresponding author), Nihon Univ, Sch Med, Dept Anat & Neurol Surg, 30-1 Oyaguchi Kamimachi, Tokyo 1738610, Japan.

EM koshimo@med.nihon-u.ac.jp

CR AKIYAMA H, 1988, J NEUROSCI RES, V20, P147, DOI 10.1002/jnr.490200202

Bellander BM, 2004, J NEUROTRAUM, V21, P605, DOI 10.1089/089771504774129937

Block ML, 2005, PROG NEUROBIOL, V76, P77, DOI 10.1016/j.pneurobio.2005.06.004

Bruce-Keller AJ, 1999, J NEUROSCI RES, V58, P191, DOI 10.1002/(SICI)1097-4547(19991001)58:1<191::AID-JNR17>3.0.CO;2-E

Caggiano AO, 1996, J COMP NEUROL, V369, P93, DOI 10.1002/(SICI)1096-9861(19960520)369:1<93::AID-CNE7>3.0.CO;2-F

Davalos D, 2005, NAT NEUROSCI, V8, P752, DOI 10.1038/nn1472

Del Rio-Hortega R., 1965, CYTOLOGY CELLULAR PA, P483

DIXON CE, 1991, J NEUROSCI METH, V39, P253

GEHRMANN J, 1992, J CEREBR BLOOD F MET, V12, P257, DOI 10.1038/jcbfm.1992.36

GEHRMANN J, 1993, BRAIN PATHOL, V3, P11, DOI 10.1111/j.1750-3639.1993.tb00720.x

GIULIAN D, 1989, J NEUROSCI, V9, P4416

GIULIAN D, 1994, NEUROCHEM INT, V25, P227, DOI 10.1016/0197-0186(94)90066-3

GRAEBER MB, 1988, J NEUROSCI RES, V21, P18, DOI 10.1002/jnr.490210104

HOSSMANN KA, 1973, ARCH NEUROL-CHICAGO, V29, P375, DOI 10.1001/archneur.1973.00490300037004

Inoue K, 2002, GLIA, V40, P156, DOI 10.1002/glia.10150

Jensen MB, 1997, EXP NEUROL, V143, P103, DOI 10.1006/exnr.1996.6337

KATAYAMA Y, 1995, BRAIN PATHOL, V5, P427, DOI 10.1111/j.1750-3639.1995.tb00621.x

KETTENMANN H, 1990, J NEUROSCI RES, V26, P278, DOI 10.1002/jnr.490260303

Koshinaga M, 2000, J NEUROTRAUM, V17, P185, DOI 10.1089/neu.2000.17.185

KREUTZBERG GW, 1987, ENCY NEUROSCIENCE, P661

Lighthall JW, 1988, J NEUROTRAUM, V5, P1, DOI 10.1089/neu.1988.5.1

Lu J, 2001, J NEUROTRAUM, V18, P399, DOI 10.1089/089771501750170976

Maeda T, 1998, J NEUROTRAUM, V15, P655, DOI 10.1089/neu.1998.15.655

MORIOKA T, 1991, J CEREBR BLOOD F MET, V11, P966, DOI 10.1038/jcbfm.1991.162

SHIPLEY MT, 1994, SOC NEUR ABSTR, V365, P4

SUZUMURA A, 1990, J NEUROIMMUNOL, V30, P111, DOI 10.1016/0165-5728(90)90094-4

WALZ W, 1993, J NEUROSCI, V13, P4403

NR 27

TC 12

Z9 17

U1 0

U2 0

PU F HERNANDEZ

PI MURCIA

PA PLAZA FUENSANTA 2-7 C, 30008 MURCIA, SPAIN

SN 0213-3911

EI 1699-5848

J9 HISTOL HISTOPATHOL

JI Histol. Histopath.

PD FEB

PY 2007

VL 22

IS 2

BP 129

EP 135

PG 7

WC Cell Biology; Pathology

WE Science Citation Index Expanded (SCI-EXPANDED)

SC Cell Biology; Pathology

GA 113CY

UT WOS:000242576600002

PM 17149685

DA 2024-03-03

ER

PT J

AU Redell, JB

Dash, PK

AF Redell, John B.

Dash, Pramod K.

TI Traumatic brain injury stimulates hippocampal catechol-<i>O</i>-methyl

transferase expression in microglia

SO NEUROSCIENCE LETTERS

LA English

DT Article

DE protein array; microarray; proteomics; dopamine; COMT; TBI

ID RAT FRONTAL-CORTEX; HYPOXIA-INDUCIBLE FACTOR-1-ALPHA; CONTROLLED

CORTICAL IMPACT; DOPAMINE TRANSPORTER; PROTEIN EXPRESSION; PREFRONTAL

CORTEX; WORKING-MEMORY; METHYLTRANSFERASE; TECHNOLOGY; GENOTYPE

AB Outcome following traumatic brain injury (TBI) is in large part determined by the combined action of multiple processes. In order to better understand the response of the central nervous system to injury, we utilized an antibody array to simultaneously screen 507 proteins for altered expression in the injured hippocampus, a structure critical for memory formation. Array analysis indicated 41 candidate proteins have altered expression levels 24h after TBI. Of particular interest was catechol-O-methyl transferase (COMT), an enzyme involved in metabolizing catecholamines released following neuronal activity. Altered catecholamine signaling has been observed after brain injury, and may contribute to the cognitive dysfunctions and behavioral deficits often experienced after TBI. Our data shows that COMT expression in the injured ipsilateral hippocampus was elevated for at least 14 d after controlled cortical impact injury. We found strong co-localization of COMT immunoreactivity with the microglia marker lbal near the injury site. Since dopamine transporter expression has been reported to be down-regulated after brain injury, COMT-mediated catecholamine metabolism may play a more prominent role in terminating catecholamine signaling in injured areas. (c) 2006 Elsevier Ireland Ltd. All rights reserved.

C1 Univ Texas, Sch Med, Dept Neurobiol, Houston, TX 77030 USA.

Univ Texas, Sch Med, Dept Anat, Houston, TX 77030 USA.

Univ Texas, Sch Med, Vivian L Smith Ctr Neurol Res, Houston, TX 77030 USA.

C3 University of Texas System; University of Texas System; University of

Texas System

RP Dash, PK (corresponding author), Univ Texas, Sch Med, Dept Neurobiol & Anat, POB 20708, Houston, TX 77225 USA.

EM p.dash@uth.tmc.edu

FU NIMH NIH HHS [R01 MH072933, MH072933] Funding Source: Medline; NINDS NIH

HHS [NS35457, NS049160, R01 NS035457, R01 NS049160] Funding Source:

Medline

CR Akil M, 2003, J NEUROSCI, V23, P2008

Anderson K, 2003, BRAIN, V126, P2052, DOI 10.1093/brain/awg208

Breitling R, 2004, FEBS LETT, V573, P83, DOI 10.1016/j.febslet.2004.07.055

CLIFTON GL, 1981, NEUROSURGERY, V8, P10, DOI 10.1227/00006123-198101000-00003

Dash PK, 2004, NEUROCHEM RES, V29, P1275, DOI 10.1023/B:NERE.0000023614.30084.eb

Denslow N, 2003, J NEUROTRAUM, V20, P401, DOI 10.1089/089771503765355487

Egan MF, 2001, P NATL ACAD SCI USA, V98, P6917, DOI 10.1073/pnas.111134598

Färber K, 2005, MOL CELL NEUROSCI, V29, P128, DOI 10.1016/j.mcn.2005.01.003

Gao WM, 2006, BRAIN RES, V1070, P31, DOI 10.1016/j.brainres.2005.11.038

Gogos JA, 1998, P NATL ACAD SCI USA, V95, P9991, DOI 10.1073/pnas.95.17.9991

Haskins WE, 2005, J NEUROTRAUM, V22, P629, DOI 10.1089/neu.2005.22.629

Jenkins LW, 2002, J NEUROTRAUM, V19, P715, DOI 10.1089/08977150260139101

Jiang YJ, 2002, J CEREBR BLOOD F MET, V22, P689, DOI 10.1097/00004647-200206000-00007

KAROUM F, 1994, J NEUROCHEM, V63, P972, DOI 10.1046/j.1471-4159.1994.63030972.x

Kline AE, 2002, J NEUROTRAUM, V19, P415, DOI 10.1089/08977150252932370

Kobori N, 2006, J NEUROSCI, V26, P4236, DOI 10.1523/JNEUROSCI.4687-05.2006

Kobori N, 2006, J NEUROTRAUM, V23, P1094, DOI 10.1089/neu.2006.23.1094

Lipsky RH, 2005, J NEUROPSYCH CLIN N, V17, P465, DOI 10.1176/appi.neuropsych.17.4.465

LUNDSTROM K, 1995, BBA-PROTEIN STRUCT M, V1251, P1, DOI 10.1016/0167-4838(95)00071-2

Massucci JL, 2004, NEUROSCI LETT, V372, P127, DOI 10.1016/j.neulet.2004.09.026

MCINTOSH TK, 1994, J NEUROCHEM, V63, P1426

Mu DZ, 2003, NEUROBIOL DIS, V14, P524, DOI 10.1016/j.nbd.2003.08.020

Privalsky ML, 2004, ANNU REV PHYSIOL, V66, P315, DOI 10.1146/annurev.physiol.66.032802.155556

Rall JM, 2003, NEUROPATH APPL NEURO, V29, P118, DOI 10.1046/j.1365-2990.2003.00439.x

Redell JB, 2003, EXP BIOL MED, V228, P261, DOI 10.1177/153537020322800304

Sesack SR, 1998, J NEUROSCI, V18, P2697

Srivastava M, 2006, MOL GENET METAB, V87, P303, DOI 10.1016/j.ymgme.2005.10.021

Yan HQ, 2002, NEUROREPORT, V13, P1899, DOI 10.1097/00001756-200210280-00013

Yan HQ, 2001, NEUROREPORT, V12, P2323, DOI 10.1097/00001756-200108080-00009

Zhu J, 2000, EXP NEUROL, V166, P136, DOI 10.1006/exnr.2000.7484

NR 30

TC 35

Z9 47

U1 0

U2 1

PU ELSEVIER IRELAND LTD

PI CLARE

PA ELSEVIER HOUSE, BROOKVALE PLAZA, EAST PARK SHANNON, CO, CLARE, 00000,

IRELAND

SN 0304-3940

J9 NEUROSCI LETT

JI Neurosci. Lett.

PD FEB 8

PY 2007

VL 413

IS 1

BP 36

EP 41

DI 10.1016/j.neulet.2006.11.060

PG 6

WC Neurosciences

WE Science Citation Index Expanded (SCI-EXPANDED)

SC Neurosciences & Neurology

GA 141JO

UT WOS:000244574800008

PM 17240060

OA Green Accepted, Green Submitted

DA 2024-03-03

ER

PT J

AU Bye, N

Habgood, MD

Callaway, JK

Malakooti, N

Potter, A

Kossmann, T

Morganti-Kossmann, MC

AF Bye, Nicole

Habgood, Mark D.

Callaway, Jennifer K.

Malakooti, Nakisa

Potter, Ann

Kossmann, Thomas

Morganti-Kossmann, M. Cristina

TI Transient neuroprotection by minocycline following traumatic brain

injury is associated with attenuated microglial activation but no

changes in cell apoptosis or neutrophil infiltration

SO EXPERIMENTAL NEUROLOGY

LA English

DT Article

DE apoptosis; cytokines; inflammation; microglia; minocycline; traumatic

brain injury

ID CLOSED-HEAD INJURY; INTERLEUKIN-1 RECEPTOR ANTAGONIST; FOCAL

CEREBRAL-ISCHEMIA; PROINFLAMMATORY CYTOKINE EXPRESSION; IMPROVES

FUNCTIONAL RECOVERY; CYTOCHROME-C RELEASE; SPINAL-CORD-INJURY; MOUSE

MODEL; TNF-ALPHA; DEATH

AB Cerebral inflammation and apoptotic cell death are two processes implicated in the progressive tissue damage that occurs following traumatic brain injury (TBI), and strategies to inhibit one or both of these pathways are being investigated as potential therapies for TBI patients. The tetracycline derivative minocycline was therapeutically effective in various models of central nervous system injury and disease, via mechanisms involving suppression of inflammation and apoptosis. We therefore investigated the effect of minocycline in TBI using a closed head injury model. Following TBI, mice were treated with minocycline or vehicle, and the effect on neurological outcome, lesion volume, inflammation and apoptosis was evaluated for up to 7 days. Our results show that while minocycline decreases lesion volume and improves neurological outcome at I day post-trauma, this response is not maintained at 4 days. The early beneficial effect is likely not due to anti-apoptotic mechanisms, as the density of apoptotic cells is not affected at either time-point. However, protection by minocycline is associated with a selective anti-inflammatory response, in that microglial activation and interleukin-1 beta expression are reduced, while neutrophil infiltration and expression of multiple cytokines are not affected. These findings demonstrate that further studies on minocycline in TBI are necessary in order to consider it as a novel therapy for brain-injured patients. Crown Copyright (c) 2006 Published by Elsevier Inc. All rights reserved.

C1 Monash Univ, Alfred Hosp Dept Med, Natl Trauma Res Inst, Clayton, Vic 3168, Australia.

Monash Univ, Alfred Hosp Dept Med, Dept Trauma Surg, Clayton, Vic 3168, Australia.

Monash Univ, Dept Med, Clayton, Vic 3168, Australia.

Monash Univ, Dept Surg, Clayton, Vic 3168, Australia.

Univ Melbourne, Dept Pharmacol, Parkville, Vic 3052, Australia.

C3 Monash University; Monash University; Monash University; Monash

University; University of Melbourne

RP Morganti-Kossmann, MC (corresponding author), Monash Univ, Alfred Hosp Dept Med, Natl Trauma Res Inst, Clayton, Vic 3168, Australia.

EM cristina.morganti-kossmann@med.monash.edu.au

RI Malakooti, Nakisa/V-4775-2019

OI Malakooti, Nakisa/0000-0002-8638-8848; Morganti-Kossmann,

Cristina/0000-0002-0807-2063

CR Arvin KL, 2002, ANN NEUROL, V52, P54, DOI 10.1002/ana.10242

Beni-Adani L, 2001, J PHARMACOL EXP THER, V296, P57

Bethea JR, 1999, J NEUROTRAUM, V16, P851, DOI 10.1089/neu.1999.16.851

Boutin H, 2001, J NEUROSCI, V21, P5528, DOI 10.1523/JNEUROSCI.21-15-05528.2001

BOUTIN H, 2001, J NEUROSCI, V21, pA1

Chen M, 2000, NAT MED, V6, P797, DOI 10.1038/77528

Chen Y, 1996, J NEUROTRAUM, V13, P557, DOI 10.1089/neu.1996.13.557

Diguet E, 2004, EUR J NEUROSCI, V19, P3266, DOI 10.1111/j.0953-816X.2004.03372.x

Du YS, 2001, P NATL ACAD SCI USA, V98, P14669, DOI 10.1073/pnas.251341998

Fan LW, 2005, J NEUROSCI RES, V82, P71, DOI 10.1002/jnr.20623

Fox C, 2005, J CEREBR BLOOD F MET, V25, P1138, DOI 10.1038/sj.jcbfm.9600121

GARCIA JH, 1995, AM J PATHOL, V147, P1477

He Y, 2001, BRAIN RES, V909, P187, DOI 10.1016/S0006-8993(01)02681-6

Hua R, 2006, BRAIN RES, V1090, P172, DOI 10.1016/j.brainres.2006.03.072

Keane RW, 2001, J CEREBR BLOOD F MET, V21, P1189, DOI 10.1097/00004647-200110000-00007

Knoblach SM, 2002, J NEUROTRAUM, V19, P1155, DOI 10.1089/08977150260337967

Krady JK, 2005, DIABETES, V54, P1559, DOI 10.2337/diabetes.54.5.1559

Lawrence CB, 1998, EUR J NEUROSCI, V10, P1188, DOI 10.1046/j.1460-9568.1998.00136.x

Ledeboer A, 2005, PAIN, V115, P71, DOI 10.1016/j.pain.2005.02.009

Lee SM, 2004, J NEUROCHEM, V91, P568, DOI 10.1111/j.1471-4159.2004.02780.x

Lee SM, 2003, J NEUROTRAUM, V20, P1017, DOI 10.1089/089771503770195867

Loddick S.A., 2002, IMMUN INFLAMM DIS, P90

Loddick SA, 1996, J CEREBR BLOOD F MET, V16, P932, DOI 10.1097/00004647-199609000-00017

McIntosh TK, 1998, NEUROPATH APPL NEURO, V24, P251

Mejia ROS, 2001, NEUROSURGERY, V48, P1393, DOI 10.1097/00006123-200106000-00051

Morganti-Kossmann MC, 2001, SHOCK, V16, P165, DOI 10.1097/00024382-200116030-00001

Morimoto N, 2005, BRAIN RES, V1044, P8, DOI 10.1016/j.brainres.2005.02.062

Muzha I, 2004, LANCET, V364, P1321, DOI 10.1016/S0140-6736(04)17188-2

Otto VI, 2001, NEUROREPORT, V12, P2059, DOI 10.1097/00001756-200107030-00053

Popovic N, 2002, ANN NEUROL, V51, P215, DOI 10.1002/ana.10092

Raghupathi R, 2004, BRAIN PATHOL, V14, P215, DOI 10.1111/j.1750-3639.2004.tb00056.x

Rancan M, 2004, J CEREBR BLOOD F MET, V24, P1110, DOI 10.1097/01.WCB.0000133470.91843.72

Schallert T, 2000, PHARMACOLOGY OF CEREBRAL ISCHEMIA 2000, P329

SHOHAMI E, 1994, J CEREBR BLOOD F MET, V14, P615, DOI 10.1038/jcbfm.1994.76

Shohami E, 1996, J CEREBR BLOOD F MET, V16, P378, DOI 10.1097/00004647-199605000-00004

Smith DL, 2003, ANN NEUROL, V54, P186, DOI 10.1002/ana.10614

Sriram K, 2006, J NEUROCHEM, V96, P706, DOI 10.1111/j.1471-4159.2005.03566.x

Stahel PF, 2000, J CEREBR BLOOD F MET, V20, P369, DOI 10.1097/00004647-200002000-00019

Stirling DP, 2004, J NEUROSCI, V24, P2182, DOI 10.1523/JNEUROSCI.5275-03.2004

Szymanska A, 2006, EXP NEUROL, V197, P189, DOI 10.1016/j.expneurol.2005.09.011

Tehranian R, 2002, J NEUROTRAUM, V19, P939, DOI 10.1089/089771502320317096

Teng YD, 2004, P NATL ACAD SCI USA, V101, P3071, DOI 10.1073/pnas.0306239101

Tikka T, 2001, J NEUROSCI, V21, P2580, DOI 10.1523/JNEUROSCI.21-08-02580.2001

Tikka TM, 2001, J IMMUNOL, V166, P7527, DOI 10.4049/jimmunol.166.12.7527

TOULMOND S, 1995, BRAIN RES, V671, P261, DOI 10.1016/0006-8993(94)01343-G

Tsuji M, 2004, EXP NEUROL, V189, P58, DOI 10.1016/j.expneurol.2004.01.011

Wang CX, 2003, BRAIN RES, V963, P327, DOI 10.1016/S0006-8993(02)04045-3

Wu DC, 2002, J NEUROSCI, V22, P1763, DOI 10.1523/JNEUROSCI.22-05-01763.2002

Xu L, 2004, BMC NEUROL, V4, DOI 10.1186/1471-2377-4-7

YAMASAKI Y, 1995, STROKE, V26, P676, DOI 10.1161/01.STR.26.4.676

Yang LC, 2003, J NEUROSCI RES, V74, P278, DOI 10.1002/jnr.10709

Yrjänheikki J, 1999, P NATL ACAD SCI USA, V96, P13496, DOI 10.1073/pnas.96.23.13496

Yrjänheikki J, 1998, P NATL ACAD SCI USA, V95, P15769, DOI 10.1073/pnas.95.26.15769

Zhu S, 2002, NATURE, V417, P74, DOI 10.1038/417074a

NR 54

TC 197

Z9 213

U1 1

U2 23

PU ACADEMIC PRESS INC ELSEVIER SCIENCE

PI SAN DIEGO

PA 525 B ST, STE 1900, SAN DIEGO, CA 92101-4495 USA

SN 0014-4886

EI 1090-2430

J9 EXP NEUROL

JI Exp. Neurol.

PD MAR

PY 2007

VL 204

IS 1

BP 220

EP 233

DI 10.1016/j.expneurol.2006.10.013

PG 14

WC Neurosciences

WE Science Citation Index Expanded (SCI-EXPANDED)

SC Neurosciences & Neurology

GA 148PC

UT WOS:000245086100022

PM 17188268

DA 2024-03-03

ER

PT J

AU Matsumoto, H

Kumon, Y

Watanabe, H

Ohnishi, T

Shudou, M

Ii, C

Takahashi, H

Imai, Y

Tanaka, J

AF Matsumoto, Hiroaki

Kumon, Yoshiaki

Watanabe, Hideaki

Ohnishi, Takanori

Shudou, Masachika

Ii, Chisato

Takahashi, Hisaaki

Imai, Yoshinori

Tanaka, Junya

TI Antibodies to CD11b, CD68, and lectin label neutrophils rather than

microglia in traumatic and ischemic brain lesions

SO JOURNAL OF NEUROSCIENCE RESEARCH

LA English

DT Article

DE MCAO; stab wound; microglia; macrophage; Iba1; NO; proinflammatory

cytokines

ID CEREBRAL-ARTERY OCCLUSION; NITRIC-OXIDE SYNTHASE; SPINAL-CORD-INJURY;

NG2 PROTEOGLYCAN; FOCAL ISCHEMIA; CELLS; EXPRESSION; REPERFUSION;

CORTEX; OLIGODENDROCYTES

AB Resident quiescent microglia have been thought to respond rapidly to various pathologic events in the brain by proliferating and producing many bioactive substances, including proinflammatory cytokines and nitric oxide (NO). In this study, we investigated the reaction of microglia in traumatic and ischemic lesions caused by stab wounds and the transient 90-min occlusion of middle cerebral artery in a mature rat brain. Although many lba1(+) resident microglia underwent apoptotic degeneration in the lesion core within 24 hr after the onset of the brain insult as revealed by TUNEL staining, numerous small, round, isolectin B4(+)/CD11b(+)/CD68(+) cells were localized in the lesion core. These small, round cells with diameters of 7-9 mu m and polymorph nuclei expressed neutrophil-specific elastase, alkaline phosphatase, and platelet-activating factor receptor. Accordingly, they were not activated microglia but neutrophils. Immunohistochemical staining with antibodies to inducible NO synthase (iNOS) showed that most iNOS(+) cells were neutrophils. The results from spatial and kinetic analyses using RT-PCR and immunoblotting were consistent with the immunohistochemical observations. These results suggest the necessity of reevaluating the traditional view on the roles of activated microglia in severe neuropathologic events. Note that the traditional microglial markers isolectin B4, CD11b, and CD68 are not specific for microglia, particularly in a pathologic brain. (c) 2007 Wiley-Liss, Inc.

C1 Ehime Univ, Grad Sch Med, Dept Mol & Cellular Physiol, Toon, Ehime 7910295, Japan.

Ehime Univ, Grad Sch Med, Dept Neurosurg, Toon, Ehime 7910295, Japan.

Ehime Univ, Integrated Ctr Sci, Toon, Ehime 7910295, Japan.

C3 Ehime University; Ehime University; Ehime University

RP Tanaka, J (corresponding author), Ehime Univ, Grad Sch Med, Dept Mol & Cellular Physiol, Toon, Ehime 7910295, Japan.

EM jtanaka@in.ehime-u.ac.jp

RI Tanaka, Junya/Y-1033-2019; Ohnishi, Takanori/ABN-8258-2022

OI Tanaka, Junya/0000-0003-1056-5948

CR BANATI RB, 1993, GLIA, V7, P111, DOI 10.1002/glia.440070117

BARONE FC, 1992, STROKE, V23, P1337, DOI 10.1161/01.STR.23.9.1337

BEDERSON JB, 1986, STROKE, V17, P472, DOI 10.1161/01.STR.17.3.472

Beray-Berthat V, 2003, BRAIN RES, V987, P32, DOI 10.1016/S0006-8993(03)03224-4

Bu J, 2001, GLIA, V34, P296, DOI 10.1002/glia.1063

Carlson SL, 1998, EXP NEUROL, V151, P77, DOI 10.1006/exnr.1998.6785

Gibson CL, 2005, GLIA, V50, P417, DOI 10.1002/glia.20143

Gowing G, 2006, GLIA, V53, P331, DOI 10.1002/glia.20288

Hashimoto H, 1999, ACTA NEUROPATHOL, V98, P603, DOI 10.1007/s004010051125

Heppner FL, 2005, NAT MED, V11, P146, DOI 10.1038/nm1177

HWANG SB, 1988, J BIOL CHEM, V263, P3225

Iadecola C, 1997, J NEUROSCI, V17, P9157

Imai Y, 1996, BIOCHEM BIOPH RES CO, V224, P855, DOI 10.1006/bbrc.1996.1112

Ito D, 2001, STROKE, V32, P1208, DOI 10.1161/01.STR.32.5.1208

Ito D, 1998, MOL BRAIN RES, V57, P1, DOI 10.1016/S0169-328X(98)00040-0

Jones LL, 2002, J NEUROSCI, V22, P2792, DOI 10.1523/JNEUROSCI.22-07-02792.2002

Kato H, 1996, BRAIN RES, V734, P203, DOI 10.1016/S0006-8993(96)00636-1

Kitamura Y, 1996, GLIA, V18, P233, DOI 10.1002/(SICI)1098-1136(199611)18:3<233::AID-GLIA7>3.0.CO;2-#

Kreutzberg GW, 1996, TRENDS NEUROSCI, V19, P312, DOI 10.1016/0166-2236(96)10049-7

Kuwabara Y, 2003, J NEUROSCI RES, V73, P22, DOI 10.1002/jnr.10637

LING EA, 1993, GLIA, V7, P9, DOI 10.1002/glia.440070105

Mobberley-Schuman PS, 2005, INFECT IMMUN, V73, P7317, DOI 10.1128/IAI.73.11.7317-7323.2005

Mori K, 2002, NEUROPHARMACOLOGY, V43, P1026, DOI 10.1016/S0028-3908(02)00211-3

MORIOKA T, 1991, J CEREBR BLOOD F MET, V11, P966, DOI 10.1038/jcbfm.1991.162

Nakajima K., 2005, NEUROGLIA, V2nd ed., P443

Neudenberger J, 2006, BRIT J HAEMATOL, V133, P337, DOI 10.1111/j.1365-2141.2006.06013.x

Paietta E, 1998, BRIT J HAEMATOL, V100, P265, DOI 10.1046/j.1365-2141.1998.00561.x

PERSSON L, 1976, VIRCHOWS ARCH B, V22, P21

SAITO N, 1991, AM J PATHOL, V139, P1053

Schroeter M, 1997, STROKE, V28, P382, DOI 10.1161/01.STR.28.2.382

Shibano M, 1999, AM J HEMATOL, V60, P12, DOI 10.1002/(SICI)1096-8652(199901)60:1<12::AID-AJH3>3.0.CO;2-P

Shimakura A, 2000, BRAIN RES, V858, P55, DOI 10.1016/S0006-8993(99)02431-2

Simard AR, 2006, NEURON, V49, P489, DOI 10.1016/j.neuron.2006.01.022

Strbian D, 2006, J CEREBR BLOOD F MET, V26, P605, DOI 10.1038/sj.jcbfm.9600228

STREIT WJ, 2005, NEUROGLIA, P60

Sugishita H, 2001, J NEUROSCI RES, V64, P392, DOI 10.1002/jnr.1090

Tanaka J, 1997, GLIA, V19, P286

Tanaka J, 1996, EXP NEUROL, V137, P367, DOI 10.1006/exnr.1996.0038

Tanaka R, 2003, NEUROSCIENCE, V117, P531, DOI 10.1016/S0306-4522(02)00954-5

Wang Y, 2007, EXP NEUROL, V203, P168, DOI 10.1016/j.expneurol.2006.08.006

Weston RM, 2007, J CEREBR BLOOD F MET, V27, P100, DOI 10.1038/sj.jcbfm.9600324

Wu CT, 2003, HEPATOLOGY, V38, P1018, DOI 10.1053/jhep.2003.50407

Yokoyama A, 2006, GLIA, V53, P754, DOI 10.1002/glia.20332

Yokoyama A, 2004, GLIA, V45, P96, DOI 10.1002/glia.10306

Zai LJ, 2005, GLIA, V50, P247, DOI 10.1002/glia.20176

Zhang ZG, 1997, BRAIN RES, V744, P189, DOI 10.1016/S0006-8993(96)01085-2

NR 46

TC 91

Z9 99

U1 2

U2 20

PU WILEY

PI HOBOKEN

PA 111 RIVER ST, HOBOKEN 07030-5774, NJ USA

SN 0360-4012

EI 1097-4547

J9 J NEUROSCI RES

JI J. Neurosci. Res.

PD APR

PY 2007

VL 85

IS 5

BP 994

EP 1009

DI 10.1002/jnr.21198

PG 16

WC Neurosciences

WE Science Citation Index Expanded (SCI-EXPANDED)

SC Neurosciences & Neurology

GA 157NK

UT WOS:000245726700008

PM 17265469

DA 2024-03-03

ER

PT J

AU Beschorner, R

Dietz, K

Schauer, K

Mittelbronn, M

Schluesener, HJ

Trautmann, K

Meyermann, R

Simon, P

AF Beschorner, R.

Dietz, K.

Schauer, K. N.

Mittelbronn, M.

Schluesener, H. J.

Trautmann, K.

Meyermann, R.

Simon, P.

TI Expression of EAAT1 reflects a possible neuroprotective function of

reactive astrocytes and activated microglia following human traumatic

brain injury

SO HISTOLOGY AND HISTOPATHOLOGY

LA English

DT Article

DE microglial activation; neuroprotection; traumatic brain injury;

excitatory amino acid transporters

ID GLIAL GLUTAMATE TRANSPORTER; AMINO-ACID TRANSPORTER-1; FOCAL

CEREBRAL-ISCHEMIA; GLT-1 EXPRESSION; CELL-DEATH; CLINICAL-TRIALS;

CORTICAL IMPACT; MICE LACKING; SPINAL-CORD; RAT MODEL

AB Glutamate-mediated excitotoxicity is known to cause secondary brain damage following stroke and traumatic brain injury (TBI). However, clinical trials using NMDA antagonists failed. Thus, glial excitatory amino acid transporters (EAATs) might be a promising target for therapeutic intervention. Methods and Results. We examined expression of EAAT1 (GLAST) and EAAT2 (Glt-1) in 36 TBI cases by immunohistochemistry. Cortical expression of both EAATs decreased rapidly and widespread throughout the brain (in lesional, adjacent and remote areas) following TBI. In the white matter numbers of EAAT1+ parenchymal cells increased 39-fold within 24h (p < 0.001) and remained markedly elevated till later stages in the lesion (90-fold, p < 0.01) and in peri-lesional regions (86-fold, p < 0.01). In contrast, EAAT2+ parenchymal cells and EAAT1+ or EAAT2+ perivascular cells did not increase significantly. Within the first days following TBI mainly activated microglia and thereafter mainly reactive astrocytes expressed EAAT1. Perivascular monocytes and foamy macrophages lacked EAAT1 immunoreactivity. We conclude that following TBI i) loss of cortical EAATs contributes to secondary brain damage, ii) glial EAAT1 expression reflects a potential neuroprotective function of microglia and astrocytes, iii) microglial EAAT1 expression is restricted to an early stage of activation, iv) blood-derived monocytes do not express EAAT1 and v) pharmacological modification of glial EAAT expression might further limit neuronal damage.

C1 Univ Tubingen, Inst Brain Res, D-72076 Tubingen, Germany.

Univ Tubingen, Dept Med Biometry, D-72076 Tubingen, Germany.

Univ Tubingen, Clin Internal Med, Dept Sports Med, D-72076 Tubingen, Germany.

C3 Eberhard Karls University of Tubingen; Eberhard Karls University of

Tubingen; Eberhard Karls University Hospital; Eberhard Karls University

of Tubingen

RP Beschorner, R (corresponding author), Univ Tubingen, Inst Brain Res, Calwestr 3, D-72076 Tubingen, Germany.

EM rudi.beschorner@med.uni-tuebingen.de

RI Simon, Perikles D/B-2293-2013; Beschorner, Rudi/M-6397-2014; Dietz,

Klaus/R-9268-2016

OI Simon, Perikles D/0000-0002-7996-4034; Dietz, Klaus/0000-0001-8503-9737;

Beschorner, Rudi/0000-0003-1109-915X; Mittelbronn,

Michel/0000-0002-2998-052X

CR ANDERSON KJ, 1991, BRAIN RES, V562, P285, DOI 10.1016/0006-8993(91)90633-7

Arvidsson A, 2001, EUR J NEUROSCI, V14, P10, DOI 10.1046/j.0953-816x.2001.01611.x

BAKER AJ, 1993, J NEUROSURG, V79, P369, DOI 10.3171/jns.1993.79.3.0369

Banner SJ, 2002, NEUROSCIENCE, V109, P27, DOI 10.1016/S0306-4522(01)00437-7

Baskaya MK, 2000, J NEUROSURG, V92, P448, DOI 10.3171/jns.2000.92.3.0448

BENVENISTE H, 1984, J NEUROCHEM, V43, P1369, DOI 10.1111/j.1471-4159.1984.tb05396.x

Beschorner R, 2002, ACTA NEUROPATHOL, V103, P541, DOI 10.1007/s00401-001-0503-7

Bonde C, 2003, NEUROCHEM INT, V43, P371, DOI 10.1016/S0197-0186(03)00024-X

BONDOLI A, 1981, RESUSCITATION, V9, P119, DOI 10.1016/0300-9572(81)90021-6

Cakir E, 2005, J CLIN NEUROSCI, V12, P923, DOI 10.1016/j.jocn.2005.03.013

Castillo J, 1996, STROKE, V27, P1060, DOI 10.1161/01.STR.27.6.1060

Chen WH, 2005, NEUROSCI LETT, V384, P117, DOI 10.1016/j.neulet.2005.04.070

CHOI DW, 1990, ANNU REV NEUROSCI, V13, P171, DOI 10.1146/annurev.neuro.13.1.171

Chrétien F, 2002, NEUROPATH APPL NEURO, V28, P410, DOI 10.1046/j.1365-2990.2002.00426.x

Chrétien F, 2004, J NEUROPATH EXP NEUR, V63, P1058, DOI 10.1093/jnen/63.10.1058

Clausen F, 2004, J NEUROTRAUM, V21, P1168, DOI 10.1089/neu.2004.21.1168

De Keyser J, 1999, TRENDS NEUROSCI, V22, P535, DOI 10.1016/S0166-2236(99)01463-0

Dirnagl U, 1999, TRENDS NEUROSCI, V22, P391, DOI 10.1016/S0166-2236(99)01401-0

Duan SM, 1999, J NEUROSCI, V19, P10193

Eisenstein M, 2005, LAB ANIMAL, V34, P10

Farkas O, 2004, REGUL PEPTIDES, V123, P69, DOI 10.1016/j.regpep.2004.05.014

Fournier KM, 2004, J BIOL CHEM, V279, P34505, DOI 10.1074/jbc.M404032200

Fukamachi S, 2001, DEV BRAIN RES, V132, P131, DOI 10.1016/S0165-3806(01)00303-0

Ginsberg SD, 1996, J NEUROCHEM, V67, P1208

Graham DI., 2002, Greenfieldfs Neuropathology, V7, P823

Gras G, 2003, BRAIN PATHOL, V13, P211

Hoyte L, 2004, CURR MOL MED, V4, P131, DOI 10.2174/1566524043479248

Hurtado O, 2005, NEUROBIOL DIS, V18, P336, DOI 10.1016/j.nbd.2004.10.006

Ikematsu K, 2002, FORENSIC SCI INT, V130, P83, DOI 10.1016/S0379-0738(02)00344-4

Ikematsu K, 2001, FORENSIC SCI INT, V118, P49, DOI 10.1016/S0379-0738(00)00378-9

Ikonomidou C, 2002, LANCET NEUROL, V1, P383, DOI 10.1016/S1474-4422(02)00164-3

Ikonomidou C, 2000, P NATL ACAD SCI USA, V97, P12885, DOI 10.1073/pnas.220412197

KANNER BI, 1978, BIOCHEMISTRY-US, V17, P3949, DOI 10.1021/bi00612a011

Kaya SS, 1999, BRAIN RES, V818, P23, DOI 10.1016/S0006-8993(98)01204-9

Knecht K, 1997, NEUROSCI LETT, V229, P201, DOI 10.1016/S0304-3940(97)00444-8

Krum JM, 2002, EXP NEUROL, V174, P137, DOI 10.1006/exnr.2002.7867

López-Bayghen E, 2004, J NEUROCHEM, V91, P200, DOI 10.1111/j.1471-4159.2004.02706.x

Lopez-Redondo F, 2000, MOL BRAIN RES, V76, P429, DOI 10.1016/S0169-328X(00)00022-X

Matsuura S, 2002, GLIA, V37, P178, DOI 10.1002/glia.10020

Mitani A, 2003, J NEUROSCI, V23, P7176, DOI 10.1523/JNEUROSCI.23-18-07176.2003

Mittelbronn M, 2001, ACTA NEUROPATHOL, V101, P249

Mori T, 2004, NEUROCHEM INT, V45, P381, DOI 10.1016/j.neuint.2003.06.001

Muir K. W., 2003, COCHRANE DB SYST REV

Nakajima K, 2001, NEUROSCI LETT, V307, P171, DOI 10.1016/S0304-3940(01)01943-7

Namura S, 2002, NEUROSCI LETT, V324, P117, DOI 10.1016/S0304-3940(02)00193-3

Niederberger E, 2003, NEUROSCIENCE, V116, P81, DOI 10.1016/S0306-4522(02)00547-X

Palacin M, 1998, PHYSIOL REV, V78, P969, DOI 10.1152/physrev.1998.78.4.969

Pawlak J, 2005, MOL BRAIN RES, V138, P1, DOI 10.1016/j.molbrainres.2004.10.043

PERSSON L, 1992, J NEUROSURG, V76, P72, DOI 10.3171/jns.1992.76.1.0072

Persson M, 2005, GLIA, V51, P111, DOI 10.1002/glia.20191

Phillis JW, 2000, BRAIN RES, V868, P105, DOI 10.1016/S0006-8993(00)02303-9

Rao VLR, 1998, J NEUROCHEM, V70, P2020

Rao VLR, 2001, NEUROCHEM RES, V26, P497

Rimaniol AC, 2000, J IMMUNOL, V164, P5430, DOI 10.4049/jimmunol.164.10.5430

Robelet S, 2004, EUR J NEUROSCI, V20, P1255, DOI 10.1111/j.1460-9568.2004.03591.x

Robinson MB, 1998, NEUROCHEM INT, V33, P479, DOI 10.1016/S0197-0186(98)00055-2

Rossi DJ, 2000, NATURE, V403, P316, DOI 10.1038/35002090

Rothstein JD, 1996, NEURON, V16, P675, DOI 10.1016/S0896-6273(00)80086-0

ROTHSTEIN JD, 1993, P NATL ACAD SCI USA, V90, P6591, DOI 10.1073/pnas.90.14.6591

Rothstein JD, 2005, NATURE, V433, P73, DOI 10.1038/nature03180

Rozyczka J, 2004, BRAIN PATHOL, V14, P406

Shimada F, 1999, EUR J PHARMACOL, V386, P263, DOI 10.1016/S0014-2999(99)00735-9

Simantov R, 1999, J NEUROCHEM, V73, P1828

Simantov R, 1999, MOL BRAIN RES, V65, P112, DOI 10.1016/S0169-328X(98)00349-0

Swanson RA, 1997, J NEUROSCI, V17, P932

Tanaka K, 1997, SCIENCE, V276, P1699, DOI 10.1126/science.276.5319.1699

TORP R, 1995, EXP BRAIN RES, V103, P51

Umemura K, 1996, STROKE, V27, P1624, DOI 10.1161/01.STR.27.9.1624

Vallat-Decouvelaere AV, 2003, J NEUROPATH EXP NEUR, V62, P475, DOI 10.1093/jnen/62.5.475

Van Landeghem FKH, 2001, GLIA, V35, P167, DOI 10.1002/glia.1082

Vera-Portocarrero LP, 2002, BRAIN RES, V927, P104, DOI 10.1016/S0006-8993(01)03329-7

Voutsinos-Porche B, 2003, CEREB CORTEX, V13, P1110, DOI 10.1093/cercor/13.10.1110

WADICHE JI, 1995, NEURON, V14, P1019, DOI 10.1016/0896-6273(95)90340-2

Wang CX, 2005, CNS NEUROL DISORD-DR, V4, P143, DOI 10.2174/1568007053544183

Werner P, 2001, ANN NEUROL, V50, P169, DOI 10.1002/ana.1077

Williams SM, 2005, GLIA, V49, P520, DOI 10.1002/glia.20139

NR 76

TC 50

Z9 60

U1 0

U2 3

PU F HERNANDEZ

PI MURCIA

PA PLAZA FUENSANTA 2-7 C, 30008 MURCIA, SPAIN

SN 0213-3911

EI 1699-5848

J9 HISTOL HISTOPATHOL

JI Histol. Histopath.

PD MAY

PY 2007

VL 22

IS 5

BP 515

EP 526

PG 12

WC Cell Biology; Pathology

WE Science Citation Index Expanded (SCI-EXPANDED)

SC Cell Biology; Pathology

GA 145TA

UT WOS:000244886200005

PM 17330806

DA 2024-03-03

ER

PT J

AU Zhang, ZY

Zhang, ZR

Artelt, M

Burnet, M

Schluesener, HJ

AF Zhang, Zhiyuan

Zhang, Zhiren

Artelt, Matthias

Burnet, Michael

Schluesener, Hermann J.

TI Dexamethasone attenuates early expression of three molecules associated

with microglia/macrophages activation following rat traumatic brain

injury

SO ACTA NEUROPATHOLOGICA

LA English

DT Article

DE dexamethasone; traumatic brain injury; EMAP-II; P2X4R; AIF-1;

microglia/macrophages

ID INFLAMMATORY FACTOR-I; NECROSIS-FACTOR-ALPHA; SPINAL-CORD-INJURY;

EXPERIMENTAL AUTOIMMUNE ENCEPHALOMYELITIS; NITRIC-OXIDE SYNTHASE;

MICROGLIAL CELLS; POLYPEPTIDE-II; MACROPHAGES/MICROGLIAL CELLS; RECEPTOR

ACTIVATION; LESIONAL EXPRESSION

AB Corticosteroids have been used in the treatment of human traumatic brain injury ( TBI), which is a leading cause of death and disability, but their efficiency is still a matter of debate. Dexamethasone was considered to delay post-traumatic inflammation and retard neuronal degeneration, resulting in attenuation of secondary injury following experimental TBI. In a rat TBI model, we have investigated the effects of dexamethasone on expression patterns of markers of inflammatory activation of microglia/macrophages by immunohistochemistry. Endothelial-monocyte activating polypeptide II (EMAP-II), P2X4 receptor (P2X4R) and allograft-inflammatory factor-1 (AIF-1) were reported to be associated with the activation of microglia/macrophages post central nervous system (CNS) injury and may play roles in inflammatory cascades of secondary brain damage. Dexamethasone significantly suppressed the accumulation of EMAP-II+, P2X4R(+) or AIF(+) cells at Day-1 and 2 post-brain-trauma but not on Days 4 and 6, which is in accordance with the reported short- but not long-term protective effects of dexamethasone in TBI. These findings indicate a rather rapid but transient anti-inflammatory effect of dexamethasone in TBI.

C1 Univ Tubingen, Brain Res Inst, D-72076 Tubingen, Germany.

Synovo GmbH, D-72076 Tubingen, Germany.

C3 Eberhard Karls University of Tubingen; Synovo GmbH

RP Zhang, ZY (corresponding author), Univ Tubingen, Brain Res Inst, Calwer Str 3, D-72076 Tubingen, Germany.

EM zhangzy@gmx.de

RI Zhang, Zhiren/O-1012-2019; Burnet, Michael/AAB-2958-2019

OI Zhang, Zhiren/0000-0002-5238-2835; Burnet, Michael/0000-0003-4311-5441

CR Adelson PD, 1998, ACT NEUR S, V71, P104

AUPHAN N, 1995, SCIENCE, V270, P286, DOI 10.1126/science.270.5234.286

BANATI RB, 1993, GLIA, V7, P111, DOI 10.1002/glia.440070117

Beschorner R, 2000, ACTA NEUROPATHOL, V100, P627, DOI 10.1007/s004010000232

BOSSCHER KD, 1997, P NATL ACAD SCI USA, V94, P13504

Brough D, 2002, MOL CELL NEUROSCI, V19, P272, DOI 10.1006/mcne.2001.1054

CARLOS TM, 1994, BLOOD, V84, P2068

Cavaliere F, 2003, NEUROSCIENCE, V120, P85, DOI 10.1016/S0306-4522(03)00228-8

CLARK RSB, 1994, J NEUROTRAUM, V11, P499, DOI 10.1089/neu.1994.11.499

Colton CA, 1996, NEUROCHEM INT, V29, P43, DOI 10.1016/0197-0186(95)00139-5

DAVID S, 1990, NEURON, V5, P463, DOI 10.1016/0896-6273(90)90085-T

Deininger MH, 2000, ACTA NEUROPATHOL, V100, P673, DOI 10.1007/s004010000233

Deininger MH, 2002, FEBS LETT, V514, P115, DOI 10.1016/S0014-5793(02)02430-4

Fan L, 1996, MOL BRAIN RES, V36, P287, DOI 10.1016/0169-328X(95)00274-V

Fauser S, 2001, ACTA NEUROPATHOL, V101, P565

FEENEY DM, 1981, BRAIN RES, V211, P67, DOI 10.1016/0006-8993(81)90067-6

Ferrari D, 1997, J EXP MED, V185, P579, DOI 10.1084/jem.185.3.579

Gahm C, 2005, ACTA NEUROCHIR, V147, P1071, DOI 10.1007/s00701-005-0590-7

Gendron FP, 2003, J NEUROCHEM, V87, P344, DOI 10.1046/j.1471-4159.2003.01995.x

Golarai G, 2001, J NEUROSCI, V21, P8523, DOI 10.1523/JNEUROSCI.21-21-08523.2001

Gottesfeld Z, 2002, J NEUROTRAUM, V19, P317, DOI 10.1089/089771502753594882

Gottschall PE, 1996, NEUROREPORT, V7, P3077, DOI 10.1097/00001756-199611250-00057

Gourin CG, 1997, J TRAUMA, V42, P1101, DOI 10.1097/00005373-199706000-00020

Graeber MB, 1998, BRAIN RES, V813, P241, DOI 10.1016/S0006-8993(98)00859-2

HALL ED, 1987, J PHARMACOL EXP THER, V242, P137

Haynes LE, 2001, NEUROSCIENCE, V104, P57, DOI 10.1016/S0306-4522(01)00070-7

He J, 2004, EXP NEUROL, V189, P404, DOI 10.1016/j.expneurol.2004.06.008

Holmin, 1996, ACTA NEUROCHIR, V138, P418, DOI 10.1007/BF01420304

Ito D, 1998, MOL BRAIN RES, V57, P1, DOI 10.1016/S0169-328X(98)00040-0

KAO J, 1992, J BIOL CHEM, V267, P20239

KIEFER R, 1991, J NEUROIMMUNOL, V34, P99, DOI 10.1016/0165-5728(91)90119-R

Knies UE, 1998, P NATL ACAD SCI USA, V95, P12322, DOI 10.1073/pnas.95.21.12322

Knoblach SM, 1999, J NEUROIMMUNOL, V95, P115, DOI 10.1016/S0165-5728(98)00273-2

Kreutzberg GW, 1996, TRENDS NEUROSCI, V19, P312, DOI 10.1016/0166-2236(96)10049-7

Ladeby R, 2005, BRAIN RES REV, V48, P196, DOI 10.1016/j.brainresrev.2004.12.009

Lehrmann E, 1997, J COMP NEUROL, V386, P461

Marshall LF, 1998, J NEUROSURG, V89, P519, DOI 10.3171/jns.1998.89.4.0519

Mitchell IJ, 1998, NEUROSCIENCE, V84, P489, DOI 10.1016/S0306-4522(97)00534-4

MORI N, 1992, EPILEPSIA, V33, P994, DOI 10.1111/j.1528-1157.1992.tb01749.x

Mueller CA, 2003, J NEUROTRAUM, V20, P1007, DOI 10.1089/089771503770195858

Mueller CA, 2003, J NEUROIMMUNOL, V135, P1, DOI 10.1016/S0165-5728(02)00427-7

Narayan RK, 2002, J NEUROTRAUM, V19, P503, DOI 10.1089/089771502753754037

North RA, 2002, PHYSIOL REV, V82, P1013, DOI 10.1152/physrev.00015.2002

Orihara Y, 2001, FORENSIC SCI INT, V123, P142, DOI 10.1016/S0379-0738(01)00537-0

Postler E, 2000, J NEUROIMMUNOL, V104, P85, DOI 10.1016/S0165-5728(99)00222-2

Pulliam L, 1998, J NEUROSCI RES, V54, P530, DOI 10.1002/(SICI)1097-4547(19981115)54:4<530::AID-JNR10>3.0.CO;2-1

Rabchevsky AG, 1997, J NEUROSCI RES, V47, P34

Sandhir R, 2004, NEUROSCI LETT, V369, P28, DOI 10.1016/j.neulet.2004.07.032

SCHEINMAN RI, 1995, SCIENCE, V270, P283, DOI 10.1126/science.270.5234.283

Schluesener HJ, 2001, J NEUROIMMUNOL, V113, P89, DOI 10.1016/S0165-5728(00)00428-8

Schluesener HJ, 1999, ACTA NEUROPATHOL, V97, P119, DOI 10.1007/s004010050964

Schluesener HJ, 1997, GLIA, V20, P365, DOI 10.1002/(SICI)1098-1136(199708)20:4<365::AID-GLIA8>3.0.CO;2-4

Schluesener HJ, 1998, GLIA, V24, P244, DOI 10.1002/(SICI)1098-1136(199810)24:2<244::AID-GLIA9>3.0.CO;2-3

Schwab JM, 2001, J NEUROIMMUNOL, V119, P214, DOI 10.1016/S0165-5728(01)00375-7

Schwab JM, 2005, J NEUROIMMUNOL, V163, P185, DOI 10.1016/j.jneuroim.2005.02.016

SHOHAMI E, 1994, J CEREBR BLOOD F MET, V14, P615, DOI 10.1038/jcbfm.1994.76

Tanaka J, 1997, GLIA, V20, P23, DOI 10.1002/(SICI)1098-1136(199705)20:1<23::AID-GLIA3>3.3.CO;2-U

Tanaka S, 1998, J NEUROSCI, V18, P6358

Tas MPR, 1996, INT J BIOCHEM CELL B, V28, P837, DOI 10.1016/1357-2725(96)00038-6

TAUPIN V, 1993, J NEUROIMMUNOL, V42, P177, DOI 10.1016/0165-5728(93)90008-M

UTANS U, 1995, J CLIN INVEST, V95, P2954, DOI 10.1172/JCI118003

Utans U, 1996, TRANSPLANTATION, V61, P1387, DOI 10.1097/00007890-199605150-00018

Wakasugi K, 1999, SCIENCE, V284, P147, DOI 10.1126/science.284.5411.147

Wennersten A, 2003, ACTA NEUROPATHOL, V105, P281, DOI 10.1007/s00401-002-0649-y

Yatsiv I, 2002, J CEREBR BLOOD F MET, V22, P971, DOI 10.1097/00004647-200208000-00008

Zhang XQ, 2006, PLASMA SCI TECHNOL, V8, P141, DOI 10.1088/1009-0630/8/2/04

Zhang ZR, 2006, EXP NEUROL, V197, P252, DOI 10.1016/j.expneurol.2005.09.015

NR 67

TC 48

Z9 52

U1 0

U2 12

PU SPRINGER

PI NEW YORK

PA ONE NEW YORK PLAZA, SUITE 4600, NEW YORK, NY, UNITED STATES

SN 0001-6322

EI 1432-0533

J9 ACTA NEUROPATHOL

JI Acta Neuropathol.

PD JUN

PY 2007

VL 113

IS 6

BP 675

EP 682

DI 10.1007/s00401-007-0195-8

PG 8

WC Clinical Neurology; Neurosciences; Pathology

WE Science Citation Index Expanded (SCI-EXPANDED)

SC Neurosciences & Neurology; Pathology

GA 171IO

UT WOS:000246729600006

PM 17265048

DA 2024-03-03

ER

PT J

AU Kurpius, D

Nolley, EP

Dailey, ME

AF Kurpius, Dana

Nolley, Eric P.

Dailey, Michael E.

TI Purines induce directed migration and rapid homing of microglia to

injured pyramidal neurons in developing hippocampus

SO GLIA

LA English

DT Article

DE brain slice; ATP; time-lapse; motility; migration; CNS trauma

ID RAT-BRAIN; IN-VITRO; EXTRACELLULAR NUCLEOTIDES; P2Y(12) RECEPTOR;

NERVOUS-SYSTEM; CELL-MIGRATION; SLICE CULTURES; GLIAL-CELLS; ACTIVATION;

EXPRESSION

AB Traumatic CNS injury activates and mobilizes resident parenchymal microglia (MG), which rapidly accumulate near injured neurons where they transform into phagocytes. The mechanisms underlying this rapid 'homing' in situ are unknown. Using time-lapse confocal imaging in acutely excised neonatal hippocampal slices, we show that rapid accumulation of MG near somata of injured pyramidal neurons in the stratum pyramidale (SP) results from directed migration from tissue regions immediately adjacent to (< 200 mu m from) the SP. Time-lapse sequences also reveal a 'spreading activation wave' wherein MG situated progressively farther from the SP begin to migrate later and exhibit less directional migration toward the SP Because purines have been implicated in MG activation and chemotaxis, we tested whether ATP/ADP released from injured pyramidal neurons might account for these patterns of MG behavior. Indeed, application of apyrase, which degrades extracellular ATP/ADP, inhibits MG motility and homing to injured neurons in the SP Moreover, bath application of exogenous ATP/ADP disrupts MG homing by inducing directional migration toward the slice exterior and away from injured neurons. These results indicate that extracellular ATP/ADP is both necessary and sufficient to induce directional migration and rapid homing of neonatal MG to injured neurons in situ. Rapid, ATP/ADP-dependent MG homing may promote clearance of dead and dying cells and help limit secondary damage during the critical first few hours after neuronal injury. (c) 2007 Wiley-Liss, Inc.

C1 Univ Iowa, Dept Biol Sci, Iowa City, IA 52242 USA.

C3 University of Iowa

RP Dailey, ME (corresponding author), Univ Iowa, Dept Biol Sci, 369 Biol Bldg, Iowa City, IA 52242 USA.

EM Michael-e-dailey@uiowa.edu

OI Dailey, Michael/0000-0003-2962-4661

FU NCRR NIH HHS [S10 RR017941] Funding Source: Medline; NINDS NIH HHS [R01

NS043468, NS43468] Funding Source: Medline

CR Bernardino L, 2005, J NEUROSCI, V25, P6734, DOI 10.1523/JNEUROSCI.1510-05.2005

Bianco F, 2005, BRAIN RES REV, V48, P144, DOI 10.1016/j.brainresrev.2004.12.004

Block ML, 2005, PROG NEUROBIOL, V76, P77, DOI 10.1016/j.pneurobio.2005.06.004

Boucsein C, 2003, EUR J NEUROSCI, V17, P2267, DOI 10.1046/j.1460-9568.2003.02663.x

Brockhaus J, 1996, GLIA, V16, P81, DOI 10.1002/(SICI)1098-1136(199601)16:1<81::AID-GLIA9>3.0.CO;2-E

Carbonell WS, 2005, J NEUROINFLAMM, V2, DOI 10.1186/1742-2094-2-5

Carbonell WS, 2005, J NEUROSCI, V25, P7040, DOI 10.1523/JNEUROSCI.5171-04.2005

Cross AK, 1999, J NEUROSCI RES, V55, P17, DOI 10.1002/(SICI)1097-4547(19990101)55:1<17::AID-JNR3>3.0.CO;2-J

DAILEY ME, 2005, IMAGING NEUROSCIENCE, P1

Dalmau I, 1998, J COMP NEUROL, V398, P333, DOI 10.1002/(SICI)1096-9861(19980831)398:3<333::AID-CNE3>3.0.CO;2-0

Davalos D, 2005, NAT NEUROSCI, V8, P752, DOI 10.1038/nn1472

Duan YL, 2003, J NEUROBIOL, V57, P183, DOI 10.1002/neu.10262

Elkabes S, 1996, J NEUROSCI, V16, P2508

Ferrari D, 1999, J BIOL CHEM, V274, P13205, DOI 10.1074/jbc.274.19.13205

Ferrari D, 1997, J CELL BIOL, V139, P1635, DOI 10.1083/jcb.139.7.1635

Fields RD, 2006, NAT REV NEUROSCI, V7, P423, DOI 10.1038/nrn1928

Franklin A, 2003, EUR J PHARMACOL, V474, P195, DOI 10.1016/S0014-2999(03)02074-0

Haas S, 1996, NEUROSCIENCE, V75, P257, DOI 10.1016/0306-4522(96)00270-9

Haynes SE, 2006, NAT NEUROSCI, V9, P1512, DOI 10.1038/nn1805

Heppner FL, 1998, EUR J NEUROSCI, V10, P3284, DOI 10.1046/j.1460-9568.1998.00379.x

Hollopeter G, 2001, NATURE, V409, P202, DOI 10.1038/35051599

Honda S, 2001, J NEUROSCI, V21, P1975, DOI 10.1523/JNEUROSCI.21-06-01975.2001

Inoue K, 2002, GLIA, V40, P156, DOI 10.1002/glia.10150

Kim JV, 2006, J IMMUNOL, V177, P5269, DOI 10.4049/jimmunol.177.8.5269

Kreutzberg GW, 1996, TRENDS NEUROSCI, V19, P312, DOI 10.1016/0166-2236(96)10049-7

KURPIUS D, 2005, IMAGING NEUROSCIENCE, P425

Kurpius D, 2006, GLIA, V54, P58, DOI 10.1002/glia.20355

Maciejewski-Lenoir D, 1999, J IMMUNOL, V163, P1628

Marella M, 2004, J NEUROSCI, V24, P620, DOI 10.1523/JNEUROSCI.4303-03.2004

MCGLADEMCCULLOH E, 1989, P NATL ACAD SCI USA, V86, P1093, DOI 10.1073/pnas.86.3.1093

McLarnon JG, 2005, J NEUROSCI RES, V81, P349, DOI 10.1002/jnr.20475

Möller T, 2000, BRAIN RES, V853, P49, DOI 10.1016/S0006-8993(99)02244-1

Nakajima Kazuyuki, 2004, Curr Drug Targets Cardiovasc Haematol Disord, V4, P65, DOI 10.2174/1568006043481284

Nasu-Tada K, 2005, GLIA, V52, P98, DOI 10.1002/glia.20224

Nicholas RA, 2001, MOL PHARMACOL, V60, P416

Nolte C, 1997, EUR J NEUROSCI, V9, P1690, DOI 10.1111/j.1460-9568.1997.tb01526.x

NORENBERG W, 1994, BRIT J PHARMACOL, V111, P942

Petersen MA, 2004, GLIA, V46, P195, DOI 10.1002/glia.10362

Raivich G, 1999, BRAIN RES REV, V30, P77, DOI 10.1016/S0165-0173(99)00007-7

Rappert A, 2002, J IMMUNOL, V168, P3221, DOI 10.4049/jimmunol.168.7.3221

Rappert A, 2004, J NEUROSCI, V24, P8500, DOI 10.1523/JNEUROSCI.2451-04.2004

Rathbone MP, 1999, PROG NEUROBIOL, V59, P663, DOI 10.1016/S0301-0082(99)00017-9

Sasaki Y, 2003, GLIA, V44, P242, DOI 10.1002/glia.10293

Schiefer J, 1999, J NEUROCYTOL, V28, P439, DOI 10.1023/A:1007048903862

Stence N, 2001, GLIA, V33, P256, DOI 10.1002/1098-1136(200103)33:3<256::AID-GLIA1024>3.0.CO;2-J

Stoll G, 2002, ADV EXP MED BIOL, V513, P87

STREIT WJ, 1987, J NEUROCYTOL, V16, P249, DOI 10.1007/BF01795308

Ullrich O, 2001, NAT CELL BIOL, V3, P1035, DOI 10.1038/ncb1201-1035

Walter L, 2003, J NEUROSCI, V23, P1398, DOI 10.1523/JNEUROSCI.23-04-01398.2003

Wang XH, 2004, NAT MED, V10, P821, DOI 10.1038/nm1082

Witting A, 2004, P NATL ACAD SCI USA, V101, P3214, DOI 10.1073/pnas.0306707101

Xiang ZH, 2005, GLIA, V52, P119, DOI 10.1002/glia.20227

YAO J, 1990, J NEUROSCI RES, V27, P36, DOI 10.1002/jnr.490270106

[No title captured]

NR 54

TC 66

Z9 79

U1 0

U2 3

PU WILEY-LISS

PI HOBOKEN

PA DIV JOHN WILEY & SONS INC, 111 RIVER ST, HOBOKEN, NJ 07030 USA

SN 0894-1491

J9 GLIA

JI Glia

PD JUN

PY 2007

VL 55

IS 8

BP 873

EP 884

DI 10.1002/glia.20509

PG 12

WC Neurosciences

WE Science Citation Index Expanded (SCI-EXPANDED)

SC Neurosciences & Neurology

GA 164IY

UT WOS:000246228300009

PM 17405148

DA 2024-03-03

ER

PT J

AU Ellis, EF

Willoughby, KA

Sparks, SA

Chen, T

AF Ellis, Earl F.

Willoughby, Karen A.

Sparks, Sallie A.

Chen, Tao

TI S100B protein is released from rat neonatal neurons, astrocytes, and

microglia by <i>in vitro</i> trauma and anti-S100 increases

trauma-induced delayed neuronal injury and negates the protective effect

of exogenous S100B on neurons

SO JOURNAL OF NEUROCHEMISTRY

LA English

DT Article

DE S100B; neuroprotection; traumatic brain injury

ID STRETCH-INDUCED INJURY; CULTURED ASTROCYTES; COGNITIVE RECOVERY;

EXPRESSION; INFUSION; DAMAGE; CELLS; ATP

AB S100B protein is found in brain, has been used as a marker for brain injury and is neurotrophic. Using a well-characterized in vitro model of brain cell trauma, we have previously shown that strain injury causes S100B release from neonatal rat neuronal plus glial cultures and that exogenous S100B reduces delayed post-traumatic neuronal damage even when given at 6 or 24 h post-trauma. The purpose of the current studies was to measure post-traumatic S100B release by specific brain cell types and to examine the effect of an antibody to S100 on post-traumatic delayed (48 h) neuronal injury and the protective effect of exogenous S100B. Neonatal rat cortical cells grown on a deformable elastic membrane were subjected to a strain (stretch) injury produced by a 50 ms displacement of the membrane. S100B was measured with an ELISA kit. Trauma released S100B from pure cultures of astrocytes, microglia, and neurons. Anti-S100 reduced released S100B to below detectable levels, increased delayed neuronal injury in traumatized cells and negated the protective effect of exogenous S100B on injured cells. Heat denatured anti-S100 did not exacerbate injury. These studies provide further evidence for a protective role for S100B following neuronal trauma.

C1 Virginia Commonwealth Univ, Sch Med, Dept Pharmacol & Toxicol, Richmond, VA 23298 USA.

C3 Virginia Commonwealth University

RP Ellis, EF (corresponding author), Virginia Commonwealth Univ, Sch Med, Dept Pharmacol & Toxicol, POB 980613, Richmond, VA 23298 USA.

EM efellis@vcu.edu

FU NINDS NIH HHS [NS07288, NS27214] Funding Source: Medline

CR Adami C, 2001, GLIA, V33, P131, DOI 10.1002/1098-1136(200102)33:2<131::AID-GLIA1012>3.0.CO;2-D

Ahlemeyer B, 2000, BRAIN RES, V858, P121, DOI 10.1016/S0006-8993(99)02438-5

Ahmed SM, 2000, J NEUROCHEM, V74, P1951, DOI 10.1046/j.1471-4159.2000.741951000000000.x

Azmitia EC, 2002, PROG BRAIN RES, V136, P87

BOYES BE, 1986, NEUROSCIENCE, V17, P857, DOI 10.1016/0306-4522(86)90050-3

Brewton LS, 2001, BRAIN RES, V912, P9, DOI 10.1016/S0006-8993(01)02519-7

Busto R, 1997, J NEUROTRAUM, V14, P35, DOI 10.1089/neu.1997.14.35

Donato R, 1999, BBA-MOL CELL RES, V1450, P191, DOI 10.1016/S0167-4889(99)00058-0

ELLIS EF, 1995, J NEUROTRAUM, V12, P325, DOI 10.1089/neu.1995.12.325

Geddes DM, 2003, J NEUROTRAUM, V20, P1039, DOI 10.1089/089771503770195885

Goforth PB, 1999, J NEUROSCI, V19, P7367, DOI 10.1523/JNEUROSCI.19-17-07367.1999

Heizmann CW, 2002, FRONT BIOSCI-LANDMRK, V7, pD1356, DOI 10.2741/heizmann

Hofmann MA, 1999, CELL, V97, P889, DOI 10.1111/j.1365-2222.2011.03714.x

Hu JR, 1997, J NEUROCHEM, V69, P2294

JONES KH, 1985, J HISTOCHEM CYTOCHEM, V33, P77, DOI 10.1177/33.1.2578146

Kao CQ, 2004, J NEUROTRAUM, V21, P259, DOI 10.1089/089771504322972059

Kleindienst A, 2005, J NEUROTRAUM, V22, P645, DOI 10.1089/neu.2005.22.645

Kleindienst A, 2004, J NEUROTRAUM, V21, P541, DOI 10.1089/089771504774129874

Kleindienst A, 2006, J NEUROTRAUM, V23, P1185, DOI 10.1089/neu.2006.23.1185

KLIGMAN D, 1985, P NATL ACAD SCI USA, V82, P7136, DOI 10.1073/pnas.82.20.7136

Lamb RG, 1997, J NEUROCHEM, V68, P1904

Marenholz I, 2004, BIOCHEM BIOPH RES CO, V322, P1111, DOI 10.1016/j.bbrc.2004.07.096

McKinney JS, 1996, STROKE, V27, P934, DOI 10.1161/01.STR.27.5.934

MOORE BW, 1965, BIOCHEM BIOPH RES CO, V19, P739, DOI 10.1016/0006-291X(65)90320-7

Narayan RK, 2002, J NEUROTRAUM, V19, P503, DOI 10.1089/089771502753754037

Neary JT, 2005, J NEUROTRAUM, V22, P491, DOI 10.1089/neu.2005.22.491

Reali C, 2005, J NEUROSCI RES, V81, P677, DOI 10.1002/jnr.20584

Rothermundt M, 2003, MICROSC RES TECHNIQ, V60, P614, DOI 10.1002/jemt.10303

Rzigalinski BA, 1998, J NEUROCHEM, V70, P2377

Schreiber D., 1995, P 1995 INT RES C BIO, P233

SELINFREUND RH, 1991, P NATL ACAD SCI USA, V88, P3554, DOI 10.1073/pnas.88.9.3554

Tavalin SJ, 1995, J NEUROPHYSIOL, V74, P2767, DOI 10.1152/jn.1995.74.6.2767

Tolias Christos M, 2004, NeuroRx, V1, P71, DOI 10.1602/neurorx.1.1.71

Tran MD, 2006, P NATL ACAD SCI USA, V103, P9321, DOI 10.1073/pnas.0603146103

Van Eldik LJ, 2003, RESTOR NEUROL NEUROS, V21, P97

Weber JT, 1999, CELL CALCIUM, V26, P289, DOI 10.1054/ceca.1999.0082

WEBER JT, 2001, J NEUROTRAUM, V18, P1183

Willoughby KA, 2004, J NEUROCHEM, V91, P1284, DOI 10.1111/j.1471-4159.2004.02812.x

Zhang L, 1996, SCIENCE, V274, P1921, DOI 10.1126/science.274.5294.1921

NR 39

TC 51

Z9 69

U1 0

U2 9

PU WILEY

PI HOBOKEN

PA 111 RIVER ST, HOBOKEN 07030-5774, NJ USA

SN 0022-3042

EI 1471-4159

J9 J NEUROCHEM

JI J. Neurochem.

PD JUN

PY 2007

VL 101

IS 6

BP 1463

EP 1470

DI 10.1111/j.1471-4159.2007.04515.x

PG 8

WC Biochemistry & Molecular Biology; Neurosciences

WE Science Citation Index Expanded (SCI-EXPANDED)

SC Biochemistry & Molecular Biology; Neurosciences & Neurology

GA 177EB

UT WOS:000247135300003

PM 17403138

DA 2024-03-03

ER

PT J

AU Obenaus, A

Robbins, M

Blanco, G

Galloway, NR

Snissarenko, E

Gillard, E

Lee, S

Currás-Collazo, M

AF Obenaus, Andre

Robbins, Michael

Blanco, Glen

Galloway, Nicholas R.

Snissarenko, Eugene

Gillard, Elizabeth

Lee, Stefan

Curras-Collazo, Margarita

TI Multi-modal magnetic resonance imaging alterations in two rat models of

mild neurotrauma

SO JOURNAL OF NEUROTRAUMA

LA English

DT Article

DE immunohistochemistry; apparent diffusion coefficient; T2 weighted

imaging; fluid percussion; controlled cortical impact; traumatic brain

injury; microglia; astrocytes

ID TRAUMATIC BRAIN-INJURY; LATERAL FLUID PERCUSSION; CORTICAL IMPACT

INJURY; CLOSED-HEAD INJURY; TIME-COURSE; DIFFUSION; EDEMA; HISTOLOGY;

HIPPOCAMPUS; PERFUSION

AB Magnetic resonance imaging (MRI) is increasingly used in the assessment of the severity and progression of neurotrauma. We evaluated temporal and regional changes after mild fluid percussion (FPI) and controlled cortical impact (CCI) injury using T2-weighted-imaging (T2WI) and diffusion-weighted imaging (DWI) MRI over 7 days. Region of interest analysis of brain areas distant to the injury site (such as the hippocampus, retrosplenial and piriform cortices, and the thalamus) was undertaken. In the hippocampus of CCI animals, we found a slow increase (51%) in apparent diffusion coefficients (ADC) over 72 h, which returned to control values. The hippocampal T2 values in the CCI animals were elevated by 18% over the 7-day time course compared to control, indicative of edema formation. Histological analysis supported the lack of overt cellular loss in most brain regions after mild CCI injury. FPI animals showed a generalized decrease in hippocampal ADC values over the first 72 h, which then returned to sham levels, with decreased T2 values over the same period, which remained depressed at 7 days. Histological assessment of FPI animals revealed numerous shrunken cells in the hippocampus and thalamus, but other regions showed little damage. Increased immunohistochemical staining for microglia and astroglia at 7 days post-injury was greater in FPI animals within the affected brain regions. In summary, traumatic brain injury is less severe in mild CCI than FPI, based on the temporal events assessed with MRI.

C1 Loma Linda Univ, Dept Radiat Med, Radiobiol Program, Loma Linda, CA 92354 USA.

Univ Calif Riverside, Dept Cell Biol & Neurosci, Riverside, CA USA.

Univ Calif Los Angeles, Div Neurosurg, Brain Injury Res Ctr, Los Angeles, CA USA.

C3 Loma Linda University; University of California System; University of

California Riverside; University of California System; University of

California Los Angeles

RP Obenaus, A (corresponding author), Loma Linda Univ, Dept Radiat Med, Radiobiol Program, 11175 Campus St,CSP A1010, Loma Linda, CA 92354 USA.

EM aobenaus@dominion.llumc.edu

RI Obenaus, Andre/KCJ-6964-2024

CR Albensi BC, 2000, EXP NEUROL, V162, P61, DOI 10.1006/exnr.2000.7256

Alsop DC, 1996, J NEUROTRAUM, V13, P515, DOI 10.1089/neu.1996.13.515

Assaf Y, 1997, MAGN RESON IMAGING, V15, P77, DOI 10.1016/S0730-725X(96)00246-9

Assaf Y, 1999, J NEUROTRAUM, V16, P1165, DOI 10.1089/neu.1999.16.1165

Babikian T, 2005, PEDIATR NEUROL, V33, P184, DOI 10.1016/j.pediatrneurol.2005.03.015

Baldwin SA, 1996, J NEUROSURG, V85, P476, DOI 10.3171/jns.1996.85.3.0476

Bartnik BL, 2005, J NEUROTRAUM, V22, P1052, DOI 10.1089/neu.2005.22.1052

Barzo P, 1997, J NEUROSURG, V87, P900, DOI 10.3171/jns.1997.87.6.0900

Chen S, 2003, EXP NEUROL, V182, P87, DOI 10.1016/S0014-4886(03)00002-5

Faden AI, 2005, NEUROPHARMACOLOGY, V49, P410, DOI 10.1016/j.neuropharm.2005.04.001

Floyd CL, 2002, J NEUROTRAUM, V19, P303, DOI 10.1089/089771502753594873

Grady MS, 2003, J NEUROTRAUM, V20, P929, DOI 10.1089/089771503770195786

HANSTOCK CC, 1994, STROKE, V25, P843, DOI 10.1161/01.STR.25.4.843

Huisman TAGM, 2003, EUR RADIOL, V13, P2283, DOI 10.1007/s00330-003-1843-6

Kadri Munaf, 2003, J Perinatol, V23, P181, DOI 10.1038/sj.jp.7210913

Kato H, 1986, No To Shinkei, V38, P295

Li HH, 2004, J NEUROTRAUM, V21, P1141, DOI 10.1089/0897715041953777

Maeda T, 2005, J NEUROTRAUM, V22, P763, DOI 10.1089/neu.2005.22.763

Osteen CL, 2001, J NEUROTRAUM, V18, P141, DOI 10.1089/08977150150502587

Pirko Istvan, 2005, NeuroRx, V2, P250, DOI 10.1007/BF03206670

Rodriguez-Paez AC, 2005, ACTA NEUROPATHOL, V109, P603, DOI 10.1007/s00401-005-1010-z

Schuhmann MU, 2003, J NEUROTRAUM, V20, P725, DOI 10.1089/089771503767869962

SMITH DH, 1995, J NEUROTRAUM, V12, P573, DOI 10.1089/neu.1995.12.573

Statler KD, 2000, J NEUROTRAUM, V17, P1179, DOI 10.1089/neu.2000.17.1179

Stroop R, 1998, ACT NEUR S, V71, P303

Thomas DL, 2000, PHYS MED BIOL, V45, pR97, DOI 10.1088/0031-9155/45/8/201

Thompson HJ, 2005, J NEUROTRAUM, V22, P42, DOI 10.1089/neu.2005.22.42

Tong KA, 2004, ANN NEUROL, V56, P36, DOI 10.1002/ana.20123

Unterberg AW, 1997, ACT NEUR S, V70, P106

Unterberg AW, 2004, NEUROSCIENCE, V129, P1021, DOI 10.1016/j.neuroscience.2004.06.046

van Pul C, 2005, AM J NEURORADIOL, V26, P469

Van Putten HP, 2005, J NEUROTRAUM, V22, P857

Vorísek I, 2002, MAGNET RESON MED, V48, P994, DOI 10.1002/mrm.10305

Wall CJ, 2000, AM J NEURORADIOL, V21, P1841

WAXWEILER RJ, 1995, J NEUROTRAUM, V12, P509, DOI 10.1089/neu.1995.12.509

Zanier ER, 2003, J NEUROTRAUM, V20, P409, DOI 10.1089/089771503765355496

NR 36

TC 57

Z9 64

U1 0

U2 5

PU MARY ANN LIEBERT INC

PI NEW ROCHELLE

PA 140 HUGUENOT STREET, 3RD FL, NEW ROCHELLE, NY 10801 USA

SN 0897-7151

J9 J NEUROTRAUM

JI J. Neurotrauma

PD JUL

PY 2007

VL 24

IS 7

BP 1147

EP 1160

DI 10.1089/neu.2006.0211

PG 14

WC Critical Care Medicine; Clinical Neurology; Neurosciences

WE Science Citation Index Expanded (SCI-EXPANDED)

SC General & Internal Medicine; Neurosciences & Neurology

GA 186DV

UT WOS:000247760200006

PM 17610354

DA 2024-03-03

ER

PT J

AU Zhang, ZY

Zhang, Z

Fauser, U

Artelt, M

Burnet, M

Schluesener, HJ

AF Zhang, Z.-Y.

Zhang, Z.

Fauser, U.

Artelt, M.

Burnet, M.

Schluesener, H. J.

TI Dexamethasone transiently attenuates up-regulation of

endostatin/collagen XVIII following traumatic brain injury

SO NEUROSCIENCE

LA English

DT Article

DE traumatic brain injury; endostatin/collagen XVIII;

microglia/macrophages; dexamethasone

ID NECROSIS-FACTOR-ALPHA; CLOSED-HEAD INJURY; ANGIOGENESIS INHIBITOR;

TUMOR-GROWTH; EXPRESSION; VEGF; RAT; DEGENERATION; CONTUSIONS; PROTEINS

AB Endostatin/collagen XVIII is a specific inhibitor of endothelial proliferation and migration in vitro. It has also been shown to have anti-angiogenic activity and tumor growth inhibitory activity in vivo and in vitro. Here we studied expression of endostatin/collagen XVIII in a rat traumatic brain injury (TBI) model, focusing on the early phase. A significant up-regulation of endostatin/collagen XVIII in TBI began as early as 24 h post-TBI. Double-staining experiment revealed that the major resource of endostatin/collagen XVIII+ cells in our TBI rat model was a subpopulation of reactivated microglia/macrophages. Our data further showed that dexamethasone attenuated up-regulation of endostatin/collagen XVIII expression at days 1 and 2, but not at day 4, post-TBI, indicating that dexamethasone might possess an early and transient influence to the angiogenesis following TBI. (c) 2007 IBRO. Published by Elsevier Ltd. All rights reserved.

C1 Univ Tubingen, Inst Brain Res, D-72076 Tubingen, Germany.

Synovo GmbH, D-72076 Tubingen, Germany.

C3 Eberhard Karls University of Tubingen; Synovo GmbH

RP Zhang, Z (corresponding author), Univ Tubingen, Inst Brain Res, Calwer Str 3, D-72076 Tubingen, Germany.

EM zhangzhiren@yahoo.com

RI Zhang, Zhiren/O-1012-2019; Burnet, Michael/AAB-2958-2019; zhang,

zhiyuan/GRS-2141-2022

OI Zhang, Zhiren/0000-0002-5238-2835; Burnet, Michael/0000-0003-4311-5441;

Zhang, Zhi-Yuan/0000-0001-9067-0670

CR Berger AC, 2000, J SURG RES, V91, P26, DOI 10.1006/jsre.2000.5890

Bix G, 2005, TRENDS CELL BIOL, V15, P52, DOI 10.1016/j.tcb.2004.11.008

Bloch W, 2000, FASEB J, V14, P2373

Deininger MH, 2006, J NEUROTRAUM, V23, P1103, DOI 10.1089/neu.2006.23.1103

Deininger MH, 2003, FASEB J, V17, P1267, DOI 10.1096/fj.02-1118com

Deininger MH, 2002, J NEUROSCI, V22, P10621

Dhanabal M, 1999, J BIOL CHEM, V274, P11721, DOI 10.1074/jbc.274.17.11721

Fan L, 1996, MOL BRAIN RES, V36, P287, DOI 10.1016/0169-328X(95)00274-V

FEENEY DM, 1981, BRAIN RES, V211, P67, DOI 10.1016/0006-8993(81)90067-6

Ferreras M, 2000, FEBS LETT, V486, P247, DOI 10.1016/S0014-5793(00)02249-3

Fukai N, 2002, EMBO J, V21, P1535, DOI 10.1093/emboj/21.7.1535

Gahm C, 2005, ACTA NEUROCHIR, V147, P1071, DOI 10.1007/s00701-005-0590-7

Golarai G, 2001, J NEUROSCI, V21, P8523, DOI 10.1523/JNEUROSCI.21-21-08523.2001

Gottesfeld Z, 2002, J NEUROTRAUM, V19, P317, DOI 10.1089/089771502753594882

Gourin CG, 1997, J TRAUMA, V42, P1101, DOI 10.1097/00005373-199706000-00020

Hajitou A, 2002, FASEB J, V16, P1802, DOI 10.1096/fj.02-0109fje

HALL ED, 1987, J PHARMACOL EXP THER, V242, P137

Hausmann R, 2000, INT J LEGAL MED, V113, P288, DOI 10.1007/s004149900126

Holmin, 1996, ACTA NEUROCHIR, V138, P418, DOI 10.1007/BF01420304

Karumanchi SA, 2001, MOL CELL, V7, P811, DOI 10.1016/S1097-2765(01)00225-8

Kim YM, 2002, J BIOL CHEM, V277, P27872, DOI 10.1074/jbc.M202771200

Knoblach SM, 1999, J NEUROIMMUNOL, V95, P115, DOI 10.1016/S0165-5728(98)00273-2

Leifeld L, 2002, J HEPATOL, V37, P613, DOI 10.1016/S0168-8278(02)00271-4

Marneros AG, 2001, MATRIX BIOL, V20, P337, DOI 10.1016/S0945-053X(01)00151-2

MUELLER CA, 2007, IN PRESS LESIONAL EX

MURAGAKI Y, 1995, P NATL ACAD SCI USA, V92, P8763, DOI 10.1073/pnas.92.19.8763

Muzha I, 2004, LANCET, V364, P1321, DOI 10.1016/S0140-6736(04)17188-2

OReilly MS, 1997, CELL, V88, P277, DOI 10.1016/S0092-8674(00)81848-6

Pulliam L, 1998, J NEUROSCI RES, V54, P530, DOI 10.1002/(SICI)1097-4547(19981115)54:4<530::AID-JNR10>3.0.CO;2-1

Sauerland S, 2004, LANCET, V364, P1291, DOI 10.1016/S0140-6736(04)17202-4

Sbarbati A, 1996, ACTA NEUROPATHOL, V92, P56, DOI 10.1007/s004010050489

SHOHAMI E, 1994, J CEREBR BLOOD F MET, V14, P615, DOI 10.1038/jcbfm.1994.76

Sköld MK, 2006, EUR J NEUROSCI, V23, P21, DOI 10.1111/j.1460-9568.2005.04527.x

Sköld MK, 2005, J NEUROTRAUM, V22, P353, DOI 10.1089/neu.2005.22.353

Sudhakar A, 2003, P NATL ACAD SCI USA, V100, P4766, DOI 10.1073/pnas.0730882100

Takahashi K, 2003, FASEB J, V17, P896, DOI 10.1096/fj.02-0824fje

TATOR CH, 1991, J NEUROSURG, V75, P15, DOI 10.3171/jns.1991.75.1.0015

TAUPIN V, 1993, J NEUROIMMUNOL, V42, P177, DOI 10.1016/0165-5728(93)90008-M

Walski M, 2003, ACTA NEUROBIOL EXP, V63, P77, DOI 10.55782/ane-2003-1457

Yamaguchi N, 1999, EMBO J, V18, P4414, DOI 10.1093/emboj/18.16.4414

Yatsiv I, 2002, J CEREBR BLOOD F MET, V22, P971, DOI 10.1097/00004647-200208000-00008

Zhang ZY, 2007, ACTA NEUROPATHOL, V113, P675, DOI 10.1007/s00401-007-0195-8

Zhang ZR, 2006, EXP NEUROL, V197, P252, DOI 10.1016/j.expneurol.2005.09.015

NR 43

TC 10

Z9 12

U1 0

U2 2

PU PERGAMON-ELSEVIER SCIENCE LTD

PI OXFORD

PA THE BOULEVARD, LANGFORD LANE, KIDLINGTON, OXFORD OX5 1GB, ENGLAND

SN 0306-4522

EI 1873-7544

J9 NEUROSCIENCE

JI Neuroscience

PD JUL 13

PY 2007

VL 147

IS 3

BP 720

EP 726

DI 10.1016/j.neuroscience.2007.04.052

PG 7

WC Neurosciences

WE Science Citation Index Expanded (SCI-EXPANDED)

SC Neurosciences & Neurology

GA 191QA

UT WOS:000248144900017

PM 17560042

DA 2024-03-03

ER

PT J

AU Tlaskowitz, D

Vitek, MP

AF Tlaskowitz, Daniel

Vitek, Michael P.

TI Apolipoprotein E and neurological disease: therapeutic potential and

pharmacogenomic interactions

SO PHARMACOGENOMICS

LA English

DT Review

DE apolipoprotein E; astrocytes; microglia; multiple; sclerosis;

neuroinflammation; stroke; subarachnoid; hemorrhage; traumatic; brain

injury

ID E-DEFICIENT MICE; PERMANENT FOCAL ISCHEMIA; GLOBAL CEREBRAL-ISCHEMIA;

CLOSED-HEAD INJURY; E EPSILON-4 ALLELE; E-BASED PEPTIDE; E GENOTYPE;

APOE GENOTYPE; ALZHEIMERS-DISEASE; E POLYMORPHISM

AB The apolipoprotein E (apoE) polymorphism is emerging as a uniquely important genetic modifier that affects functional outcome from both acute and chronic neurological injuries. Recent attention has focused on common denominator mechanisms by which apoE might affect brain injury and/or brain repair responses in clinically diverse diseases. Although endogenous apoE likely serves several adaptive functions in the injured CNS, there is growing evidence that its effect on modifying brain inflammatory responses and providing protection from excitotoxic injury may be central to its protective properties. A more complete understanding of the role that apoE plays in the injured brain has led to novel therapeutic strategies for both acute and chronic neurological disease.

C1 Duke Univ, Med Ctr, Dept Med Neurol, Durham, NC 27710 USA.

Duke Univ, Med Ctr, Dept Neurobiol, Durham, NC 27710 USA.

Cognosci Inc, Res Triangle Pk, NC 27709 USA.

C3 Duke University; Duke University

RP Tlaskowitz, D (corresponding author), Duke Univ, Med Ctr, Dept Med Neurol, Box 2900, Durham, NC 27710 USA.

EM danl@neuro.duke.edu

OI Vitek, Michael/0000-0001-8140-8048

CR AGGERBECK LP, 1988, J BIOL CHEM, V263, P6249

ALBERTS MJ, 1995, LANCET, V346, P575, DOI 10.1016/S0140-6736(95)91411-0

Aono M, 2003, NEUROSCIENCE, V116, P437, DOI 10.1016/S0306-4522(02)00709-1

Aono M, 2002, NEUROBIOL DIS, V11, P214, DOI 10.1006/nbdi.2002.0541

AVILA EM, 1982, J BIOL CHEM, V257, P5900

Bales K R, 2002, Mol Interv, V2, P363, DOI 10.1124/mi.2.6.363

Barger SW, 1997, NATURE, V388, P878, DOI 10.1038/42257

Bart RD, 1998, NEUROREPORT, V9, P2615, DOI 10.1097/00001756-199808030-00035

Beffert U, 2004, J LIPID RES, V45, P403, DOI 10.1194/jlr.R300017-JLR200

BELLOSTA S, 1995, J BIOL CHEM, V270, P27063, DOI 10.1074/jbc.270.45.27063

Blázquez L, 2006, NEUROSCI LETT, V406, P235, DOI 10.1016/j.neulet.2006.07.037

Brecht WJ, 2004, J NEUROSCI, V24, P2527, DOI 10.1523/JNEUROSCI.4315-03.2004

Brown CM, 2002, FREE RADICAL BIO MED, V32, P1071, DOI 10.1016/S0891-5849(02)00803-1

Burwick RM, 2006, NEUROLOGY, V66, P1373, DOI 10.1212/01.wnl.0000210531.19498.3f

Buttini M, 2000, NEUROSCIENCE, V97, P207, DOI 10.1016/S0306-4522(00)00069-5

Chang SJ, 2005, P NATL ACAD SCI USA, V102, P18694, DOI 10.1073/pnas.0508254102

Chapman J, 2001, NEUROLOGY, V56, P312, DOI 10.1212/WNL.56.3.312

Chen Y, 1997, NEUROSCIENCE, V80, P1255, DOI 10.1016/S0306-4522(97)00007-9

Chiang MF, 2003, ACTA NEUROCHIR, V145, P649, DOI 10.1007/s00701-003-0069-3

Colton CA, 2004, J NEUROIMMUNOL, V147, P62, DOI 10.1016/j.jneuroim.2003.10.015

Colton CA, 2002, ANN NY ACAD SCI, V962, P212, DOI 10.1111/j.1749-6632.2002.tb04070.x

CORDER EH, 1995, NEUROLOGY, V45, P1323, DOI 10.1212/WNL.45.7.1323

CORDER EH, 1993, SCIENCE, V261, P921, DOI 10.1126/science.8346443

Crawford FC, 2002, NEUROLOGY, V58, P1115, DOI 10.1212/WNL.58.7.1115

CURTISS LK, 1976, J IMMUNOL, V116, P1452

CURTISS LK, 1981, J IMMUNOL, V126, P1382

de Bont N, 1999, J LIPID RES, V40, P680

DONG LM, 1994, J BIOL CHEM, V269, P22358

Dong LM, 1996, J BIOL CHEM, V271, P19053, DOI 10.1074/jbc.271.32.19053

Drabe N, 2001, EUR J CARDIO-THORAC, V20, P609, DOI 10.1016/S1010-7940(01)00842-9

Dunn LT, 2001, NEUROSURGERY, V48, P1006, DOI 10.1097/00006123-200105000-00007

Egensperger R, 1998, BRAIN PATHOL, V8, P439

Ely EW, 2007, CRIT CARE MED, V35, P112, DOI 10.1097/01.CCM.0000251925.18961.CA

Fazekas F, 2001, NEUROLOGY, V57, P853, DOI 10.1212/WNL.57.5.853

Fleming LM, 1996, EXP NEUROL, V138, P252, DOI 10.1006/exnr.1996.0064

Friedman G, 1999, NEUROLOGY, V52, P244, DOI 10.1212/WNL.52.2.244

GALLO G, 1994, AM J PATHOL, V145, P526

Gao J, 2006, NEUROCRIT CARE, V4, P25, DOI 10.1385/NCC:4:1:025

Grocott HP, 2001, J THORAC CARDIOV SUR, V122, P622, DOI 10.1067/mtc.2001.115152

Grünenfelder J, 2004, HEART SURG FORUM, V7, pE8

Herz J, 2000, NAT REV NEUROSCI, V1, P51, DOI 10.1038/35036221

Hoane MR, 2007, J NEUROTRAUM, V24, P1108, DOI 10.1089/neu.2006.0254

Hoe HS, 2006, J BIOL CHEM, V281, P3425, DOI 10.1074/jbc.M509380200

Hoe HS, 2005, J NEUROCHEM, V93, P145, DOI 10.1111/j.1471-4159.2004.03007.x

Horsburgh K, 2000, J CEREBR BLOOD F MET, V20, P458, DOI 10.1097/00004647-200003000-00003

Horsburgh K, 1999, NEUROREPORT, V10, P837, DOI 10.1097/00001756-199903170-00031

Hu JR, 1998, J NEUROCHEM, V71, P1626

Huang XM, 2006, ARCH NEUROL-CHICAGO, V63, P189, DOI 10.1001/archneur.63.2.189

Huang YD, 2004, J MOL NEUROSCI, V23, P189, DOI 10.1385/JMN:23:3:189

Huang YD, 2001, P NATL ACAD SCI USA, V98, P8838, DOI 10.1073/pnas.151254698

HUI DY, 1980, J BIOL CHEM, V255, P1775

Jiang Y, 2006, NEUROSCI LETT, V408, P155, DOI 10.1016/j.neulet.2006.08.082

Jordan BD, 1997, JAMA-J AM MED ASSOC, V278, P136, DOI 10.1001/jama.278.2.136

Kantarci OH, 2004, NEUROLOGY, V62, P811, DOI 10.1212/01.WNL.0000113721.83287.83

Kutner KC, 2000, NEUROSURGERY, V47, P651, DOI 10.1097/00006123-200009000-00026

Lanterna LA, 2005, NEUROLOGY, V64, P1238, DOI 10.1212/01.WNL.0000156523.77347.B4

Laskowitz DT, 2006, ACTA NEUROL SCAND, V114, P15, DOI 10.1111/j.1600-0404.2006.00680.x

Laskowitz DT, 2007, J NEUROTRAUM, V24, P1093, DOI 10.1089/neu.2006.0192

Laskowitz DT, 2000, J LIPID RES, V41, P613

Laskowitz DT, 1997, J CEREBR BLOOD F MET, V17, P753

Laskowitz DT, 1997, J NEUROIMMUNOL, V76, P70, DOI 10.1016/S0165-5728(97)00021-0

Laskowitz DT, 2001, EXP NEUROL, V167, P74, DOI 10.1006/exnr.2001.7541

Laskowitz DT, 1998, NEUROREPORT, V9, P615, DOI 10.1097/00001756-199803090-00010

Laskowitz DT, 1998, J CEREBR BLOOD F MET, V18, P465, DOI 10.1097/00004647-199805000-00001

Leung CHS, 2002, STROKE, V33, P548, DOI 10.1161/hs0202.102326

Li FQ, 2006, J PHARMACOL EXP THER, V318, P956, DOI 10.1124/jpet.106.103671

Liaquat I, 2002, J NEUROSURG, V96, P90, DOI 10.3171/jns.2002.96.1.0090

Lichtman SW, 2000, NEUROLOGY, V55, P1536, DOI 10.1212/WNL.55.10.1536

LINTON MF, 1991, J CLIN INVEST, V88, P270, DOI 10.1172/JCI115288

Lomnitski L, 1999, BBA-MOL BASIS DIS, V1453, P359, DOI 10.1016/S0925-4439(99)00010-1

Lynch JR, 2003, J BIOL CHEM, V278, P48529, DOI 10.1074/jbc.M306923200

Lynch JR, 2002, ANN NEUROL, V51, P113, DOI 10.1002/ana.10098

Lynch JR, 2005, EXP NEUROL, V192, P109, DOI 10.1016/j.expneurol.2004.11.014

Maezawa I, 2006, J NEUROINFLAMM, V3, DOI 10.1186/1742-2094-3-21

Mahley RW, 2006, P NATL ACAD SCI USA, V103, P5644, DOI 10.1073/pnas.0600549103

Martínez-González NA, 2006, J NEUROL NEUROSUR PS, V77, P1329, DOI 10.1136/jnnp.2006.097543

McAdoo JD, 2005, NEUROSCI LETT, V381, P305, DOI 10.1016/j.neulet.2005.02.036

McCarron MO, 2003, ACTA NEUROL SCAND, V107, P106, DOI 10.1034/j.1600-0404.2003.01365.x

Mesis Rachel G, 2006, Neurosurg Focus, V21, pE4

Misra UK, 2001, J LEUKOCYTE BIOL, V70, P677

Miyata M, 1996, NAT GENET, V14, P55, DOI 10.1038/ng0996-55

Moretti EW, 2005, CRIT CARE MED, V33, P2521, DOI 10.1097/01.CCM.0000186368.96146.FB

Mori T, 2005, J CEREBR BLOOD F MET, V25, P748, DOI 10.1038/sj.jcbfm.9600063

Mori T, 2004, J CEREBR BLOOD F MET, V24, P646, DOI 10.1097/01.WCB.0000120787.53851.A4

Morris PG, 2004, ACTA NEUROL SCAND, V109, P205, DOI 10.1034/j.1600-0404.2003.00206.x

Naidu A, 2002, BRAIN RES, V958, P100, DOI 10.1016/S0006-8993(02)03480-7

NATHAN BP, 1994, SCIENCE, V264, P850, DOI 10.1126/science.8171342

Nathoo N, 2003, J CLIN PATHOL-MOL PA, V56, P132, DOI 10.1136/mp.56.3.132

NICOLL JAR, 1995, NAT MED, V1, P135, DOI 10.1038/nm0295-135

Niskakangas T, 2001, STROKE, V32, P1181, DOI 10.1161/01.STR.32.5.1181

Ophir G, 2005, NEUROBIOL DIS, V20, P709, DOI 10.1016/j.nbd.2005.05.002

Overmyer M, 1999, ACTA NEUROPATHOL, V97, P383, DOI 10.1007/s004010051002

PEPE MG, 1986, J IMMUNOL, V136, P3716

PIEDRAHITA JA, 1992, P NATL ACAD SCI USA, V89, P4471, DOI 10.1073/pnas.89.10.4471

Pinholt M, 2005, MULT SCLER J, V11, P511, DOI 10.1191/1352458505ms1207oa

Raffaï RL, 2001, P NATL ACAD SCI USA, V98, P11587, DOI 10.1073/pnas.201279298

RAJAVASHISTH TB, 1985, P NATL ACAD SCI USA, V82, P8085, DOI 10.1073/pnas.82.23.8085

RALL SC, 1982, J BIOL CHEM, V257, P4171

Risner ME, 2006, PHARMACOGENOMICS J, V6, P246, DOI 10.1038/sj.tpj.6500369

Roselaar SE, 1998, J LIPID RES, V39, P1740

Roses AD, 2004, NAT REV GENET, V5, P645, DOI 10.1038/nrg1432

Sabo T, 2000, NEUROSCIENCE, V101, P879, DOI 10.1016/S0306-4522(00)00438-3

Schiefermeier M, 2000, STROKE, V31, P2068, DOI 10.1161/01.STR.31.9.2068

SCHMECHEL DE, 1993, P NATL ACAD SCI USA, V90, P9649, DOI 10.1073/pnas.90.20.9649

Sheng HX, 1998, J CEREBR BLOOD F MET, V18, P361, DOI 10.1097/00004647-199804000-00003

Sheng HX, 1999, STROKE, V30, P1118, DOI 10.1161/01.STR.30.5.1118

STRITTMATTER WJ, 1994, P NATL ACAD SCI USA, V91, P11183, DOI 10.1073/pnas.91.23.11183

Sullivan PM, 1997, J BIOL CHEM, V272, P17972, DOI 10.1074/jbc.272.29.17972

Tang J, 2003, SURG NEUROL, V60, P391, DOI 10.1016/S0090-3019(03)00323-9

Tardiff BE, 1997, ANN THORAC SURG, V64, P715, DOI 10.1016/S0003-4975(97)00757-1

Teasdale GM, 1997, LANCET, V350, P1069, DOI 10.1016/S0140-6736(97)04318-3

UTERMANN G, 1980, AM J HUM GENET, V32, P339

Van Oosten MV, 2001, J BIOL CHEM, V276, P8820, DOI 10.1074/jbc.M009915200

Vitek MP, 1997, BIOCHEM BIOPH RES CO, V240, P391, DOI 10.1006/bbrc.1997.7408

Wang H, 2007, NEUROSCIENCE, V144, P1324, DOI 10.1016/j.neuroscience.2006.11.017

WEISGRABER KH, 1994, ADV PROTEIN CHEM, V45, P249

WELLONS JC, 2000, NEUROSURGERY, V47

Xu Q, 2006, J NEUROSCI, V26, P4985, DOI 10.1523/JNEUROSCI.5476-05.2006

Ye SM, 2005, P NATL ACAD SCI USA, V102, P18700, DOI 10.1073/pnas.0508693102

Zerbinatti CV, 2005, REV NEUROSCIENCE, V16, P123

NR 120

TC 50

Z9 56

U1 0

U2 3

PU FUTURE MEDICINE LTD

PI LONDON

PA UNITEC HOUSE, 3RD FLOOR, 2 ALBERT PLACE, FINCHLEY CENTRAL, LONDON, N3

1QB, ENGLAND

SN 1462-2416

EI 1744-8042

J9 PHARMACOGENOMICS

JI Pharmacogenomics

PD AUG

PY 2007

VL 8

IS 8

BP 959

EP 969

DI 10.2217/14E22416.8.8.959

PG 11

WC Pharmacology & Pharmacy

WE Science Citation Index Expanded (SCI-EXPANDED)

SC Pharmacology & Pharmacy

GA 208YS

UT WOS:000249355700014

PM 17716229

DA 2024-03-03

ER

PT J

AU Venneti, S

Wagner, AK

Wang, G

Slagel, SL

Chen, X

Lopresti, BJ

Mathis, CA

Wiley, CA

AF Venneti, Sriram

Wagner, Amy K.

Wang, Guoji

Slagel, Susan L.

Chen, Xiangbal

Lopresti, Brian J.

Mathis, Chester A.

Wiley, Clayton A.

TI The high affinity peripheral benzodiazepine receptor ligand DAA1106

binds specifically to microglia in a rat model of traumatic brain

injury: Implications for PET imaging

SO EXPERIMENTAL NEUROLOGY

LA English

DT Article

DE peripheral benzodiazepine receptor; microglia; PET imaging; traumatic

brain injury; DAA1106; PK11195

ID POSITRON-EMISSION-TOMOGRAPHY; CONTROLLED CORTICAL IMPACT; MILD COGNITIVE

IMPAIRMENT; IN-VIVO; ISCHEMIC-STROKE; ACTIVATED MICROGLIA;

MULTIPLE-SCLEROSIS; PARKINSONS-DISEASE; PK11195 BINDING; MOUSE-BRAIN

AB Traumatic brain injury (TBI) is a significant cause of mortality, morbidity, and disability. Microglial activation is commonly observed in response to neuronal injury which, when prolonged, is thought to be detrimental to neuronal survival. Activated microglia can be labeled using PK11195, a ligand that binds the peripheral benzodiazepine receptor (PBR), receptors which are increased in activated microglia and sparse in the resting brain. We compared the binding properties of two PBR ligands PK11195 and DAA1106 in rats using the controlled cortical impact (CCI) model of experimental TBI. While both ligands showed relative increases with specific binding in the cortex ipsitateral to injury compared to the contralateral side, [H-3]DAA1106 showed higher binding affinity compared with [H-3](R)-PK11195. Combined immunohistochemistry and autoradiography in brain tissues near the injury site showed that [H-3]DAA1106 binding co-registered with activated microglia more than astrocytes. Further, increased [H-3]DAA1106-specific binding positively correlated with the degree of microglial activation, and to a lesser degree with reactive astrocytosis. Finally, in vivo administration of each ligand in rats with TBI showed greater retention of [C-11]DAA1106 compared to [C-11](R)-PK11195 at the site of the contusion as assessed by ex vivo autoradiography. These results in a rat model of TBI indicate that [C-11] DAA1106 binds with higher affinity to microglia when compared with PK11195, suggesting that [C-11]DAA1106 may represent a better ligand than [C-11](R)-PK11195 for in vivo PET imaging of activated microglia in TBI. (c) 2007 Elsevier Inc. All rights reserved.

C1 Univ Pittsburgh, Sch Med, Dept Pathol, Pittsburgh, PA 15213 USA.

Univ Pittsburgh, Sch Med, Dept Phys Med & Rehabil, Pittsburgh, PA 15213 USA.

Univ Pittsburgh, Sch Med, Dept Safar Ctr Resuscitat Res, Pittsburgh, PA 15213 USA.

Univ Pittsburgh, Sch Med, Dept Radiol, Pittsburgh, PA 15213 USA.

C3 Pennsylvania Commonwealth System of Higher Education (PCSHE); University

of Pittsburgh; Pennsylvania Commonwealth System of Higher Education

(PCSHE); University of Pittsburgh; Pennsylvania Commonwealth System of

Higher Education (PCSHE); University of Pittsburgh; Pennsylvania

Commonwealth System of Higher Education (PCSHE); University of

Pittsburgh

RP Wiley, CA (corresponding author), Presbyterian Univ Hosp, Div Neuropathol, 200 Lothrop St A515, Pittsburgh, PA 15213 USA.

EM wiley1@pitt.edu

RI Mathis, Chester/A-8607-2009

OI Mathis, Chester/0000-0001-9811-0950

FU NICHD NIH HHS [K08HD40833, K08 HD040833] Funding Source: Medline; NIMH

NIH HHS [R01 MH64921, R01 MH064921, K24 MH01717, R01 MH071151, K24

MH001717, R01 MH071151-03, K24 MH001717-08] Funding Source: Medline

CR Banati RB, 2002, GLIA, V40, P206, DOI 10.1002/glia.10144

Banati RB, 2000, BRAIN, V123, P2321, DOI 10.1093/brain/123.11.2321

Bellander BM, 2004, J NEUROTRAUM, V21, P605, DOI 10.1089/089771504774129937

Beschorner R, 2002, ACTA NEUROPATHOL, V103, P541, DOI 10.1007/s00401-001-0503-7

Block ML, 2007, NAT REV NEUROSCI, V8, P57, DOI 10.1038/nrn2038

BONTKE CF, 1996, PHYS MED REHABILITAT, P1027

Cagnin A, 2002, EUR NEUROPSYCHOPHARM, V12, P581, DOI 10.1016/S0924-977X(02)00107-4

Cagnin A, 2001, LANCET, V358, P461, DOI 10.1016/S0140-6736(01)05625-2

Casellas P, 2002, NEUROCHEM INT, V40, P475, DOI 10.1016/S0197-0186(01)00118-8

Chaki S, 1999, EUR J PHARMACOL, V371, P197, DOI 10.1016/S0014-2999(99)00118-1

Chen MK, 2004, BRAIN, V127, P1379, DOI 10.1093/brain/awh161

Culty M, 2001, DRUG DEVELOP RES, V52, P475, DOI 10.1002/ddr.1149

Davalos D, 2005, NAT NEUROSCI, V8, P752, DOI 10.1038/nn1472

Debruyne JC, 2003, EUR J NEUROL, V10, P257, DOI 10.1046/j.1468-1331.2003.00571.x

Dihné M, 2001, BRAIN RES, V902, P178, DOI 10.1016/S0006-8993(01)02378-2

DIXON CE, 1991, J NEUROSCI METH, V39, P253

Dommergues MA, 2003, NEUROSCIENCE, V121, P619, DOI 10.1016/S0306-4522(03)00558-X

Engel S, 2000, ACTA NEUROPATHOL, V100, P313, DOI 10.1007/s004019900172

Fetler L, 2005, SCIENCE, V309, P392, DOI 10.1126/science.1114852

Gerhard A, 2000, NEUROREPORT, V11, P2957, DOI 10.1097/00001756-200009110-00025

Gerhard A, 2005, NEUROIMAGE, V24, P591, DOI 10.1016/j.neuroimage.2004.09.034

Gerhard A, 2006, NEUROBIOL DIS, V21, P404, DOI 10.1016/j.nbd.2005.08.002

GIORDANA MT, 1994, NEUROPATH APPL NEURO, V20, P163, DOI 10.1111/j.1365-2990.1994.tb01175.x

GROOM GN, 1995, J NUCL MED, V36, P2207

Hammoud DA, 2005, J NEUROVIROL, V11, P346, DOI 10.1080/13550280500187351

Igarashi T, 2001, EXP NEUROL, V172, P332, DOI 10.1006/exnr.2001.7820

Ikoma Y, 2007, J CEREBR BLOOD F MET, V27, P173, DOI 10.1038/sj.jcbfm.9600325

Kline AE, 2000, NEUROSCI LETT, V280, P163, DOI 10.1016/S0304-3940(00)00797-7

Kline AE, 2002, J NEUROTRAUM, V19, P415, DOI 10.1089/08977150252932370

Kreutzberg GW, 1996, TRENDS NEUROSCI, V19, P312, DOI 10.1016/0166-2236(96)10049-7

Kuhlmann AC, 2000, J NEUROCHEM, V74, P1694, DOI 10.1046/j.1471-4159.2000.0741694.x

Maeda J, 2004, SYNAPSE, V52, P283, DOI 10.1002/syn.20027

Mankowski JL, 2003, J NEUROVIROL, V9, P94, DOI 10.1080/13550280390173283

MINTUN MA, 1984, ANN NEUROL, V15, P217, DOI 10.1002/ana.410150302

MYERS R, 1991, J CEREBR BLOOD F MET, V11, P314, DOI 10.1038/jcbfm.1991.64

Nimmerjahn A, 2005, SCIENCE, V308, P1314, DOI 10.1126/science.1110647

Okuyama S, 1999, LIFE SCI, V64, P1455, DOI 10.1016/S0024-3205(99)00079-X

Ouchi Y, 2005, ANN NEUROL, V57, P168, DOI 10.1002/ana.20338

Pappata S, 2000, NEUROLOGY, V55, P1052, DOI 10.1212/WNL.55.7.1052

Park LCH, 2001, MECH AGEING DEV, V123, P21, DOI 10.1016/S0047-6374(01)00336-0

Pavese N, 2006, NEUROLOGY, V66, P1638, DOI 10.1212/01.wnl.0000222734.56412.17

Pedersen MD, 2006, EUR J NEUROSCI, V24, P991, DOI 10.1111/j.1460-9568.2006.04975.x

PETITTABOUE MC, 1991, EUR J PHARMACOL, V200, P347, DOI 10.1016/0014-2999(91)90594-G

Polazzi E, 2002, REV NEUROSCIENCE, V13, P221

Price CJS, 2006, STROKE, V37, P1749, DOI 10.1161/01.STR.0000226980.95389.0b

RAMSAY SC, 1992, LANCET, V339, P1054, DOI 10.1016/0140-6736(92)90576-O

Rao VLR, 2000, EXP NEUROL, V161, P102, DOI 10.1006/exnr.1999.7269

Schuitemaker A, 2004, NEUROBIOL AGING, V25, pS286, DOI 10.1016/S0197-4580(04)80947-3

Schuitemaker A, 2006, NEUROIMAGE, V31, pT159, DOI 10.1016/j.neuroimage.2006.04.142

SHAH SP, 1995, ADV CEM BASED MATER, V2, P1, DOI 10.1016/1065-7355(95)90032-2

STEPHENSON DT, 1995, J NEUROSCI, V15, P5263

Stoll G, 1999, PROG NEUROBIOL, V58, P233, DOI 10.1016/S0301-0082(98)00083-5

Streit WJ, 1996, NEUROTOXICOLOGY, V17, P671

Streit WJ, 2000, TOXICOL PATHOL, V28, P28, DOI 10.1177/019262330002800104

Thurman DJ, 1999, J HEAD TRAUMA REHAB, V14, P602, DOI 10.1097/00001199-199912000-00009

Turner MR, 2004, NEUROBIOL DIS, V15, P601, DOI 10.1016/j.nbd.2003.12.012

Venneti S, 2004, J CLIN INVEST, V113, P981, DOI 10.1172/JCI200420227

Versijpt J, 2005, MULT SCLER J, V11, P127, DOI 10.1191/1352458505ms1140oa

Vowinckel E, 1997, J NEUROSCI RES, V50, P345, DOI 10.1002/(SICI)1097-4547(19971015)50:2<345::AID-JNR22>3.0.CO;2-5

Wagner AK, 2002, NEUROSCI LETT, V334, P165, DOI 10.1016/S0304-3940(02)01103-5

Wiley CA, 2006, J NEUROVIROL, V12, P262, DOI 10.1080/13550280600873868

Zhang MR, 2003, NUCL MED BIOL, V30, P513, DOI 10.1016/S0969-8051(03)00016-7

NR 62

TC 48

Z9 56

U1 0

U2 9

PU ACADEMIC PRESS INC ELSEVIER SCIENCE

PI SAN DIEGO

PA 525 B ST, STE 1900, SAN DIEGO, CA 92101-4495 USA

SN 0014-4886

J9 EXP NEUROL

JI Exp. Neurol.

PD SEP

PY 2007

VL 207

IS 1

BP 118

EP 127

DI 10.1016/j.expneurol.2007.06.003

PG 10

WC Neurosciences

WE Science Citation Index Expanded (SCI-EXPANDED)

SC Neurosciences & Neurology

GA 210TB

UT WOS:000249477400013

PM 17658516

OA Green Accepted

DA 2024-03-03

ER

PT J

AU Kelley, BJ

Lifshitz, J

Povlishock, JT

AF Kelley, Brian Joseph

Lifshitz, Jonathan

Povlishock, John Theodore

TI Neuroinflammatory responses after experimental diffuse traumatic brain

injury

SO JOURNAL OF NEUROPATHOLOGY AND EXPERIMENTAL NEUROLOGY

LA English

DT Article

DE fluid percussion; macrophage; microglia; rat; traumatic axonal injury

ID AXONAL INJURY; INFLAMMATORY RESPONSE; MICROGLIAL ACTIVATION;

NERVOUS-SYSTEM; CELL-DYNAMICS; BARRIER; NUCLEUS; AXOTOMY; MODEL; SITES

AB Little is known about microglial activation and macrophage localization after diffuse brain injury (DBI). DBI-mediated perisomatic traumatic axonal injury (TAI) was recently identified within the neocortex, hippocampus, and thalamus, providing an opportunity to characterize immune cell responses within diffusely injured brain loci uncomplicated by contusion. By using moderate midline/central fluid percussion injury, microglial/macrophage responses were examined with antibodies targeting immune cell phenotypes and amyloid precursor protein, a marker of TAI. Parallel assessments of blood-brain barrier alterations were also performed. Within 6 to 48 hours postinjury, microglial activation within injured loci was observed, whereas microglia within non-TAI-containing regions maintained a resting phenotype. Microglial activation shared a spatiotemporal relationship with TAI though no clear interactions were observed. By 7 to 28 days postinjury, activated microglia contained myelin debris, yet revealed limited aggregation. Immunophenotypic macrophages were also localized to injured loci. Select macrophages approximated somatic membranes of perisomatically axotomized neurons with evidence of bouton disruption. No causality was established between blood-brain barrier alterations and these inflammatory responses. These findings indicate rapid, yet initially nonspecific, and persistent microglial/macrophage responses to DBI. DBI-mediated inflammatory responses suggest further expansion of traumatic brain injury histopathologic evaluations to identify neuroinflammation indicative of diffuse pathology.

C1 Virginia Commonwealth Univ, Dept Anat & Neurobiol, Sch Med, Richmond, VA 23298 USA.

C3 Virginia Commonwealth University

RP Povlishock, JT (corresponding author), Virginia Commonwealth Univ, Dept Anat & Neurobiol, Sch Med, 1101 E Marshall St,Sanger Hall Room 12-050,POB 98, Richmond, VA 23298 USA.

EM jtpovlis@vcu.edu

OI Kelley, Brian/0000-0002-6002-8828

FU NICHD NIH HHS [HD055813, F32 HD049343] Funding Source: Medline; NINDS

NIH HHS [T32NS007288, 5P30-NS047463, NS045824] Funding Source: Medline

CR Adams A, 2001, BCS CONF SERIES, P49

AIHARA N, 1995, J NEUROTRAUM, V12, P53, DOI 10.1089/neu.1995.12.53

Aldskogius H, 1998, PROG NEUROBIOL, V55, P1, DOI 10.1016/S0301-0082(97)00093-2

Aloisi F, 2001, GLIA, V36, P165, DOI 10.1002/glia.1106

Babcock AA, 2003, J NEUROSCI, V23, P7922

Bajetto A, 2002, J NEUROCHEM, V82, P1311, DOI 10.1046/j.1471-4159.2002.01091.x

Barzo P, 1996, J NEUROSURG, V85, P1113, DOI 10.3171/jns.1996.85.6.1113

Baskaya MK, 1997, NEUROSCI LETT, V226, P33

BLINZING.K, 1968, Z ZELLFORSCH MIK ANA, V85, P145, DOI 10.1007/BF00325030

Carbonell WS, 2005, J NEUROSCI, V25, P7040, DOI 10.1523/JNEUROSCI.5171-04.2005

Carbonnel WS, 1999, ACTA NEUROPATHOL, V98, P396, DOI 10.1007/s004010051100

Chen S, 2003, EXP NEUROL, V182, P87, DOI 10.1016/S0014-4886(03)00002-5

Cho BP, 2006, GLIA, V53, P92, DOI 10.1002/glia.20265

CLARK RSB, 1994, J NEUROTRAUM, V11, P499, DOI 10.1089/neu.1994.11.499

CORTEZ SC, 1989, BRAIN RES, V482, P271, DOI 10.1016/0006-8993(89)91190-6

Csuka E, 2000, NEUROREPORT, V11, P2587, DOI 10.1097/00001756-200008030-00047

Dailey ME, 1999, METHODS, V18, P222, DOI 10.1006/meth.1999.0775

Davalos D, 2005, NAT NEUROSCI, V8, P752, DOI 10.1038/nn1472

DIXON CE, 1987, J NEUROSURG, V67, P110, DOI 10.3171/jns.1987.67.1.0110

Geddes JF, 1997, NEUROPATH APPL NEURO, V23, P339, DOI 10.1046/j.1365-2990.1997.4498044.x

Gentleman SM, 2004, FORENSIC SCI INT, V146, P97, DOI 10.1016/j.forsciint.2004.06.027

GIULIAN D, 1989, J NEUROSCI, V9, P4416

Graeber MB, 1998, BRAIN RES, V813, P241, DOI 10.1016/S0006-8993(98)00859-2

Grossmann R, 2002, GLIA, V37, P229, DOI 10.1002/glia.10031

Hansson E, 2003, FASEB J, V17, P341, DOI 10.1096/fj.02-0429rev

Hartl R, 1997, ACT NEUR S, V70, P240

Hartl R, 1997, J CEREBR BLOOD F MET, V17, P1210

Hermann DM, 2000, ACTA NEUROPATHOL, V99, P147, DOI 10.1007/PL00007418

Honda S, 2001, J NEUROSCI, V21, P1975, DOI 10.1523/JNEUROSCI.21-06-01975.2001

Kelley BJ, 2006, EXP NEUROL, V198, P350, DOI 10.1016/j.expneurol.2005.12.017

Kim SU, 2005, J NEUROSCI RES, V81, P302, DOI 10.1002/jnr.20562

Kreutzberg Georg W., 1995, P355

Kreutzberg GW, 1996, TRENDS NEUROSCI, V19, P312, DOI 10.1016/0166-2236(96)10049-7

MATHEW P, 1994, ACTA NEUROCHIR, P428

MCCLAIN C, 1991, J LAB CLIN MED, V118, P225

Morganti-Kossmann Maria Cristina, 2002, Curr Opin Crit Care, V8, P101

Nimmerjahn A, 2005, SCIENCE, V308, P1314, DOI 10.1126/science.1110647

Oehmichen M, 1999, ACTA NEUROPATHOL, V97, P491, DOI 10.1007/s004010051018

OEHMICHEN M, 2006, FORENSIC NEUROPATHOL, P15

Okonkwo DO, 1998, BRAIN RES, V784, P1, DOI 10.1016/S0006-8993(97)01075-5

PERSSON L, 1976, VIRCHOWS ARCH B, V22, P21

Peters A., 1991, FINE STRUCTURE NERVO

Pettus EH, 1996, BRAIN RES, V722, P1, DOI 10.1016/0006-8993(96)00113-8

Polazzi E, 2002, REV NEUROSCIENCE, V13, P221

Popovich PG, 1997, J COMP NEUROL, V377, P443, DOI 10.1002/(SICI)1096-9861(19970120)377:3<443::AID-CNE10>3.0.CO;2-S

POVLISHOCK JT, 1985, LAB INVEST, V52, P540

Povlishock JT, 1996, ACT NEUR S, V66, P81

POVLISHOCK JT, 1992, BRAIN PATHOL, V2, P1

Raivich G, 1998, J NEUROSCI, V18, P5804

Rodriguez-Paez AC, 2005, ACTA NEUROPATHOL, V109, P603, DOI 10.1007/s00401-005-1010-z

SCHMIDT RH, 1993, J NEUROTRAUM, V10, P415, DOI 10.1089/neu.1993.10.415

SHOLAMI E, 1999, CYTOKINE GROWTH F R, V10, P119

Singleton RH, 2002, J NEUROSCI, V22, P791, DOI 10.1523/JNEUROSCI.22-03-00791.2002

Soares HD, 1995, J NEUROSCI, V15, P8223

Stence N, 2001, GLIA, V33, P256, DOI 10.1002/1098-1136(200103)33:3<256::AID-GLIA1024>3.0.CO;2-J

Stone JR, 2000, BRAIN RES, V871, P288, DOI 10.1016/S0006-8993(00)02485-9

Stone JR, 1999, ACTA NEUROPATHOL, V97, P335, DOI 10.1007/s004010050996

Streit WJ, 2000, TOXICOL PATHOL, V28, P28, DOI 10.1177/019262330002800104

SWANSON LW, 1980, NEUROENDOCRINOLOGY, V31, P410, DOI 10.1159/000123111

NR 59

TC 149

Z9 172

U1 0

U2 12

PU OXFORD UNIV PRESS INC

PI CARY

PA JOURNALS DEPT, 2001 EVANS RD, CARY, NC 27513 USA

SN 0022-3069

EI 1554-6578

J9 J NEUROPATH EXP NEUR

JI J. Neuropathol. Exp. Neurol.

PD NOV

PY 2007

VL 66

IS 11

BP 989

EP 1001

DI 10.1097/NEN.0b013e3181588245

PG 13

WC Clinical Neurology; Neurosciences; Pathology

WE Science Citation Index Expanded (SCI-EXPANDED)

SC Neurosciences & Neurology; Pathology

GA 229HE

UT WOS:000250793100003

PM 17984681

OA Bronze

DA 2024-03-03

ER

PT J

AU Nagamoto-Combs, K

Mcneal, DW

Morecraft, RJ

Combs, CK

AF Nagamoto-Combs, Kumi

Mcneal, David W.

Morecraft, Robert J.

Combs, Colin K.

TI Prolonged microgliosis in the rhesus monkey central nervous system after

traumatic brain injury

SO JOURNAL OF NEUROTRAUMA

LA English

DT Article

DE microglia; motor cortex; motor recovery; spinal cord; stroke

ID TRKB NEUROTROPHIN RECEPTORS; OUTGROWTH IN-VITRO; RAT SPINAL-CORD;

TRUNCATED TRKB; KAINIC ACID; ARM REPRESENTATION; MULTIPLE-SCLEROSIS;

NONHUMAN-PRIMATES; MOTOR CORTEX; RECOVERY

AB Impaired fine motor functions after traumatic brain injury (TBI) in humans and non-human primates often continue to improve months after injury. To initiate a series of studies in the primate model designed to investigate possible involvement of microglia/macrophage in the long-term recovery processes, changes in these cells were studied in the rhesus monkey central nervous system at 1, 6, and 12 months after a combined unilateral lesion of the arm area of the primary motor cortex and arm area of the lateral premotor cortex. Immunohistological studies showed profound CD68 immunoreactivity in the lesion area and the contralateral lateral corticospinal tract in the spinal cord at all time points, demonstrating that microglia/macrophage remain reactive at the sites of injury and axonal degeneration/survival for at least 12 months. We also observed marked increases in brain-derived neurotrophic factor (BDNF) and its receptor subtypes, TrkB[gp145] and TrkB[TK-], around the cortical lesion site after 6-month survival. Similar increases were also observed in the spinal cord, although it was less apparent for TrkB[gp145]. Double-labeling revealed that a sub-population of CD68-immunoreacitve microglia/macrophage co-expressed BDNF in the cortex and spinal cord, and also TrkB[gp145] or TrkB[TK-] in the spinal cord. In contrast, cytokine expression of tumor necrosis factor-alpha (TNF-alpha), interleukin-1 beta (IL-1 beta), and interleukin-6 (IL-6) at these time intervals was less prominent, suggesting that immediate inflammatory responses had subsided. These results demonstrate that microglia/macrophage undergo prolonged activation after TBI in the non-human primate brain and express BDNF and its receptors, suggesting their tropic/trophic roles in the long-term recovery processes.

C1 Univ N Dakota, Sch Med & Hlth Sci, Dept Pharmacol Physiol & Therapeut, Grand Forks, ND 58202 USA.

Univ S Dakota, Sch Med, Div Basic Biomed Sci, Vermillion, SD USA.

C3 University of North Dakota Grand Forks; University of South Dakota

RP Nagamoto-Combs, K (corresponding author), Univ N Dakota, Sch Med & Hlth Sci, Dept Pharmacol Physiol & Therapeut, 504 Hamline St, Neurosci Bldg, Grand Forks, ND 58202 USA.

EM ccombs@medicine.nodak.edu

RI Nagamoto-Combs, Kumi/AAN-6697-2021

CR Allan SM, 2001, NAT REV NEUROSCI, V2, P734, DOI 10.1038/35094583

Asami T, 2006, BIOCHEM BIOPH RES CO, V344, P941, DOI 10.1016/j.bbrc.2006.03.228

Batchelor PE, 2002, MOL CELL NEUROSCI, V21, P436, DOI 10.1006/mcne.2002.1185

Batchelor PE, 2002, EUR J NEUROSCI, V15, P826, DOI 10.1046/j.1460-9568.2002.01914.x

Beck H, 2003, J CEREBR BLOOD F MET, V23, P709, DOI 10.1097/01.WCB.0000065940.18332.8D

BECK KD, 1993, J NEUROSCI, V13, P4001

Bütefisch CM, 2006, NEUROL SCI, V27, pS18, DOI 10.1007/s10072-006-0540-z

Cagnin A, 2001, BRAIN, V124, P2014, DOI 10.1093/brain/124.10.2014

Carmichael ST, 2003, NEUROSCIENTIST, V9, P64, DOI 10.1177/1073858402239592

Coull JAM, 2005, NATURE, V438, P1017, DOI 10.1038/nature04223

Dancause N, 2005, J NEUROSCI, V25, P10167, DOI 10.1523/JNEUROSCI.3256-05.2005

Darling WG, 2006, J NEUROSCI METH, V154, P38, DOI 10.1016/j.jneumeth.2005.11.013

Eide FF, 1996, J NEUROSCI, V16, P3123

Elkabes S, 1996, J NEUROSCI, V16, P2508

Fleming JC, 2006, BRAIN, V129, P3249, DOI 10.1093/brain/awl296

Frost SB, 2003, J NEUROPHYSIOL, V89, P3205, DOI 10.1152/jn.01143.2002

Fryer RH, 1997, EXP NEUROL, V148, P616, DOI 10.1006/exnr.1997.6699

Gerhard A, 2005, NEUROIMAGE, V24, P591, DOI 10.1016/j.neuroimage.2004.09.034

Goutan E, 1998, MOL BRAIN RES, V59, P154, DOI 10.1016/S0169-328X(98)00156-9

Haapasalo A, 2001, BIOCHEM BIOPH RES CO, V280, P1352, DOI 10.1006/bbrc.2001.4296

JORGENSEN MB, 1993, EXP NEUROL, V120, P70, DOI 10.1006/exnr.1993.1041

Kasahara K, 2006, SPINE, V31, P2059, DOI 10.1097/01.brs.0000231893.21964.f2

Katz DI, 2004, ARCH PHYS MED REHAB, V85, P865, DOI 10.1016/j.apmr.2003.11.020

KATZ DI, 1994, ARCH NEUROL-CHICAGO, V51, P661, DOI 10.1001/archneur.1994.00540190041013

Kerschensteiner M, 1999, J EXP MED, V189, P865, DOI 10.1084/jem.189.5.865

King VR, 2000, EXP NEUROL, V165, P327, DOI 10.1006/exnr.2000.7480

KLEIN R, 1990, CELL, V61, P647, DOI 10.1016/0092-8674(90)90476-U

Kopp B, 1999, NEUROREPORT, V10, P807, DOI 10.1097/00001756-199903170-00026

Kuhtz-Buschbeck JP, 2003, DEV MED CHILD NEUROL, V45, P821, DOI 10.1017/S001216220300152X

Ladeby R, 2005, BRAIN RES REV, V48, P196, DOI 10.1016/j.brainresrev.2004.12.009

Livingston DH, 2005, J TRAUMA, V59, P1298, DOI 10.1097/01.ta.0000196002.03681.18

Mack CL, 2003, J NEUROIMMUNOL, V144, P68, DOI 10.1016/j.jneuroim.2003.08.032

Malcangio M, 2003, TRENDS PHARMACOL SCI, V24, P116, DOI 10.1016/S0165-6147(03)00025-7

Morecraft RJ, 2004, J COMP NEUROL, V469, P37, DOI 10.1002/cne.10980

Morecraft RJ, 2002, BRAIN, V125, P176, DOI 10.1093/brain/awf011

MORECRAFT RJ, 1992, J COMP NEUROL, V323, P341, DOI 10.1002/cne.903230304

Morecraft RJ, 2007, J COMP NEUROL, V504, P149, DOI 10.1002/cne.21438

Morecraft RJ, 2007, J COMP NEUROL, V500, P134, DOI 10.1002/cne.21165

Nakamura M, 2001, EXP NEUROL, V169, P407, DOI 10.1006/exnr.2001.7670

Niemeier JP, 2007, NEUROPSYCHOL REHABIL, V17, P293, DOI 10.1080/09602010600814729

Novikova LN, 2000, EUR J NEUROSCI, V12, P776, DOI 10.1046/j.1460-9568.2000.00978.x

Nudo Randolph J, 2006, NeuroRx, V3, P420

NUDO RJ, 1992, J NEUROSCI, V12, P2918, DOI 10.1523/JNEUROSCI.12-08-02918.1992

Nudo RJ, 1999, CURR OPIN NEUROBIOL, V9, P740, DOI 10.1016/S0959-4388(99)00027-6

Panagis L, 2005, EUR J NEUROSCI, V21, P2305, DOI 10.1111/j.1460-9568.2005.04046.x

Pizzimenti MA, 2007, J NEUROPHYSIOL, V98, P1015, DOI 10.1152/jn.00354.2007

Rose CR, 2003, NATURE, V426, P74, DOI 10.1038/nature01983

Salie R, 2005, INT J DEV NEUROSCI, V23, P587, DOI 10.1016/j.ijdevneu.2005.07.003

SBORDONE RJ, 1995, BRAIN INJURY, V9, P285, DOI 10.3109/02699059509008199

Shelton FDAP, 2001, STROKE, V32, P107, DOI 10.1161/01.STR.32.1.107

Smith ME, 2001, MICROSC RES TECHNIQ, V54, P81, DOI 10.1002/jemt.1123

Streit WJ, 2000, TOXICOL PATHOL, V28, P28, DOI 10.1177/019262330002800104

Streit WJ, 1999, PROG NEUROBIOL, V57, P563, DOI 10.1016/S0301-0082(98)00069-0

Sun W, 2003, MOL CELL NEUROSCI, V24, P875, DOI 10.1016/S1044-7431(03)00219-7

Suzuki H, 2001, NEUROSCI LETT, V312, P95, DOI 10.1016/S0304-3940(01)02198-X

Tanaka R, 2003, NEUROSCIENCE, V117, P531, DOI 10.1016/S0306-4522(02)00954-5

Taub Edward, 2003, Phys Med Rehabil Clin N Am, V14, pS77, DOI 10.1016/S1047-9651(02)00052-9

WADE DT, 1985, J NEUROL NEUROSUR PS, V48, P7, DOI 10.1136/jnnp.48.1.7

Weidner N, 2001, P NATL ACAD SCI USA, V98, P3513, DOI 10.1073/pnas.051626798

Wenzelburger R, 2005, BRAIN, V128, P64, DOI 10.1093/brain/awh317

Widenfalk J, 2001, J NEUROSCI, V21, P3457, DOI 10.1523/JNEUROSCI.21-10-03457.2001

Wilson S, 2004, J NEUROTRAUM, V21, P239, DOI 10.1089/089771504322972031

Wong JYF, 1997, EXP NEUROL, V148, P83, DOI 10.1006/exnr.1997.6670

Zhang JM, 2003, EXP NEUROL, V183, P469, DOI 10.1016/S0014-4886(03)00222-X

NR 64

TC 110

Z9 131

U1 0

U2 9

PU MARY ANN LIEBERT, INC

PI NEW ROCHELLE

PA 140 HUGUENOT STREET, 3RD FL, NEW ROCHELLE, NY 10801 USA

SN 0897-7151

EI 1557-9042

J9 J NEUROTRAUM

JI J. Neurotrauma

PD NOV

PY 2007

VL 24

IS 11

BP 1719

EP 1742

DI 10.1089/neu.2007.0377

PG 24

WC Critical Care Medicine; Clinical Neurology; Neurosciences

WE Science Citation Index Expanded (SCI-EXPANDED)

SC General & Internal Medicine; Neurosciences & Neurology

GA 234MZ

UT WOS:000251169500005

PM 18001202

DA 2024-03-03

ER

PT J

AU Zhang, ZY

Zhang, ZR

Fauser, U

Schluesener, HJ

AF Zhang, Zhi-Yuan

Zhang, Zhiren

Fauser, Uwe

Schluesener, Hermann J.

TI Global hypomethylation defines a sub-population of reactive

microglia/macrophages in experimental traumatic brain injury

SO NEUROSCIENCE LETTERS

LA English

DT Article

DE traumatic brain injury; hypomethylation; 5-methylcytosine;

microglia/macrophages; dexamethasone

ID DNA METHYLATION; HEAD-INJURY; SPINAL-CORD; MICROGLIA; RATS; ACTIVATION;

EXPRESSION; CHROMATIN; IMPACT; CELLS

AB Global alterations in gene expression have been observed indifferent traumatic brain injury (TBI) models and are considered of crucial importance to the development of subsequent tissue injury and repair. Cytosine methylation is a well-known process of endogenous DNA modification in mammals and the primary mechanism responsible for changes in epigenetic gene expression. Here we have investigated the early global spatio-temporal changes of the status of cellular DNA methylation in a rat TBI model by immunohistochemistry and analyzed the effects of dexamethasone on these changes. Global cellular hypomethylation was seen as early as day 1 in pannecrosis and day 2 in peripannecrosis following TBI. A subpopulation of reactive microglia/macrophages was identified as the major source of hypomethylated cells by double-staining experiments. Further, peripheral administration of dexamethasone suppressed this lesional hypomethylation at day 2 post-injury. In sum, our data suggest that lesional hypomethylation defines a sub-population of activated microglia/macrophages involved in the early processes following traumatic brain injury. (c) 2007 Elsevier Ireland Ltd. All rights reserved.

C1 [Zhang, Zhi-Yuan; Zhang, Zhiren; Fauser, Uwe; Schluesener, Hermann J.] Univ Tubingen, Inst Brain Res, D-72076 Tubingen, Germany.

C3 Eberhard Karls University of Tubingen

RP Zhang, ZR (corresponding author), Univ Tubingen, Inst Brain Res, Calwer St 3, D-72076 Tubingen, Germany.

EM zhangzhiren@yahoo.com

RI zhang, zhiyuan/GRS-2141-2022; Zhang, Zhiren/O-1012-2019

OI Zhang, Zhiren/0000-0002-5238-2835; Zhang, Zhi-Yuan/0000-0001-9067-0670

CR AIHARA N, 1995, J NEUROTRAUM, V12, P53, DOI 10.1089/neu.1995.12.53

Attwood JT, 2002, CELL MOL LIFE SCI, V59, P241, DOI 10.1007/s00018-002-8420-z

Carmichael ST, 2003, CURR OPIN NEUROL, V16, P699, DOI 10.1097/00019052-200312000-00009

CLARK RSB, 1994, J NEUROTRAUM, V11, P499, DOI 10.1089/neu.1994.11.499

Costello JF, 2001, J MED GENET, V38, P285, DOI 10.1136/jmg.38.5.285

Dash PK, 2004, NEUROCHEM RES, V29, P1275, DOI 10.1023/B:NERE.0000023614.30084.eb

De Capoa A, 1999, FASEB J, V13, P89

Drew PD, 2000, BRAIN RES BULL, V52, P391, DOI 10.1016/S0361-9230(00)00275-6

Endres M, 2001, NEUROREPORT, V12, P3763, DOI 10.1097/00001756-200112040-00032

Endres M, 2000, J NEUROSCI, V20, P3175, DOI 10.1523/JNEUROSCI.20-09-03175.2000

Garcia-Segura LM, 2006, GLIA, V54, P485, DOI 10.1002/glia.20404

Gloria L, 1996, CANCER, V78, P2300, DOI 10.1002/(SICI)1097-0142(19961201)78:11<2300::AID-CNCR5>3.0.CO;2-Q

Gottesfeld Z, 2002, J NEUROTRAUM, V19, P317, DOI 10.1089/089771502753594882

Griesbach GS, 2002, J NEUROTRAUM, V19, P803, DOI 10.1089/08977150260190401

Holmin, 1996, ACTA NEUROCHIR, V138, P418, DOI 10.1007/BF01420304

Kempermann G, 2003, SCIENCE, V302, P1689, DOI 10.1126/science.1092864

Kreutzberg GW, 1996, TRENDS NEUROSCI, V19, P312, DOI 10.1016/0166-2236(96)10049-7

Ladeby R, 2005, BRAIN RES REV, V48, P196, DOI 10.1016/j.brainresrev.2004.12.009

Muzha I, 2004, LANCET, V364, P1321, DOI 10.1016/S0140-6736(04)17188-2

Orihara Y, 2001, FORENSIC SCI INT, V123, P142, DOI 10.1016/S0379-0738(01)00537-0

Rabchevsky AG, 1997, J NEUROSCI RES, V47, P34

Seifert HH, 2007, EXP MOL PATHOL, V82, P292, DOI 10.1016/j.yexmp.2006.08.002

Sharma RP, 2005, J PSYCHIATR NEUROSCI, V30, P257

Smith SS, 2000, J MOL BIOL, V302, P1, DOI 10.1006/jmbi.2000.4046

Stenvinkel P, 2007, J INTERN MED, V261, P488, DOI 10.1111/j.1365-2796.2007.01777.x

Streit WJ, 2002, GLIA, V40, P133, DOI 10.1002/glia.10154

Woods AG, 1999, NEUROSCIENCE, V91, P1277, DOI 10.1016/S0306-4522(98)00685-X

Yatsiv I, 2002, J CEREBR BLOOD F MET, V22, P971, DOI 10.1097/00004647-200208000-00008

Zhang ZY, 2007, ACTA NEUROPATHOL, V113, P675, DOI 10.1007/s00401-007-0195-8

NR 29

TC 50

Z9 54

U1 0

U2 7

PU ELSEVIER IRELAND LTD

PI CLARE

PA ELSEVIER HOUSE, BROOKVALE PLAZA, EAST PARK SHANNON, CO, CLARE, 00000,

IRELAND

SN 0304-3940

EI 1872-7972

J9 NEUROSCI LETT

JI Neurosci. Lett.

PD DEC 11

PY 2007

VL 429

IS 1

BP 1

EP 6

DI 10.1016/j.neulet.2007.09.061

PG 6

WC Neurosciences

WE Science Citation Index Expanded (SCI-EXPANDED)

SC Neurosciences & Neurology

GA 245UP

UT WOS:000251964600001

PM 17996371

DA 2024-03-03

ER

PT J

AU Zhang, ZR

Fauser, U

Schluesener, HJ

AF Zhang, Zhiren

Fauser, Uwe

Schluesener, Hermann J.

TI Dexamethasone suppresses infiltration of RhoA<SUP>+</SUP> cells into

early lesions of rat traumatic brain injury

SO ACTA NEUROPATHOLOGICA

LA English

DT Article

DE RhoA; dexamethasone; traumatic brain injury; microglia; macrophages;

weight-drop model

ID CENTRAL-NERVOUS-SYSTEM; SEVERE HEAD-INJURY; EXPERIMENTAL AUTOIMMUNE

ENCEPHALOMYELITIS; SPINAL-CORD-INJURY; RHO-GTPASES; LEUKOCYTE

RECRUITMENT; CLOSTRIDIUM-BOTULINUM; ACTIN CYTOSKELETON; AXON

REGENERATION; ADP-RIBOSYLATION

AB Inflammatory cell infiltration is a major part of secondary tissue damage in traumatic brain injury (TBI). RhoA is an important member of Rho GTPases and is involved in leukocyte migration. Inhibition of RhoA and its downstream target, Rho-associated coiled kinase (ROCK), has been proven to promote axon regeneration and function recovery following injury in the central nervous system (CNS). Previously, we showed that dexamethasone, an immunosuppressive corticosteroid, attenuated early expression of three molecules associated with microglia/macrophages activation following TBI in rats. Here, the effects of dexamethasone on the early expression of RhoA have been investigated in brains of TBI rats by immunohistochemistry. In brains of rats treated with TBI alone, significant RhoA(+) cell accumulation was observed at 18 h post-injury and continuously increased during our observed time period. The accumulated RhoA(+) cells were distributed to the areas of pannecrosis and selective neuronal loss. Most accumulated RhoA(+) cells were identified as active microglia/macrophages by double-labelling. Dexamethasone (1 mg/kg body weight) was intraperitoneally injected on day 0 and 2 immediately following brain injury. Numbers of RhoA(+) cells were significantly reduced on day 1 and 2 following administration of dexamethasone but returned to vehicle control level on day 4. However, dexamethasone treatment did not change the proportion of RhoA(+) cells. These observations suggest that dexamethasone has only a transient effect on early leukocyte recruitment.

C1 [Zhang, Zhiren; Fauser, Uwe; Schluesener, Hermann J.] Univ Tubingen, Brain Res Inst, D-72076 Tubingen, Germany.

C3 Eberhard Karls University of Tubingen

RP Zhang, ZR (corresponding author), Univ Tubingen, Brain Res Inst, Calwer Str 3, D-72076 Tubingen, Germany.

EM zhangzhiren@yahoo.com

RI Zhang, Zhiren/O-1012-2019

OI Zhang, Zhiren/0000-0002-5238-2835

CR Alblas J, 2001, MOL BIOL CELL, V12, P2137, DOI 10.1091/mbc.12.7.2137

Angkachatchai V, 1999, J IMMUNOL, V163, P3819

BARKS JDE, 1991, PEDIATR RES, V29, P558, DOI 10.1203/00006450-199106010-00008

Brabeck C, 2004, J NEUROTRAUM, V21, P697, DOI 10.1089/0897715041269597

CHARDIN P, 1989, EMBO J, V8, P1087, DOI 10.1002/j.1460-2075.1989.tb03477.x

Conrad S, 2005, J COMP NEUROL, V487, P166, DOI 10.1002/cne.20561

COOPER PR, 1979, J NEUROSURG, V51, P307, DOI 10.3171/jns.1979.51.3.0307

DEARDEN NM, 1986, J NEUROSURG, V64, P81, DOI 10.3171/jns.1986.64.1.0081

Dergham P, 2002, J NEUROSCI, V22, P6570

Donovan FM, 1997, J NEUROSCI, V17, P5316

Dubreuil CI, 2003, J CELL BIOL, V162, P233, DOI 10.1083/jcb.200301080

Etienne-Manneville S, 2002, NATURE, V420, P629, DOI 10.1038/nature01148

Eto M, 2002, CIRCULATION, V105, P1756, DOI 10.1161/01.CIR.0000015465.73933.3B

Felszeghy K, 2004, NEUROIMMUNOMODULAT, V11, P404, DOI 10.1159/000080151

Fournier AE, 2003, J NEUROSCI, V23, P1416, DOI 10.1523/JNEUROSCI.23-04-01416.2003

Gaab M R, 1994, Zentralbl Neurochir, V55, P135

Gahm C, 2005, ACTA NEUROCHIR, V147, P1071, DOI 10.1007/s00701-005-0590-7

Gottesfeld Z, 2002, J NEUROTRAUM, V19, P317, DOI 10.1089/089771502753594882

Graham DI, 2000, J NEUROPATH EXP NEUR, V59, P641, DOI 10.1093/jnen/59.8.641

Greenwood J, 2003, FASEB J, V17, P905, DOI 10.1096/fj.02-1014fje

Hall A, 1998, SCIENCE, V279, P509, DOI 10.1126/science.279.5350.509

HALL ED, 1987, J PHARMACOL EXP THER, V242, P137

Hendricks JJA, 2004, J EXP MED, V200, P1667, DOI 10.1084/jem.20040819

Holmin, 1996, ACTA NEUROCHIR, V138, P418, DOI 10.1007/BF01420304

Irazuzta J, 2005, INTENS CARE MED, V31, P146, DOI 10.1007/s00134-004-2462-7

Kim JS, 2005, EXP MOL MED, V37, P575, DOI 10.1038/emm.2005.71

Knaus UG, 2000, IMMUNOL RES, V21, P103, DOI 10.1385/IR:21:2-3:103

Kubes P, 2000, BRAIN PATHOL, V10, P127

LANG P, 1992, J BIOL CHEM, V267, P11677

Luo LQ, 2000, NAT REV NEUROSCI, V1, P173, DOI 10.1038/35044547

Maderna P, 2002, AM J PATHOL, V160, P2275, DOI 10.1016/S0002-9440(10)61175-3

Marshall LF, 2000, NEUROSURGERY, V47, P546, DOI 10.1097/00006123-200009000-00002

Mueller BK, 2005, NAT REV DRUG DISCOV, V4, P387, DOI 10.1038/nrd1719

Mueller CA, 2003, J NEUROIMMUNOL, V135, P1, DOI 10.1016/S0165-5728(02)00427-7

Muzha I, 2004, LANCET, V364, P1321, DOI 10.1016/S0140-6736(04)17188-2

Negishi M, 2002, J BIOCHEM, V132, P157, DOI 10.1093/oxfordjournals.jbchem.a003205

O'Connor KL, 2000, J CELL BIOL, V148, P253, DOI 10.1083/jcb.148.2.253

O'Kane EM, 2003, MOL BRAIN RES, V114, P1, DOI 10.1016/S0169-328X(03)00121-9

Pertz O, 2006, NATURE, V440, P1069, DOI 10.1038/nature04665

Pixley FJ, 2005, J CELL SCI, V118, P1873, DOI 10.1242/jcs.02314

Ridley AJ, 2001, J CELL SCI, V114, P2713

Ridley AJ, 2001, TRENDS CELL BIOL, V11, P471, DOI 10.1016/S0962-8924(01)02153-5

Ridley AJ, 2001, FEBS LETT, V498, P168, DOI 10.1016/S0014-5793(01)02481-4

RUBIN EJ, 1988, MOL CELL BIOL, V8, P418, DOI 10.1128/MCB.8.1.418

Sandvig A, 2004, GLIA, V46, P225, DOI 10.1002/glia.10315

Sauerland S, 2004, LANCET, V364, P1291, DOI 10.1016/S0140-6736(04)17202-4

Schluesener HJ, 1997, GLIA, V20, P365, DOI 10.1002/(SICI)1098-1136(199708)20:4<365::AID-GLIA8>3.0.CO;2-4

Schwab ME, 2004, CURR OPIN NEUROBIOL, V14, P118, DOI 10.1016/j.conb.2004.01.004

Skaper SD, 2001, PROG NEUROBIOL, V65, P593, DOI 10.1016/S0301-0082(01)00017-X

Stramer B, 2005, J CELL BIOL, V168, P567, DOI 10.1083/jcb.200405120

Tapon N, 1997, CURR OPIN CELL BIOL, V9, P86, DOI 10.1016/S0955-0674(97)80156-1

Walters CE, 2002, J IMMUNOL, V168, P4087, DOI 10.4049/jimmunol.168.8.4087

Wheeler AP, 2004, EXP CELL RES, V301, P43, DOI 10.1016/j.yexcr.2004.08.012

Yang JT, 2005, EXP NEUROL, V192, P437, DOI 10.1016/j.expneurol.2004.12.023

Zhang ZY, 2007, ACTA NEUROPATHOL, V113, P675, DOI 10.1007/s00401-007-0195-8

Zhang ZR, 2006, EXP NEUROL, V197, P252, DOI 10.1016/j.expneurol.2005.09.015

NR 56

TC 13

Z9 15

U1 0

U2 6

PU SPRINGER

PI NEW YORK

PA 233 SPRING ST, NEW YORK, NY 10013 USA

SN 0001-6322

EI 1432-0533

J9 ACTA NEUROPATHOL

JI Acta Neuropathol.

PD MAR

PY 2008

VL 115

IS 3

BP 335

EP 343

DI 10.1007/s00401-007-0301-y

PG 9

WC Clinical Neurology; Neurosciences; Pathology

WE Science Citation Index Expanded (SCI-EXPANDED)

SC Neurosciences & Neurology; Pathology

GA 265GB

UT WOS:000253346000007

PM 17929039

DA 2024-03-03

ER

PT J

AU Hailer, NP

AF Hailer, Nils P.

TI Immunosuppression after traumatic or ischemic CNS damage: It is

neuroprotective and illuminates the role of microglial cells

SO PROGRESS IN NEUROBIOLOGY

LA English

DT Review

DE spinal cord injury; cerebral ischemia; traumatic brain injury;

microglial cell; microglia; astrocyte; neuron; immunology;

immunosuppression; immunosuppressive drugs; neuroprotection;

cytosine-arabinoside; steroids; methylprednisolone; dexamethasone;

cyclosporin A; CsA; tacrolimus; FK506; rapamycin; sirolimus;

mycophenolate mofetil; mycophenolic acid; minocycline; clodronate; IL-1

receptor antagonist (IL-1ra)

ID SPINAL-CORD-INJURY; HIPPOCAMPAL SLICE CULTURES; FOCAL CEREBRAL-ISCHEMIA;

CENTRAL-NERVOUS-SYSTEM; INTERLEUKIN-1 RECEPTOR ANTAGONIST; ADHESION

MOLECULE EXPRESSION; COLONY-STIMULATING FACTOR; TUMOR-NECROSIS-FACTOR;

PERIPHERAL BENZODIAZEPINE RECEPTOR; IMPROVES FUNCTIONAL RECOVERY

AB Acute traumatic and ischemic events in the central nervous system (CNS) invariably result in activation of microglial cells as local representatives of the immune system. It is still under debate whether activated microglia promote neuronal survival, or whether they exacerbate the original extent of neuronal damage. Protagonists of the view that microglial cells cause secondary damage have proposed that inhibition of microglial activation by immunosuppression is beneficial after acute CNS damage. It is the aim of this review to analyse the effects of immunosuppressants on isolated microglial cells and neurons, and to scrutinize the effects of immunosuppression in different in vivo models of acute CNS trauma or ischemia. It is found that the immunosuppressants cytosine-arabinoside, different steroids, cyclosporin A, FK506, rapamycin, mycophenolate mofetil, and minocycline all have direct inhibitory effects on microglial cells. These effects are mainly exerted by inhibiting microglial proliferation or microglial secretion of neurotoxic substances such as proinflammatory cytokines and nitric oxide. Furthermore, immunosuppression after acute CNS trauma or ischemia results in improved structure preservation and, mostly, in enhanced function. However, all investigated immunosuppressants also have direct effects on neurons, and some immunosuppressants affect other glial cells such as astrocytes. In summary, it is safe to conclude that immunosuppression after acute CNS trauma or ischemia is neuroprotective. Furthermore, circumferential evidence indicates that microglial activation after traumatic or ischemic CNS damage is not beneficial to adjacent neurons in the immediate aftermath of such acute lesions. Further experiments with more specific agents or genetic approaches that specifically inhibit microglial cells are needed in order to fully answer the question of whether microglial activation is "good or bad". (c) 2007 Elsevier Ltd. All rights reserved.

C1 Univ Uppsala Hosp, Inst Surg Sci, Dept Orthopaed, SE-75185 Uppsala, Sweden.

C3 Uppsala University; Uppsala University Hospital

RP Hailer, NP (corresponding author), Univ Uppsala Hosp, Inst Surg Sci, Dept Orthopaed, SE-75185 Uppsala, Sweden.

EM nils.hailer@surgsci.uu.se

OI Hailer, Nils/0000-0002-3233-2638

CR ABROMSONLEEMAN S, 1993, J NEUROIMMUNOL, V45, P89, DOI 10.1016/0165-5728(93)90168-X

AKIYAMA H, 1990, J NEUROIMMUNOL, V30, P81, DOI 10.1016/0165-5728(90)90055-R

Alessandri B, 2002, J NEUROTRAUM, V19, P829, DOI 10.1089/08977150260190429

Allan SM, 2005, NAT REV IMMUNOL, V5, P629, DOI 10.1038/nri1664

Allison AC, 2005, TRANSPLANTATION, V80, pS181, DOI 10.1097/01.tp.0000186390.10150.66

Allison AC, 2000, IMMUNOPHARMACOLOGY, V47, P85, DOI 10.1016/S0162-3109(00)00188-0

Ambrosini E, 2004, NEUROCHEM RES, V29, P1017, DOI 10.1023/B:NERE.0000021246.96864.89

Arii T, 2001, NEUROL RES, V23, P755, DOI 10.1179/016164101101199135

Babcock AA, 2003, J NEUROSCI, V23, P7922

Bavetta S, 1999, EXP NEUROL, V158, P382, DOI 10.1006/exnr.1999.7119

Bechmann I, 2001, EUR J NEUROSCI, V14, P1651, DOI 10.1046/j.0953-816x.2001.01793.x

BEHRMANN DL, 1994, EXP NEUROL, V126, P61, DOI 10.1006/exnr.1994.1042

Bennett PC, 2002, BRAIN RES, V927, P180, DOI 10.1016/S0006-8993(01)03353-4

BENVENISTE EN, 1992, AM J PHYSIOL, V263, pC1, DOI 10.1152/ajpcell.1992.263.1.C1

Bessis A, 2007, GLIA, V55, P233, DOI 10.1002/glia.20459

Bianco F, 2006, J NEUROCHEM, V99, P745, DOI 10.1111/j.1471-4159.2006.04101.x

BLACK RA, 1988, J BIOL CHEM, V263, P9437

BOJE KM, 1992, BRAIN RES, V587, P250, DOI 10.1016/0006-8993(92)91004-X

Bracken MB, 1998, J NEUROSURG, V89, P699, DOI 10.3171/jns.1998.89.5.0699

BRAUGHLER JM, 1984, J NEUROSURG, V61, P290, DOI 10.3171/jns.1984.61.2.0290

BROSH S, 1992, J NEUROCHEM, V58, P1485, DOI 10.1111/j.1471-4159.1992.tb11368.x

Büki A, 1999, J NEUROTRAUM, V16, P511, DOI 10.1089/neu.1999.16.511

BURCH PA, 1988, J NATL CANCER I, V80, P1211, DOI 10.1093/jnci/80.15.1211

Butcher SP, 1997, J NEUROSCI, V17, P6939

Caggiano AO, 1998, J NEUROCHEM, V70, P2357

Capano M, 2002, BIOCHEM J, V363, P29, DOI 10.1042/bj3630029

Carlson SL, 1998, EXP NEUROL, V151, P77, DOI 10.1006/exnr.1998.6785

CARR SF, 1993, J BIOL CHEM, V268, P27286

Carreau A, 1997, NEUROPHARMACOLOGY, V36, P1755, DOI 10.1016/S0028-3908(97)00160-3

Cayli SR, 2004, EUR SPINE J, V13, P724, DOI 10.1007/s00586-003-0550-y

Çetin A, 2006, EUR SPINE J, V15, P1539, DOI 10.1007/s00586-006-0091-2

Chang JY, 2000, NEUROCHEM RES, V25, P903, DOI 10.1023/A:1007511221666

CHAO CC, 1992, J INFECT DIS, V166, P847, DOI 10.1093/infdis/166.4.847

Cheneval D, 1998, J BIOL CHEM, V273, P17846, DOI 10.1074/jbc.273.28.17846

Choi HB, 2002, J NEUROCHEM, V83, P546, DOI 10.1046/j.1471-4159.2002.01122.x

CLIPSTONE NA, 1992, NATURE, V357, P695, DOI 10.1038/357695a0

CONSTANTINI S, 1994, J NEUROSURG, V80, P97, DOI 10.3171/jns.1994.80.1.0097

Costantini LC, 2000, EXP NEUROL, V164, P60, DOI 10.1006/exnr.2000.7417

Cottrell BL, 2006, PLAST RECONSTR SURG, V118, P615, DOI 10.1097/01.prs.0000233029.57397.4a

DAWSON TM, 1993, P NATL ACAD SCI USA, V90, P9808, DOI 10.1073/pnas.90.21.9808

Dehghani F, 2004, EXP NEUROL, V189, P241, DOI 10.1016/j.expneurol.2004.06.010

Dehghani F, 2003, EUR J NEUROSCI, V18, P1061, DOI 10.1046/j.1460-9568.2003.02821.x

DEHGHANI F, UNPUB MICROGLIAL CEL

del Zoppo GJ, 2007, STROKE, V38, P646, DOI 10.1161/01.STR.0000254477.34231.cb

Diaz-Ruiz A, 1999, NEUROSCI LETT, V266, P61, DOI 10.1016/S0304-3940(99)00255-4

Drew PD, 2000, BRAIN RES BULL, V52, P391, DOI 10.1016/S0361-9230(00)00275-6

Eder C, 2005, J NEUROSCI RES, V81, P314, DOI 10.1002/jnr.20476

Eder C, 1998, J NEUROSCI, V18, P7127

EDER C, 1995, PFLUG ARCH EUR J PHY, V430, P526, DOI 10.1007/BF00373889

Eder C, 1999, EUR J NEUROSCI, V11, P4251, DOI 10.1046/j.1460-9568.1999.00852.x

Edwards P, 2005, LANCET, V365, P1957

Elkabes S, 1996, J NEUROSCI, V16, P2508

Erlich S, 2007, NEUROBIOL DIS, V26, P86, DOI 10.1016/j.nbd.2006.12.003

FAGAN AM, 1990, EXP NEUROL, V110, P105, DOI 10.1016/0014-4886(90)90055-W

Fedoroff S, 1991, Adv Exp Med Biol, V296, P135

Ferrari D, 1999, J BIOL CHEM, V274, P13205, DOI 10.1074/jbc.274.19.13205

Festoff BW, 2006, J NEUROCHEM, V97, P1314, DOI 10.1111/j.1471-4159.2006.03799.x

FIELD EJ, 1955, J ANAT, V89, P201

FISCHER HG, 1995, NEUROSCIENCE, V64, P183, DOI 10.1016/0306-4522(94)00398-O

Flügel A, 2001, J CEREBR BLOOD F MET, V21, P69

Frith JC, 2001, ARTHRITIS RHEUM, V44, P2201, DOI 10.1002/1529-0131(200109)44:9<2201::AID-ART374>3.0.CO;2-E

Fujita T, 1998, ACTA NEUROCHIR, V140, P275, DOI 10.1007/s007010050095

Furuichi Y, 2003, J CEREBR BLOOD F MET, V23, P1183, DOI 10.1097/01.WCB.0000088761.02615.EB

Furuichi Y, 2007, EXP NEUROL, V204, P138, DOI 10.1016/j.expneurol.2006.10.003

Gabryel B, 2004, POL J PHARMACOL, V56, P129

GANTER S, 1992, J NEUROSCI RES, V33, P218, DOI 10.1002/jnr.490330205

GEHRMANN J, 1993, BRAIN PATHOL, V3, P11, DOI 10.1111/j.1750-3639.1993.tb00720.x

GIORDANA MT, 1994, NEUROPATH APPL NEURO, V20, P163, DOI 10.1111/j.1365-2990.1994.tb01175.x

GIULIAN D, 1989, J NEUROSCI, V9, P4416

GIULIAN D, 1986, J NEUROSCI, V6, P2163

GIULIAN D, 1994, NEUROCHEM INT, V25, P227, DOI 10.1016/0197-0186(94)90066-3

Glomsda BA, 2003, SPINAL CORD, V41, P610, DOI 10.1038/sj.sc.3101512

GOLD BG, 1995, J NEUROSCI, V15, P7509

Gorio A, 2005, P NATL ACAD SCI USA, V102, P16379, DOI 10.1073/pnas.0508479102

GRASSI F, 1994, BRAIN RES, V659, P226, DOI 10.1016/0006-8993(94)90883-4

GRIFFIN WST, 1989, P NATL ACAD SCI USA, V86, P7611

GUTH L, 1985, EXP NEUROL, V88, P44, DOI 10.1016/0014-4886(85)90112-8

Haghighi SS, 2000, SPINAL CORD, V38, P733, DOI 10.1038/sj.sc.3101074

Hailer NP, 1997, EUR J NEUROSCI, V9, P863, DOI 10.1111/j.1460-9568.1997.tb01436.x

Hailer NP, 1999, EUR J NEUROSCI, V11, P3359, DOI 10.1046/j.1460-9568.1999.00808.x

Hailer NP, 2005, EUR J NEUROSCI, V21, P2347, DOI 10.1111/j.1460-9568.2005.04067.x

Hailer NP, 1998, BRAIN PATHOL, V8, P459

Hailer NP, 2001, EUR J NEUROSCI, V14, P315, DOI 10.1046/j.0953-816x.2001.01649.x

Hailer NP, 1996, GLIA, V18, P319, DOI 10.1002/(SICI)1098-1136(199612)18:4<319::AID-GLIA6>3.0.CO;2-S

Hamby ME, 2006, J NEUROSCI METH, V150, P128, DOI 10.1016/j.jneumeth.2005.06.016

Hanisch UK, 2002, GLIA, V40, P140, DOI 10.1002/glia.10161

HARADA T, 1993, ADV EXP MED BIOL, V338, P183

Hayashi Y, 2005, J NEUROTRAUM, V22, P1267, DOI 10.1089/neu.2005.22.1267

He JL, 1997, NATURE, V385, P645, DOI 10.1038/385645a0

Heppner FL, 2005, NAT MED, V11, P146, DOI 10.1038/nm1177

Heyen JRR, 2000, MOL BRAIN RES, V77, P138, DOI 10.1016/S0169-328X(00)00042-5

Hurlbert RJ, 2006, SPINE, V31, pS16, DOI 10.1097/01.brs.0000218264.37914.2c

Ibarra A, 2004, EUR J NEUROSCI, V19, P2984, DOI 10.1111/j.0953-816X.2004.03402.x

Ibarra A, 2006, CURR MED CHEM, V13, P2703, DOI 10.2174/092986706778201503

Jacobsson J, 2006, NEUROSCIENCE, V139, P475, DOI 10.1016/j.neuroscience.2005.12.046

Jaworski J, 2005, J NEUROSCI, V25, P11300, DOI 10.1523/JNEUROSCI.2270-05.2005

Jayakumar AR, 2002, J NEUROCHEM, V83, P1226, DOI 10.1046/j.1471-4159.2002.01261.x

Jiang S, 2004, INT J IMMUNOPATH PH, V17, P353, DOI 10.1177/039463200401700315

Jonsson CA, 2002, CELL IMMUNOL, V216, P93, DOI 10.1016/S0008-8749(02)00502-6

JORGENSEN MB, 1993, EXP NEUROL, V120, P70, DOI 10.1006/exnr.1993.1041

Kaminska B, 2004, J CELL MOL MED, V8, P45, DOI 10.1111/j.1582-4934.2004.tb00259.x

Kaptanoglu E, 2000, J NEUROSURG, V93, P77, DOI 10.3171/spi.2000.93.1.0077

Karpova A, 2006, J NEUROSCI, V26, P4949, DOI 10.1523/JNEUROSCI.4573-05.2006

KIEFER R, 1995, INT J DEV NEUROSCI, V13, P331

Kiefer R, 1998, J NEUROPATH EXP NEUR, V57, P385, DOI 10.1097/00005072-199805000-00002

KIEFER R, 1991, J NEUROIMMUNOL, V34, P99, DOI 10.1016/0165-5728(91)90119-R

KITAMURA T, 1978, ACTA NEUROPATHOL, V44, P31, DOI 10.1007/BF00691636

Kohl A, 2003, EXP NEUROL, V181, P1, DOI 10.1016/S0014-4886(02)00049-3

Koyanagi I, 1997, NEUROL RES, V19, P289, DOI 10.1080/01616412.1997.11740815

Kraus RL, 2005, J NEUROCHEM, V94, P819, DOI 10.1111/j.1471-4159.2005.03219.x

Kremlev SG, 2004, J NEUROIMMUNOL, V149, P1, DOI 10.1016/j.jneuroim.2003.11.012

KREUTZBERG GW, 1968, ACTA NEUROPATHOL B S, V5

Labrande C, 2006, NEUROSCIENCE, V137, P231, DOI 10.1016/j.neuroscience.2005.08.080

Lampl Y, 2007, NEUROLOGY, V69, P1404, DOI 10.1212/01.wnl.0000277487.04281.db

LEE SC, 1993, J IMMUNOL, V150, P2659

Lee SM, 2003, J NEUROTRAUM, V20, P1017, DOI 10.1089/089771503770195867

Lewis RS, 2001, ANNU REV IMMUNOL, V19, P497, DOI 10.1146/annurev.immunol.19.1.497

LING EA, 1979, J ANAT, V128, P847

LING EA, 1982, J ANAT, V134, P705

LIU J, 1991, CELL, V66, P807, DOI 10.1016/0092-8674(91)90124-H

Lockhart BP, 1998, BRIT J PHARMACOL, V123, P879, DOI 10.1038/sj.bjp.0701664

Loddick SA, 1997, BIOCHEM BIOPH RES CO, V234, P211, DOI 10.1006/bbrc.1997.6436

López-Vales R, 2005, J NEUROSCI RES, V81, P827, DOI 10.1002/jnr.20605

Loscher CE, 2003, J NEUROIMMUNOL, V137, P117, DOI 10.1016/S0165-5728(03)00072-9

Lu DY, 2006, BIOCHEM PHARMACOL, V72, P992, DOI 10.1016/j.bcp.2006.06.038

LYONS WE, 1994, P NATL ACAD SCI USA, V91, P3191, DOI 10.1073/pnas.91.8.3191

Madsen JR, 1998, EXP NEUROL, V154, P673, DOI 10.1006/exnr.1998.6974

Mejia ROS, 2001, NEUROSURGERY, V48, P1393, DOI 10.1097/00006123-200106000-00051

Melcangi RC, 2006, NEUROSCIENCE, V138, P733, DOI 10.1016/j.neuroscience.2005.10.066

MENTEN J, 1989, INT J RADIAT ONCOL, V17, P131, DOI 10.1016/0360-3016(89)90380-5

Messina E, 2005, NEUROSCI LETT, V375, P97, DOI 10.1016/j.neulet.2004.10.076

Messina E, 2004, NUCLEOS NUCLEOT NUCL, V23, P1545, DOI 10.1081/NCN-200027770

MICHAELS J, 1988, ACTA NEUROPATHOL, V76, P373, DOI 10.1007/BF00686974

MICHIKAWA M, 1994, J NEUROSCI RES, V37, P62, DOI 10.1002/jnr.490370109

Miljkovic D, 2002, GLIA, V39, P247, DOI 10.1002/glia.10089

Mitrasinovic OM, 2001, J BIOL CHEM, V276, P30142, DOI 10.1074/jbc.M104265200

Miwa T, 1997, J NEUROSCI RES, V50, P1023

Miyata K, 2001, NEUROSCIENCE, V105, P571, DOI 10.1016/S0306-4522(01)00225-1

Morgan SC, 2004, J NEUROCHEM, V90, P89, DOI 10.1111/j.1471-4159.2004.02461.x

MORRIS RE, 1991, IMMUNOL TODAY, V12, P137

MORSHEAD CM, 1990, BRAIN RES, V535, P237, DOI 10.1016/0006-8993(90)91606-H

Mulcahy NJ, 2003, BRIT J PHARMACOL, V140, P471, DOI 10.1038/sj.bjp.0705462

MURPHY S, 1993, TRENDS NEUROSCI, V16, P323, DOI 10.1016/0166-2236(93)90109-Y

NAGY SE, 1993, IMMUNOPHARMACOLOGY, V26, P11, DOI 10.1016/0162-3109(93)90062-U

NASO WB, 1995, NEUROSCI LETT, V189, P176, DOI 10.1016/0304-3940(95)11473-A

NATHAN C, 1992, FASEB J, V6, P3051, DOI 10.1096/fasebj.6.12.1381691

Nimmerjahn A, 2005, SCIENCE, V308, P1314, DOI 10.1126/science.1110647

Nissl F, 1894, ZENTRALBLATT NERVENH, V17, P337

Nolte C, 1996, NEUROSCIENCE, V73, P1091, DOI 10.1016/0306-4522(96)00106-6

NORENBERG W, 1992, NEUROSCI LETT, V147, P171, DOI 10.1016/0304-3940(92)90587-W

NORENBERG W, 1994, J PHYSIOL-LONDON, V475, P15

Norenberg W, 1997, BRIT J PHARMACOL, V121, P1087, DOI 10.1038/sj.bjp.0701241

Nottingham S, 2002, EXP NEUROL, V177, P242, DOI 10.1006/exnr.2002.7975

Oest TM, 2006, HIPPOCAMPUS, V16, P437, DOI 10.1002/hipo.20182

Okonkwo DO, 2006, J NEUROSURG-SPINE, V4, P64, DOI 10.3171/spi.2006.4.1.64

Okonkwo DO, 1999, J CEREBR BLOOD F MET, V19, P443, DOI 10.1097/00004647-199904000-00010

Ono K, 1999, BIOCHEM BIOPH RES CO, V262, P610, DOI 10.1006/bbrc.1999.1223

ORCHINIK M, 1991, SCIENCE, V252, P1848, DOI 10.1126/science.2063198

Oudega M, 1999, EUR J NEUROSCI, V11, P2453, DOI 10.1046/j.1460-9568.1999.00666.x

Palladini G, 1996, J BRAIN RES, V37, P145

Parks WC, 2004, NAT REV IMMUNOL, V4, P617, DOI 10.1038/nri1418

Parsons RG, 2006, J NEUROSCI, V26, P12977, DOI 10.1523/JNEUROSCI.4209-06.2006

Pearse DD, 2004, J NEUROTRAUM, V21, P1223, DOI 10.1089/0897715041953876

PERRY VH, 1985, NEUROSCIENCE, V15, P313, DOI 10.1016/0306-4522(85)90215-5

Pi RB, 2004, J NEUROCHEM, V91, P1219, DOI 10.1111/j.1471-4159.2004.02796.x

Polfliet MMJ, 2001, J NEUROIMMUNOL, V116, P188, DOI 10.1016/S0165-5728(01)00282-X

Postler E, 1997, GLIA, V19, P27, DOI 10.1002/(SICI)1098-1136(199701)19:1<27::AID-GLIA3>3.0.CO;2-7

Pyrzynska B, 2001, NEUROCHEM INT, V38, P409, DOI 10.1016/S0197-0186(00)00105-4

Rabchevsky AG, 2002, J NEUROSCI RES, V68, P7, DOI 10.1002/jnr.10187

Rabchevsky AG, 2001, J NEUROTRAUM, V18, P513, DOI 10.1089/089771501300227314

RAIVICH G, 1994, EUR J NEUROSCI, V6, P1615, DOI 10.1111/j.1460-9568.1994.tb00552.x

Rao A, 1997, ANNU REV IMMUNOL, V15, P707, DOI 10.1146/annurev.immunol.15.1.707

Rappert A, 2002, J IMMUNOL, V168, P3221, DOI 10.4049/jimmunol.168.7.3221

Rappert A, 2004, J NEUROSCI, V24, P8500, DOI 10.1523/JNEUROSCI.2451-04.2004

Raught B, 2001, P NATL ACAD SCI USA, V98, P7037, DOI 10.1073/pnas.121145898

Rio- Hortega PD, 1932, CYTOLOGY CELLULAR PA, P482

SanzRodriguez C, 1997, NEUROSCI LETT, V223, P141, DOI 10.1016/S0304-3940(97)13412-7

SAWADA M, 1992, BIOCHEM BIOPH RES CO, V189, P869, DOI 10.1016/0006-291X(92)92284-5

SAWADA M, 1989, BRAIN RES, V491, P394, DOI 10.1016/0006-8993(89)90078-4

Schilling T, 2004, J PHYSIOL-LONDON, V557, P105, DOI 10.1113/jphysiol.2004.060632

Schilling T, 2004, EUR J NEUROSCI, V19, P1469, DOI 10.1111/j.1460-9568.2004.03265.x

Schneider H, 1998, P NATL ACAD SCI USA, V95, P7778, DOI 10.1073/pnas.95.13.7778

Schneider-Gold C, 2006, MUSCLE NERVE, V34, P284, DOI 10.1002/mus.20543

SCHUMACHER M, 1990, TRENDS NEUROSCI, V13, P359, DOI 10.1016/0166-2236(90)90016-4

Seabrook TJ, 2006, GLIA, V53, P776, DOI 10.1002/glia.20338

SHAFITZAGARDO B, 1993, INT J DEV NEUROSCI, V11, P189, DOI 10.1016/0736-5748(93)90078-R

SHARKEY J, 1994, NATURE, V371, P336, DOI 10.1038/371336a0

Sheehan Jason, 2006, Neurosurg Focus, V20, pE9

SHIBATA M, 1991, BRAIN RES, V562, P323, DOI 10.1016/0006-8993(91)90639-D

SHIGA Y, 1992, BRAIN RES, V595, P145, DOI 10.1016/0006-8993(92)91465-Q

Shimazawa M, 2005, BRAIN RES, V1053, P185, DOI 10.1016/j.brainres.2005.06.053

Shimizu T, 2005, J CEREBR BLOOD F MET, V25, P325, DOI 10.1038/sj.jcbfm.9600029

SIEVERS J, 1994, GLIA, V12, P245, DOI 10.1002/glia.440120402

Singleton RH, 2001, J NEUROTRAUM, V18, P607, DOI 10.1089/089771501750291846

SJOSTRAND J, 1965, Z ZELLFORSCH MIK ANA, V68, P481, DOI 10.1007/BF00347712

Sosa I, 2005, EXP NEUROL, V195, P7, DOI 10.1016/j.expneurol.2005.04.016

Steiner JP, 1997, P NATL ACAD SCI USA, V94, P2019, DOI 10.1073/pnas.94.5.2019

Steiner JP, 1997, NAT MED, V3, P421, DOI 10.1038/nm0497-421

Stence N, 2001, GLIA, V33, P256, DOI 10.1002/1098-1136(200103)33:3<256::AID-GLIA1024>3.0.CO;2-J

Stirling DP, 2004, J NEUROSCI, V24, P2182, DOI 10.1523/JNEUROSCI.5275-03.2004

SUZUMURA A, 1987, J NEUROIMMUNOL, V15, P263, DOI 10.1016/0165-5728(87)90121-4

SUZUMURA A, 1993, J IMMUNOL, V151, P2150

SVENSSON M, 1993, J NEUROSCI RES, V35, P373, DOI 10.1002/jnr.490350404

SVENSSON M, 1993, EUR J NEUROSCI, V5, P85, DOI 10.1111/j.1460-9568.1993.tb00208.x

SVENSSON M, 1993, GLIA, V7, P286, DOI 10.1002/glia.440070404

SVENSSON M, 1993, EXP NEUROL, V120, P123, DOI 10.1006/exnr.1993.1046

Tachibana T, 2005, J THORAC CARDIOV SUR, V129, P123, DOI 10.1016/j.jtcvs.2004.04.047

Takamatsu H, 2001, J NUCL MED, V42, P1833

Takami T, 2002, J NEUROTRAUM, V19, P653, DOI 10.1089/089771502753754118

Tanaka J, 1997, GLIA, V20, P23, DOI 10.1002/(SICI)1098-1136(199705)20:1<23::AID-GLIA3>3.3.CO;2-U

Teichner A, 1993, J Hirnforsch, V34, P343

Teng YD, 2004, P NATL ACAD SCI USA, V101, P3071, DOI 10.1073/pnas.0306239101

Tikka T, 2001, J NEUROSCI, V21, P2580, DOI 10.1523/JNEUROSCI.21-08-02580.2001

TILLEY BC, 1995, ANN INTERN MED, V122, P81, DOI 10.7326/0003-4819-122-2-199501150-00001

Tischmeyer W, 2003, EUR J NEUROSCI, V18, P942, DOI 10.1046/j.1460-9568.2003.02820.x

Tokime T, 1996, NEUROSCI LETT, V206, P81, DOI 10.1016/S0304-3940(96)12438-1

Torvik A, 1975, Acta Neuropathol Suppl, VSuppl 6, P297

TOULMOND S, 1995, BRAIN RES, V671, P261, DOI 10.1016/0006-8993(94)01343-G

Tsirka SE, 1997, J MOL MED, V75, P341, DOI 10.1007/s001090050119

Uchino H, 1995, ACTA PHYSIOL SCAND, V155, P469, DOI 10.1111/j.1748-1716.1995.tb09999.x

ULVESTAD E, 1994, IMMUNOLOGY, V82, P535

ULVESTAD E, 1994, J LEUKOCYTE BIOL, V56, P732, DOI 10.1002/jlb.56.6.732

vanRooijen N, 1996, J IMMUNOL METHODS, V193, P93, DOI 10.1016/0022-1759(96)00056-7

VIJAYAN VK, 1987, EXP NEUROL, V96, P307, DOI 10.1016/0014-4886(87)90049-5

VINCENT SR, 1994, PROG NEUROBIOL, V42, P129, DOI 10.1016/0301-0082(94)90023-X

WAKITA H, 1995, STROKE, V26, P1415, DOI 10.1161/01.STR.26.8.1415

WALZ W, 1993, J NEUROSCI, V13, P4403

Wang M S, 1999, J Spinal Cord Med, V22, P287

Wells JEA, 2003, BRAIN, V126, P1628, DOI 10.1093/brain/awg178

Wennberg L, 2001, TRANSPLANTATION, V71, P1797, DOI 10.1097/00007890-200106270-00016

WHITTEMORE ER, 1993, BRAIN RES, V621, P59, DOI 10.1016/0006-8993(93)90297-Z

Wirjatijasa F, 2002, J NEUROSCI RES, V68, P579, DOI 10.1002/jnr.10254

WOODROOFE MN, 1991, J NEUROIMMUNOL, V33, P227, DOI 10.1016/0165-5728(91)90110-S

Wu YP, 2000, AM J PATHOL, V156, P1849, DOI 10.1016/S0002-9440(10)65058-4

YABALURI N, 1995, MOL PHARMACOL, V48, P690

Yan J, 2006, STEM CELLS, V24, P1976, DOI 10.1634/stemcells.2005-0518

Yrjänheikki J, 1999, P NATL ACAD SCI USA, V96, P13496, DOI 10.1073/pnas.96.23.13496

Yrjänheikki J, 1998, P NATL ACAD SCI USA, V95, P15769, DOI 10.1073/pnas.95.26.15769

Yu Y, 2007, BRAIN RES, V1148, P62, DOI 10.1016/j.brainres.2007.02.022

Zawadzka M, 2005, GLIA, V49, P36, DOI 10.1002/glia.20092

Zemke D, 2004, CLIN NEUROPHARMACOL, V27, P293, DOI 10.1097/01.wnf.0000150867.98887.3e

Zhang J, 2006, J NEUROCHEM, V97, P772, DOI 10.1111/j.1471-4159.2006.03746.x

Zhou Y, 2007, J NEUROCHEM, V102, P667, DOI 10.1111/j.1471-4159.2007.04535.x

ZIELASEK J, 1992, CELL IMMUNOL, V141, P111, DOI 10.1016/0008-8749(92)90131-8

NR 244

TC 148

Z9 177

U1 0

U2 25

PU PERGAMON-ELSEVIER SCIENCE LTD

PI OXFORD

PA THE BOULEVARD, LANGFORD LANE, KIDLINGTON, OXFORD OX5 1GB, ENGLAND

SN 0301-0082

EI 1873-5118

J9 PROG NEUROBIOL

JI Prog. Neurobiol.

PD MAR

PY 2008

VL 84

IS 3

BP 211

EP 233

DI 10.1016/j.pneurobio.2007.12.001

PG 23

WC Neurosciences

WE Science Citation Index Expanded (SCI-EXPANDED)

SC Neurosciences & Neurology

GA 282WP

UT WOS:000254598200001

PM 18262323

DA 2024-03-03

ER

PT J

AU Nadler, Y

Alexandrovich, A

Grigorladis, N

Hartmann, T

Rao, KSJ

Shohami, E

Stein, R

AF Nadler, Yasmine

Alexandrovich, Alexander

Grigorladis, Nikolaos

Hartmann, Tobias

Rao, Kosagi S. Jagannatha

Shohami, Esther

Stein, Reuven

TI Increased expression of the γ-secretase components presenilin-1 and

nicastrin in activated astrocytes and microglia following traumatic

brain injury

SO GLIA

LA English

DT Article

DE closed head injury; brain-stabbing; presenilin; nicastrin; astrocytes;

microglia

ID CLOSED-HEAD-INJURY; AMYLOID PRECURSOR PROTEIN; FAMILIAL

ALZHEIMERS-DISEASE; BETA-SECRETASE; MOUSE-BRAIN; REACTIVE ASTROCYTES;

GLIAL SCAR; IMMUNOHISTOCHEMICAL ANALYSIS; MISSENSE MUTATIONS; ERBB-4

RECEPTOR

AB gamma-Secretase is an aspartyl protease composed of four proteins: presenilin (PS), nicastrin (Net), APH1, and PEN2. These proteins assemble into a membrane complex that cleaves a variety of substrates within the transmembrane domain. The gamma-secretase cleavage products play an important role in various biological processes such as embryonic development and Alzheimer's disease (AD). The major role of gamma-secretase in brain pathology has been linked to AD and to the production of the amyloid P-peptide. However, little is known about the possible role of gamma-secretase following acute brain insult. Here we examined by immunostaining the expression patterns of two gamma-secretase components, PSI and Net, in three paradigms of brain insult in mice: closed head injury, intracerebroventricular injection of LPS, and brain stabbing. Our results show that in naive and sham-injured brains expression of PS1 and Net is restricted mainly to neurons. However, following insult, the expression of both proteins is also observed in nonneuronal cells, consisting of activated astrocytes and microglia. Furthermore, the proteins are coexpressed within the same astrocytes and microglia, implying that these cells exhibit an enhanced gamma-secretase activity following brain damage. In view of the important role played by astrocytes and microglia in brain disorders, our findings suggest that gamma-secretase may participate in brain damage and repair processes by regulating astrocyte and microglia activation and/or function. (C) 2008 Wiley-Liss, Inc.

C1 [Nadler, Yasmine; Stein, Reuven] Tel Aviv Univ, George S Wise Fac Life Sci, Dept Neurobiochem, IL-69978 Tel Aviv, Israel.

[Alexandrovich, Alexander; Shohami, Esther] Hebrew Univ Jerusalem, Dept Pharmacol, IL-91120 Jerusalem, Israel.

[Grigorladis, Nikolaos] Aristotle Univ Thessaloniki, AHEPA Univ Hosp, Dept Neurol B, GR-54636 Thessaloniki, Greece.

[Grigorladis, Nikolaos] Aristotle Univ Thessaloniki, Lab Expt Neurol, GR-54636 Thessaloniki, Greece.

[Hartmann, Tobias] Univ Saarland, Uniklinikum Homburg, D-6600 Saarbrucken, Germany.

[Rao, Kosagi S. Jagannatha] Cent Food Technol Res Inst, Dept Biochem & Nutr, Mysore 570013, Karnataka, India.

C3 Tel Aviv University; Hebrew University of Jerusalem; Aristotle

University of Thessaloniki; Ahepa University Hospital; Aristotle

University of Thessaloniki; Saarland University; Council of Scientific &

Industrial Research (CSIR) - India; CSIR - Central Food Technological

Research Institute (CFTRI)

RP Stein, R (corresponding author), Tel Aviv Univ, George S Wise Fac Life Sci, Dept Neurobiochem, IL-69978 Tel Aviv, Israel.

EM reuvens@post.tau.ac.il

RI Hartmann, Tobias/AAB-8297-2022; kosagisharaf, jagannatha Rao/N-6788-2014

OI kosagisharaf, jagannatha Rao/0000-0003-4290-7666; Hartmann,

Tobias/0000-0001-7481-6430

CR Arumugam TV, 2006, NAT MED, V12, P621, DOI 10.1038/nm1403

Beni-Adani L, 2001, J PHARMACOL EXP THER, V296, P57

Berezovska O, 1998, J NEUROPATH EXP NEUR, V57, P738, DOI 10.1097/00005072-199808000-00003

Blanchard V, 1997, BRAIN RES, V758, P209, DOI 10.1016/S0006-8993(97)00231-X

Blasko I, 2004, J NEURAL TRANSM, V111, P523, DOI 10.1007/s00702-003-0095-6

Block ML, 2007, NAT REV NEUROSCI, V8, P57, DOI 10.1038/nrn2038

Brunkan AL, 2005, J NEUROCHEM, V93, P769, DOI 10.1111/j.1471-4159.2005.03099.x

Chen Y, 1996, J NEUROTRAUM, V13, P557, DOI 10.1089/neu.1996.13.557

Cribbs DH, 1996, NEUROREPORT, V7, P1773, DOI 10.1097/00001756-199607290-00016

De Strooper B, 2005, CELL, V122, P318, DOI 10.1016/j.cell.2005.07.021

De Strooper B, 1998, NATURE, V391, P387, DOI 10.1038/34910

DeKosky ST, 2007, ARCH NEUROL-CHICAGO, V64, P541, DOI 10.1001/archneur.64.4.541

Dewji NN, 2004, P NATL ACAD SCI USA, V101, P1057, DOI 10.1073/pnas.0307290101

Diehlmann A, 1999, J NEUROSCI RES, V56, P405

Duff K, 1996, NATURE, V383, P710, DOI 10.1038/383710a0

EDDLESTON M, 1993, NEUROSCIENCE, V54, P15, DOI 10.1016/0306-4522(93)90380-X

Erlich S, 2000, MOL CELL NEUROSCI, V16, P597, DOI 10.1006/mcne.2000.0894

Fawcett JW, 1999, BRAIN RES BULL, V49, P377, DOI 10.1016/S0361-9230(99)00072-6

Fukumoto H, 2002, ARCH NEUROL-CHICAGO, V59, P1381, DOI 10.1001/archneur.59.9.1381

Gaiano N, 2000, NEURON, V26, P395, DOI 10.1016/S0896-6273(00)81172-1

Garden GA, 2006, J NEUROIMMUNE PHARM, V1, P127, DOI 10.1007/s11481-006-9015-5

Givogri MI, 2006, DEV NEUROSCI-BASEL, V28, P81, DOI 10.1159/000090755

Glezer I, 2007, NEUROSCIENCE, V147, P867, DOI 10.1016/j.neuroscience.2007.02.055

Grandbarbe L, 2003, DEVELOPMENT, V130, P1391, DOI 10.1242/dev.00374

Grandbarbe L, 2007, GLIA, V55, P1519, DOI 10.1002/glia.20553

Guo Z, 2000, NEUROLOGY, V54, P1316, DOI 10.1212/WNL.54.6.1316

HAASS C, 1993, CELL, V75, P1039, DOI 10.1016/0092-8674(93)90312-E

Hartlage-Rübsamen M, 2003, GLIA, V41, P169, DOI 10.1002/glia.10178

Heales SJR, 2004, NEUROCHEM RES, V29, P513, DOI 10.1023/B:NERE.0000014822.69384.0f

Hébert SS, 2004, NEUROBIOL DIS, V17, P260, DOI 10.1016/j.nbd.2004.08.002

Holsinger RMD, 2002, ANN NEUROL, V51, P783, DOI 10.1002/ana.10208

Huynh DP, 1997, J NEUROPATH EXP NEUR, V56, P1009, DOI 10.1097/00005072-199709000-00006

Ikonomovic MD, 2004, EXP NEUROL, V190, P192, DOI 10.1016/j.expneurol.2004.06.011

Kim KS, 1997, BRAIN RES, V757, P159, DOI 10.1016/S0006-8993(97)00243-6

KODAM A, IN PRESS NEUROBIOL A

Koo EH, 2004, NAT MED, V10, pS26, DOI 10.1038/nm1065

Laudon H, 2004, J NEUROCHEM, V89, P44, DOI 10.1046/j.1471-4159.2003.02298.x

Lee J, 2002, NEUROMOL MED, V2, P29

Lee MK, 1996, J NEUROSCI, V16, P7513, DOI 10.1523/jneurosci.16-23-07513.1996

Lesné S, 2003, J BIOL CHEM, V278, P18408, DOI 10.1074/jbc.M300819200

LEVYLAHAD E, 1995, SCIENCE, V269, P973, DOI 10.1126/science.7638622

Lin CH, 2006, EXP NEUROL, V201, P225, DOI 10.1016/j.expneurol.2006.04.014

Logan A, 2002, ADV EXP MED BIOL, V513, P115

Lye TC, 2000, NEUROPSYCHOL REV, V10, P115, DOI 10.1023/A:1009068804787

Magnus T, 2007, J NEUROSCI RES, V85, P2126, DOI 10.1002/jnr.21368

Marambaud P, 2002, EMBO J, V21, P1948, DOI 10.1093/emboj/21.8.1948

Marchetti B, 2005, TRENDS PHARMACOL SCI, V26, P517, DOI 10.1016/j.tips.2005.08.007

MATHEWSON AJ, 1985, BRAIN RES, V327, P61, DOI 10.1016/0006-8993(85)91499-4

Miake H, 1999, ACTA NEUROPATHOL, V98, P337, DOI 10.1007/s004010051090

Moussaoui S, 1996, FEBS LETT, V383, P219, DOI 10.1016/0014-5793(96)00250-5

Murakami D, 2003, ONCOGENE, V22, P1511, DOI 10.1038/sj.onc.1206298

Ni CY, 2001, SCIENCE, V294, P2179, DOI 10.1126/science.1065412

Otto VI, 2001, NEUROREPORT, V12, P2059, DOI 10.1097/00001756-200107030-00053

Pekny M, 2005, GLIA, V50, P427, DOI 10.1002/glia.20207

Pennypacker KR, 1999, BRAIN RES BULL, V48, P539, DOI 10.1016/S0361-9230(99)00031-3

Pitsi D, 2004, J BIOL CHEM, V279, P25333, DOI 10.1074/jbc.M312710200

Plassman BL, 2000, NEUROLOGY, V55, P1158, DOI 10.1212/WNL.55.8.1158

Ramirez MJ, 2001, BRAIN RES, V907, P222, DOI 10.1016/S0006-8993(01)02580-X

Rao VLR, 2003, J NEUROSCI RES, V71, P208, DOI 10.1002/jnr.10486

Ridet JL, 1997, TRENDS NEUROSCI, V20, P570, DOI 10.1016/S0166-2236(97)01139-9

ROBERTS GW, 1994, J NEUROL NEUROSUR PS, V57, P419, DOI 10.1136/jnnp.57.4.419

ROGAEV EI, 1995, NATURE, V376, P775, DOI 10.1038/376775a0

Rossner S, 2005, J NEUROCHEM, V92, P226, DOI 10.1111/j.1471-4159.2004.02857.x

Schmidt OI, 2005, BRAIN RES REV, V48, P388, DOI 10.1016/j.brainresrev.2004.12.028

Seifert G, 2006, NAT REV NEUROSCI, V7, P194, DOI 10.1038/nrn1870

Selkoe D, 2003, ANNU REV NEUROSCI, V26, P565, DOI 10.1146/annurev.neuro.26.041002.131334

Shah S, 2005, CELL, V122, P435, DOI 10.1016/j.cell.2005.05.022

SHAPIRA Y, 1988, CRIT CARE MED, V16, P258, DOI 10.1097/00003246-198803000-00010

SHERRINGTON R, 1995, NATURE, V375, P754, DOI 10.1038/375754a0

Silver J, 2004, NAT REV NEUROSCI, V5, P146, DOI 10.1038/nrn1326

Siman R, 2004, NEUROSCIENCE, V129, P615, DOI 10.1016/j.neuroscience.2004.08.028

Sofroniew MV, 2005, NEUROSCIENTIST, V11, P400, DOI 10.1177/1073858405278321

Suh YH, 2002, PHARMACOL REV, V54, P469, DOI 10.1124/pr.54.3.469

Tanigaki K, 2001, NEURON, V29, P45, DOI 10.1016/S0896-6273(01)00179-9

Tanimukai H, 1998, MOL BRAIN RES, V54, P212, DOI 10.1016/S0169-328X(97)00337-9

Toninelli GF, 2003, NEUROREPORT, V14, P917, DOI 10.1097/01.wnr.0000069962.11849.e6

Uchihara T, 2006, NEUROBIOL AGING, V27, P88, DOI 10.1016/j.neurobiolaging.2004.12.011

Uchihara T, 1996, ACTA NEUROPATHOL, V92, P325, DOI 10.1007/s004010050526

Van Den Heuvel C, 2007, PROG BRAIN RES, V161, P303, DOI 10.1016/S0079-6123(06)61021-2

Volterra A, 2005, NAT REV NEUROSCI, V6, P626, DOI 10.1038/nrn1722

WAXWEILER RJ, 1995, J NEUROTRAUM, V12, P509, DOI 10.1089/neu.1995.12.509

Weggen S, 1998, NEUROREPORT, V9, P3279

Wyss-Coray T, 2006, NAT MED, V12, P1005, DOI 10.1038/nml1484

Yamada T, 1997, EXP NEUROL, V148, P10, DOI 10.1006/exnr.1997.6661

Yatsiv I, 2002, J CEREBR BLOOD F MET, V22, P971, DOI 10.1097/00004647-200208000-00008

Yu G, 2000, NATURE, V407, P48, DOI 10.1038/35024009

NR 86

TC 75

Z9 78

U1 0

U2 3

PU WILEY

PI HOBOKEN

PA 111 RIVER ST, HOBOKEN 07030-5774, NJ USA

SN 0894-1491

EI 1098-1136

J9 GLIA

JI Glia

PD APR

PY 2008

VL 56

IS 5

BP 552

EP 567

DI 10.1002/glia.20638

PG 16

WC Neurosciences

WE Science Citation Index Expanded (SCI-EXPANDED)

SC Neurosciences & Neurology

GA 275KM

UT WOS:000254070300007

PM 18240300

DA 2024-03-03

ER

PT J

AU Utagawa, A

Bramlett, HM

Daniels, L

Lotocki, G

Dekaban, GA

Weaver, LC

Dietrich, WD

AF Utagawa, Akira

Bramlett, Helen M.

Daniels, Linda

Lotocki, George

Dekaban, Gregory A.

Weaver, Lynne C.

Dietrich, W. Dalton

TI Transient blockage of the CD11d/CD18 integrin reduces contusion volume

and macrophage infiltration after traumatic brain injury in rats

SO BRAIN RESEARCH

LA English

DT Article

DE traumatic brain injury; microglia/macrophages; CD11d/CD18 integrin;

adhesion molecules; inflammation; histopathology

ID INTERCELLULAR-ADHESION MOLECULE-1; CLOSED-HEAD INJURY;

SPINAL-CORD-INJURY; FLUID-PERCUSSION INJURY; TNF-ALPHA; INFLAMMATORY

RESPONSE; BARRIER DYSFUNCTION; IMPROVED RECOVERY; MICE DEFICIENT;

CELL-DEATH

AB The early inflammatory response to traumatic brain injury (TBI) may result in secondary damage. The purpose of this study was to evaluate the effects of a transient treatment employing a blocking monoclonal antibody (mAb) to the CD11d/CD18 integrin on histopathological outcome and macrophage infiltration following TBI. A parasagittal fluid percussion (FP) brain injury (1.8-2.1 atm) was induced in male Sprague-Dawley rats. Rats were randomized into two trauma groups, treated (N = 7) and nontreated (N = 8) animals. In the treated group, a mAb to the CD11d subunit of the CD11d/CD18 integrin was administered 30 min, 24 and 48 h after brain injury. Control animals received an isotype-matched irrelevant mAb using the same dose and treatment regimen. At 3 days after TBI, animals were perfusion-fixed for histopathological and immunocytochemical analysis. The anti-CD11d mAb treatment reduced contusion areas as well as overall contusion volume compared to vehicle treated animals. For example, overall contusion volume was reduced from 2.7 +/- 0.5 mm(3) (mean +/- SEM) to 1.4 +/- 0.4 with treatment (p < 0.05). Immunocytochemical studies identifying CD68 immunoreactive macrophages showed that treatment caused significant attenuation of leukocyte infiltration into the contused cortical areas. These data emphasize the beneficial effects of blocking inflammatory cell recruitment into the injured brain on histopathological outcome following traumatic brain injury. (C) 2008 Elsevier B.V. All rights reserved.

C1 [Utagawa, Akira; Bramlett, Helen M.; Daniels, Linda; Lotocki, George; Dietrich, W. Dalton] Univ Miami, Miller Sch Med, Dept Neurol Surg, Miami, FL 33136 USA.

[Utagawa, Akira; Bramlett, Helen M.; Daniels, Linda; Lotocki, George; Dietrich, W. Dalton] Univ Miami, Miller Sch Med, Neurotrauma Res Ctr, Miami, FL 33136 USA.

[Utagawa, Akira; Bramlett, Helen M.; Daniels, Linda; Lotocki, George; Dietrich, W. Dalton] Univ Miami, Miller Sch Med, Miami Project Cure Paralysis, Miami, FL 33136 USA.

[Dekaban, Gregory A.; Weaver, Lynne C.] Univ Western Ontario, Robarts Res Inst, Spinal Cord Injury Team, Biotherapeut Res Grp, London, ON, Canada.

C3 University of Miami; University of Miami; University of Miami; Western

University (University of Western Ontario)

RP Dietrich, WD (corresponding author), Univ Miami, Miller Sch Med, Dept Neurol Surg, 1095 NW 14th Terrace,LPLC 2-30, Miami, FL 33136 USA.

EM ddietrich@miami.edu

RI Dekaban, Gregory/L-1987-2013; Weaver, Lynne C/K-8491-2013

OI Dekaban, Gregory/0000-0002-3087-4660;

FU NINDS NIH HHS [P50 NS030291-15, NS042133, R01 NS042133, R01 NS042133-05,

NS056072, P50 NS030291, R01 NS056072, NS030291] Funding Source: Medline

CR Barone FC, 1999, J CEREBR BLOOD F MET, V19, P819, DOI 10.1097/00004647-199908000-00001

Becker Kyra J, 2002, Curr Med Res Opin, V18 Suppl 2, ps18, DOI 10.1185/030079902125000688

Bethea JR, 2002, CURR OPIN NEUROL, V15, P355, DOI 10.1097/00019052-200206000-00021

BEVILACQUA MP, 1993, ANNU REV IMMUNOL, V11, P767, DOI 10.1146/annurev.iy.11.040193.004003

Bye N, 2007, EXP NEUROL, V204, P220, DOI 10.1016/j.expneurol.2006.10.013

CARLOS TM, 1994, BLOOD, V84, P2068

Carlos TM, 1997, J LEUKOCYTE BIOL, V61, P279, DOI 10.1002/jlb.61.3.279

Chatzipanteli K, 2000, J CEREBR BLOOD F MET, V20, P531, DOI 10.1097/00004647-200003000-00012

Clark RSB, 1996, J NEUROTRAUM, V13, P333, DOI 10.1089/neu.1996.13.333

Csuka E, 1999, J NEUROIMMUNOL, V101, P211, DOI 10.1016/S0165-5728(99)00148-4

del Zoppo G, 2000, BRAIN PATHOL, V10, P95

DIETRICH WD, 1994, ACTA NEUROPATHOL, V87, P250, DOI 10.1007/BF00296740

DIXON CE, 1987, J NEUROSURG, V67, P110, DOI 10.3171/jns.1987.67.1.0110

Farooque M, 1999, NEUROREPORT, V10, P131, DOI 10.1097/00001756-199901180-00024

Faxon DP, 2002, J AM COLL CARDIOL, V40, P1199, DOI 10.1016/S0735-1097(02)02136-8

Furuya K, 2001, STROKE, V32, P2665, DOI 10.1161/hs3211.098535

Gong C, 2000, BRAIN RES, V871, P57, DOI 10.1016/S0006-8993(00)02427-6

Grady MS, 1999, J NEUROTRAUM, V16, P13, DOI 10.1089/neu.1999.16.13

Grayson MH, 1999, INT ARCH ALLERGY IMM, V118, P263, DOI 10.1159/000024094

Gris D, 2004, J NEUROSCI, V24, P4043, DOI 10.1523/JNEUROSCI.5343-03.2004

Hamada Y, 1996, J NEUROCHEM, V66, P1525

Loddick S.A., 2002, IMMUN INFLAMM DIS, P90

Mabon PJ, 2000, EXP NEUROL, V166, P52, DOI 10.1006/exnr.2000.7488

McIntosh TK, 1998, NEUROPATH APPL NEURO, V24, P251

Morganti-Kossmann MC, 2001, SHOCK, V16, P165, DOI 10.1097/00024382-200116030-00001

MUIZELAAR JP, 1991, J NEUROSURG, V75, P731, DOI 10.3171/jns.1991.75.5.0731

NEISH AS, 1995, MOL CELL BIOL, V15, P2558

Nguyen HX, 2007, J NEUROCHEM, V102, P900, DOI 10.1111/j.1471-4159.2007.04643.x

Noti JD, 2000, J BIOL CHEM, V275, P8959, DOI 10.1074/jbc.275.12.8959

Otto VI, 2001, NEUROREPORT, V12, P2059, DOI 10.1097/00001756-200107030-00053

Paxinos G., 1997, RAT BRAIN STEROTAXIC, VThird

Raghupathi R, 2004, BRAIN PATHOL, V14, P215, DOI 10.1111/j.1750-3639.2004.tb00056.x

Rancan M, 2004, J CEREBR BLOOD F MET, V24, P1110, DOI 10.1097/01.WCB.0000133470.91843.72

Rodriguez-Paez AC, 2005, ACTA NEUROPATHOL, V109, P603, DOI 10.1007/s00401-005-1010-z

Saville LR, 2004, J NEUROIMMUNOL, V156, P42, DOI 10.1016/j.jneuroim.2004.07.002

Scherbel U, 1999, P NATL ACAD SCI USA, V96, P8721, DOI 10.1073/pnas.96.15.8721

Scholz M, 2007, MED RES REV, V27, P401, DOI 10.1002/med.20064

Shanley TP, 1998, J IMMUNOL, V160, P1014

Sherman DG, 2001, NEUROLOGY, V57, P1428, DOI 10.1212/wnl.57.8.1428

SHOHAMI E, 1994, J CEREBR BLOOD F MET, V14, P615, DOI 10.1038/jcbfm.1994.76

Shohami E, 1996, J CEREBR BLOOD F MET, V16, P378, DOI 10.1097/00004647-199605000-00004

Sinz EH, 1999, J CLIN INVEST, V104, P647, DOI 10.1172/JCI6670

SMITH CW, 1993, SEMIN HEMATOL, V30, P45

Stahel PF, 2000, J CEREBR BLOOD F MET, V20, P369, DOI 10.1097/00004647-200002000-00019

Suzuki T, 2004, J NEUROTRAUM, V21, P842, DOI 10.1089/0897715041526186

Tehranian R, 2002, J NEUROTRAUM, V19, P939, DOI 10.1089/089771502320317096

TOULMOND S, 1995, BRAIN RES, V671, P261, DOI 10.1016/0006-8993(94)01343-G

Van der Vieren M, 1999, J IMMUNOL, V163, P1984

VanderVieren M, 1995, IMMUNITY, V3, P683, DOI 10.1016/1074-7613(95)90058-6

Whalen MJ, 1998, J NEUROTRAUM, V15, P777, DOI 10.1089/neu.1998.15.777

Whalen MJ, 1999, J NEUROTRAUM, V16, P299, DOI 10.1089/neu.1999.16.299

Zausinger S, 2002, BRAIN RES PROTOC, V9, P112, DOI 10.1016/S1385-299X(02)00138-1

ZHANG RL, 1995, STROKE, V26, P1438, DOI 10.1161/01.STR.26.8.1438

ZILLES L, 1985, CORTEX RAT STEREOTAX

NR 54

TC 37

Z9 40

U1 2

U2 6

PU ELSEVIER

PI AMSTERDAM

PA RADARWEG 29, 1043 NX AMSTERDAM, NETHERLANDS

SN 0006-8993

EI 1872-6240

J9 BRAIN RES

JI Brain Res.

PD MAY 1

PY 2008

VL 1207

BP 155

EP 163

DI 10.1016/j.brainres.2008.02.057

PG 9

WC Neurosciences

WE Science Citation Index Expanded (SCI-EXPANDED)

SC Neurosciences & Neurology

GA 299AW

UT WOS:000255729200016

PM 18374312

OA Green Accepted

DA 2024-03-03

ER

PT J

AU Suma, T

Koshinaga, M

Fukushima, M

Kano, T

Katayama, Y

AF Suma, Takeshi

Koshinaga, Morimichi

Fukushima, Masamichi

Kano, Tsuneo

Katayama, Yoichi

TI Effects of <i>in situ</i> administration of excitatory amino acid

antagonists on rapid microglial and astroglial reactions in rat

hippocampus following traumatic brain injury

SO NEUROLOGICAL RESEARCH

LA English

DT Article; Proceedings Paper

CT American-Heart-Association International Stroke Conference 2008

CY FEB 20-22, 2008

CL New Orleans, LA

DE astroglial swelling; excitatory amino acid; microglial reaction;

traumatic brain injury; traumatic depolarization

ID SPREADING DEPRESSION; EXTRACELLULAR POTASSIUM; GLUCOSE-UTILIZATION;

CORTICAL CONTUSION; CEREBRAL-CORTEX; SPINAL-CORD; ASTROCYTES; GLUTAMATE;

RELEASE; CELLS

AB Objective: Both microglia and astrocytes respond immediately to traumatic brain injury (TBI). The present study was undertaken to examine whether or not excitatory amino acid (EAA) antagonists could attenuate such glial responses.

Methods: EAA antagonists, including the broad spectrum EAA antagonist, kynurenic acid (KYN), specific N-methyl-D-aspartate (NMDA) receptor blocker, 2-amino-5-phosphonovalerate (AP-5), and AMPA-KA receptor blocker, 6,7-dinitroquinoxaline-2,3-dione (DNQX), as well as the voltage-dependent ion channel blocker, tetrodotoxin (TTX), were administered into the unilateral hippocampus of rats through a dialysis probe for 30 minutes before the induction of unilateral controlled cortical impact injury. The rats were killed 10 minutes after injury and their brains were processed immunohistochemically for OX42 (marker for microglia) and glial fibrillary acidic protein (GFAP; marker for astrocytes).

Objective: Ten minutes after injury, microglial activation with increased OX42 immunoreactivity was evident in the entire hemisphere including the hippocampus ipsilateral to the injury side. Similarly, swollen astrocytes with increased GFAP expression could be detected exclusively on the injury side. When KYN was administered in situ before injury, both the rapid microglial and astroglial responses in the hippocampus were significantly attenuated. However, AP-5, DNQX and TTX, the voltage-dependent ion channel blocker, at doses which can inhibit each channel activation, failed to attenuate these glial reactions.

Discussion: These findings indicate that massive ionic fluxes and/or concomitantly occurring EAA release may be closely related to the initiation of microglial and astroglial responses following TBI.

C1 [Koshinaga, Morimichi] Nihon Univ, Sch Med, Dept Anat, Itabashi Ku, Tokyo 1738610, Japan.

[Suma, Takeshi; Koshinaga, Morimichi; Fukushima, Masamichi; Kano, Tsuneo; Katayama, Yoichi] Nihon Univ, Sch Med, Dept Neurol Surg, Itabashi Ku, Tokyo 1738610, Japan.

C3 Nihon University; Nihon University

RP Koshinaga, M (corresponding author), Nihon Univ, Sch Med, Dept Anat, Itabashi Ku, 30-1 Oyaguchi Kamimachi, Tokyo 1738610, Japan.

EM koshimo@med.nihon-u.ac.jp

CR Abe K, 2000, BIOL PHARM BULL, V23, P1051

Anderson MF, 2003, NEUROCHEM RES, V28, P293, DOI 10.1023/A:1022385402197

AQUINO DA, 1988, J NEUROCHEM, V51, P1085, DOI 10.1111/j.1471-4159.1988.tb03072.x

BARRES BA, 1990, ANNU REV NEUROSCI, V13, P441, DOI 10.1146/annurev.ne.13.030190.002301

BENVENISTE EN, 1997, IMMUNOLOGY NERVOUS S, P419

Bruce-Keller AJ, 1999, J NEUROSCI RES, V58, P191, DOI 10.1002/(SICI)1097-4547(19991001)58:1<191::AID-JNR17>3.0.CO;2-E

Caggiano AO, 1996, J COMP NEUROL, V369, P93, DOI 10.1002/(SICI)1096-9861(19960520)369:1<93::AID-CNE7>3.0.CO;2-F

Chang RCC, 2000, NEUROSCIENCE, V97, P757, DOI 10.1016/S0306-4522(00)00059-2

Chen YM, 2003, J CEREBR BLOOD F MET, V23, P137, DOI 10.1097/01.WCB.0000044631.80210.3C

Chung S, 1998, NEUROSCI LETT, V242, P73, DOI 10.1016/S0304-3940(98)00029-9

Del Rio-Hortega R., 1965, CYTOLOGY CELLULAR PA, P483

Dietrich WD, 1999, J NEUROTRAUM, V16, P567, DOI 10.1089/neu.1999.16.567

DIETRICH WD, 1994, J NEUROTRAUM, V11, P289, DOI 10.1089/neu.1994.11.289

DIXON CE, 1991, J NEUROSCI METH, V39, P253

Dombro RS, 2000, INT J DEV NEUROSCI, V18, P161, DOI 10.1016/S0736-5748(99)00084-2

Duchen L. W, 1984, GREENFIELDS NEUROPAT, P1

Eder C, 1999, EUR J NEUROSCI, V11, P4251, DOI 10.1046/j.1460-9568.1999.00852.x

GEHRMANN J, 1993, BRAIN PATHOL, V3, P11, DOI 10.1111/j.1750-3639.1993.tb00720.x

GIULIAN D, 1994, DEV NEUROSCI-BASEL, V16, P128, DOI 10.1159/000112099

GIULIAN D, 1994, NEUROCHEM INT, V25, P227, DOI 10.1016/0197-0186(94)90066-3

GIULIAN D, 1990, ANN NEUROL, V27, P33, DOI 10.1002/ana.410270107

HAMBERGER A, 1979, BRAIN RES, V168, P531, DOI 10.1016/0006-8993(79)90307-X

Hansson E, 2003, FASEB J, V17, P341, DOI 10.1096/fj.02-0429rev

Hinkle DA, 1997, J NEUROTRAUM, V14, P729, DOI 10.1089/neu.1997.14.729

Jensen MB, 1997, EXP NEUROL, V143, P103, DOI 10.1006/exnr.1996.6337

KATAYAMA Y, 1995, BRAIN PATHOL, V5, P427, DOI 10.1111/j.1750-3639.1995.tb00621.x

KATAYAMA Y, 1990, J NEUROSURG, V73, P889, DOI 10.3171/jns.1990.73.6.0889

KATZ B, 1967, PROC R SOC SER B-BIO, V167, P8, DOI 10.1098/rspb.1967.0010

KAWAMATA T, 1992, J CEREBR BLOOD F MET, V12, P12, DOI 10.1038/jcbfm.1992.3

KETTENMANN H, 1990, J NEUROSCI RES, V26, P278, DOI 10.1002/jnr.490260303

KETTENMANN H, 1993, GLIA, V7, P93, DOI 10.1002/glia.440070115

Koshinaga M, 2000, J NEUROTRAUM, V17, P185, DOI 10.1089/neu.2000.17.185

Kraig Richard P., 1995, P964

KRAIG RP, 1991, J NEUROSCI, V11, P2187

KREUTZBERG GW, 1987, ENCY NEUROSCIENCE, P661

Maeda T, 1997, ACT NEUR S, V70, P102

Maeda T, 1998, J NEUROTRAUM, V15, P655, DOI 10.1089/neu.1998.15.655

Miele M, 1996, J PHYSIOL-LONDON, V497, P745, DOI 10.1113/jphysiol.1996.sp021805

MURPHY S, 1987, NEUROSCIENCE, V22, P381, DOI 10.1016/0306-4522(87)90342-3

NARAHASHI T, 1964, J GEN PHYSIOL, V47, P965, DOI 10.1085/jgp.47.5.965

Noda M, 1999, NEUROSCIENCE, V92, P1465, DOI 10.1016/S0306-4522(99)00036-6

NORENBERG MD, 1982, J NEUROPATH EXP NEUR, V41, P347, DOI 10.1097/00005072-198205000-00026

OCHS S, 1971, J NEUROCHEM, V18, P107, DOI 10.1111/j.1471-4159.1971.tb00172.x

PIANI D, 1991, NEUROSCI LETT, V133, P159, DOI 10.1016/0304-3940(91)90559-C

PUEL JL, 1994, J COMP NEUROL, V341, P241, DOI 10.1002/cne.903410209

Queen SA, 1997, BRAIN RES, V777, P42, DOI 10.1016/S0006-8993(97)00717-8

Ridet JL, 1997, TRENDS NEUROSCI, V20, P570, DOI 10.1016/S0166-2236(97)01139-9

SHIMOHAMA S, 1989, MOL BRAIN RES, V5, P271, DOI 10.1016/0169-328X(89)90061-2

Sperlágh B, 1998, NEUROSCIENCE, V86, P1195, DOI 10.1016/S0306-4522(98)00026-8

STYS PK, 1993, P NATL ACAD SCI USA, V90, P6976, DOI 10.1073/pnas.90.15.6976

SUGAYA E, 1975, J NEUROPHYSIOL, V38, P822, DOI 10.1152/jn.1975.38.4.822

SWANSON RA, 1992, NEUROSCI LETT, V147, P143, DOI 10.1016/0304-3940(92)90580-Z

THOMAS WE, 1992, BRAIN RES REV, V17, P61

Vernadakis A, 1996, PROG NEUROBIOL, V49, P185, DOI 10.1016/S0301-0082(96)00012-3

WALZ W, 1989, PROG NEUROBIOL, V33, P309, DOI 10.1016/0301-0082(89)90005-1

WALZ W, 1993, J NEUROSCI, V13, P4403

Walz W, 2000, NEUROCHEM INT, V36, P291, DOI 10.1016/S0197-0186(99)00137-0

NR 57

TC 16

Z9 22

U1 0

U2 1

PU TAYLOR & FRANCIS LTD

PI ABINGDON

PA 2-4 PARK SQUARE, MILTON PARK, ABINGDON OR14 4RN, OXON, ENGLAND

SN 0161-6412

EI 1743-1328

J9 NEUROL RES

JI Neurol. Res.

PD MAY

PY 2008

VL 30

IS 4

BP 420

EP 429

DI 10.1179/016164107X251745

PG 10

WC Clinical Neurology; Neurosciences

WE Science Citation Index Expanded (SCI-EXPANDED); Conference Proceedings Citation Index - Science (CPCI-S)

SC Neurosciences & Neurology

GA 314VR

UT WOS:000256837800019

PM 18248696

DA 2024-03-03

ER

PT J

AU Zhang, Z

Fauser, U

Schluesener, HJ

AF Zhang, Z.

Fauser, U.

Schluesener, H. J.

TI Early attenuation of lesional interleukin-16 up-regulation by

dexamethasone and FTY720 in experimental traumatic brain injury

SO NEUROPATHOLOGY AND APPLIED NEUROBIOLOGY

LA English

DT Article

DE dexamethasone; FTY720; interleukin-16; microglia; macrophages; traumatic

brain injury

ID SPINAL-CORD-INJURY; EXPERIMENTAL AUTOIMMUNE ENCEPHALOMYELITIS;

HEAD-INJURY; ACTIVATED MICROGLIA/MACROPHAGES; INFLAMMATORY CYTOKINES;

TIRILAZAD MESYLATE; CEREBRAL-ISCHEMIA; MICROGLIAL CELLS; HUMAN

MONOCYTES; NERVOUS-SYSTEM

AB Aims: Interleukin-16 (IL16) is an immunomodulatory cytokine, which induces lymphocyte migration, expression of proinflammatory IL1 beta, IL6 and tumour necrosis factor-alpha, and modulates apoptosis. IL16 expression has been observed in several central nervous system diseases and may play a role in promoting inflammatory responses. Inflammation contributes considerably to secondary injury following traumatic brain injury (TBI). The aim of this study was to investigate early IL16 expression following experimental TBI and the effects of dexamethasone and FTY720 on early expression of IL16 in TBI rats. Methods: Rat TBI was induced using an open-skull weight-drop model. IL16 expression was studied by immunohistochemistry. TBI rats received an intraperitoneal injection of dexamethasone (1 mg/kg in 1 ml saline), FTY720 (1 mg/kg in 1 ml saline) or saline (1 ml) on Day 0 and Day 2 immediately after surgery. Results: Significant up-regulation of IL16 was seen as early as 24 h post TBI. Double-staining experiments, together with morphological classification, revealed a multicellular origin of IL16, including activated microglia/macrophages (about 85%), astrocytes (about 8%), neurones (about 5%) and granulocytes. Following peripheral administration of dexamethasone and FTY720, attenuated numbers of IL16(+) cells were observed on Days 1 and 2 but not on Day 4 post TBI for dexamethasone and on Day 4 but not earlier for FTY720 respectively. Conclusions: Our observations reveal that dexamethasone and FTY720 have different but complementary effects on reduction of early IL16 expression following TBI.

C1 [Zhang, Z.; Fauser, U.; Schluesener, H. J.] Univ Tubingen, Inst Brain Res, D-72076 Tubingen, Germany.

C3 Eberhard Karls University of Tubingen

RP Zhang, Z (corresponding author), Univ Tubingen, Inst Brain Res, Calwer St 3, D-72076 Tubingen, Germany.

EM zhangzhiren@yahoo.com

RI Zhang, Zhiren/O-1012-2019

OI Zhang, Zhiren/0000-0002-5238-2835

CR ADACHI K, 1995, BIOORG MED CHEM LETT, V5, P853, DOI 10.1016/0960-894X(95)00127-F

Arima M, 1999, AM J RESP CELL MOL, V21, P684, DOI 10.1165/ajrcmb.21.6.3671

Bethea JR, 2002, CURR OPIN NEUROL, V15, P355, DOI 10.1097/00019052-200206000-00021

Brinkmann V, 2002, J BIOL CHEM, V277, P21453, DOI 10.1074/jbc.C200176200

Chiba K, 2006, CELL MOL IMMUNOL, V3, P11

Cruikshank WW, 2000, J LEUKOCYTE BIOL, V67, P757, DOI 10.1002/jlb.67.6.757

DAMOISEAUX JGMC, 1994, IMMUNOLOGY, V83, P140

Deng JM, 2006, CHINESE MED J-PEKING, V119, P1017, DOI 10.1097/00029330-200606020-00009

Dirnagl U, 1999, TRENDS NEUROSCI, V22, P391, DOI 10.1016/S0166-2236(99)01401-0

Feuerstein GZ, 1997, ANN NY ACAD SCI, V825, P179, DOI 10.1111/j.1749-6632.1997.tb48428.x

Gahm C, 2005, ACTA NEUROCHIR, V147, P1071, DOI 10.1007/s00701-005-0590-7

Ghirnikar RS, 1998, NEUROCHEM RES, V23, P329, DOI 10.1023/A:1022453332560

Gottesfeld Z, 2002, J NEUROTRAUM, V19, P317, DOI 10.1089/089771502753594882

Guo LH, 2004, J NEUROIMMUNOL, V146, P39, DOI 10.1016/j.jneuroim.2003.09.017

HALL ED, 1987, J PHARMACOL EXP THER, V242, P137

Holmin, 1996, ACTA NEUROCHIR, V138, P418, DOI 10.1007/BF01420304

Kaneider NC, 2004, FASEB J, V18, P1309, DOI 10.1096/fj.03-1050fje

Kappos L, 2006, NEW ENGL J MED, V355, P1124, DOI 10.1056/NEJMoa052643

Kerschensteiner M, 1999, J EXP MED, V189, P865, DOI 10.1084/jem.189.5.865

Klimiuk PA, 1999, J IMMUNOL, V162, P4293

Laberge S, 1997, J ALLERGY CLIN IMMUN, V100, P569, DOI 10.1016/S0091-6749(97)70152-0

Lee JM, 1999, NATURE, V399, pA7, DOI 10.1038/399a007

Leker RR, 2002, BRAIN RES REV, V39, P55, DOI 10.1016/S0165-0173(02)00157-1

Liebrich M, 2007, ARCH IMMUNOL THER EX, V55, P41, DOI 10.1007/s00005-007-0003-0

Lipton P, 1999, PHYSIOL REV, V79, P1431, DOI 10.1152/physrev.1999.79.4.1431

Marshall LF, 1998, J NEUROSURG, V89, P519, DOI 10.3171/jns.1998.89.4.0519

Mathy NL, 2000, IMMUNOLOGY, V100, P63, DOI 10.1046/j.1365-2567.2000.00997.x

MEINIG G, 1990, ACT NEUR S, V51, P100

Morganti-Kossmann Maria Cristina, 2002, Curr Opin Crit Care, V8, P101

Morganti-Kossmann MC, 2001, SHOCK, V16, P165, DOI 10.1097/00024382-200116030-00001

Mueller CA, 2006, J NEUROSURG-SPINE, V4, P233, DOI 10.3171/spi.2006.4.3.233

Muzha I, 2004, LANCET, V364, P1321, DOI 10.1016/S0140-6736(04)17188-2

Narayan RK, 2002, J NEUROTRAUM, V19, P503, DOI 10.1089/089771502753754037

Raivich G, 1999, ACTA NEUROCHIR SUPPL, V73, P21

Rausch M, 2004, J MAGN RESON IMAGING, V20, P16, DOI 10.1002/jmri.20057

Sauerland S, 2004, LANCET, V364, P1291, DOI 10.1016/S0140-6736(04)17202-4

Schluesener HJ, 1997, GLIA, V20, P365, DOI 10.1002/(SICI)1098-1136(199708)20:4<365::AID-GLIA8>3.0.CO;2-4

Schwab JM, 2001, J NEUROIMMUNOL, V114, P232, DOI 10.1016/S0165-5728(00)00433-1

Schwab JM, 2005, J NEUROIMMUNOL, V163, P185, DOI 10.1016/j.jneuroim.2005.02.016

Skundric DS, 2005, J NEUROSCI RES, V79, P680, DOI 10.1002/jnr.20377

Stoll G, 2002, ADV EXP MED BIOL, V513, P87

Webb M, 2004, J NEUROIMMUNOL, V153, P108, DOI 10.1016/j.jneuroim.2004.04.015

Yopp AC, 2006, CLIN TRANSPLANT, V20, P788, DOI 10.1111/j.1399-0012.2006.00570.x

Zhang Z, 2006, NEUROSCIENCE, V141, P637, DOI 10.1016/j.neuroscience.2006.04.027

ZHANG Z, J CELL MOL MED, V11, P307

Zhang ZY, 2007, ACTA NEUROPATHOL, V113, P675, DOI 10.1007/s00401-007-0195-8

NR 46

TC 31

Z9 35

U1 0

U2 8

PU WILEY

PI HOBOKEN

PA 111 RIVER ST, HOBOKEN 07030-5774, NJ USA

SN 0305-1846

EI 1365-2990

J9 NEUROPATH APPL NEURO

JI Neuropathol. Appl. Neurobiol.

PD JUN

PY 2008

VL 34

IS 3

BP 330

EP 339

DI 10.1111/j.1365-2990.2007.00893.x

PG 10

WC Clinical Neurology; Neurosciences; Pathology

WE Science Citation Index Expanded (SCI-EXPANDED)

SC Neurosciences & Neurology; Pathology

GA 292SU

UT WOS:000255287200007

PM 17983426

DA 2024-03-03

ER

PT J

AU Zhang, B

West, EJ

Van, KC

Gurkoff, GG

Zhou, J

Zhang, XM

Kozikowski, AP

Lyeth, BG

AF Zhang, Bin

West, Eric J.

Van, Ken C.

Gurkoff, Gene G.

Zhou, Jia

Zhang, Xiu-Mei

Kozikowski, Alan P.

Lyeth, Bruce G.

TI HDAC inhibitor increases histone H3 acetylation and reduces microglia

inflammatory response following traumatic brain injury in rats

SO BRAIN RESEARCH

LA English

DT Article

DE traumatic brain injury; microglia; inflammation; histone deacetylase;

fluid percussion

ID LATERAL FLUID-PERCUSSION; ACUTE NEURONAL DEGENERATION; TRANSGENIC MOUSE

MODEL; DEACETYLASE INHIBITORS; VALPROIC ACID; NEURODEGENERATIVE

DISEASES; BEHAVIORAL DEFICITS; HUNTINGTONS-DISEASE; ALZHEIMERS-DISEASE;

SODIUM-BUTYRATE

AB Traumatic brain injury (TBI) produces a rapid and robust inflammatory response in the brain characterized in part by activation of microglia. A novel histone deacetylase (HDAC) inhibitor, 4-dimethylamino-N-[5-(2-mercaptoacetylamino)pentyl]benzamide (DMA-PB), was administered (0, 0.25, 2.5, 25 mg/kg) systemically immediately after lateral fluid percussion TBI in rats. Hippocampal CA2/3 tissue was processed for acetyl-histone H3 immunolocalization, OX-42 immunolocalization (for microglia), and Fluoro-Jade B histofluorescence (for degenerating neurons) at 24 h after injury. Vehicle-treated TBI rats exhibited a significant reduction in acetyl-histone H3 immunostaining in the ipsilateral CA2/3 hippocampus compared to the sham TBI group (p<0.05). The reduction in acetyl-histone H3 immunostaining was attenuated by each of the DMA-PB dosage treatment groups. Vehicle-treated TBI rats exhibited a high density of phagocytic microglia in the ipsilateral CA2/3 hippocampus compared to sham TBI in which none were observed. All doses of DMA-PB significantly reduced the density of phagocytic microglia (P<0.05). There was a trend for DMA-PB to reduce the number of degenerating neurons in the ipsilateral CA2/3 hippocampus (p = 0.076). We conclude that the HDAC inhibitor DMA-PB is a potential novel therapeutic for inhibiting neuroinflammation associated with TBI. (C) 2008 Elsevier B.V. All rights reserved.

C1 [Zhang, Bin; West, Eric J.; Van, Ken C.; Gurkoff, Gene G.; Lyeth, Bruce G.] Univ Calif Davis, Dept Neurol Surg, Davis, CA 95616 USA.

[Zhang, Bin; Zhang, Xiu-Mei] Shandong Univ, Sch Med, Dept Pharmacol, Jinan 250012, Shandong, Peoples R China.

[Kozikowski, Alan P.] Univ Illinois, Drug Discovery Program, Dept Med Chem & Pharmacognosy, Chicago, IL 60612 USA.

[Zhou, Jia] PsychoGen Inc, Tarrytown, NY 10591 USA.

C3 University of California System; University of California Davis;

Shandong University; University of Illinois System; University of

Illinois Chicago; University of Illinois Chicago Hospital

RP Lyeth, BG (corresponding author), Univ Calif Davis, Dept Neurol Surg, 1515 Newton Court,1 Shields Ave, Davis, CA 95616 USA.

EM bglyeth@ucdavis.edu

OI Lyeth, Bruce/0000-0003-4811-1474

FU NIH [NS29995, NS45136]

FX This research was supported by NIH NS29995, NS45136 to BGL.

CR Adcock IM, 2007, BRIT J PHARMACOL, V150, P829, DOI 10.1038/sj.bjp.0707166

AIHARA N, 1995, J NEUROTRAUM, V12, P53, DOI 10.1089/neu.1995.12.53

Atkins CM, 2007, EXP NEUROL, V208, P145, DOI 10.1016/j.expneurol.2007.08.011

Butler KV, 2008, CURR PHARM DESIGN, V14, P505

Camelo S, 2005, J NEUROIMMUNOL, V164, P10, DOI 10.1016/j.jneuroim.2005.02.022

Carbonnel WS, 1999, ACTA NEUROPATHOL, V98, P396, DOI 10.1007/s004010051100

Chen B, 2005, BIOORG MED CHEM LETT, V15, P1389, DOI 10.1016/j.bmcl.2005.01.006

Chen PS, 2007, NEUROSCIENCE, V149, P203, DOI 10.1016/j.neuroscience.2007.06.053

Chung YL, 2003, MOL THER, V8, P707, DOI 10.1016/S1525-0016(03)00235-1

De Ruijter AJM, 2003, BIOCHEM J, V370, P737, DOI 10.1042/BJ20021321

Dietrich WD, 2004, ACTA NEUROCHIR SUPPL, V89, P69

DIXON CE, 1987, J NEUROSURG, V67, P110, DOI 10.3171/jns.1987.67.1.0110

Dompierre JP, 2007, J NEUROSCI, V27, P3571, DOI 10.1523/JNEUROSCI.0037-07.2007

Faraco G, 2006, MOL PHARMACOL, V70, P1876, DOI 10.1124/mol.106.027912

Ferrante RJ, 2003, J NEUROSCI, V23, P9418

Gao WM, 2006, BRAIN RES, V1070, P31, DOI 10.1016/j.brainres.2005.11.038

Garden GA, 2006, J NEUROIMMUNE PHARM, V1, P127, DOI 10.1007/s11481-006-9015-5

Gardian G, 2005, J BIOL CHEM, V280, P556, DOI 10.1074/jbc.M410210200

Gehrmann Jochen, 1995, P883

GIULIAN D, 1990, ANN NEUROL, V27, P33, DOI 10.1002/ana.410270107

Glauben R, 2006, J IMMUNOL, V176, P5015, DOI 10.4049/jimmunol.176.8.5015

Hallam TM, 2004, J NEUROTRAUM, V21, P521, DOI 10.1089/089771504774129865

HUITINGA I, 1990, J EXP MED, V172, P1025, DOI 10.1084/jem.172.4.1025

Huuskonen J, 2004, BRIT J PHARMACOL, V141, P874, DOI 10.1038/sj.bjp.0705682

Jellinger KA, 2001, EUR J NEUROL, V8, P707, DOI 10.1046/j.1468-1331.2001.00322.x

JIANG JY, 1991, J NEUROSURG, V74, P492, DOI 10.3171/jns.1991.74.3.0492

Kawaguchi Y, 2003, CELL, V115, P727, DOI 10.1016/S0092-8674(03)00939-5

Kim HJ, 2007, J PHARMACOL EXP THER, V321, P892, DOI 10.1124/jpet.107.120188

KOZIKOWSKI AP, 2007, J MED CHEM

Kreutzberg GW, 1996, TRENDS NEUROSCI, V19, P312, DOI 10.1016/0166-2236(96)10049-7

Langlois JA., 2006, TRAUMATIC BRAIN INJU

Lin HS, 2007, BRIT J PHARMACOL, V150, P862, DOI 10.1038/sj.bjp.0707165

Liu B, 2003, J PHARMACOL EXP THER, V304, P1, DOI 10.1124/jpet.102.035048

Lyeth BG, 2001, EXP NEUROL, V169, P191, DOI 10.1006/exnr.2001.7643

McGeer PL, 1998, ALZ DIS ASSOC DIS, V12, pS1

McIntosh TK, 1998, J NEUROTRAUM, V15, P731, DOI 10.1089/neu.1998.15.731

MCINTOSH TK, 1989, NEUROSCIENCE, V28, P233, DOI 10.1016/0306-4522(89)90247-9

Morganti-Kossmann MC, 2007, INJURY, V38, P1392, DOI 10.1016/j.injury.2007.10.005

Morganti-Kossmann Maria Cristina, 2002, Curr Opin Crit Care, V8, P101

Nemetz PN, 1999, AM J EPIDEMIOL, V149, P32

Nishida K, 2004, ARTHRITIS RHEUM-US, V50, P3365, DOI 10.1002/art.20709

Pandey UB, 2007, AUTOPHAGY, V3, P643, DOI 10.4161/auto.5050

Petri S, 2006, NEUROBIOL DIS, V22, P40, DOI 10.1016/j.nbd.2005.09.013

Popovich PG, 1999, EXP NEUROL, V158, P351, DOI 10.1006/exnr.1999.7118

Popovich PG, 2002, J NEUROPATH EXP NEUR, V61, P623, DOI 10.1093/jnen/61.7.623

Ren M, 2004, J NEUROCHEM, V89, P1358, DOI 10.1111/j.1471-4159.2004.02406.x

Saha RN, 2006, CELL DEATH DIFFER, V13, P539, DOI 10.1038/sj.cdd.4401769

Schmued LC, 1997, BRAIN RES, V751, P37, DOI 10.1016/S0006-8993(96)01387-X

Schmued LC, 2000, TOXICOL PATHOL, V28, P91, DOI 10.1177/019262330002800111

Sinn DI, 2007, NEUROBIOL DIS, V26, P464, DOI 10.1016/j.nbd.2007.02.006

Streit WJ, 2002, GLIA, V40, P133, DOI 10.1002/glia.10154

Teasdale GM, 1998, NEUROSURGERY, V43, P723, DOI 10.1097/00006123-199810000-00001

Thompson HJ, 2005, J NEUROTRAUM, V22, P42, DOI 10.1089/neu.2005.22.42

Van Den Heuvel C, 2007, PROG BRAIN RES, V161, P303, DOI 10.1016/S0079-6123(06)61021-2

Wang HC, 2007, EXP NEUROL, V206, P59, DOI 10.1016/j.expneurol.2007.03.031

Zhao XR, 2003, GLIA, V44, P140, DOI 10.1002/glia.10283

Zhong CL, 2005, J NEUROTRAUM, V22, P266, DOI 10.1089/neu.2005.22.266

Zhou Y, 2005, MOL CELL PROTEOMICS, V4, P1471, DOI 10.1074/mcp.M500114-MCP200

NR 58

TC 111

Z9 126

U1 0

U2 17

PU ELSEVIER

PI AMSTERDAM

PA RADARWEG 29, 1043 NX AMSTERDAM, NETHERLANDS

SN 0006-8993

EI 1872-6240

J9 BRAIN RES

JI Brain Res.

PD AUG 21

PY 2008

VL 1226

BP 181

EP 191

DI 10.1016/j.brainres.2008.05.085

PG 11

WC Neurosciences

WE Science Citation Index Expanded (SCI-EXPANDED)

SC Neurosciences & Neurology

GA 351MG

UT WOS:000259427800021

PM 18582446

OA Green Accepted

DA 2024-03-03

ER

PT J

AU Sandhir, R

Onyszchuk, G

Berman, NEJ

AF Sandhir, Rajat

Onyszchuk, Gregory

Berman, Nancy E. J.

TI Exacerbated glial response in the aged mouse hippocampus following

controlled cortical impact injury

SO EXPERIMENTAL NEUROLOGY

LA English

DT Article

DE aging; astrocyte; CD11b; GFAP; Iba1; hippocampus; inflammation;

microglia; traumatic brain injury; S100B

ID TRAUMATIC BRAIN-INJURY; FIBRILLARY ACIDIC PROTEIN; GENE-EXPRESSION;

MICROGLIAL ACTIVATION; MESSENGER-RNA; AGING BRAIN; INFLAMMATION;

ASTROCYTES; YOUNG; MICE

AB Old age is associated with enhanced Susceptibility to and poor recovery from brain injury. An exacerbated microglial and astrocyte response to brain injury might be involved in poor outcomes observed in the elderly. The present study was therefore designed to quantitate the expression of markers of microglia and astrocyte activation using real-time RT-PCR, immunoblot and immunohistochemical analysis in aging brain in response to brain injury. We examined the hippocampus, a region that undergoes secondary neuron death, in aged (21-24 months) and adult (5-6 months) mice following controlled cortical impact (CCI) injury to the sensorimotor cortex. Basal mRNA expression of CD11b and Iba1, markers of activated microglia, was higher in aged hippocampus as compared to the adult. The mRNA expression of microglial markers increased and reached maximum 3 days post-injury in both adult and aged mice. but was higher in the aged mice at all time points studied, and in the aged mice the return to baseline levels was delayed. Basal mRNA expression of GFAP and S100B. markers of activated astrocytes, was higher in aged mice. Both markers increased and reached maximum 7 days post-injury. The mRNA expression of astrocyte markers returned to near basal levels rapidly after injury in the adult mice, whereas again in the aged mice return to baseline was delayed. Immunochemical analysis using Iba1 and GFAP antibodies indicated accentuated glial responses in the aged hippocampus after injury. The pronounced and prolonged activation of microglia and astrocytes in hippocampus may contribute to worse cognitive outcomes in the elderly following TBI. (c) 2008 Elsevier Inc. All rights reserved.

C1 [Sandhir, Rajat; Berman, Nancy E. J.] Univ Kansas, Med Ctr, Dept Anat & Cell Biol, Kansas City, KS 66160 USA.

[Sandhir, Rajat; Berman, Nancy E. J.] Univ Kansas, Med Ctr, Steve Palermo Nerve Regenerat Lab, Kansas City, KS 66160 USA.

[Onyszchuk, Gregory; Berman, Nancy E. J.] Univ Kansas, Med Ctr, Dept Neurosurg, Kansas City, KS 66160 USA.

C3 University of Kansas; University of Kansas Medical Center; University of

Kansas; University of Kansas Medical Center; University of Kansas;

University of Kansas Medical Center

RP Berman, NEJ (corresponding author), Univ Kansas, Med Ctr, Dept Anat & Cell Biol, 3901 Rainbow Blvd, Kansas City, KS 66160 USA.

EM nberman@kumc.edu

FU National Institute of Aging [AG026482, P30 NICHD HD 02528]

FX The authors acknowledge the assistance of Eugene Gregory in carrying out

the work and Eileen Roach with processing of the images. Special thanks

to Dr. Y.Y. He for his help with controlled cortical impact injury. The

study was supported in part by the Steve Palermo Endowment and grant

from the National Institute of Aging (AG026482 and P30 NICHD HD 02528).

CR Ashcroft GS, 2002, BIOGERONTOLOGY, V3, P337, DOI 10.1023/A:1021399228395

Berman NEJ, 1999, NEUROBIOL DIS, V6, P486, DOI 10.1006/nbdi.1999.0261

Bigler ED, 2002, AM J NEURORADIOL, V23, P255

Bramlett HM, 2007, PROG BRAIN RES, V161, P125, DOI 10.1016/S0079-6123(06)61009-1

Brown AW, 2004, NEUROREHABILITATION, V19, P37

Chung JH, 2008, AGEING RES REV, V7, P126, DOI 10.1016/j.arr.2008.01.001

Conde JR, 2006, J NEUROPATH EXP NEUR, V65, P199, DOI 10.1097/01.jnen.0000202887.22082.63

Conde JR, 2006, NEUROBIOL AGING, V27, P1451, DOI 10.1016/j.neurobiolaging.2005.07.012

Coronado VG, 2005, J HEAD TRAUMA REHAB, V20, P215, DOI 10.1097/00001199-200505000-00005

Deng XH, 2006, NEUROSCIENCE, V141, P645, DOI 10.1016/j.neuroscience.2006.04.016

DIAMOND MS, 1991, CELL, V65, P961, DOI 10.1016/0092-8674(91)90548-D

Felzien LK, 2001, BRAIN RES, V890, P137, DOI 10.1016/S0006-8993(00)03090-0

Ferrell Richard B, 2002, Curr Psychiatry Rep, V4, P354

Floyd CL, 2007, PROG BRAIN RES, V161, P61, DOI 10.1016/S0079-6123(06)61005-4

FRANEK KJ, 1998, CURR TREND IMMUNOL, V1, P29

Godbout JP, 2005, FASEB J, V19, P1329, DOI 10.1096/fj.05-3776fje

Goldstein FC, 2001, J CLIN EXP NEUROPSYC, V23, P739, DOI 10.1076/jcen.23.6.739.1028

GOSS JR, 1991, NEUROBIOL AGING, V12, P165, DOI 10.1016/0197-4580(91)90056-P

Hauwel M, 2005, BRAIN RES REV, V48, P220, DOI 10.1016/j.brainresrev.2004.12.012

Hukkelhoven CWPM, 2003, J NEUROSURG, V99, P666, DOI 10.3171/jns.2003.99.4.0666

Johnstone B, 1998, BRAIN INJURY, V12, P569, DOI 10.1080/026990598122331

Kim KY, 2004, NEUROBIOL AGING, V25, P491, DOI 10.1016/j.neurobiolaging.2003.07.005

Klein M, 1996, J NERV MENT DIS, V184, P459, DOI 10.1097/00005053-199608000-00002

Kreutzberg GW, 1996, TRENDS NEUROSCI, V19, P312, DOI 10.1016/0166-2236(96)10049-7

Kyrkanides S, 2001, J NEUROIMMUNOL, V119, P269, DOI 10.1016/S0165-5728(01)00404-0

Lee CK, 2000, NAT GENET, V25, P294, DOI 10.1038/77046

LETARTE P, 2006, TRAUMA, P397

Liu B, 2003, ENVIRON HEALTH PERSP, V111, P1065, DOI 10.1289/ehp.6361

Liu B, 2002, ANN NY ACAD SCI, V962, P318, DOI 10.1111/j.1749-6632.2002.tb04077.x

Lucas SM, 2006, BRIT J PHARMACOL, V147, pS232, DOI 10.1038/sj.bjp.0706400

Maeda J, 2007, BRAIN RES, V1157, P100, DOI 10.1016/j.brainres.2007.04.054

Mahesh VB, 2006, MOL CELL ENDOCRINOL, V246, P1, DOI 10.1016/j.mce.2005.11.017

Major DE, 1997, NEUROBIOL AGING, V18, P523, DOI 10.1016/S0197-4580(97)00102-4

Marchetti B, 2005, TRENDS PHARMACOL SCI, V26, P517, DOI 10.1016/j.tips.2005.08.007

MOHINDRA S, SURG NEUROL IN PRESS

Mrak RE, 2005, NEUROBIOL AGING, V26, P349, DOI 10.1016/j.neurobiolaging.2004.05.010

Myer DJ, 2006, BRAIN, V129, P2761, DOI 10.1093/brain/awl165

Ohsawa K, 2000, J CELL SCI, V113, P3073

Onyszchuk G, 2008, J NEUROTRAUM, V25, P153, DOI 10.1089/neu.2007.0430

Onyszchuk G, 2007, J NEUROSCI METH, V160, P187, DOI 10.1016/j.jneumeth.2006.09.007

Pelinka LE, 2004, J NEUROTRAUM, V21, P1553, DOI 10.1089/0897715042441846

Pertusa M, 2007, J NEUROCHEM, V101, P794, DOI 10.1111/j.1471-4159.2006.04369.x

PETCU EB, 2007, GERONTOLOGY

Pfaffl MW, 2002, NUCLEIC ACIDS RES, V30, DOI 10.1093/nar/30.9.e36

Popa-Wagner A, 2007, CURR NEUROVASC RES, V4, P216

Rapoport MJ, 2006, AM J GERIAT PSYCHIAT, V14, P456, DOI 10.1097/01.JGP.0000199339.79689.8a

RINK A, 1995, AM J PATHOL, V147, P1575

Sandhir R, 2004, NEUROSCI LETT, V369, P28, DOI 10.1016/j.neulet.2004.07.032

Sawada M, 2008, NEURODEGENER DIS, V5, P254, DOI 10.1159/000113717

Schmidt OliverI., 2004, European Journal of Trauma, P135, DOI DOI 10.1007/S00068-004-1394-9

SCHOLZ J, 2008, PAIN

Schwartz M, 2006, TRENDS NEUROSCI, V29, P68, DOI 10.1016/j.tins.2005.12.005

Sheffield LG, 1998, NEUROBIOL AGING, V19, P47, DOI 10.1016/S0197-4580(97)00168-1

Stolzing A, 2005, REDOX REP, V10, P207, DOI 10.1179/135100005X70198

Streit WJ, 2004, GLIA, V45, P208, DOI 10.1002/glia.10319

Sugama S, 2003, BRAIN RES, V964, P288, DOI 10.1016/S0006-8993(02)04085-4

TOPP KS, 1989, GLIA, V2, P201, DOI 10.1002/glia.440020309

WHITEGBADEBO D, 1993, J NEUROTRAUM, V10, P297, DOI 10.1089/neu.1993.10.297

Wilhelmsson U, 2004, J NEUROSCI, V24, P5016, DOI 10.1523/JNEUROSCI.0820-04.2004

Wu DY, 2007, J IMMUNOL, V179, P4829, DOI 10.4049/jimmunol.179.7.4829

Yoshida T, 1996, NEUROSCI LETT, V215, P107, DOI 10.1016/S0304-3940(96)12966-9

Zhu W, 2003, J GERONTOL A-BIOL, V58, P117

NR 62

TC 141

Z9 160

U1 2

U2 16

PU ACADEMIC PRESS INC ELSEVIER SCIENCE

PI SAN DIEGO

PA 525 B ST, STE 1900, SAN DIEGO, CA 92101-4495 USA

SN 0014-4886

EI 1090-2430

J9 EXP NEUROL

JI Exp. Neurol.

PD OCT

PY 2008

VL 213

IS 2

BP 372

EP 380

DI 10.1016/j.expneurol.2008.06.013

PG 9

WC Neurosciences

WE Science Citation Index Expanded (SCI-EXPANDED)

SC Neurosciences & Neurology

GA 351ZG

UT WOS:000259464200014

PM 18692046

OA Green Accepted

DA 2024-03-03

ER

PT J

AU Hilton, GD

Stoica, BA

Byrnes, KR

Faden, AI

AF Hilton, Genell D.

Stoica, Bogdan A.

Byrnes, Kimberly R.

Faden, Alan I.

TI Roscovitine reduces neuronal loss, glial activation, and neurologic

deficits after brain trauma

SO JOURNAL OF CEREBRAL BLOOD FLOW AND METABOLISM

LA English

DT Article
[truncated: 5,785,590 more chars]
